# Platform for Orthogonal *N*-Cysteine-Specific Protein Modification Enabled by Cyclopropenone Reagents

**DOI:** 10.1021/jacs.2c02185

**Published:** 2022-06-05

**Authors:** Alena Istrate, Michael B. Geeson, Claudio D. Navo, Barbara B. Sousa, Marta C. Marques, Ross J. Taylor, Toby Journeaux, Sebastian R. Oehler, Michael R. Mortensen, Michael J. Deery, Andrew D. Bond, Francisco Corzana, Gonzalo Jiménez-Osés, Gonçalo J. L. Bernardes

**Affiliations:** † Yusuf Hamied Department of Chemistry, University of Cambridge, Lensfield Road, CB2 1EW Cambridge, United Kingdom; ‡ Center for Cooperative Research in Biosciences (CIC bioGUNE), Basque Research and Technology Alliance (BRTA), Bizkaia Technology Park, Building 800, 48160 Derio, Spain; ¶ Instituto de Medicina Molecular, João Lobo Antunes, Faculdade de Medicina da Universidade de Lisboa, Av. Prof. Egas Moniz, 1649-028 Lisboa, Portugal; § Department of Chemistry and Applied Biosciences, ETH Zürich, Vladimir-Prelog-Weg 3, 8093 Zürich, Switzerland; ∥ Cambridge Centre for Proteomics, Gleeson Building, University of Cambridge, Tennis Court Road, CB2 1QR Cambridge, United Kingdom; ⊥ Departamento de Química, Universidad de La Rioja, Centro de Investigación en Síntesis Química, 26006 Logroño, Spain; ○ Ikerbasque, Basque Foundation for Science, 48013 Bilbao, Spain

## Abstract

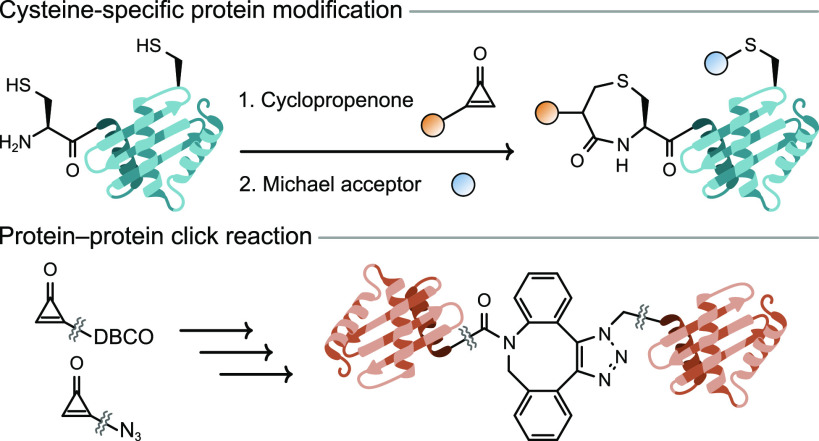

Protein conjugates are valuable tools for studying biological processes or producing therapeutics, such as antibody–drug conjugates. Despite the development of several protein conjugation strategies in recent years, the ability to modify one specific amino acid residue on a protein in the presence of other reactive side chains remains a challenge. We show that monosubstituted cyclopropenone (CPO) reagents react selectively with the 1,2-aminothiol groups of N-terminal cysteine residues to give a stable 1,4-thiazepan-5-one linkage under mild, biocompatible conditions. The CPO-based reagents, all accessible from a common activated ester CPO-pentafluorophenol (**CPO-PFP**), allow selective modification of N-terminal cysteine-containing peptides and proteins even in the presence of internal, solvent-exposed cysteine residues. This approach enabled the preparation of a dual protein conjugate of **2**×**cys-GFP**, containing both internal and N-terminal cysteine residues, by first modifying the N-terminal residue with a CPO-based reagent followed by modification of the internal cysteine with a traditional cysteine-modifying reagent. CPO-based reagents enabled a copper-free click reaction between two proteins, producing a dimer of a de novo protein mimic of IL2 that binds to the β-IL2 receptor with low nanomolar affinity. Importantly, the reagents are compatible with the common reducing agent dithiothreitol (DTT), a useful property for working with proteins prone to dimerization. Finally, quantum mechanical calculations uncover the origin of selectivity for CPO-based reagents for N-terminal cysteine residues. The ability to distinguish and specifically target N-terminal cysteine residues on proteins facilitates the construction of elaborate multilabeled bioconjugates with minimal protein engineering.

## Introduction

Protein conjugates are important tools for creating valuable therapeutics, such as antibody–drug conjugates (ADCs)^1,2^ and PEGylated proteins,^3^ building new functionalized materials,^4^ and studying biological processes.^5,6^ Among the various strategies used for protein conjugation, modification of naturally occurring amino acids remains the method of choice because it offers the advantage of straightforward accessibility without the need for sequence alterations by means of genetic methods.^7^ Ideally, conjugation reactions should proceed with complete chemo- and site-selectivity to generate well-defined protein constructs, which is a crucial requirement for several applications such as ADCs.^1,2^ Similarly, such transformations should occur rapidly in mild aqueous solutions at room temperature and physiological pH. Although many protein conjugation strategies have been developed over recent years, the ability to modify one specific amino acid on a protein in the presence of other side chains with similar reactivity remains a challenge.^7^ Protection of particularly reactive amino acids such as cysteine^8^ or extensive sequence engineering with the introduction of specific tags for enhanced reactivity^9^ is often required in order to achieve selectivity for the intended residue.

Lysine^10−12^ and cysteine^9,13−15^ are the most commonly targeted proteinogenic amino acids for bioconjugation because they are nucleophilic under physiological conditions. Native lysine residues are very convenient targets, but they are abundant on protein surfaces, so it is difficult to achieve a high degree of selectivity for a given residue.^10^ Conversely, cysteine residues are less abundant in proteins (<2%) and commonly less solvent-exposed, which makes them an excellent target for site-selective conjugation.^16^ However, when cysteine residues are relied on for protein modification, there are several factors that must be taken into account. Specifically, cysteine residues often form disulfide bonds that are critical for a protein’s structure, and modification of such residues can lead to a loss of protein function. Moreover, many surface-exposed endogenous cysteine residues are directly involved in the catalytic activity of the protein and thus cannot be exploited for modification. Therefore, methods that can distinguish one cysteine residue from another within one protein could enable the construction of functional and well-defined biomolecule conjugates without the need for extensive genetic engineering or the incorporation of unnatural amino acids.

**Figure 1:**
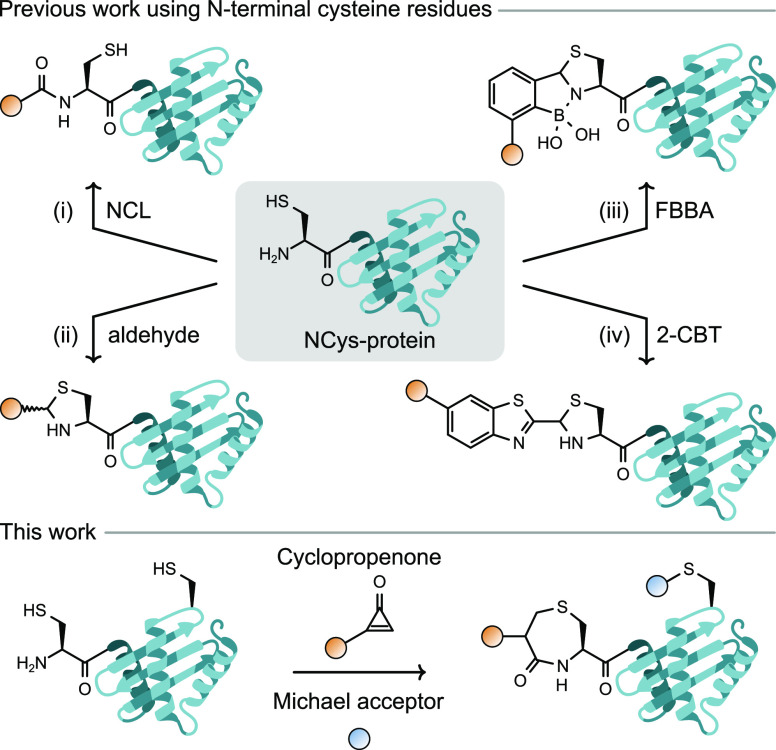
Overview of recent methods for targeting N-terminal cysteine residues for bioconjugation and the site-specific dual protein conjugation described here. NCL, native chemical ligation; 2-CBT, 2-cyanobenzothiazol; FBBA, formyl benzeno boronic acid.

The most reliable strategy to differentiate one cysteine in the presence of other thiol groups is to target an N-terminal cysteine residue (NCys). Several methods for selective NCys modification have been developed, including reaction with thioesters via native chemical ligation (NCL) or condensation with aromatic aldehydes or 2-cyanobenzothiazole derivatives (Figure 1).^17^ NCL enables linking of protein or peptide fragments in a robust and chemoselective manner through transthioesterification and S-to-N acyl exchange (Figure 1).^18,19^ However, this method is rarely used to produce protein conjugates because of difficulties in preparation and lack of stability of suitable thioester reagents. The reaction of 1,2-aminothiols with aldehydes to form thiazolidine has also been explored as a strategy for NCys modification (Figure 1).^20^ Neri and co-workers have successfully applied this approach for site-specific coupling of cytotoxic aldehydes to tumor-targeting antibodies, which produced homogeneous conjugates that were then used for targeted delivery and slow release of the cytotoxic component.^21^ However, this reaction requires long incubation times (>48 h), occurs under acidic conditions (pH 4–5), and is typically performed with a large excess of the aldehyde derivative. These limitations can be addressed by using formyl benzeno boronic acids (FBBA) that stabilize thiazolidine formation through N→B coordination (Figure 1). Recently, FBBA reagents have been used to rapidly modify several model NCys-containing peptides at neutral pH.^22,23^ This reaction is reversible, and the product is unstable in an acidic environment (pH < 6) or in the presence of nucleophiles (e.g., free cysteine or benzyl hydroxylamine). It is however possible to use a thiazolidino boronate intermediate which undergoes an intramolecular acyl transfer to give more stable N-acyl thiazolidines.^24^

Another N-terminal Cys-labeling reaction was inspired by the final step of the chemical synthesis of D-luciferin^25^ and is based on the condensation of free cysteine with 2-cyanobenzothiazol (CBT) (Figure 1). After Rao and co-workers first demonstrated the potential of this reaction for NCys modification,^26^ the approach has been widely used in site-specific protein labeling and molecular imaging. This method has major advantages for bioconjugation because of its efficiency, biocompatibility, and the stability of the resulting luciferin linkage.^27^ However, 2-cyanobenzothiazol derivatives also react quickly (although reversibly) with simple thiols. As a result, when using excess CBT to ensure complete conjugation, other reduced protein thiols must be protected. Therefore, alternative bioconjugation reagents for fast and selective labeling of N-terminal cysteine residues are still required to enable the construction of complex protein conjugates of well-defined structures.

In this work, the cyclopropenone (CPO) functional group was developed for site-specific modification of N-terminal cysteine residues on peptides and proteins. The three-membered rings of cyclopropenones feature significant ring-strain, though the aromatic character of the ring^28^ renders it remarkably stable. The strain and large dipole moment allow this functional group to participate in cycloaddition and ring-opening reactions,^29,30^ and as α,β-unsaturated ketones, cyclopropenones also act as electrophiles in 1,2- and 1,4-nucleophilic addition reactions. Several natural products contain cyclopropenone functional groups, highlighting their biocompatibility and stability under physiological conditions.^31,32^ Cyclopropenone-containing molecules have been featured in some previous biological applications, for example, in the design of selective protease inhibitors^33^ or as components for bioorthogonal reactions on proteins.^34−36^ The latter work, pioneered by Prescher and co-workers, focused on the reaction of CPO-containing molecules (installed on lysine residues with NHS-ester chemistry) with phosphines as a strategy for bioorthogonal reactions and real-time cell imaging.^37^ Finally, reactions of N-terminal cysteine residues in peptides with hybrid aminosulfhydryl-stapling reagents lead to products containing 1,4-thiazepan-5-one rings; due to the NHS-ester functional group present in those reagents, internal stapling of cysteine and lysine residues was targeted as a conjugation strategy.^38^

Here, we report the efficient and selective reaction of monosubstituted cyclopropenone-containing reagents with the 1,2-aminothiol groups of N-terminal cysteine residues (Figure 1). Importantly, the selectivity exhibited by CPO-based reagents toward N-terminal cysteine residues enables sequential and site-specific dual-modification of proteins. Such selectivity is also demonstrated to be operational in complex mixtures of proteins that contain internal or N-terminal cysteine residues and in the presence or absence of DTT.

## Results and Discussion

### Monosubstituted Cyclopropenones Are Efficient Reagents for N-Terminal Cysteine Labeling

**Figure 2:**
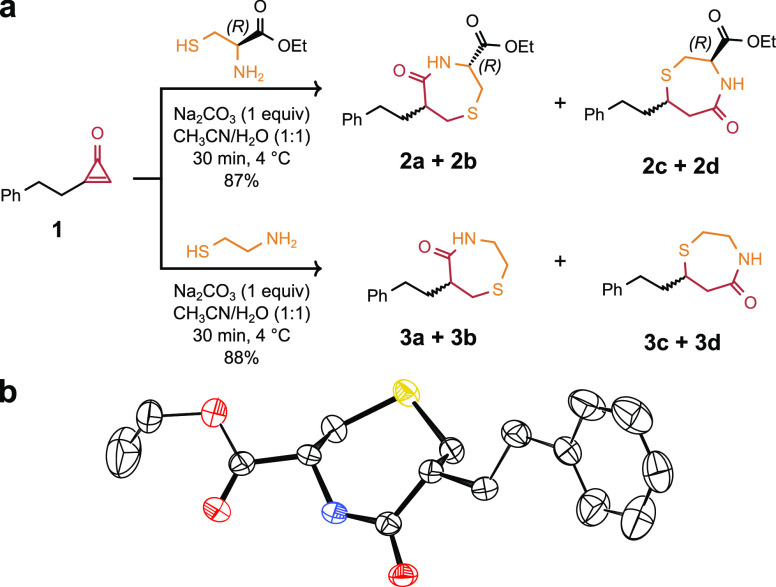
(a) Reaction of cyclopropenone **1** with l-cysteine ethyl ester hydrochloride and cysteamine. (b) Oak Ridge thermal ellipsoid plot (ORTEP) of compound **2a** with thermal ellipsoids at the 50% probability level and hydrogen atoms omitted for clarity.

Our investigations began with the synthesis of a model cyclopropenone in order to assess the stability of this functional group in aqueous buffers. Along the lines of a literature procedure,^39^ 2-phenylethylcyclopropenone (**1**) was prepared from commercially available 4-phenyl-1-butyne by (i) treatment with TMSCF_3_, a formal source of difluorocarbene, to afford the corresponding 3,3-difluorocyclopropene derivative^40^ followed by (ii) hydrolysis on wet silica gel.^35,39^ Cyclopropenone **1** showed excellent stability after treatment with phosphate buffers (50 mM, pH 7–8) at 37 °C for 7 days (Figure S7). Next, the reaction of cyclopropenone **1** with l-cysteine ethyl ester was tested in the presence of base (Na_2_CO_3_) at 4 °C. This low temperature was found to be necessary with small, highly accessible nucleophiles such as amino acids and peptides in order to avoid side reactions and monitor the kinetics of the reaction, although it was not necessary for proteins. The reaction resulted in four isomeric compounds **2a**–**d** in 87% yield after 30 min in aqueous solution. Analysis by liquid chromatography (LC)–mass spectrometry (MS) and NMR spectroscopy showed that the products are two pairs of diastereomeric regioisomers of a 1,4-thiazepan-5-one derivative, a stable seven-membered ring (Figure 2) that results from a ring-expansion of the cyclopropenone group. Isomer **2a** was isolated and characterized by X-ray crystallography, revealing its stereochemistry and confirming its structure (Figure 2). To study the reaction further and confirm its outcome on a simpler, nonchiral model, cysteamine (**CA**) was treated with cyclopropenone **1**. As expected, the reaction yielded two pairs of enantiomeric regioisomers (Figure 2) that were separated by column chromatography and characterized by NMR spectroscopy.

### Reaction Kinetics and Selectivity of CPO-Based Reagents

Next, the reaction kinetics of l-cysteine ethyl ester with CPO-based reagents were examined, and the limits of chemoselectivity were established. To evaluate the reaction kinetics, the HPLC chromatogram peak area of starting cyclopropenone **1** was monitored as a function of time over the course of the reaction (Figure S6). The second-order rate constant for this reaction was determined to be 3.0 M^–1^·s^–1^ at 4 °C, comparable to the value reported for the CBT–cysteine reaction (9.19 M^–1^·s^–1^) and strain-promoted azide–alkyne cycloaddition reactions (10^–2^–1 M^–1^·s^–1^) performed at higher temperatures (37 °C).^41^ In fact, the rate constant for the reaction between cyclopropenone **1** and cysteine extrapolated to 37 °C would be 67 M^–1^·s^–1^, which supersedes the aforementioned values.

A screen was performed to establish whether CPO-based reagent **1** underwent a reaction with other biologically relevant nucleophiles, including lysine, serine, threonine, tyrosine, glutathione, and cysteine methyl ester with a *tert*-butoxycarbonyl protected amino group (Boc-Cys-OMe). As shown by LC–MS, compound **1** did not react with lysine, serine, threonine, or tyrosine but showed excellent selectivity for l-cysteine. In addition, these amino acids did not interfere with the reaction between **1** and cysteine when present in the reaction mixture (Figures S9–S12). With the N-terminus protected, Boc-Cys-OMe nevertheless underwent a reaction with compound **1** to form a complex mixture of products, although the reaction was significantly slower compared to l-cysteine ethyl ester with an unprotected amino group at its N-terminus (Figure S14). In fact, when a stoichiometric mixture of both N-protected Boc-Cys-OMe and N-unprotected l-cysteine ethyl ester was treated with CPO-based reagent **1**, high selectivity was observed toward the 1,2-aminothiol group of l-cysteine ethyl ester, resulting in almost exclusive formation of 1,4-thiazepan-5-one products **2a**–**d** (Figure S14). Importantly, glutathione, the most abundant low-molecular-weight thiol in cells, did not react with CPO-based reagent **1** and did not interfere with the reaction between **1** and l-cysteine ethyl ester (Figure S13). CPO-based reagents have a clear advantage over 2-cyanobenzothiazole (CBT) reagents, which react with glutathione and other thiol nucleophiles.^26^

### CPO-Based Reagents with a Range of Functionality Are Accessible from **CPO-PFP**

**Scheme 1:**
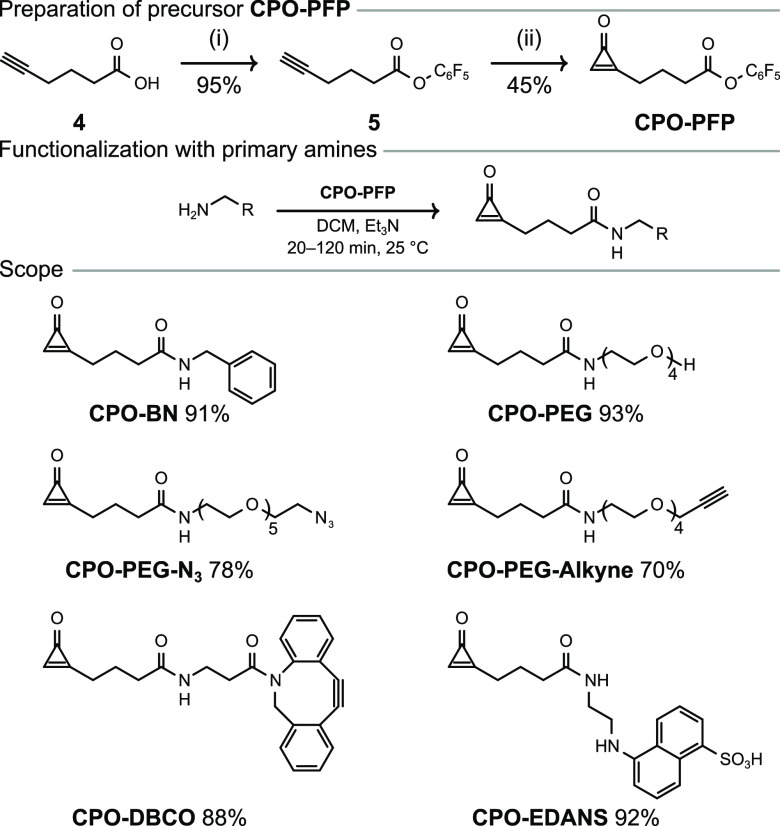
Synthesis of Cyclopropenone-Based Reagents with Different Functional Groups Conditions: (i) PTFTFA, DIPEA, DCM, 1 h, 25 °C. (ii) 1. TMSCF_3_, NaI, THF, 25 °C, 48 h. 2. SiO_2_, CHCl_3_.

Before the applicability of the reaction on peptides and proteins was tested, several cyclopropenone-based reagents were designed and synthesized with different functionalities (Scheme 1). The synthetic protocol began with commercially available 5-hexynoic acid **4**, which was converted to the corresponding pentafluorophenol (PFP) ester **5** with pentafluorophenyl trifluoroacetate (PTFTFA, Scheme 1). Ester **5** was subjected to cyclopropenation, affording the corresponding CPO-containing activated ester **CPO-PFP**, which is an easy-to-handle, stable (>6 months) white solid that can be accessed on a large scale. **CPO-PFP** can be used to append a cyclopropenone unit to a compound of interest if it contains a primary amine via a simple amide-bond-forming procedure in a fast and efficient manner. The reaction conditions of the coupling were optimized using benzylamine, providing **CPO-BN** in high yield (91%). This procedure was used to prepare several CPO-containing derivatives of interest for peptide and protein bioconjugation reactions. Cyclopropenone-based reagents were prepared that contained a poly(ethylene glycol) unit (**CPO-PEG**), a fluorescent dye (**CPO-EDANS**), and several derivatives with functional groups of relevance to click chemistry (**CPO-PEG-Alkyne**, **CPO-N**_**3**_, and **CPO-DBCO**). Overall, intermediate **CPO-PFP** provides ready access to several CPO-based reagents by treatment with amine-bearing molecules of interest at room temperature in high yields and short reaction times (Scheme 1).

### CPO-Based Reagents Selectively Modify N-Terminal Cysteine Residues on Peptides

**Figure 3:**
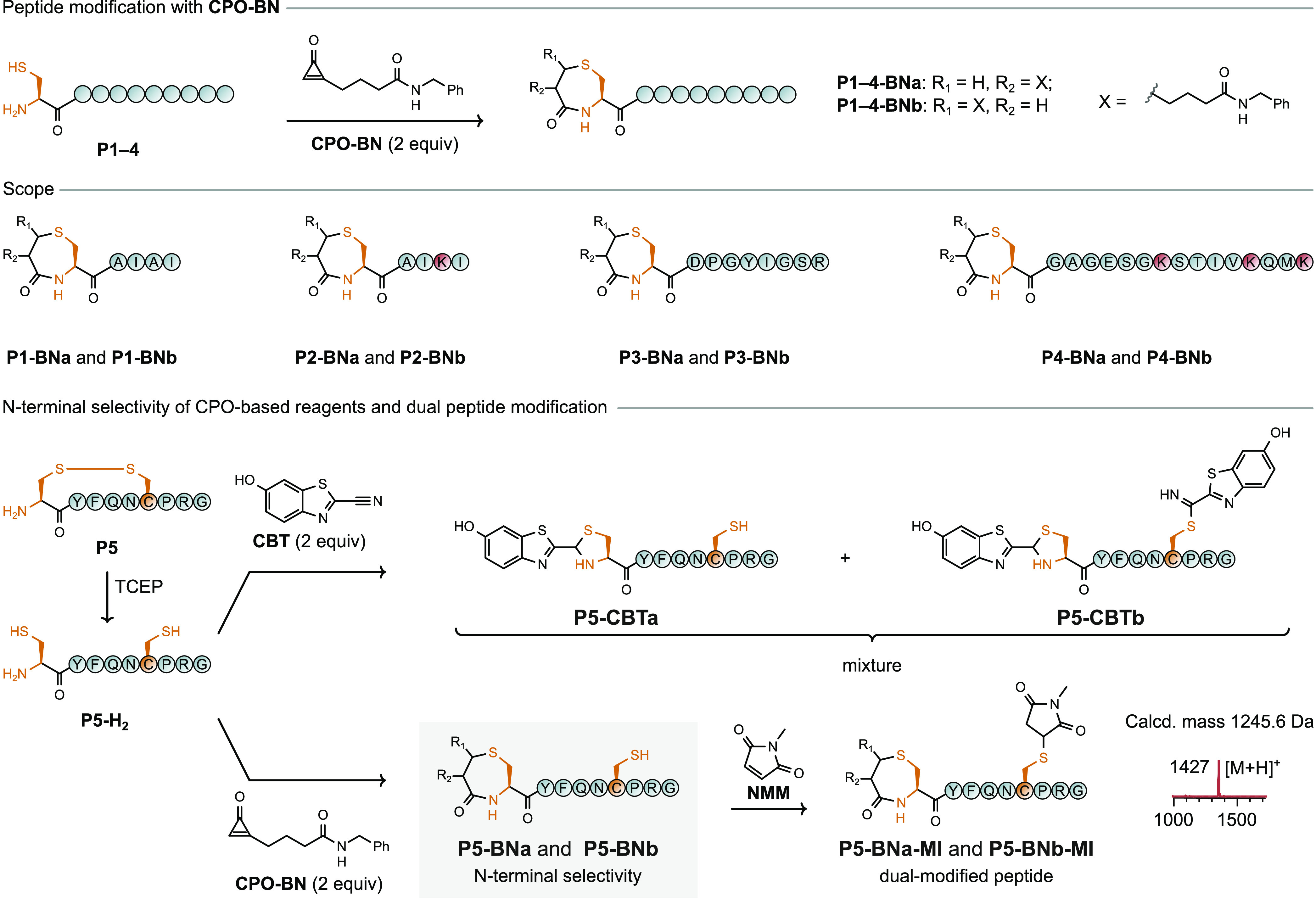
Chemoselective modification of 1,2-aminothiols on peptides. (a) Site-specific modification of peptides **P1**–**P4** by **CPO-BN**. Modification occurs only on the N-terminal cysteine residue. (b) **CPO-BN** selectively modifies the N-terminal cysteine residue of vasopressin (**P5**) and leaves the internal cysteine residue unmodified and accessible for further functionalization.

The high reactivity and selectivity of compound **1** toward 1,2-aminothiols prompted us to investigate the ability of cyclopropenone-based reagents to modify N-terminal cysteine residues in peptides. Investigations began with two unprotected 5-mer peptides with N-terminal cysteine residues: CAIAI (**P1**) and CAIKI (**P2**). Notably, **P2** also contains a lysine residue and therefore provides a test for the selectivity of CPO-based reagents for cysteine versus lysine residues in peptides. Treatment of both peptides (2 mM) with **CPO-BN** (2 equiv) in NaP_i_ buffer (20 mM, pH 7)/acetonitrile resulted in complete conversion into the expected products after 1 h at 4 °C (Figure 3 and Figures S15 and S17). Similarly, modification of peptides **P1** and **P2** with **CPO-PEG** resulted in the formation of the expected PEGylated products, as confirmed by LC–MS analysis (Figures S16 and S18).

Next, more complex peptides that contained multiple nucleophilic side chains were treated with CPO-based reagents. Initial efforts focused on laminin-derived synthetic peptide **P3**, an inhibitor of tumor growth.^42^ This peptide contains nine amino acid residues, including an N-terminal cysteine, tyrosine, serine, asparagine, and arginine. Application of the optimized N-terminal cysteine modification protocol resulted in full conversion of the starting peptide after 1 h, as confirmed by LC–MS (Figure 3 and Figure S19). The GTP-binding protein fragment (**P4**) is conceivably a more challenging substrate with 16 residues and multiple nucleophilic side chains (one N-terminal cysteine, three lysine, two serine, one threonine, and one methionine residue). Nevertheless, treatment of peptide **P4** with **CPO-BN** (2 equiv) resulted in full conversion to products **P4–BNa** and **P4–BNb** within 1 h at 4 °C (Figure 3 and Figure S20, respectively). No signal for double addition of **CPO-BN** was observed by mass spectrometry, highlighting the chemoselectivity of the protocol for cysteine residues over alternatives.

To test whether chemoselectivity between N-terminal and internal cysteine residues held at the peptide level, a peptide that contains both was treated with **CPO-BN**. Vasopressin (**P5**), a 9-mer cyclic peptide by virtue of a disulfide bond, was reduced with TCEP to generate a linear peptide (**P5-H**_**2**_) with cysteine residues in positions 1 (N-terminal) and 6 (internal). In control experiments without the addition of the reducing agent, vasopressin reacted with neither **CPO-BN** nor *N*-methylmaleimide (**NMM**), another common reagent for cysteine conjugation. In the course of the reduction of **P5** to **P5-H**_**2**_, care was taken to ensure that excess TCEP was removed from the reaction mixture before subsequent labeling (e.g., by using immobilized TCEP or limiting the amount used to 1 equiv) due to the incompatibility of phosphines with cysteine-labeling reagents, including cyclopropenones.^34,35^ Reduced peptide **P5-H**_**2**_ was treated with either **NMM** or **CPO-BN** (2 equiv) to compare the selectivity of the two approaches (Figure 3). As expected, **NMM** did not distinguish between the two cysteine residues, resulting in modification at both positions (Figure S22). In contrast, treatment of the reduced vasopressin peptide **P5-H**_**2**_ with **CPO-BN** resulted in selective modification of the N-terminal cysteine residue (Figure 3 and Figure S24). Further experiments confirmed that the internal cysteine residue was still available for modification after the N-terminal cysteine residue was labeled with **CPO-BN**; treatment of CPO-modified vasopressin **P5-BN** with **NMM** led to quantitative modification of the internal cysteine residue (Figure 3 and Figure S25), and analysis by LC–MS/MS confirmed the site specificity of the modifications (Figure S26).

CBT-based reagents (Figure 1), arguably the state-of-the-art method for N-terminal cysteine modification,^26^ were tested under the same conditions to provide a direct comparison between CPO- and CBT-based reagents for selectivity toward the N-terminal cysteine residue of vasopressin. In contrast to the case with **CPO-BN**, treatment of **P5-H**_**2**_ with 2-cyano-6-hydroxybenzothiazole (CBT) afforded a mixture of single- and double-modified peptides (Figure 3 and Figure S23). This case study highlights the potential for cyclopropenone-based reagents to offer increased site-specificity for N-terminal cysteine residues compared to the CBT analogues.

### CPO-Based Reagents React Exclusively with N-Terminal Cysteine Residues on Proteins

With excellent selectivity and reaction kinetics demonstrated at the peptide level, attention turned to proteins. Fortunately, recombinant proteins with N-terminal cysteine residues are widely used for NCL, and so various approaches have been developed for their direct production.^43−46^ A recombinant enhanced green fluorescent protein containing an N-terminal cysteine residue (**cys-GFP**) was produced by engineering a variant with the tobacco etch virus (TEV) protease recognition sequence (ENLYFQ↓C; arrow indicates the cleavage site) introduced after the His_6_ purification tag at the N-terminus. In this way, the cleavage of the expressed protein by TEV protease simultaneously removed the His_6_ tag and generated **cys-GFP**.

**Figure 4:**
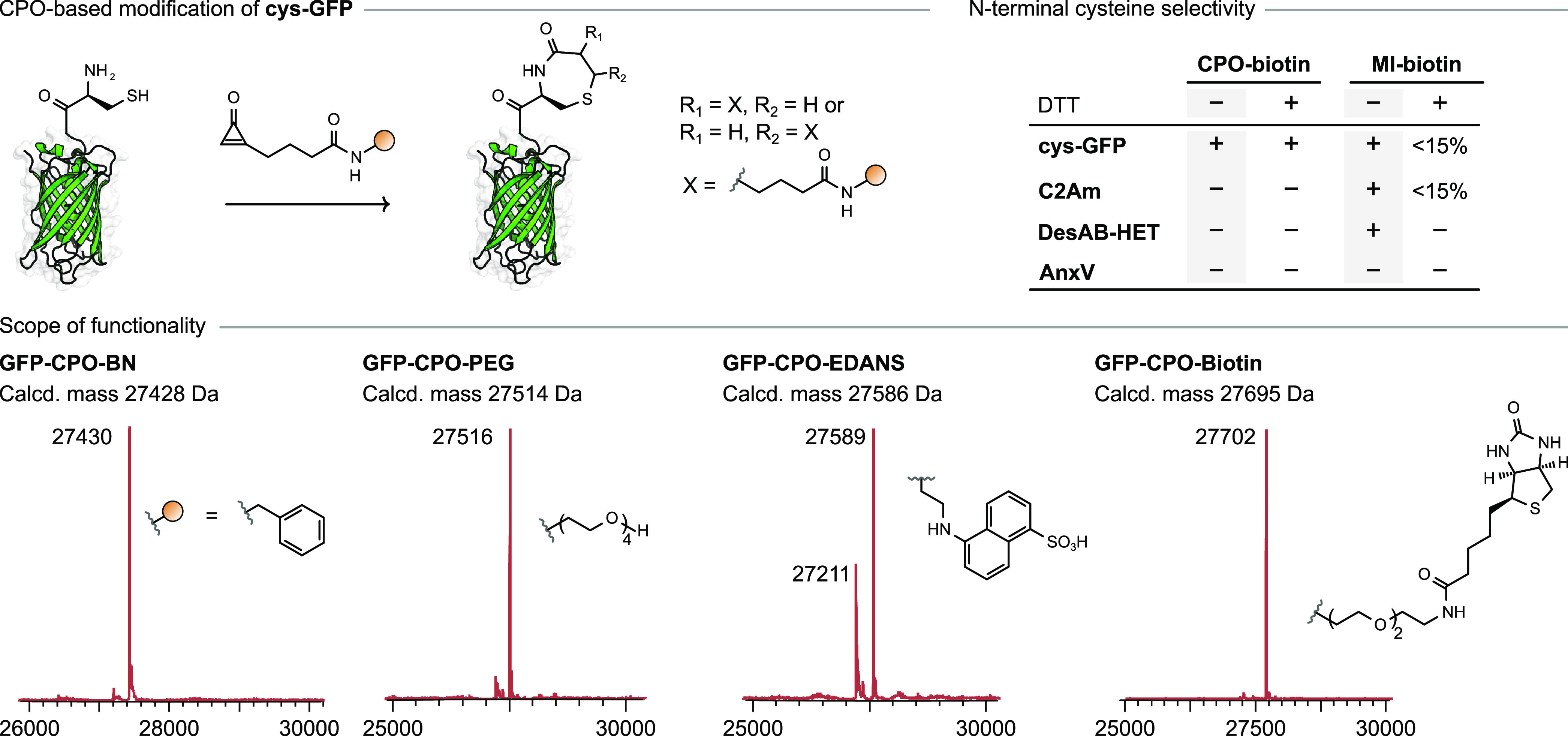
Top left: Chemoselective modification of 1,2-aminothiols on proteins. Bottom: Site-specific bioconjugation of **cys-GFP** with **CPO-BN**, **CPO-PEG**, **CPO-EDANS**, and **CPO-biotin**. Top right: Treatment of a mixture of **cys-GFP**, **C2Am**, **AnxV**, and **DesAB-HET** (5 μM each; N-terminal residues C, G, A, and M, respectively) with **CPO-biotin** or **MI-biotin** (50 equiv) in NaP_i_ buffer (20 mM, pH 7.0) with or without DTT (500 equiv).

Incubation of **cys-GFP** with **CPO-BN** (100 equiv) in NaP_i_ buffer (20 mM, pH 7.0) at 25 °C for 2 h gave the desired conjugate **GFP-CPO-BN** with high efficiency, as confirmed by LC–MS spectrometry (Figure 4 and Figure S38). Because proteins were modified at lower concentrations (ca. 30 μM) than peptides, higher stoichiometric equivalents of CPO-based reagents were used to ensure short reaction times (2–4 h). Following modification, digestion of **GFP-CPO-BN** with trypsin and subsequent analysis by LC–MS/MS confirmed the site of modification (Figure S41). The protein conjugate **GFP-CPO-BN** also displayed excellent stability; after incubation with glutathione (5 mM, 24 h, 37 °C), no deconjugation was observed (Figure S40). Molecular dynamics simulations (0.5 μs) were performed on the four possible conjugates of **GFP-CPO-BN** (Figure S66). The root-mean-square deviation values of the peptide backbone in all complexes ranged from 1.21 to 2.67 Å, suggesting that the addition of **CPO-BN** does not cause significant structural modifications to the protein. This theoretical approach was validated with experiments for other proteins by using circular dichroism spectroscopy.

After demonstrating the chemoselectivity and efficiency of **CPO-BN** for N-terminal cysteine bioconjugation, we expanded the scope of the reaction to other CPO-based reagents. Testing **CPO-PEG** and **CPO-EDANS** under the same reaction conditions as used for **CPO-BN** resulted in successful conversion of **cys-GFP** to the expected products (Figure 4 and Figures S42 and S43). In the case of **CPO-EDANS**, the lower conversion (70%) ostensibly arises from the poor solubility of the reagent. In order to determine whether purification of CPO-based reagents was necessary for protein conjugation, we tested out a telescoped procedure. **CPO-PFP** was mixed with a commercially available amine-functionalized derivative of biotin, providing **CPO-biotin** as assayed by LC–MS (Figure S47). Then, without any additional purification steps, the resulting solution was directly added to **cys-GFP** (100 equiv of **CPO-biotin** to 1 equiv of **cys-GFP**), and the reaction was monitored by LC–MS. To our delight, conversion of **cys-GFP** to the expected conjugate was complete after incubation for 2 h (Figure 4 and Figure S45). Thus, in this case there is no need to purify the CPO-based reagent before the protein-labeling step which improves the throughput of the protein-modification workflow.

In the course of our studies employing **cys-GFP**, it was found that the addition of dithiothreitol (DTT) to the protein stock solution was necessary to maintain the reactivity of the cysteine residue, presumably due to high susceptibility of the N-terminal cysteine residue to undergo oxidation and lead to protein dimerization. Nevertheless, an excess of DTT (500 equiv) in the reaction mixture did not hinder the bioconjugation reaction of CPO-based reagents with the N-terminal cysteine residue. In contrast, the reaction of **cys-GFP** with the traditional maleimide-based reagent **MI-biotin** under otherwise identical conditions produced very low conversion (∼13%; Figure S46), presumably a result of the incompatibility of maleimides with excess DTT.^47^ Once again, this highlights the selectivity of the CPO-based reagents toward 1,2-aminothiols; 100 equiv of the reagent was enough to afford the desired protein conjugate even in the presence of an excess of DTT (Figure 4).

Next, we explored whether CPO-based reagents could selectively label a N-terminal cysteine-containing protein in the presence of other internal cysteine-containing proteins. A mixture of four proteins was prepared: **cys-GFP** (three free cysteine residues, including one N-terminal), an engineered variant of the C2A domain of synaptotagmin-I (**C2Am**, one free cysteine residue), annexin V (**AnxV**, one free cysteine residue), and an engineered variant of a nanobody (**DesAB-HET**, one free cysteine residue), and it was incubated with **CPO-biotin** or the analogous maleimide-based reagent, **MI-biotin**. In the case of **CPO-biotin**, successful conjugation was observed with **cys-GFP** whereas proteins **C2Am**, **AnxV**, and **DesAB-HET** were left unchanged. Similar results were observed regardless of whether DTT (500 equiv) was present in the reaction mixture (Figure 4 and Figures S50 and S52). In contrast, **MI-biotin** did not exhibit any selectivity and fully modified three out of four proteins (**cys-GFP**, **C2Am**, and **DesAB-HET**) when DTT was absent from the reaction mixture (Figure 4 and Figure S51). **AnxV** was not modified because it usually requires a large excess of reagents, higher temperatures, or longer reaction times for the cysteine modification to proceed.^48^ As expected, in the presence of DTT, only minor modification of proteins (0–15%) with **MI-biotin** was observed (Figure 4 and Figure S53). Overall, these data confirm the ability of CPO-based reagents to orthogonally label N-terminal cysteine residues in complex mixtures of other proteins bearing reactive internal cysteine residues and in the presence of DTT.

### Site-Specific Cysteine Labeling of a Single Protein

**Figure 5:**
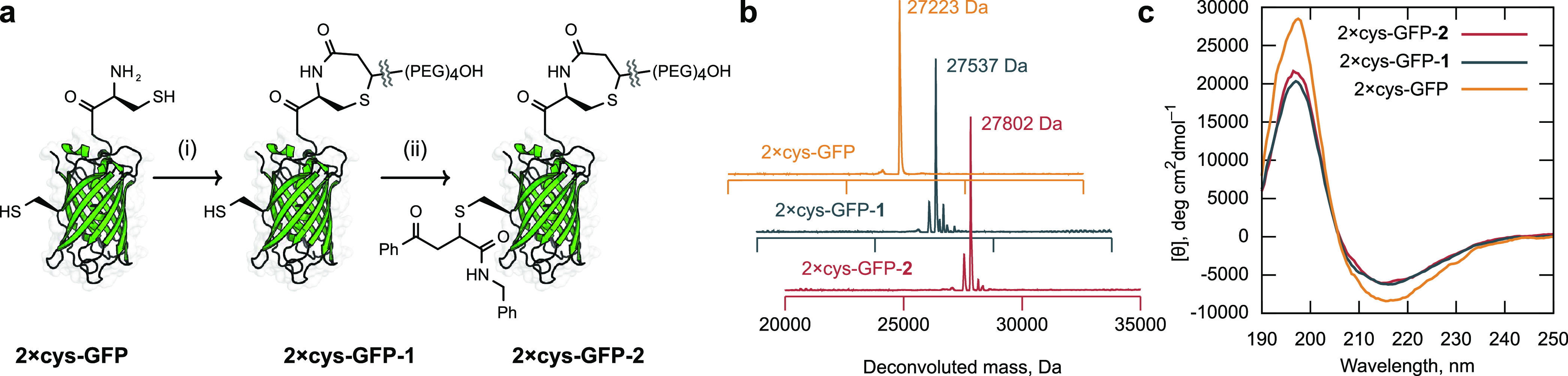
(a) Selective modification of **2**×**cys-GFP** with (i) **CPO-PEG** and (ii) benzoylacrylic reagent. Only the major regioisomer is shown for clarity. (b) Deconvoluted mass spectra of GFP species. (c) Circular dichroism of GFP derivatives.

Finally, CPO-based reagents were applied to the preparation of a protein conjugate endowed with two modifications selectively applied at different cysteine residues (dual protein conjugate). Preparation of dual protein conjugates typically relies on adding both of the two new functionalities to a single residue^49,50^ or relying on disulfide groups for protection;^51^ few methods exploit the inherent reactivity of distinct cysteine residues to achieve site-specific labeling,^9,52^ especially in the absence of a tag sequence.^53−55^ To test CPO-based reagents for this purpose, a double mutant of GFP (**2**×**cys-GFP**) was produced that contained both an internal (S147C) and an N-terminal cysteine residue (Figure 5a). Treatment of **2**×**cys-GFP** with **CPO-PEG** (100 equiv, 2 h) in the presence of DTT (500 equiv) led to a protein conjugate with a mass corresponding to **2**×**cys-GFP-1** (Figure 5b). After excess **CPO-PEG** and DTT were removed, the product was treated with the carbonylacrylic-based reagent^15^**CAA-BN** (20 equiv, 2 h), providing the dual protein conjugate **2**×**cys-GFP-2** (Figure 5a). Attempts to handle **2**×**cys-GFP** in the absence of DTT resulted in extremely rapid dimerization (<5 min) of the protein as assayed by LC–MS, highlighting the importance of DTT-compatible CPO-based reagents for bioconjugation reactions. Following treatment with trypsin, analysis of both **2**×**cys-GFP-1** and **2**×**cys-GFP-2** by LC–MS/MS supported the site-specificity of the two modifications. Analysis by circular dichroism spectroscopy (Figure 5c) confirmed that the initial protein and subsequent conjugates retained the secondary structure with high proportions of antiparallel β-sheets.^56^

### Cyclopropenone Ring-Opening after Double Nucleophilic Attack Makes the Reaction with 1,2-Aminothiols Irreversible

**Figure 6:**
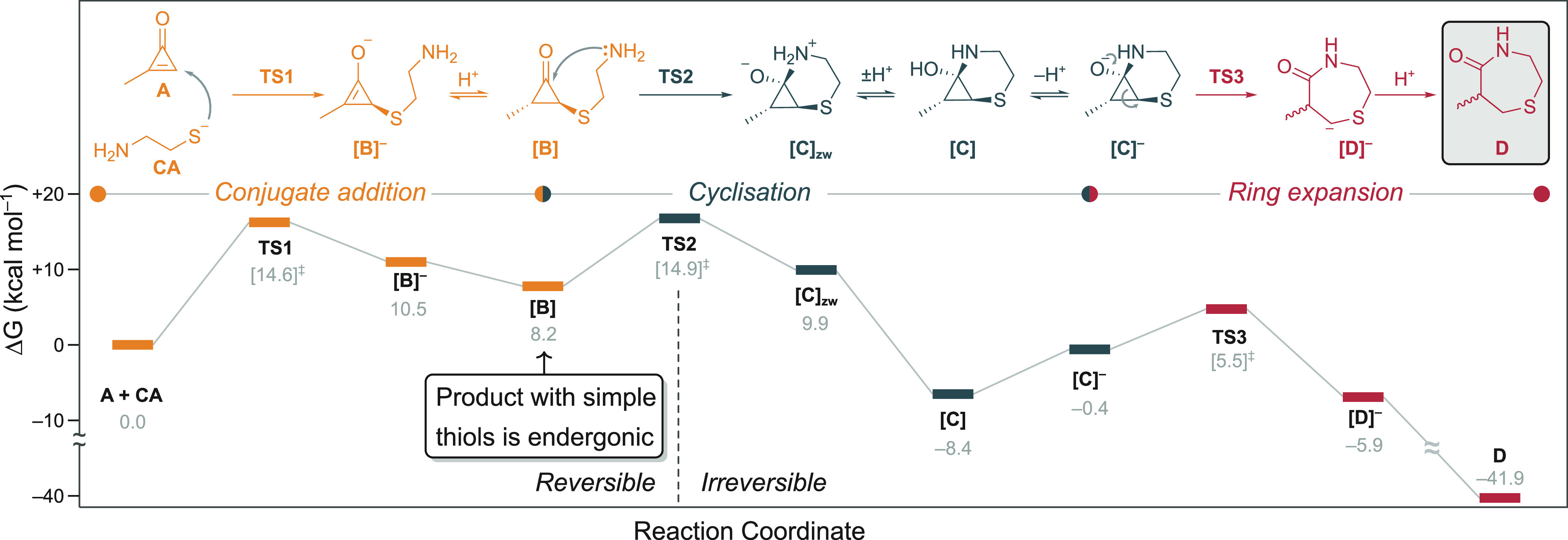
Proposed mechanism and minimum energy pathway for the reaction between 2-methylcyclopropenone (**A**) and cysteamine thiolate (**CA**) calculated at the PCM (H_2_O)/M06-2X/6-31+G(d,p) level of theory. Acid–base equilibria were calculated using trihydrated bicarbonate and carbonate anions as an acid and a base, respectively (see the Supporting Information). Given the intrinsic inaccuracy of calculating such equilibria, relative energies of charged/neutral species should be considered with caution, though qualitative trends hold. The addition of **CA** to **A** to different positions of the double bond can result in two possible pathways, leading to a enantiomeric mixture of two regioisomers (see the Supporting Information); only the minimum energy profile is shown here for simplicity.

To understand the origin of the selectivity for CPO-based reagents for N-terminal cysteine residues over other thiols such as internal cysteine residues and DTT, the whole reaction mechanism was interrogated through quantum mechanical (QM) calculations. 2-Methylcyclopropenone (**A**) and cysteamine (**CA**) provided a suitable abbreviated model for this study (Figure 6), permitting calculation of a comprehensive free energy surface linking each of the four possible regio- and stereoisomeric products to the starting materials (see the Supporting Information). Here, discussion is limited to the minimum energy pathway, although calculations were in good qualitative agreement with the observed ratio of products (Supporting Information).

In the first step of the mechanism, cysteamine thiolate undergoes nucleophilic conjugate addition to the slightly more favored, least-hindered β-carbon of **A** through transition state **TS1** (Δ*G*^‡^_**TS1**_ = 14.6 kcal·mol^–1^). The thio–enolate adduct (**[B]**^–^) is unstable with respect to the starting materials (Δ*G*_[**B**]^−^_ = 10.5 kcal·mol^–1^), as commonly calculated for such intermediates in S-Michael-type reactions.^57^ In contrast to the common trend calculated for noncyclopropenone electrophiles, protonation of the enolate does not lead to significant stabilization of the adduct (Δ*G*_[**B**]^−^_ = 8.2 kcal·mol^–1^). Therefore, the addition of simple thiolates to cyclopropenones is endergonic and reversible. This fact is crucial for the selectivity of CPO-based regents for N-terminal cysteine residues over other thiols such as internal cysteine residue side chains, glutathione, and DTT.

The second part of the reaction mechanism is only accessible to thiols with pendant nucleophiles, thus excluding internal cysteine residues as substrates. The intramolecular addition of the amino group to the cyclopropanone carbonyl proceeds via **TS2** (Δ*G*^‡^_**TS2**_ = 14.9 kcal·mol^–1^) which, upon tautomerization, provides tetrahedral intermediate [**C**] (Δ*G*_[**C**]_ = −8.4 kcal·mol^–1^). Despite the presence of a bicyclic 2-thia-5-azabicyclo[4.1.0]heptane structure in [**C**], the formation of this intermediate is exothermic due to the significant strain released by carbonyl sp^2^→sp^3^ rehybridization, in line with previous observations.^58^ In an alternative mechanistic scenario, we were unable to locate transition states from [**B**]^−^ corresponding to spontaneous ring-opening and formation of ketene–ylide intermediates of the type proposed in the bioorthogonal ligation of cyclopropenones assisted by triarylphosphines,^34^ possibly a result of the substitution for a cationic phosphonium to a neutral thioether substituent. Anionic intermediate [**C**]^−^, formed upon deprotonation of **C**, undergoes a fast ring-expansion with an intrinsic barrier (**TS3**) of 5.9 kcal·mol^–1^ in a process that resembles mechanistic aspects of Favorskii rearrangements.^58,59^ The formation of 1,4-thiazepan-5-one species **D** is strongly exothermic (Δ*G*_[**D**]_ = −41.9 kcal·mol^–1^), in good agreement with the observed stability of protein- and peptide-conjugates containing this functional group. These computational results are in good agreement with the experimentally determined kinetic parameters of the reaction of **1** with l-cysteine ethyl ester.

As a model reaction for lysine side chains reacting with CPO-based reagents, calculations were performed on the addition of methylamine to 2-methylcyclopropenone (**A**). The activation barrier for the initial conjugate addition was calculated to have a free energy of 19.5 kcal·mol^–1^ (Figure S4b), significantly higher than the case for thiolates (Δ*G*^‡^_**TS1**_ = 14.6 kcal·mol^–1^). These calculated results are in accordance with experiment, where the preference for CPO-based reagents to react with thiolates (and 1,2-aminothiols) over primary amines was demonstrated in competition experiments.

### CPO-Based Modification and Dimerization of **nIL2**

**Figure 7:**
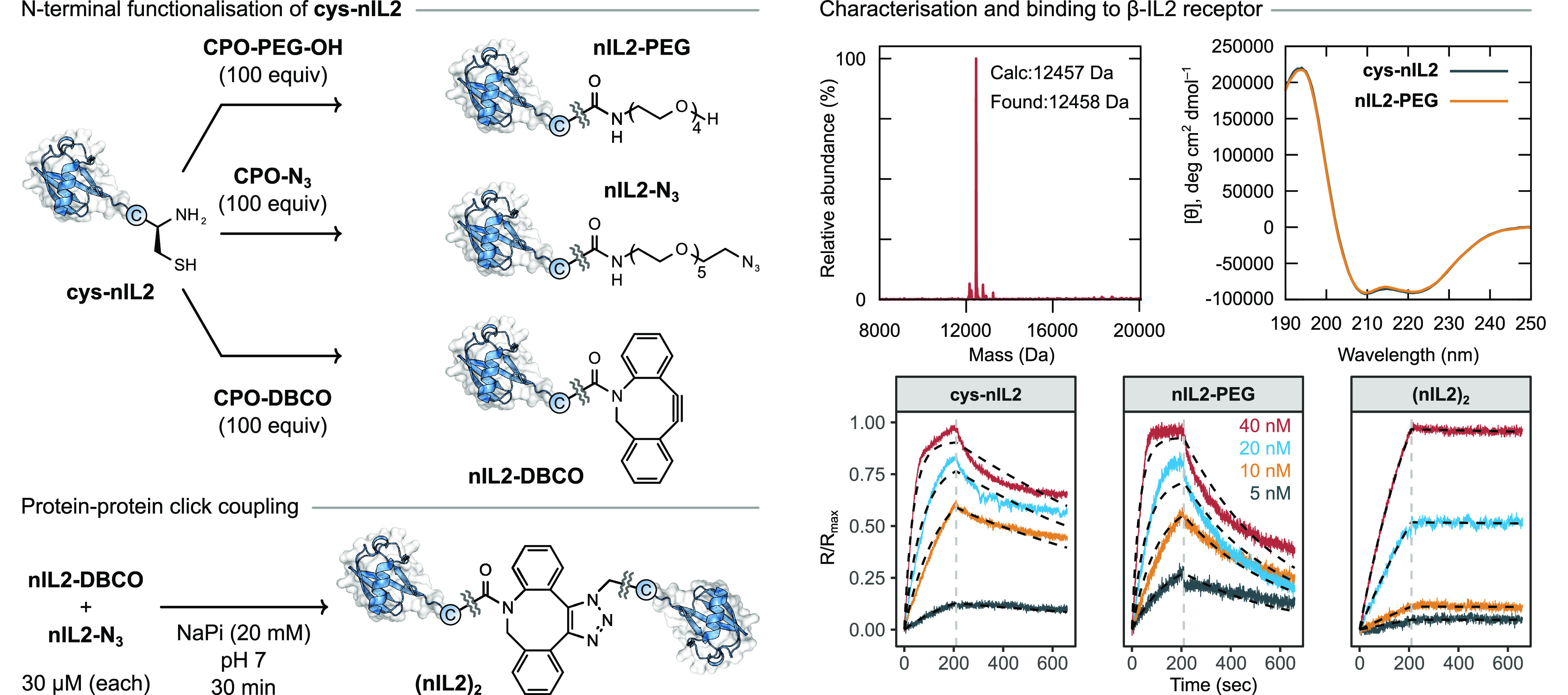
Preparation of a covalent dimer of **cys-nIL2** using CPO-based chemistry. Conditions: (i, ii) **CPO-N**_**3**_ or **CPO-DBCO** (100 equiv, respectively), DTT (100 equiv), NaP_i_ (20 mM), pH 7, 4 h; (iii) NaP_i_ (20 mM), pH 7, 1 h.

Finally, the scope of proteins that could be modified with CPO-based reagents was extended to a protein of relevance to the IL2 receptor. Recent work demonstrated the potential for de novo protein design by preparing a mimic of IL2 (**nIL2**) that binds selectively to the β, γ subunits of the IL2 receptor but not to the α subunit.^60^ The properties of **nIL2** have resulted in its evaluation in a Phase I clinical trial for treating advanced solid tumors with it.^61^ A variant of **nIL2** that has an N-terminal cysteine residue (**cys-nIL2**) was prepared for the purpose of site-specific conjugation with CPO-based reagents. The introduction of an N-terminal cysteine residue was achieved using an enterokinase cleavage site (DDDDK↓C), which also resulted in the removal of the His_6_ purification tag. Because PEGylation of proteins can result in enhanced biological properties and chemical stability,^3^ initial studies focused on the bioconjugation of **cys-nIL2** with **CPO-PEG** (100 equiv); once again the presence of DTT was necessary in order to prevent the formation of a disulfide dimer of **cys-nIL2** but did not interfere with the protein conjugation step. The resulting conjugate, **nIL2-PEG**, formed quantitatively after 3 h and was characterized by mass spectrometry and CD spectroscopy (Figure 7). The latter showed that CPO-based PEGylation did not affect the secondary structure of **cys-nIL2**.

We wondered whether it might be possible to perform a copper-free click reaction between two proteins prepared using a protocol employing CPO-based reagents (Figure 7). De novo designed **cys-nIL2** represents an interesting case study in this regard, as dimerization would be expected to enhance binding to the IL2-β receptor compared to monomeric **nIL2** as a result of avidity effects. In order to prepare a dimer, **cys-nIL2** was treated with either **CPO-N**_**3**_ or **CPO-DBCO**, providing the desired click-labeled protein conjugates **nIL2-N**_**3**_ and **nIL2-DBCO**, respectively. These orthogonally labeled proteins were then combined (∼30 μM), and the reaction was monitored by LC–MS spectrometry. The desired dimer **(nIL2)**_**2**_ formed in 30 min and after purification by size exclusion chromatography was obtained in 52% isolated yield and was characterized by SDS–PAGE and LC–MS spectrometry. This approach utilizing N-terminal cysteine residues is complementary to other methods for protein–protein conjugation, which rely on longer peptide sequence tags at internal or C-terminal positions. In contrast to the use of a bis(maleimide)-PEG_3_ reagent, this approach using bioorthogonal click chemistry enabled by CPO-based protein conjugation permits the use of DTT and the opportunity for N-terminal site-selectivity in the presence of other cysteine residues.

The binding of **cys-nIL2**, **nIL2-PEG**, and **(nIL2)**_**2**_ to the β-IL2 receptor was measured using biolayer interferometry. The unmodified protein **cys-nIL2** exhibited excellent binding (*K*_d_ ≈ 13 nM) in line with the previous measurement of the original protein **nIL2** (*K*_d_ ≈ 19 nM). PEGylated derivative **nIL2-PEG** displayed a slightly diminished binding (*K*_d_ ≈ 34 nM), ostensibly as a result of increased steric bulk engendered by the −(PEG)_4_OH modification. To our delight the dimer **(nIL2)**_**2**_, prepared by CPO-based modification and subsequent click-coupling, provided an order of magnitude increase in the binding (*K*_d_ ≈ 3 nm) compared to the corresponding monomer. This primarily arises due to a large decrease in *k*_off_, a result of the increased avidity offered by two equivalent binding regions on **(nIL2)**_**2**_.

## Conclusion

We have developed an efficient method for selectively modifying N-terminal cysteine residues on peptides and proteins. The method relies on the reaction of monosubstituted cyclopropenones with 1,2-aminothiols, resulting in the formation of a stable 1,4-thiazepan-5-one linkage. Various functional groups (dyes, ligands, biotin, PEG groups, and handles for click chemistry) can be readily added to proteins via the universal precursor **CPO-PFP**, an isolable and bench-stable solid. The reaction of N-terminal cysteine residues and CPO-based reagents proceeds with high efficiency under mild conditions (aqueous buffer, pH 7, 4–25 °C). Remarkably, the reaction is selective for N-terminal cysteine residues in the presence of (i) internal cysteine residues on the same protein, (ii) internal cysteine residues on other proteins, (iii) biological thiols, and (iv) nucleophilic reagents such as DTT. The ability to target N-terminal cysteine residues, readily available on proteins as components of NCL reactions, in the presence of other solvent-exposed and reactive cysteine residues represents a straightforward method for constructing complex and chemically precise bioconjugates, such as **2**×**cys-GFP-2** and **(nIL2)**_**2**_, without the need for extensive protein engineering.

## Supporting Information Available

Detailed methods, characterization data, and additional figures (PDF)

## Accession Codes

CCDC 2010531 contains the supplementary crystallographic data for this paper. These data can be obtained free of charge via www.ccdc.cam.ac.uk/data_request/cif, or by emailing data_request@ccdc.cam.ac.uk, or by contacting The Cambridge Crystallographic Data Centre, 12 Union Road, Cambridge CB2 1EZ, UK; fax: +44 1223 336033.
