# Supplementary material for: Platform for Orthogonal N-Cysteine-Specific Protein Modification Enabled by Cyclopropenone Reagents
Source: J Am Chem Soc. 2022 Jun 5;144(23):10396–406. doi: 10.1021/jacs.2c02185 (PMC9490850; doi:10.1021/jacs.2c02185)
Supplement: Supplementary file 1 — ja2c02185_si_001.pdf [file ja2c02185_si_001.pdf]

Supporting Information for

Platform for Orthogonal N-Cysteine-Specific Protein Modification Enabled by Cyclopropenone Reagents

Alena Istrate,[1] Michael B. Geeson,[1] Claudio D. Navo,[2] Barbara B. Sousa,[3] Marta C. Marques,[3] Ross J. Taylor,[1] Toby Journeaux,[1] Sebastian R. Oehler,[4] Michael R. Mortensen,[4] Michael J. Deery,[5] Andrew D. Bond,[1] Francisco Corzana,[6] Gonzalo Jiménez-Osés,[*,2,7] and Gonçalo J. L. Bernardes[*,1,3]

[1] Yusuf Hamied Department of Chemistry, University of Cambridge, Lensfield Road, CB2 1EW Cambridge, United Kingdom

[2] Center for Cooperative Research in Biosciences (CIC bioGUNE), Basque Research and Technology Alliance (BRTA), Bizkaia Technology Park, Building 800, 48160 Derio, Spain

[3] Instituto de Medicina Molecular João Lobo Antunes, Faculdade de Medicina da Universidade de Lisboa, Av. Prof. Egas Moniz, 1649-028 Lisboa, Portugal

[4] Department of Chemistry and Applied Biosciences ETH Zürich, Vladimir-Prelog-Weg 3, 8093, Zürich, Switzerland

[5] Cambridge Centre for Proteomics, Gleeson Building, University of Cambridge, Tennis Court Road, Cambridge, CB2 1QR

[6] Departamento de Química, Universidad de La Rioja, Centro de Investigación en Síntesis Química, 26006 Logroño, Spain

[7] Ikerbasque, Basque Foundation for Science, 48013 Bilbao, Spain

Correspondence: gb453@cam.ac.uk (G.J.L.B.) and/or gjoses@cicbiogune.es (G.J.-O.)

Table of Contents

1 List of abbreviations 5
2 General methods 6
2.1 Chemicals and characterization of the compounds 6
2.2 LC–MS method for analysis 6
3 Synthetic procedures 7
3.1 2-Phenethylcycloprop-2-en-1-one (1) 7
3.2 Compounds 2a–d 8
3.3 Compounds 3a–d 9
3.4 Compound 5 10
3.5 CPO-PFP (6) 11
3.6 CPO-BN 12
3.7 CPO-PEG 13
3.8 CPO-PEG-Alkyne 14
3.9 CPO-EDANS 15
3.10 CPO-DBCO 15
3.11 CPO-N_3 16
3.12 CAA-BN 17
4 X-ray structure analysis of 2a 18
4.1 Methods 18
4.2 X-Ray Crystallography 18
5 Quantum Mechanical Calculations 20
5.1 Cartesian coordinates of the lowest-energy calculated structures. 28
6 Kinetic study for the reaction of cyclopropenone probe 1 with cysteine 36
6.1 Stability studies of cyclopropenone probe 1 in buffer 37
7 Reaction and stability of cyclopropenones with nucleophiles 38
7.1 Selectivity studies and competition experiments. General methods 38
7.1.1 Control experiment 40
7.1.2 Selectivity in the presence of lysine 41
7.1.3 Selectivity in the presence of tyrosine 42
7.1.4 Selectivity in the presence of threonine 43
7.1.5 Selectivity in the presence of serine 44
7.1.6 Selectivity in the presence of glutathione 45
7.1.7 Selectivity in the presence of N-(tert-butoxycarbonyl)-protected cysteine 46
8 Peptide modification 47

8.1 General procedures and characterization methods....47
8.2 Reactions and characterization of peptide conjugates 48
9 Protein modification....59
9.1 General procedures 59
9.1.1 Analysis of protein conjugation by LC–MS. 59
9.1.2 Enzymatic digestion and LC–MS/MS analysis....60
9.1.3 Circular dichroism spectroscopy....61
9.1.4 Bio-layer interferometry (BLI) 61
9.2 Proteins used in this study....62
9.2.1 Cys-GFP....62
9.2.2 2×Cys-GFP 63
9.2.3 C2Am....66
9.2.4 Annexin V....66
9.2.5 Nanobody DesAB-HET[12]....67
9.2.6 cys-nIL2 68
9.3 Reactions and characterization of Cys-GFP-conjugates....69
9.3.1 Reaction of Cys-GFP with CPO-BN to give GFP-CPO-BN 69
9.3.2 Stability of GFP-CPO-BN in glutathione (GSH).....71
9.3.3 Enzymatic digestion of GFP-CPO-BN and LC–MS/MS analysis confirming the modified site....73
9.3.4 Reaction of Cys-GFP with CPO-PEG....74
9.3.5 Reaction of Cys-GFP with CPO-EDANS....75
9.3.6 Reaction of Cys-GFP with *N*-methylmaleimide 76
9.3.7 Reaction of Cys-GFP with CPO-biotin....77
9.3.8 Reaction of Cys-GFP with Maleimide-Biotin....78
9.3.9 Selective modification of Cys-GFP in a mixture of proteins....79
9.4 Reactions of 2×Cys-GFP 87
9.5 Reactions of cys-nIL2....87
9.5.1 Preparation of nIL2-PEG 87
9.5.2 Preparation of $(nIL2)_2$....88
9.6 Enzymatic digestion 2×Cys-GFP-1, 2×Cys-GFP-2, and nIL2-PEG followed by LC−MS/MS analysis....90
9.6.1 2×Cys-GFP-1 90
9.6.2 2×Cys-GFP-2 90
9.6.3 nIL2-PEG....91
9.7 Control Reactions....92

9.7.1 Wild-type GFP (WT-GFP) with CPO-PEG (+/- DTT) ... 92
9.7.2 Lysozyme with CPO-PEG (+/- DTT) ... 94
9.7.3 Attempt to prepare a dimer of cys-nIL2 using a bismaleimide reagent (+/- DTT) ... 95
9.7.4 Reactivity of 2×Cys-GFP with NMM ... 96
9.8 Control to check binding of nIL2-PEG or $(nIL2)_2$ to IL2-R_{α} ... 97
10 Molecular dynamics (MD) simulations ... 98
11 NMR spectra ... 100
12 References ... 115

1 List of abbreviations

**BLI**	bio-layer interferometry
**CC**	column chromatography
**CD**	circular dichroism
**CAABN**	(2*E*)-N-benzyl-4-oxo-4-phenylbut-2-enamide
**CPO-BN**	4-(3’-oxocycloprop-1’-en-1’-yl)-N-phenylbutanamide
**CPO-biotin**	5-[(3aS,4S,6aR)-2-oxo-hexahydro-1H-thieno[3,4-d]imidazol-4-yl]-N-[2-(2-{2-[4-(3-oxocycloprop-1-en-1-yl)butanamido]ethoxy}ethoxy)ethyl]pentan-amide
**CPO-DBCO**	N-(3-{2-azatricyclo[10.4.0.04,9]hexadeca-1(16),4,6,8,12,14-hexaen-10-yn-2-yl}-3-oxopropyl)-4-(3-oxocycloprop-1-en-1-yl)butanamide
**CPO-EDANS**	Sodium 5-((2’-(4’’-(3’’’-oxocycloprop-1’’’-en-1’’’-yl)butanamido)ethyl)am-ino) naphthalene-1-sulfonate
**CPO-N_3**	N-(17-azido-3,6,9,12,15-pentaoxaheptadecan-1-yl)-4-(3-oxocycloprop-1-en-1-yl)butanamide
**CPO-PEG**	N-(2-(2-(2-(2-hydroxyethoxy)ethoxy)ethoxy)ethyl)-4-(3’-oxocycloprop-1’-en-1’-yl)butanamide
**CPO-PEG-Alkyne**	4-(3’-oxocycloprop-1’-en-1’-yl)-N-(3’’,6’’,9’’,12’’-tetraoxapentadec-14’’-yn-1’’-yl)butanamide
**CPO-PFP**	perfluorophenyl 4-(3’-oxocycloprop-1’-en-1’-yl)butanoate
**DIPEA**	N,N-diisopropylethylamine
**DMF**	dimethylformamide
**DTT**	dithiothreitol
**GSH**	glutathione
**HPLC**	high-performance liquid chromatography
**LC–MS**	liquid chromatography–mass spectrometry
**MD**	molecular dynamics
**MS**	mass spectrometry
**PE**	petroleum ether
**r.t.**	room temperature
**THF**	tetrahydrofuran
**TLC**	thin layer chromatography
**Tris HCl**	tris(hydroxymethyl)aminomethane hydrochloride

2 General methods

2.1 Chemicals and characterization of the compounds

Chemicals were purchased and used without further purification. Anhydrous solvents for reactions were obtained by distillation over drying agents or purchased from Sigma Aldrich (DMF). All non-aqueous reactions were performed under an atmosphere of argon in oven-dried glassware. Argon gas was pre-dried *via* passage through calcium chloride. Column chromatography (CC) was performed on silica gel (pore size 60 Å, *230-400 mesh* particle size, Merck). All solvents for CC were of technical grade and distilled prior to use. Thin-layer chromatography (TLC) was performed on silica gel plates (TLC Silica gel 60 F_{254}, Merck). Visualization was achieved either under UV light (254 nm) and/or by staining in dip solutions [Cer(IV)-sulfate (10.5 g), phosphormolybdenic acid (21 g), concentrated H_2SO_4 (60 ml), H_2O (900 ml) or $KMnO_4$ (6 g), K_2CO_3 (40 g), 15% NaOH (3 ml) in H_2O (800 ml)] followed by heating with a heat gun. NMR spectra were recorded on a Bruker Avance III HD 400 MHz, Bruker Avance III HD 500 MHz spectrometers at 400 or 500 MHz (^{1}H), 100 or 125 MHz (^{13}C), at 376.5 MHz (^{19}F) of in either $CDCl_3$ or DMF-d_7 at room temperature. Chemical shifts (δ) are reported relative to the undeuterated residual solvent peak [$CHCl_3$: 7.26 ppm (^{1}H) and 77.16 ppm (^{13}C); DMF-d_7: 2.92 ppm (^{1}H) and 163.15 ppm (^{13}C)]. Signal assignments are based on DEPT or APT experiments, and on ^{1}H,^{1}H and ^{1}H,^{13}C correlation experiments. Multiplicities are abbreviated as *b* (broad), *s* (singlet), *d* (doublet), *t* (triplet), *q* (quartet), *m* (multiplet) or combinations thereof. High-resolution mass spectra were performed by the Mass Spectrometry Service, Department of Chemistry, University of Cambridge using a Waters LCT Premier or a Waters Xevo G2-S spectrometer and ionized by ESI or ASAP.

2.2 LC–MS method for analysis

LC–MS was performed on a Waters SQ Detector 2 mass spectrometer coupled to an Acquity UPLC system using an Acquity UPLC BEH C18 column (130Å, 1.7 µm, 2.1 mm X 50 mm). Solvents A, water with 0.1% formic acid and B, 71% acetonitrile, 29% water and 0.075% formic acid were used as the mobile phase at a flow rate of 0.2 mL min^{-1}. The gradient was programmed as follows: 100% A for 2 min, then 100% A to 100% B in 9 min, then 100% B for 5 min, and after that 100% A for 4 min. The electrospray source was operated with a capillary voltage of 3.0 kV and a cone voltage of 30 V. Nitrogen was used as the desolvation gas at a total flow of 800 L h^{-1}. *m/z* values of positively and negatively charged ions were measured in the mass analyser, which was scanned between *m/z* 100-2000 for the generation of mass spectra. The major peak(s) were selected for integration and analysed using MassLynx software (v. 4.1 from Waters).

3 Synthetic procedures

3.1 2-Phenethylcycloprop-2-en-1-one (1)

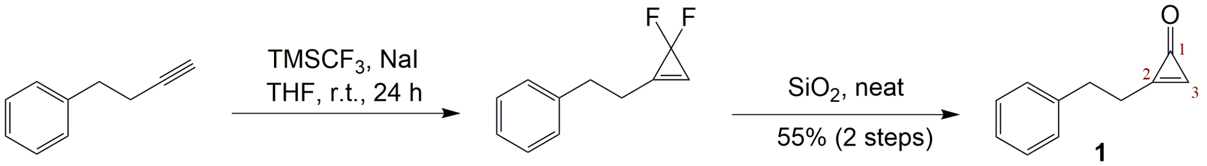

1

In a glove box, sodium iodide (330 mg, 2.2 mmol, 2.2 equiv.) was added to an oven-dried flask equipped with a magnetic stir bar, after which the flask was closed with septa, transferred to a fume hood and filled with N_2. Next, dry THF (3 mL), starting alkyne (140 uL, 1 mmol, 1 equiv.), and $TMSCF_3$ (0.3 mL, 2 mmol, 2 equiv.) were added via syringe, and the reaction mixture was allowed to stir at r.t. for 24 h. Subsequently, the reaction mixture was poured into H_2O (10 ml) and extracted with Et_2O (3 x 10 ml). The combined organic phases were dried over $MgSO_4$, filtrated, evaporated, and the crude difluorocyclopropene intermediate was adsorbed on silica gel (1 g) and allowed to stand at r.t. overnight to generate the corresponding cyclopropenone product. Purification of the adsorbed material by CC (PE/EtOAc 2:1 → EtOAc) afforded the product **1** (87 mg, 55%) as a dark-brown oil.

TLC (PE/EtOAc 2:3) R_f = 0.17.

^{1}H NMR ($CDCl_3$, 400 MHz): δ 8.34 (*s*, H-C(3)), 7.37–7.18 (*m*, 5H, H-arom), 3.09–2.98 (*m*, 4H, CH_2).

^{13}C NMR ($CDCl_3$, 100 MHz): δ 169.47 (C(2)), 157.47 (C(1)), 149.24 (C(3)), 139.34 (C-arom), 128.94 (CH-arom), 128.41 (CH-arom), 126.96 (CH-arom), 31.89 (CH_2), 29.24 (CH_2).

ESI$^+$-HRMS: calculated for $C_{11}H_{11}O$ ($[M+H]^+$) 159.0810, found 159.0808.

3.2 Compounds 2a–d

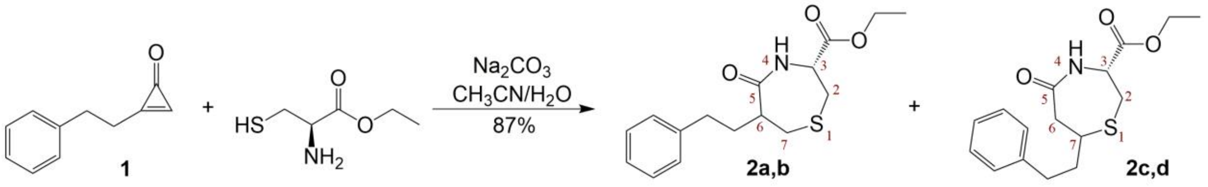

L-Cysteine ethyl ester hydrochloride (37.32 mg, 0.2 mmol, 1 equiv.) was dissolved in H_2O/CH_3CN (20 mL, 1/9) and cooled to 0˚C. Next, Na_2CO_3 (21 mg, 0.4 mmol, 2 equiv.) and 2-phenethylcycloprop-2-en-1-one (300 µL of 0.67 M stock solution in CH_2Cl_2, 0.2 mmol of **1**, 1 equiv.), and the resulting solution was stirred for 30 min at 0˚C. The reaction mixture was washed with 0.15 M HCl solution, and the aqueous phase was extracted with CH_2Cl_2 (3 × 10 mL). The combined organic phases were dried over $MgSO_4$, evaporated and the crude product was purified by CC (PE/ethyl acetate 8:2 → 3:2) to give two fractions: **2a** (9.9 mg, 16%; 53.2 mg; 3R,6R-isomer) and **2b–d** (43.8 mg, 71%; mixture of three isomers) as white solids.

2a (3R,6R-isomer):

TLC (PE/EtOAc 2:3) R_f = 0.64.

^{1}H NMR ($CDCl_3$, 400 MHz): δ 7.32–7.26 (m, 2H, H-arom), 7.23–7.17 (m, 3H, H-arom), 6.58 (d, *J* = 6.1 Hz, 1H, NH), 4.49 (ddd, *J* = 9.4, 6.1, 1.5 Hz, 1H, H(3)), 4.28 (q, *J* = 7.1 Hz, 2H, C*H*$_2$-CH$_3$), 2.99 (d, *J* = 14.1, 1H, H(2a)), 2.86–2.70 (m, 4H, H(2b), H(6), H(7b), CH$_2$CH^a**H^b**Ph), 2.66–2.57 (ddd, *J* = 13.7, 8.8, 6.4 Hz, 1H, CH$_2$C**H^a**H^{b}Ph), 2.49 (d, *J* = 13.1, 1H, H(7a)), 2.34-2.23 (dddd, *J* = 13.9, 8.8, 7.6, 6.2 Hz, 1H, CH^a**H^b**CH$_2$Ph), 1.73–1.62 (m, 1H, C**H^a**H^{b}CH$_2$Ph), 1.32 (t, *J* = 7.1 Hz, 3H, CH$_2$-C**H$_3$**).

^{13}C NMR ($CDCl_3$, 100 MHz): δ 175.73 (NH**C**O), 169.72 (**C**OEt), 141.64 (C-arom), 128.65 (CH-arom), 128.58 (CH-arom), 126.13 (CH-arom), 62.89 (**C**H$_2$CH$_3$), 58.42 (C(3)), 47.78 (C(6)), 34.70 (C(2)), 33.77 (**C**H$_2$CH$_2$Ph), 33.63 (CH$_2$**C**H$_2$Ph), 30.45 (C(7)), 14.22 (CH$_2$**C**H$_3$).

ESI$^+$-HRMS: calculated for $C_{16}H_{22}NO_3S$ ($[M+H]^+$) 308.1320, found 308.1318.

2b–d:

TLC (PE/EtOAc 2:3) R_f = 0.40.

^{1}H NMR ($CDCl_3$, 400 MHz): δ 7.38–7.19 (m, 5H, H-arom), 6.62–6.46 (m, 1H), 4.53–4.44 (m, 1H), 4.36–4.25 (m, 2H), 3.27–2.58 (m, 7H), 2.35–1.89 (m, 2H), 1.42–1.29 (m, 3H).

^{1}H NMR and ^{13}C NMR not assigned due to mixture of isomers.

ESI$^+$-HRMS: calculated for $C_{16}H_{22}NO_3S$ ($[M+H]^+$) 308.1320, found 308.1318.

3.3 Compounds 3a–d

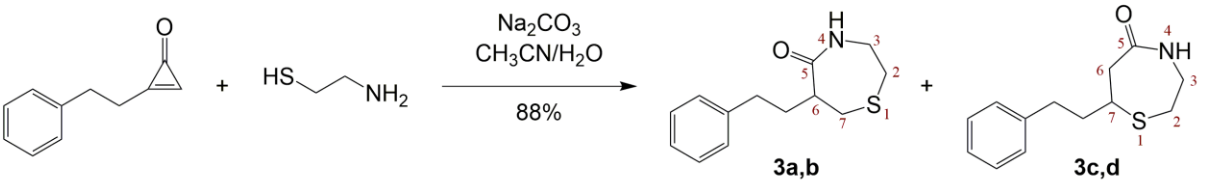

3a,b **3c,d**

Cysteamine (22 mg, 0.29 mmol, 1.5 equiv.) was dissolved in H_2O/CH_3CN (90 mL, 1/1) and cooled to 0˚C. Next, Na_2CO_3 (20 mg, 0.19 mmol, 1 equiv.) and 2-phenethylcycloprop-2-en-1-one (2.92 mL of 0.065 M stock solution in CH_3CN, 0.19 mmol of **1**, 1 equiv.), and the resulting solution was stirred for 1 h at 0˚C. The reaction mixture was then concentrated to one-half volume and extracted with CH_2Cl_2 (3 × 20 mL). The combined organic phases were dried over $MgSO_4$, evaporated and the crude product was purified by CC (petrol/ethyl acetate 2:3 → 2:6) to give products **3** (12.3 mg, 28%) and **3** (26.6 mg, 60%) as white solids.

3a,b:

TLC (PE/EtOAc 2:3) R_f = 0.31.

^{1}H NMR ($CDCl_3$, 400 MHz): δ 7.31–7.09 (*m*, 5H, H-arom), 6.14 (*s*, 1H, NH), 3.68–3.43 (*m*, 2H, H-C(3)), 2.87 (*dddd*, *J* = 9.7, 7.9, 6.1, 1.7 Hz, 1H, H-C(6)), 2.76–2.51 (*m*, 5H, $CH_2C\mathbf{H_2}Ph$, 2xH-C(2), H-C(7)), 2.43 (*d*, *J* = 14.7 Hz, 1H, H-C(7)), 2.28–2.13 (*m*, 1H, $C\mathbf{H^a}H^bCH_2Ph$), 1.74–1.58 (*m*, 1H, $CH^a\mathbf{H^b}CH_2Ph$).

^{13}C NMR ($CDCl_3$, 100 MHz): δ 178.43 (C(5)), 141.72 (*C*-arom), 128.67, 128.56, 126.11 (*C*H-arom), 47.42 (C(6)), 45.04 (C(3)), 33.69 ($CH_2\mathbf{C}H_2Ph$), 33.54 ($\mathbf{C}H_2CH_2Ph$), 31.17 (C(2)), 30.36 (C(7)).

ESI$^+$-HRMS: calculated for $C_{13}H_{18}NOS$ ($[M+H]^+$) 236.1109, found 236.1104.

3c,d:

TLC (PE/EtOAc 2:3) R_f = 0.16.

^{1}H NMR ($CDCl_3$, 400 MHz): δ 7.31–7.14 (*m*, 5H, H-arom), 6.62 (*s*, 1H, NH), 3.58 (*td*, *J* = 6.0, 4.3 Hz, 2H, H-C(3)), 3.01–2.90 (*m*, 2H, 2xH-C(6)), 2.86–2.73 (*m*, 4H, C(7), $CH_2C\mathbf{H_2}Ph$, H-C(2)), 2.71–2.62 (*m*, 1H, H-C(2)), 2.03–1.84 (*m*, 2H, $C\mathbf{H^aH^b}CH_2Ph$).

^{13}C NMR ($CDCl_3$, 100 MHz): δ 176.24 (C(5)), 141.15 (*C*-arom), 128.65, 128.59, 126.19 (*C*H-arom), 46.56, C(6)), 45.90 (C(3)), 37.01 (C(7)), 36.58 ($\mathbf{C}H_2CH_2Ph$), 33.37 ($CH_2\mathbf{C}H_2Ph$), 29.98 (C(2)).

ESI$^+$-HRMS: calculated for $C_{13}H_{18}NOS$ ($[M+H]^+$) 236.1109, found 236.1102.

3.4 Compound 5

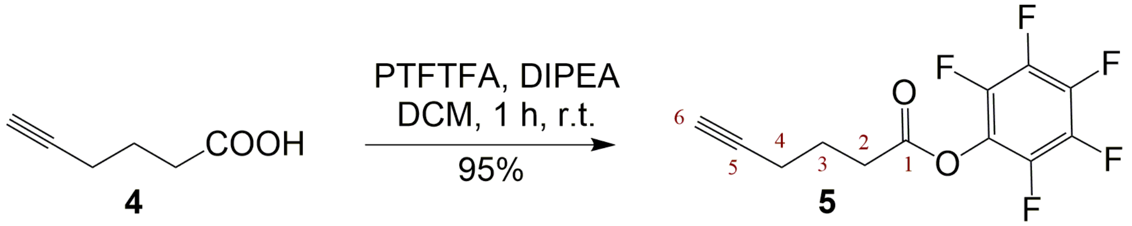

A solution of 5-hexynoic acid **4** (4.00 g, 35.7 mmol, 1 equiv) in dry CH_2Cl_2 (130 mL) was cooled to 0°C. Next, DIPEA (14 mL, 80.4 mmol, 2.25 equiv.) was added, followed by dropwise addition of pentafluorophenyl trifluoroacetate (12 mL, 69.8 mmol, 1.95 equiv). The resulting solution was allowed to warm up to r.t. then stirred for 1.5 h. The reaction mixture was concentrated on a rotovap and the crude product was purified by CC (PE/Et_2O 98:2) to give product **5** (9.44 g, 95%) as a colourless liquid.

TLC (PE/Et_2O 4:1) R_f= 0.80.

^{1}H NMR ($CDCl_3$, 400 MHz): δ 2.84 (t, J = 7.4 Hz, 2H, H-C(2)), 2.36 (td, J = 6.9, 2.7 Hz, 2H, H-C(4)), 2.05–1.94 (m, 3H, H-C(6), H-C(3)).

^{13}C NMR ($CDCl_3$, 100 MHz): δ 169.15 (C(1)), 142.67–142.12, 141.01–140.48, 140.13–139.69, 139.45–138.92, 138.53–137.94, 137.11–136.27, 125.44–124.70 (m, (C-arom)), 82.57 (C(5)), 69.90 (C(6)), 32.02 (C(2)), 23.46 (C(3)), 17.75 (C(4)).

^{19}F NMR ($CDCl_3$, 376 MHz): δ −153.72 to −153.88 (m), −159.01 (t, J = 21.6 Hz), −163.24 to −163.44 (m).

ESI$^+$-HRMS: calculated for $C_{12}H_8F_5O_2$ ($[M+H]^+$) 279.0444, found 279.0435.

3.5 CPO-PFP (6)

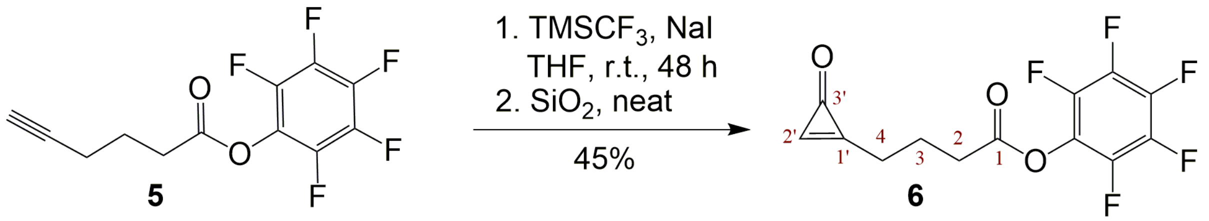

In a glovebox, to an oven-dried pressure tube (10 mL) equipped with a magnetic stir bar was added anhydrous NaI (330 mg, 2.2 mmol). The flask was transferred to a fume hood and placed under an inert atmosphere. Next, anhydrous THF (3.0 mL), perfluorophenyl hex-5-ynoate **5** (278 mg, 1.0 mmol), and trifluoromethyltrimethylsilane (300 µL, 2.0 mmol) were added, and the reaction was stirred vigorously at room temperature for 24 h. After that, a second portion of trifluoromethyltrimethylsilane (150 µL, 1.0 mmol) was added, and the reaction mixture was stirred for another 24 h. The reaction mixture was then diluted with H_2O (20 mL) and extracted with Et_2O (3 × 20 mL). The combined organic phases were dried over $MgSO_4$, filtered, concentrated, adsorbed on silica, and left overnight at room temperature to generate the corresponding cyclopropenone. The product was then purified by CC (PE/EtOAc 95:5 to 1:1 to 0:1) to afford the perfluorophenyl 4-(3'-oxocycloprop-1'-en-1'-yl)butanoate **6** (131 mg, 43%) as a white solid.

TLC of the difluorocyclopropene intermediate (PE/Et_2O 4:1) R_f= 0.45.

TLC of **6** (EtOAc) R_f= 0.50.

^{1}H NMR ($CDCl_3$, 500 MHz): δ 8.53 (s, 1H, H-C(2')), 2.89–2.82 (m, 4H, H-C(2), H-C(4)), 2.22 (p, *J* = 7.3 Hz, 2H, H-C(3)).

^{13}C NMR ($CDCl_3$, 125 MHz): δ 168.85 (C(1)), 168.59 (C(1')), 157.21 (C(3')), 149.25 (C(2')), 142.30–141.82, 140.84–140.41, 140.16–139.86, 139.09–138.37, 137.07–136.71, 125.01–124.60 (m, (C-arom)), 32.25 (C(2)), 26.80 (C(4)), 20.97 (C(3)).

^{19}F NMR ($CDCl_3$, 376 MHz): δ −152.03 to −153.11 (m), −157.47 (t, *J* = 21.7 Hz), −160.76 to −162.53 (m).

ESI$^+$-HRMS: calculated for $C_{13}H_7F_5O_3Na$ ($[M+Na]^+$) 329.0208, found 329.0200.

3.6 CPO-BN

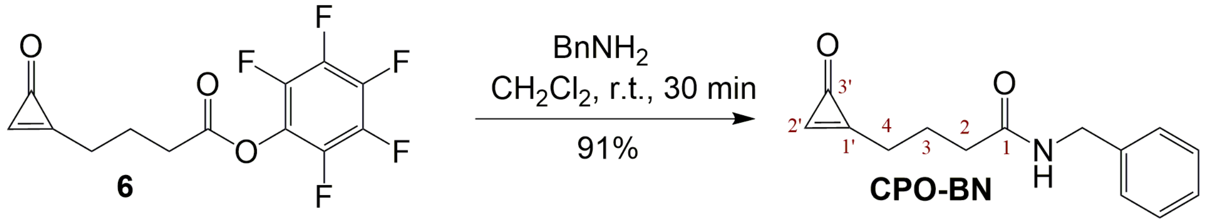

Perfluorophenyl 4-(3'-oxocycloprop-1'-en-1'-yl)butanoate **6** (800 mg, 2.6 mmol) was dissolved in CH_2Cl_2 (32 mL), and portions of benzylamine (600 µL in total, 5.5 mmol, 2.1 equiv.) were added every 5–10 min until there was no starting material left according to TLC. After that, the reaction mixture was concentrated* and purified by CC (EtOAc 100%; CH_2Cl_2/MeOH 95:5)** to give **CPO-BN** (542.5 mg, 91%) as a brown oil.

* Avoid complete evaporation of solvent as this can result in product decomposition if large excess of benzylamine is used.

** Column first washed with EtOAc to remove the by-product (pentafluorophenol) and the product then washed out with CH_2Cl_2/MeOH 95:5.

TLC (EtOAc) R_f= 0.17.

^{1}H NMR ($CDCl_3$, 400 MHz): δ 8.40 (s, 1H, H-C(2')), 7.36–7.23 (m, 5H, H-arom), 6.42 (br, 1H, NH), 4.43 (d, J = 5.8 Hz, 2H, CH_2Ph), 2.74 (t, J = 7.0 Hz, 2H, H-C(4)), 2.40 (t, J = 7.0 Hz, 2H, H-C(2)), 2.08 (p, J = 7.0 Hz, 2H, H-C(3)).

^{13}C NMR ($CDCl_3$, 125 MHz): δ 171.48 (C(1)), 169.60 (C(1')), 158.22 (C(3')), 148.14 (C(2')), 138.34 (C-arom), 128.86, 128.01, 127.68 (CH-arom), 43.82 (CH_2Ph), 34.90 (C(2)), 26.82 (C(4)), 21.74 (C(3)).

ESI$^+$-HRMS: calculated for $C_{14}H_{16}NO_2$ ($[M+H]^+$) 230.1176, found 230.1169.

3.7 CPO-PEG

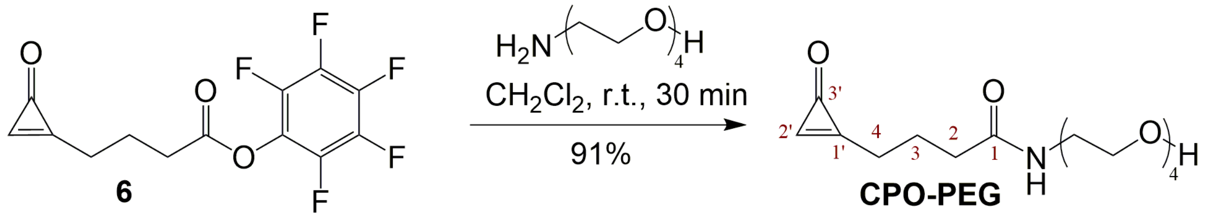

6 **CPO-PEG**

Perfluorophenyl 4-(3'-oxocycloprop-1'-en-1'-yl)butanoate **6** (10 mg, 0.03 mmol) was dissolved in CH_2Cl_2 (1 mL), and portions of HO-PEG4-NH_2 (10 mg in total, 0.05 mmol, 1.6 equiv.) were added every 5–10 min until there was no starting material left according to TLC. After that, the reaction mixture was concentrated and purified by CC (CH_2Cl_2/MeOH 93:7 to 4:1) to give **CPO-PEG** (9.2 mg, 89%) as a colourless oil.

TLC (CH_2Cl_2/MeOH 93:7) R_f= 0.27.

^{1}H NMR ($CDCl_3$, 400 MHz): δ 8.46 (s, 1H, H-C(2')), 7.40 (br, 1H, NH), 3.75–3.70 (m, 4H, 2 x CH_2-PEG), 3.68–3.59 (m, 8H, 4 x CH_2-PEG), 3.54–3.51 (m, 2H, CH_2-PEG), 3.44 (q, J = 5.0 Hz, 2H, C<u>H</u>$_2$NH(PEG)), 2.75 (t, J = 7.1 Hz, 2H, H-C(4)), 2.34 (t, J = 7.2 Hz, 2H, H-C(2)), 2.06 (p, J = 7.1 Hz, 2H, H-C(3)).

^{13}C NMR ($CDCl_3$, 125 MHz): δ 171.92 (C(1)), 169.77 (C(1')), 158.04 (C(3')), 148.57 (C(2')), 72.68, 70.82, 70.57, 70.32, 70.12, 70.07, 61.65 (7 x CH_2-PEG), 39.30 (**C**H_2NH(PEG)), 34.81 (C(2)), 27.11 (C(4)), 21.74 (C(3)).

ESI$^+$-HRMS: calculated for $C_{15}H_{26}O_6N_1$ ([M+H]$^+$) 316.1755, found 316.1744.

3.8 CPO-PEG-Alkyne

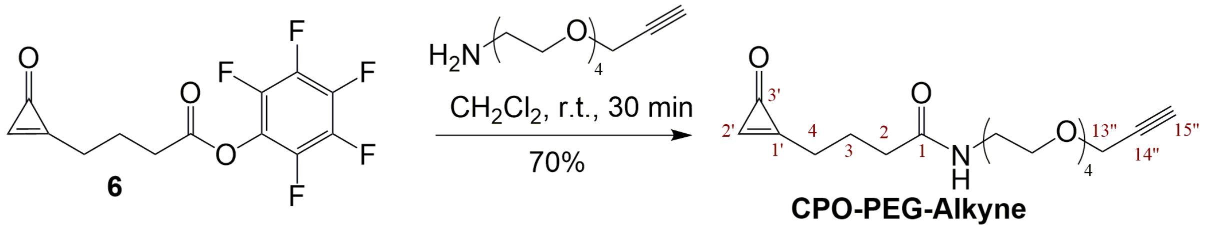

Perfluorophenyl 4-(3'-oxocycloprop-1'-en-1'-yl)butanoate **6** (10.0 mg, 32.7 µmol) was dissolved in CH_2Cl_2 (1 mL), and portions of alkyne-PEG$_4$-NH$_2$ (15.1 mg in total, 65.3 µmol, 2 equiv.) were added every 5–10 min until there was no starting material left according to TLC. After that, the reaction mixture was concentrated and purified by CC (CH_2Cl_2/MeOH 93:7 to 4:1) to give **CPO-PEG-Alkyne** (8.0 mg, 70%) as a colourless oil.

TLC (CH_2Cl_2/MeOH 9:1) R_f= 0.20.

^{1}H NMR ($CDCl_3$, 400 MHz): δ 8.47 (s, 1H, H-C(2')), 6.68 (br, 1H, NH), 4.19 (d, J = 2.4 Hz, 2H, H-C(13'')), 3.73–3.59 (m, 12H, 6 x CH_2-PEG), 3.57–3.52 (m, 2H, CH_2-PEG), 3.44 (q, J = 5.2 Hz, 2H, C$\underline{H}_2$NH(PEG)), 2.76 (t, J = 7.1 Hz, 2H, H-C(4)), 2.44 (t, J = 2.3 Hz, 1H, H-C(15'')), 2.35 (t, J = 7.1 Hz, 2H, H-C(2)), 2.06 (p, J = 7.1 Hz, 2H, H-C(3)).

^{13}C NMR ($CDCl_3$, 100 MHz): δ 171.82 (C(1)), 169.66 (C(1')), 158.03 (C(3')), 148.48 (C(2')), 79.61 (C(14'')), 74.84 (C(15'')), 70.65, 70.61, 70.44, 70.29, 69.92, 69.22 (6 x CH_2-PEG), 58.53 (C(13'')), 39.36 (CH_2NH(PEG)), 34.89 (C(2)), 26.96 (C(4)), 21.71 (C(3)).

ESI$^+$-HRMS: calculated for $C_{18}H_{28}O_6N_1$ ([M+H]$^+$) 354.1911, found 354.1902.

3.9 CPO-EDANS

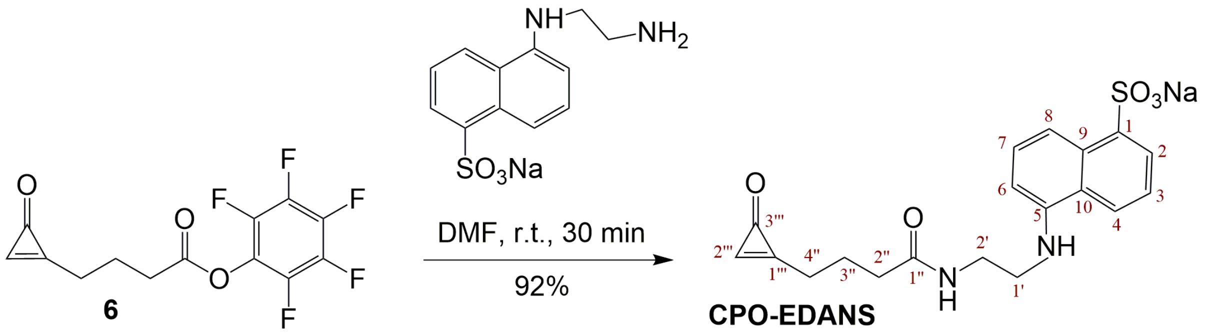

Perfluorophenyl 4-(3'-oxocycloprop-1'-en-1'-yl)butanoate **6** (10.0 mg, 32.7 µmol) was dissolved in DMF (400 µL), and N-(aminoethyl)-5-naphthylamine-1-sulfonic acid in DMF (18.8 mg in 600 µL DMF, 65.2 µmol, 2 equiv.) was added and the reaction mixture was stirred for 30 min, concentrated, and purified by CC (EtOAc/MeOH 1:0 to 1:1) to give **CPO-EDANS** (12.3 mg, 92%).

TLC (EtOAc/MeOH 7:3) R_f= 0.23.

^{1}H NMR (DMF-d_7, 500 MHz): δ 8.97 (s, 1H, H-C(2''')), 8.43 (d, J = 8.7 Hz, 1H, H-arom), 8.27 (s, 1H, NH), 8.15 (d, J = 8.5 Hz, 1H, H-arom), 8.12 (d, J = 7.0 Hz, 1H, H-arom), 7.35–7.24 (m, 2H, H-arom), 6.59 (d, J = 7.5 Hz, 1H, H-arom), 6.13 (s, 1H, NH), 3.59–3.52 (m, 2H, H-C(2')), 3.41–3.36 (m, 2H, H-C(1')), 2.78–2.71 (m, 2H, H-C(4'')), 2.37 (t, J = 7.3 Hz, 2H, H-C(2'')), 2.04–1.95 (m, 2H, H-C(3'')).

^{13}C NMR (DMF, 126 MHz): δ 173.58 (C(1'')), 170.79 (C(1''')), 158.10 (C(3''')), 151.04 (C(2''')), 146.25 (C-arom), 145.12 (C-arom), 131.97 (C-arom), 127.06 (CH-arom), 125.62 (CH-arom), 125.14 (C-arom), 123.46 (CH-arom), 123.45 (CH-arom), 117.61 (CH-arom), 103.75 (CH-arom), 45.16 (C(1')), 39.22 (C(2')), 35.39 (C(4'')), 27.59 (C(2'')), 22.93 (C(3'')).

ES$^-$-HRMS: calculated for $C_{19}H_{19}N_2O_5S$ ($[M]^-$) 387.1015, found 387.1019.

3.10 CPO-DBCO

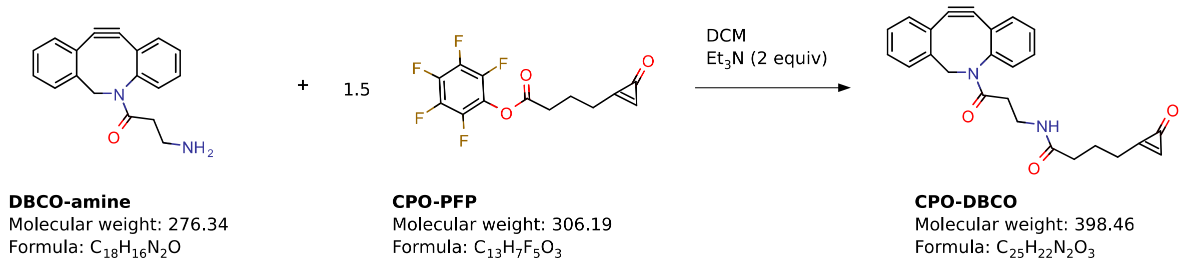

In a fumehood, **DBCO-amine** (14 mg, 0.051 mmol, 1.0 equiv) and **CPO-PFP** (23 mg, 0.076 mmol, 1.5 equiv) were weighed into a glass vial (2 mL) and dissolved in DCM (1.5 mL). To the reaction mixture was added triethylamine (10.8 mg, 15 µL, 0.102 mmol, 2 equiv) and the resulting mixture was stirred for 2.5 h at ambient temperature (23 °C). Silica gel (ca. 2 mL) was added to the reaction mixture and volatile material was removed from the mixture to give a fine powder. The material was purified by column chromatography (DCM/MeOH; 98:2→95:5) to provide **CPO-DBCO** as a yellow oil (18 mg, 0.045 mmol, 88%).

TLC (DCM/MeOH 95:5) Rf= 0.38.

^{1}H NMR (400 MHz, CDCl3) δ 8.44 (s, 1H), 7.75–7.66 (m, 1H), 7.51–7.25 (m, 8H), 6.28 (s, 1H), 5.16 (d, J = 14.0 Hz, 1H), 3.72 (d, J = 13.9 Hz, 1H), 3.44–3.19 (m, 2H), 2.68 (td, J = 7.1, 1.4 Hz, 2H), 2.48 (ddd, J = 16.7, 7.4, 3.9 Hz, 1H), 2.22–1.90 (m, 5H).

^{13}C NMR (101 MHz, CDCl3) δ 172.26, 171.39, 169.57, 157.77, 151.08, 148.60, 148.12, 132.26, 129.16, 128.78, 128.63, 128.51, 128.03, 127.42, 125.69, 123.05, 122.62, 114.86, 107.91, 77.48, 77.16, 76.84, 55.68, 35.42, 34.97, 34.82, 26.95, 21.59.

HRMS m/z (formula, ion, relative intensity): 399.1732 ($C_{25}H_{22}N_2O_3$, $[M+H]^+$, 100%).

3.11 CPO-N_3

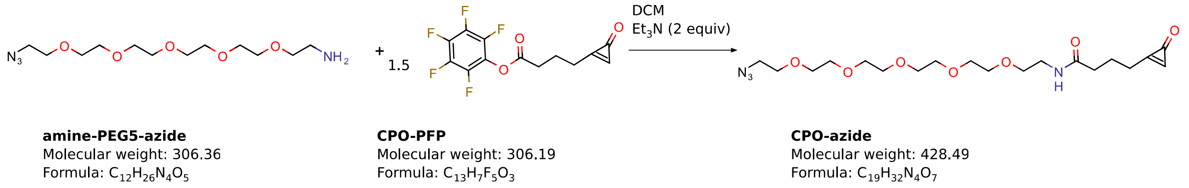

In a fumehood, **amine-PEG$_5$-azide** (20 mg, 0.065 mmol, 1.0 equiv) and **CPO-PFP** (30 mg, 0.098 mmol, 1.5 equiv) were weighed into a glass vial (2 mL) and dissolved in DCM (1.5 mL). To the reaction mixture was added triethylamine (13 mg, 17 µL, 0.13 mmol, 2 equiv) and the resulting mixture was stirred for 2.5 h at ambient temperature (23 °C). Silica gel (2 mL) was added to the reaction mixture and volatile material was removed from the mixture to give a fine powder. The material was purified by column chromatography (DCM/MeOH; 98:2→95:5) to provide **CPO-N$_3$** as a yellow oil (22 mg, 0.051 mmol, 78%).

TLC (DCM/MeOH 93:7) R_f= 0.15.

^1H NMR (400 MHz, $CDCl_3$) δ 8.47 (s, 1H), 6.68 (s, 1H), 3.68–3.63 (m, 14H), 3.61 (q, J = 1.8 Hz, 4H), 3.54 (dd, J = 5.6, 4.5 Hz, 2H), 3.44 (t, J = 4.5 Hz, 2H), 3.37 (t, J = 5.1 Hz, 2H), 2.75 (t, J = 7.1 Hz, 2H), 2.35 (t, J = 7.1 Hz, 2H), 2.06 (p, J = 7.1 Hz, 2H).

^{13}C NMR (101 MHz, $CDCl_3$) δ 171.83, 169.63, 158.00, 148.50, 77.16, 70.78, 70.76, 70.69, 70.63, 70.31, 70.14, 69.84, 50.80, 39.39, 34.84, 26.93, 21.70.

HRMS m/z (formula, ion, relative intensity): 429.2346 ($C_{19}H_{32}N_4O_7$, $[M+H]^+$,100%).

3.12 CAA-BN

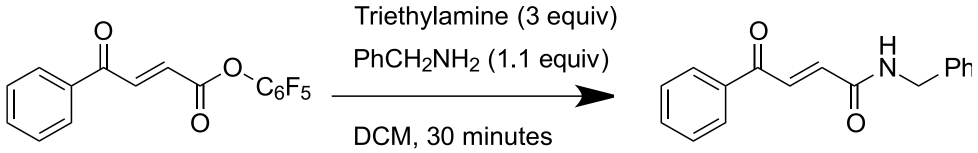

CAA-PFP **CAA-BN**

To a DCM (2 mL) solution of benzoacrylic acid pentafluorophenyl ester (68 mg, 0.2 mmol, 1 equiv) was added triethylamine (80 µL, 0.6 mmol, 3 equiv) followed by benzylamine (24 µL, 0.22 mmol, 1.1 equiv). The reaction was stirred at room temperature for 30 minutes then the reaction mixture was concentrated to give an oil which was purified by CC (DCM) to give **CAA-BN** as a yellow oil (34 mg, 0.12 mmol, 63% yield).

^{1}H NMR (400 MHz, $CDCl_3$) δ 8.08–7.91 (m, 3H), 7.61 (t, J = 7.4 Hz, 1H), 7.49 (t, J = 7.6 Hz, 2H), 7.33 (hept, J = 6.8 Hz, 5H), 7.02 (d, J = 15.0 Hz, 1H), 6.39 (s, 1H), 4.60 (d, J = 5.7 Hz, 2H). NMR data were consistent with those in the literature.[1]

4 X-ray structure analysis of 2a

4.1 Methods

Single-crystal X-ray diffraction data were collected using a D8-QUEST PHOTON-100 diffractometer equipped with an Incoatec IμS Cu microsource (CuKα radiation, λ= 1.5418 Å). The temperature was held at 180(2) K using an Oxford Cryosystems N_2 cryostat. Data integration and reduction were undertaken with SAINT in the APEX3 software suite. Multi-scan corrections were applied using SADABS. Structures were solved using SHELXT and refined using SHELXL.[2, 3]

4.2 X-Ray Crystallography

The crystal structure of compound **2a** is refined as a 2-component non-merohedral twin (HKLF-5 refinement in SHELXL). The benzene ring C11–C16 is modelled as disordered over two components. The intermolecular contacts indicate that the two components must alternate along the short *b* axis, and the site occupancies of both components are therefore constrained to 0.5. There was no clear evidence of doubling of this cell axis. The displacement ellipsoid of atom C8 indicates clearly that this site has partial site occupancy. The atoms makes unreasonably short contacts to its symmetry equivalent generated by the action of 2-fold rotation axis, so its site occupancy factor was also constrained to 0.5. The disorder represents a mixture of EtO and MeO substituents.

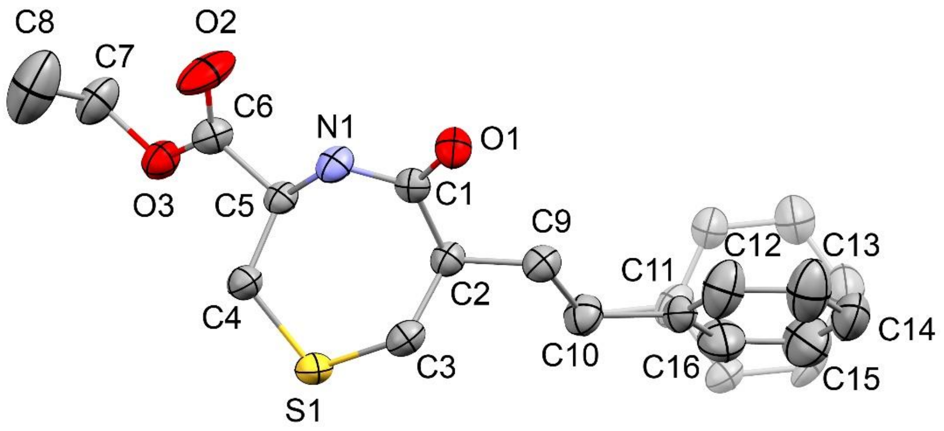

Figure S1 Molecular structure of **2a** showing displacement ellipsoids at 50% probability. H atoms are omitted. One disorder component of the benzene ring C11–C16 is shown as semi-transparent. Atom C8 has 50% site occupancy.

Table S1. Crystal data, data collection, and refinement parameters for **2a.**

Compound	**2a**
CCDC Deposition No.	2010531
Cambridge Data No.	GB_B1_0001
Formula	$C_{16}H_{21}NO_3S$, $C_{15}H_{19}NO_3S$
M.w.	600.77
Crystal system	monoclinic
Space group (IT no.)	*C*2 (5)
a [Å]	22.5982 (14)
b [Å]	5.2231 (3)
c [Å]	13.4924 (8)
α [°]	90
β [°]	106.471 (4)
γ [°]	90
Z	2
V [Å^3]	1527.19(16)
D_x [g cm^{-3}]	1.306
Crystal size [mm]	0.30 × 0.05 × 0.01
Crystal colour, shape	colourless lath
μ [mm^{-1}]	1.954
T_{min},T_{max}	0.592, 0.981
Measured reflections	17502
Independent reflections (R_{int}^a)	2572 (0.065)
Observed reflections [I>2σ(I)]	2294
No. of parameters / restraints	226 / 147
$R1$ (observed data)^b	0.065
$wR2(F^2)$ for all data	0.165
GOF^c	1.05
Residual electron density [e·Å^{-3}]	–0.26, 0.30
Flack parameter	0.04 (5)

^{a} $R_{int} = \Sigma|F_o^2 - F_{o,mean}^2|/\Sigma F_o^2$

^{b} $R(F) = \Sigma||F_o| - |F_c||/\Sigma|F_o|$; $wR(F^2) = [\Sigma(w(F_o^2 - F_c^2)^2)/(\Sigma w(F_o^2)^2)]^{½}$

^{c} $GOF = [\Sigma(w(F_o^2 - F_c^2)^2)/(N_{diffrs} - N_{params})]^{½}$

5 Quantum Mechanical Calculations

Full geometry optimizations were carried out with Gaussian 16[4] using the M06-2X[5] hybrid functional and 6-31+G(d,p) basis set in combination with ultrafine integration grids. Bulk solvent effects in water were considered implicitly through the IEF-PCM polarizable continuum model.[6] The possibility of different conformations was taken into account. Frequency analyses were carried out at the same level used in the geometry optimizations, and the nature of the stationary points was determined in each case according to the appropriate number of negative eigenvalues of the Hessian matrix. The quasiharmonic approximation reported by Truhlar *et al.* was used to replace the harmonic oscillator approximation for the calculation of the vibrational contribution to enthalpy and entropy.[7] Scaled frequencies were not considered. Mass-weighted intrinsic reaction coordinate (IRC) calculations were carried out by using the Hratchian and Schlegel algorithm[8] in order to ensure that the TSs indeed connected the appropriate reactants and products. Gibbs free energies (ΔG) were used for the discussion on the relative stabilities of the considered structures. Free energies calculated using the gas phase standard state concentration (1 atm = 1/24.5 M) were converted to reproduce the standard state concentration in solution (1 M) by adding or subtracting 1.89 kcal mol^{-1} for bimolecular additions and decompositions, respectively. The lowest energy conformer for each calculated stationary point was considered in the discussion; all the computed structures can be obtained from authors upon request.

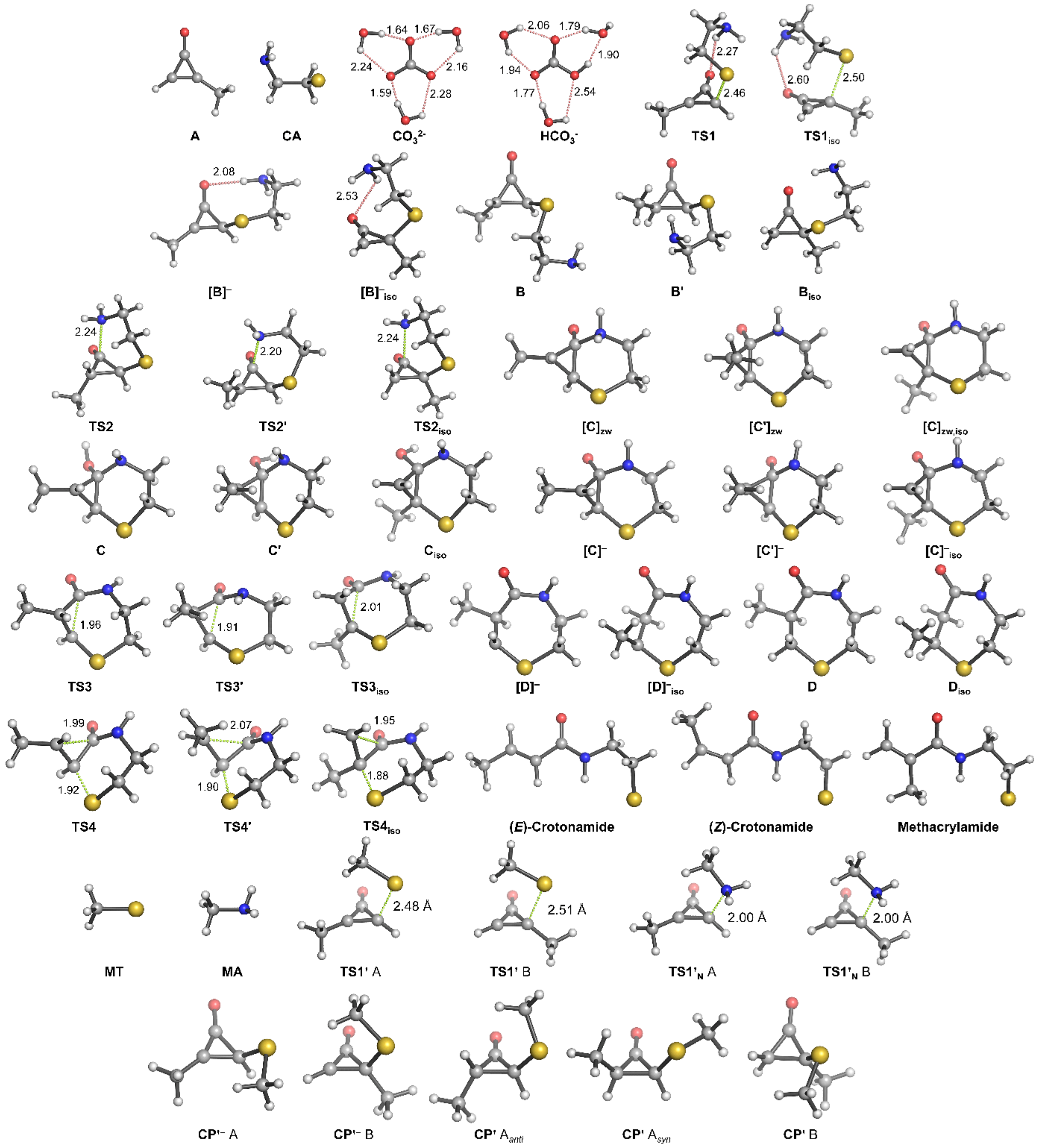

Figure S2 Lowest-energy structures calculated with PCM(H_2O)/M06-2X/6-31+G(d,p)

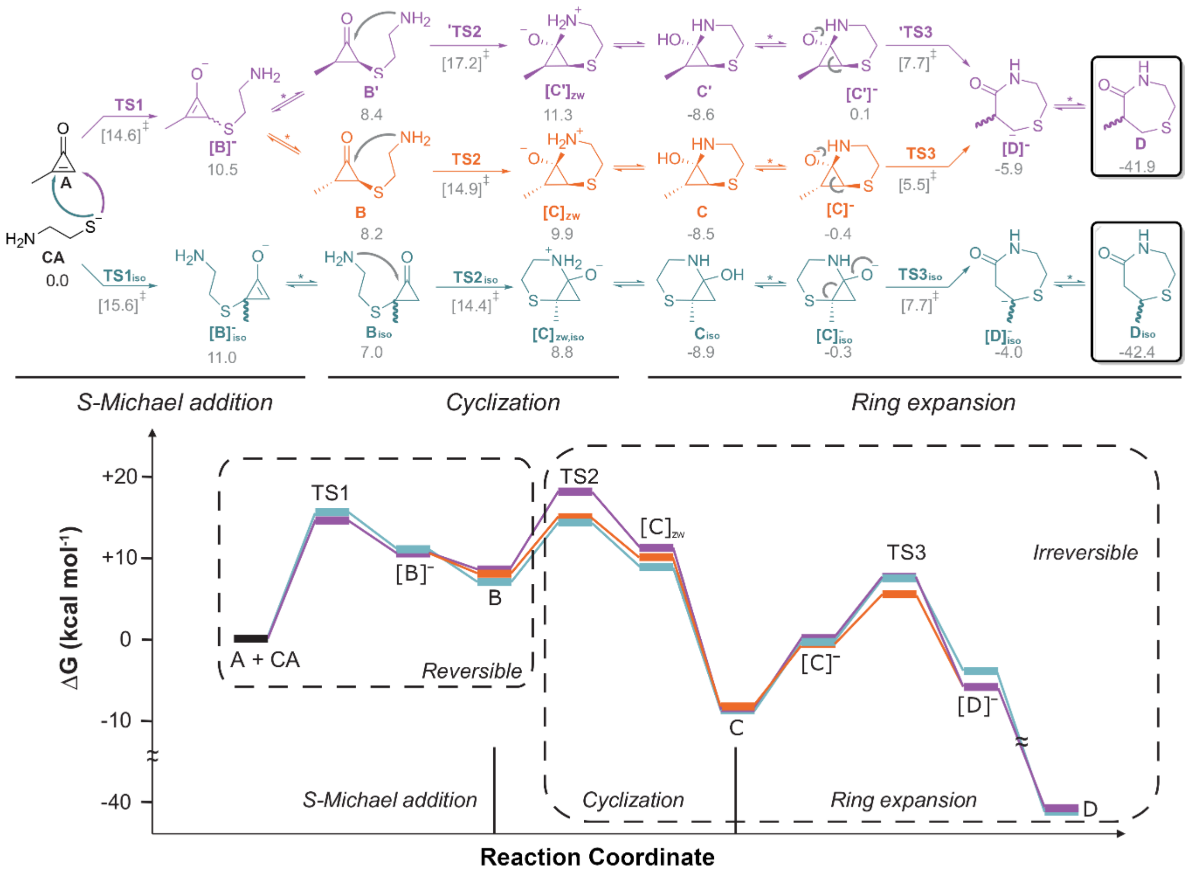

Figure S3 a) Minimum energy pathways for the reaction between 2-methylcyclopropenone (**1'**) and cysteamine (**CA**) calculated with PCM(H_2O)/M06-2X/6-31+G(d,p). Note that each calculated structure represents a racemic compound constituted by two enantiomers with identical energies. *: protonation and deprotonation equilibria were calculated using tri-hydrated bicarbonate ($HCO_3^{2-} \cdot 3\ H_2O$) and carbonate ($CO_3^{2-} \cdot 3$ H2O) anions as an acid and a base, respectively. Given the intrinsic inaccuracy of such estimations, relative energies of charges/neutral species must be considered with caution. Relative free energies (ΔG) are given in kcal mol^{-1}. Numbers in brackets represent the relative energies of transition structures.

Note that the purple path corresponding to S-Michael addition and the red path of the cyclization and ring expansion phases constitute the minimum energy pathway depicted in the main text.

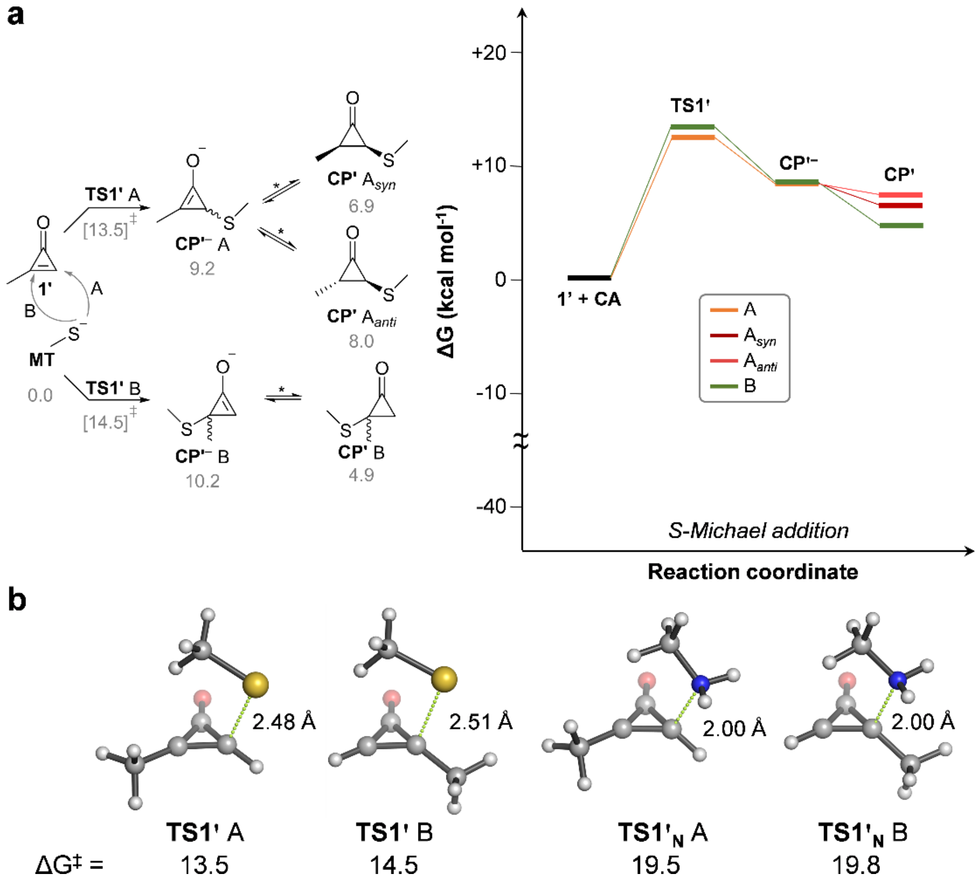

Figure S4 a) Minimum energy pathways for the reaction between 2-methylcyclopropenone (**1'**) and methanethiolate (**MT**) calculated with PCM(H_2O)/M06-2X/6-31+G(d,p). Note that each calculated structure represents a racemic compound constituted by two enantiomers with identical energies. b) Lowest energy transition structures calculated at the same level for the reactions between **1'** and methanethiolate (**TS1'** A and **TS1'** B) or methylamine (**TS1$_N$'** A and **TS1$_N$'**). *: protonation and deprotonation equilibria were calculated using tri-hydrated bicarbonate (HCO_3^{2-} · 3 H_2O) and carbonate (CO_3^{2-} · 3 H2O) anions as an acid and a base, respectively. Given the intrinsic inaccuracy of such estimations, relative energies of charges/neutral species must be considered with caution. Relative free energies (ΔG) are given in kcal mol^{-1}. Numbers in brackets represent the relative energies of transition structures.

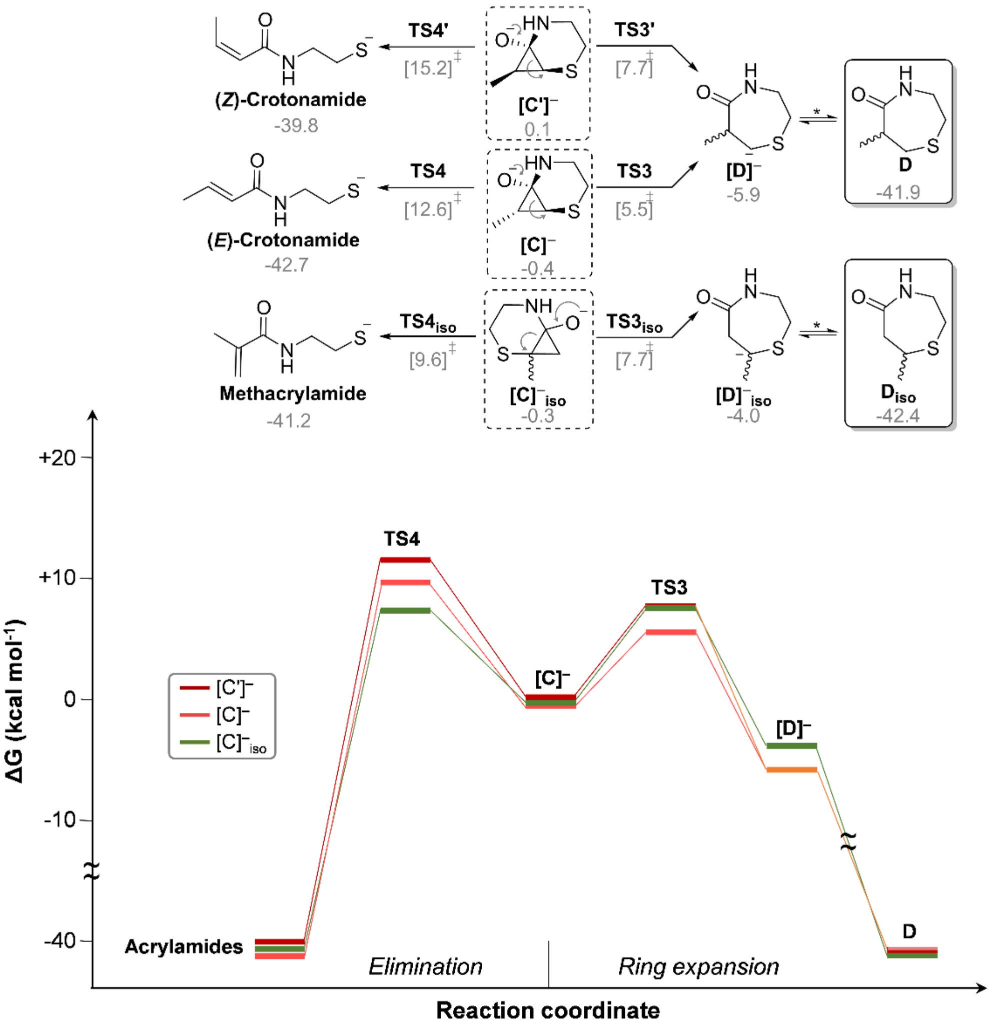

Figure S5 Comparison between the ring-expansion reaction pathways (**TS3**) to give the experimentally observed 1,4-thiazepan-5-ones (**D**), and the elimination reaction pathways (**TS4**) to afford non-detected acrylamides, calculated with PCM(H_2O)/M06-2X/6-31+G(d,p). Both reaction channels are accessible from the same deprotonated hemiaminal intermediates (**[C]**$^-$). *: protonation and deprotonation equilibria were calculated using tri-hydrated bicarbonate (HCO_3^{2-} · 3 H_2O) and carbonate (CO_3^{2-} · 3 H_2O) anions as an acid and a base, respectively. Given the intrinsic inaccuracy of such estimations, relative energies of charges/neutral species must be considered with caution. Relative free energies (ΔG) are given in kcal mol^{-1}. Numbers in brackets represent the relative energies of transition structures.

Table S2. Energies, entropies, and lowest frequencies of the lowest energy calculated structures.

Structure	E_{elec} (Hartree)[a]	E_{elec} + ZPE (Hartree)[a]	H (Hartree)[a]	S (cal mol^{-1} K^{-1})[b]	G (Hartree)[a,b]	Lowest freq. (cm^{-1})	# of imag. freq.
A	–229.894568	–229.827621	–229.822095	71.2	–229.853405	86.0	0
CA	–532.803161	–532.718809	–532.713469	70.3	–532.744529	161.5	0
$CO_3^{2–}$	–493.241249	–493.153831	–493.142707	106.1	–493.186493	21.0	0
$HCO_3^{–}$	–493.724975	–493.624401	–493.612104	111.2	–493.658113	26.9	0
TS1	–762.697276	–762.544737	–762.534279	101.4	–762.577754	–203.9	1
$TS1_{iso}$	–762.696075	–762.543708	–762.533464	99.1	–762.576138	–198.1	1
$[B]^{–}$	–762.704939	–762.551431	–762.541039	99.6	–762.584242	54.1	0
$[B]^{–}_{iso}$	–762.703922	–762.551200	–762.540847	99.1	–762.583429	27.3	0
B	–763.193541	–763.026745	–763.016203	101.2	–763.059447	36.9	0
B'	–763.193715	–763.026639	–763.016390	97.8	–763.059106	63.3	0
B_{iso}	–763.195992	–763.029221	–763.018959	97.2	–763.061417	62.5	0
TS2	–763.185246	–763.017571	–763.008353	92.0	–763.048752	–163.1	1
TS2'	–763.182180	–763.014015	–763.004984	90.9	–763.045077	–227.8	1
$TS2_{iso}$	–763.186291	–763.018828	–763.009757	90.6	–763.049682	–160.4	1
$[C]_{zw}$	–763.197557	–763.026260	–763.017442	90.5	–763.056830	51.7	0
$[C']_{zw}$	–763.195426	–763.024040	–763.015438	88.6	–763.054511	91.5	0
$[C]_{zw,iso}$	–763.199218	–763.028153	–763.019456	88.8	–763.058533	85.7	0
C	–763.225435	–763.055351	–763.046300	90.8	–763.086129	71.9	0
C'	–763.225867	–763.055648	–763.046739	89.6	–763.086305	117.0	0
C_{iso}	–763.226112	–763.056312	–763.047413	89.4	–763.086804	90.7	0

$[C]^{–}$	–762.727403	–762.571089	–762.562409	89.8	–762.601515	53.8	0
$[C']^{–}$	–762.727193	–762.570507	–762.562032	88.2	–762.600794	79.6	0
$[C]^{–}_{iso}$	–762.727376	–762.571277	–762.562659	89.4	–762.601461	46.3	0
TS3	–762.716572	–762.561878	–762.553359	87.9	–762.592162	–444.8	1
TS3'	–762.713517	–762.558268	–762.549783	88.2	–762.588623	–478.1	1
$TS3_{iso}$	–762.712731	–762.558312	–762.549787	88.4	–762.588715	–383.2	1
$[D]^{–}$	–762.735858	–762.579768	–762.570995	89.3	–762.610385	95.9	0
$[D]^{–}_{iso}$	–762.732639	–762.576799	–762.568003	89.3	–762.607399	95.9	0
D	–763.279806	–763.108658	–763.099819	90.1	–763.139395	79.6	0
D_{iso}	–763.281038	–763.109631	–763.100892	89.3	–763.140201	85.7	0
TS4	–762.703919	–762.549917	–762.541109	89.9	–762.580819	–333.8	1
TS4'	–762.701186	–762.546553	–762.538154	87.2	–762.576670	–362.4	1
$TS4_{iso}$	–762.709512	–762.555732	–762.547245	87.1	–762.585720	–413.5	1
(E)-Crotonamide	–762.792298	–762.636320	–762.626139	100.6	–762.669065	37.5	0
(Z)-Crotonamide	–762.787855	–762.631682	–762.621500	101.7	–762.664396	24.9	0
Methacrylamide	–762.790467	–762.634338	–762.624416	99.5	–762.666527	25.5	0
MT	–438.170103	–438.133138	–438.129472	55.2	–438.153846	715.7	0
TS1' A	–668.063096	–667.957948	–667.949298	91.7	–667.988765	–194.5	1
TS1' B	–668.062025	–667.956994	–667.948546	89.3	–667.987212	–198.0	1
$CP'^{–}$ A	–668.071038	–667.965047	–667.956305	92.0	–667.995658	26.4	0
$CP'^{–}$ B	–668.070953	–667.965492	–667.956962	88.6	–667.995543	55.4	0
CP' A_{anti}	–668.558509	–668.439023	–668.430411	90.2	–668.469169	31.1	0
CP' A_{syn}	–668.560304	–668.440529	–668.431914	90.1	–668.470901	43.9	0

CP' B	–668.563369	–668.443998	–668.435535	87.8	–668.474020	71.5	0
MA	–95.815844	–95.751246	–95.747258	56.6	–95.772265	303.9	0
TS1'$_N$ A	–325.702132	–325.567645	–325.559027	90.6	–325.597637	–295.5	1
TS1'$_N$ B	–325.702159	–325.567715	–325.559485	86.3	–325.597134	–296.1	1

[a]Energy values calculated with PCM$_{H2O}$/M06-2X/6-31+G(d,p). 1 Hartree = 627.51 kcal mol-1. [b]Thermal corrections at 277.15 K.

5.1 Cartesian coordinates of the lowest-energy calculated structures.

A

C	0.553927	0.106997	-0.000058
C	-0.200592	1.228355	-0.000011
C	-0.859919	-0.019001	0.000013
O	-1.880816	-0.693094	0.000006
C	1.917424	-0.464407	0.000003
H	2.044666	-1.099026	0.880794
H	2.044734	-1.099262	-0.880591
H	2.674736	0.319984	-0.000012
H	-0.182645	2.311389	0.000077

CA

S	1.494857	-0.317705	-0.062999
C	0.145407	0.882075	0.293348
H	0.428395	1.878346	-0.066373
H	-0.000133	0.962880	1.378788
C	-1.190719	0.499276	-0.347470
H	-1.938612	1.270946	-0.123543
H	-1.068182	0.473856	-1.437271
H	-0.986120	-1.478995	-0.025911
N	-1.735146	-0.793686	0.076122
H	-1.935172	-0.756055	1.074171

CO_3^{2-}

C	0.053655	-0.081861	-0.000940
O	0.281550	-1.340444	-0.001466
O	1.023036	0.778619	0.001399
O	-1.175651	0.365905	-0.002758
O	3.104947	-0.768906	0.000440
O	-0.807729	2.967098	0.000635
O	-2.492021	-1.923540	0.002049
H	2.553996	-1.563766	-0.000579
H	2.367579	-0.078622	0.000943
H	0.151343	2.839708	0.002847
H	-1.080834	2.003545	-0.001514
H	-2.162001	-0.982357	0.000098
H	-1.625069	-2.357191	0.001457

HCO_3^-

C	-0.011258	0.045057	-0.018193
O	-1.081607	0.706512	-0.027765
O	1.159465	0.496492	-0.008912
O	-0.098963	-1.321056	-0.018236
O	0.323411	3.177070	0.022322
O	2.871284	-1.656853	0.022788
O	-2.911116	-1.257834	-0.070052
H	-0.515467	2.689693	0.005524
H	0.942491	2.425356	0.016546
H	2.316596	-0.846175	0.011112
H	2.220082	-2.367566	0.014222
H	-1.042123	-1.576954	-0.015973
H	-2.513395	-0.361251	-0.007902
H	-3.440421	-1.388092	0.724467

TS1

C	-1.931297	-0.017403	0.123667
C	-1.269200	-0.199365	-1.085318
C	-1.248875	1.057366	-0.401308
O	-0.858604	2.239362	-0.368652
C	-2.777412	-0.686973	1.142535
H	-2.223469	-1.523785	1.582878
H	-3.059663	0.000591	1.941442
H	-3.684391	-1.097073	0.688434
H	-1.423639	-0.670130	-2.046328
S	0.846389	-1.434770	-0.835568
C	1.178044	-0.798847	0.836074
H	1.385697	-1.629816	1.519438
H	0.258920	-0.309928	1.202523
C	2.330469	0.210867	0.907511
H	2.436076	0.550543	1.945965
H	3.267196	-0.291373	0.638006
N	2.185811	1.388943	0.051671
H	2.085592	1.054014	-0.906190
H	1.293237	1.841918	0.251457

$TS1_{iso}$

C	-1.323928	0.636680	1.316182
C	-1.594853	-0.097026	0.166779
C	-1.173237	1.269832	0.098709
O	-0.902850	2.262172	-0.593732
S	0.363119	-1.522141	-0.464762
C	1.504563	-0.824931	0.766139
H	1.965525	-1.629676	1.349371
H	0.908607	-0.218117	1.471308
C	2.600847	0.066677	0.173336
H	3.251459	0.421402	0.982911
H	3.225155	-0.528547	-0.503375
N	2.112994	1.233459	-0.563933
H	1.429512	0.901768	-1.244654
H	1.570570	1.824247	0.064466
C	-2.627599	-1.044864	-0.348105
H	-2.426303	-1.319342	-1.384762
H	-2.664735	-1.950719	0.259783
H	-3.603705	-0.547768	-0.297887
H	-1.348915	0.631215	2.398177

$[B]^-$

C	2.094457	0.085901	0.140741
C	0.717036	-0.224533	0.594566
C	1.203488	1.054296	-0.025853
O	0.780126	2.189528	-0.406028
C	3.446010	-0.527957	0.035425
H	3.830172	-0.832616	1.016604
H	3.432224	-1.423499	-0.598021
H	4.160587	0.175977	-0.400686
H	0.436387	-0.331256	1.653320
S	-0.337682	-1.408468	-0.388397
C	-1.997525	-1.084496	0.294992
H	-2.600535	-1.964290	0.047907
H	-1.911760	-1.045681	1.388695
C	-2.697358	0.177180	-0.219862
H	-2.702624	0.160449	-1.315523
H	-3.745469	0.128601	0.103148
H	-2.112128	1.482850	1.233779

H	-1.187037	1.588830	-0.107622
N	-2.147795	1.456793	0.217276

$[B]^-_{iso}$

C	-1.157894	0.581619	1.342605
C	-1.276272	-0.235736	0.120587
C	-1.059817	1.237845	0.179350
O	-0.903291	2.267143	-0.533086
S	0.136781	-1.416937	-0.421372
C	1.512379	-0.940803	0.665191
H	1.067883	-0.420293	1.524144
H	1.982547	-1.859073	1.029742
C	2.556494	-0.043806	-0.005735
H	3.375146	0.123762	0.704830
H	2.985033	-0.566962	-0.867851
H	1.664665	1.757390	0.334271
H	1.276507	1.116804	-1.086224
N	2.065849	1.256588	-0.456523
C	-2.556165	-0.895954	-0.366920
H	-2.499856	-1.154071	-1.431471
H	-2.768087	-1.815677	0.191002
H	-3.394897	-0.205911	-0.228509
H	-1.224406	0.542783	2.421890

B

C	1.568666	0.710618	0.456861
C	0.670470	-0.204651	-0.453066
C	2.094363	-0.482415	-0.199422
O	3.021152	-1.217810	-0.414728
C	1.919678	2.104470	-0.022772
H	1.095141	2.791007	0.185455
H	2.815195	2.467269	0.486893
H	2.112906	2.110378	-1.098574
H	0.419773	0.216476	-1.426606
S	-0.552549	-1.275481	0.297800
C	-1.655636	-0.011944	1.015174
H	-2.406345	-0.578919	1.575183
H	-1.080363	0.585415	1.730485
C	-2.325836	0.892117	-0.022012
H	-1.557604	1.459340	-0.559758
H	-2.931770	1.626132	0.519976
H	-3.928472	-0.272004	-0.528191
H	-2.639102	-0.478039	-1.503465
N	-3.176446	0.224332	-1.000257
H	1.377103	0.603629	1.524841

B'

C	-1.330990	0.938479	0.681330
C	-0.868533	-0.580501	0.636288
C	-2.191211	-0.130253	0.186720
O	-3.186353	-0.431989	-0.420900
C	-0.813169	1.978570	-0.288083
H	-1.527295	2.801335	-0.369745
H	0.143034	2.383697	0.056209
H	-0.672769	1.550390	-1.284502
H	-0.817066	-1.035996	1.624587
S	0.278637	-1.256792	-0.556072
C	1.833252	-1.088651	0.387548
H	1.715704	-1.616710	1.338841
H	2.580385	-1.623559	-0.207618
C	2.269430	0.359025	0.618774
H	3.169066	0.343125	1.243180
H	1.497515	0.880277	1.196647
H	1.766039	1.097386	-1.218206
H	3.345249	0.719517	-1.079811
N	2.560386	1.133601	-0.582525
H	-1.482605	1.269907	1.706970

B_{iso}

C	2.223856	0.399644	-0.749526
C	1.120081	-0.338691	0.085406
C	1.189251	1.126907	-0.013087
O	0.687688	2.184182	0.270673
S	-0.141875	-1.115245	-0.942738
C	-1.553639	-1.145656	0.212669
H	-2.369960	-1.580401	-0.373279
H	-1.332115	-1.835367	1.032512
C	-1.962663	0.225726	0.760154
H	-1.140618	0.650764	1.346531
H	-2.793756	0.060424	1.454776
H	-3.112777	0.844691	-0.814901
H	-1.593729	1.444960	-0.837523
N	-2.372845	1.217825	-0.224978
H	2.171803	0.364079	-1.835149
C	1.544004	-1.059322	1.346871
H	1.934599	-2.049081	1.094883
H	2.322604	-0.493594	1.866892
H	0.700638	-1.175401	2.032893
H	3.226393	0.362972	-0.329281

TS2

C	-1.345989	-0.249863	0.668754
C	-0.574800	-0.725922	-0.597370
C	-1.052555	0.669435	-0.459953
O	-1.581104	1.549605	-1.118881
C	-2.762004	-0.724621	0.911922
H	-2.762272	-1.730567	1.341523
H	-3.282709	-0.055605	1.602415
H	-3.327735	-0.750622	-0.024144
H	-1.179591	-1.304303	-1.297570
S	1.086602	-1.417946	-0.603613
H	0.527468	1.944586	1.290288
N	0.786272	1.661294	0.347848
H	-0.726153	-0.149269	1.555483
C	1.961824	-0.461033	0.670752
H	1.478148	-0.604515	1.642097
H	2.947537	-0.930855	0.725445
C	2.103839	1.015711	0.336189
H	2.796389	1.482084	1.048782
H	2.529929	1.119262	-0.664676
H	0.776396	2.498801	-0.227493

TS2'

C	-1.828358	-0.309849	-0.176098
C	-0.501176	-0.488807	-1.013626
C	-0.968274	0.835075	-0.491923
O	-1.028936	1.992081	-0.876791
C	-2.015601	-0.958700	1.173958

H	-2.775547	-0.415393	1.742411
H	-2.352068	-1.992403	1.051210
H	-1.088937	-0.966360	1.745923
H	-0.643689	-0.614987	-2.084989
S	0.824726	-1.477885	-0.329329
C	2.163340	-0.262368	-0.035875
H	2.747089	-0.702969	0.777408
H	2.807331	-0.192390	-0.914497
C	1.671602	1.133359	0.346344
H	1.409104	1.697792	-0.552788
H	2.492872	1.663105	0.842983
H	0.180720	1.982947	1.485759
H	0.612234	0.476633	1.991348
N	0.462676	1.058510	1.169178
H	-2.721160	-0.328293	-0.802097

TS2$_{iso}$

C	-0.994529	0.422558	1.369972
C	-0.949522	-0.449297	0.080832
C	-0.981648	1.028956	0.012714
O	-1.501413	1.929941	-0.623638
S	0.432702	-1.558147	-0.284102
H	1.349877	2.087365	0.732981
N	1.196912	1.546298	-0.114861
H	-0.098234	0.464615	1.978810
C	1.897501	-0.736762	0.407795
H	1.762972	-0.575810	1.481810
H	2.697842	-1.472131	0.287811
C	2.259328	0.554467	-0.309655
H	3.230059	0.912732	0.056006
H	2.355096	0.354842	-1.379551
H	1.152999	2.206271	-0.885866
C	-2.246327	-1.128701	-0.325468
H	-2.234874	-1.380187	-1.390131
H	-2.390768	-2.046801	0.252617
H	-3.095583	-0.464970	-0.142576
H	-1.928530	0.433481	1.929723

[C]$_{zw}$

C	-1.498594	-0.449710	0.513140
C	-0.413852	-0.720694	-0.540114
C	-0.731071	0.700584	-0.069552
O	-1.182467	1.661262	-0.807481
C	-2.948445	-0.606354	0.128321
H	-3.242705	-1.659986	0.123636
H	-3.593827	-0.072492	0.832381
H	-3.121299	-0.188677	-0.866516
H	-0.756135	-0.959073	-1.543876
S	1.101054	-1.508739	-0.008713
C	2.294617	-0.130950	-0.218145
H	3.089268	-0.333519	0.502642
H	2.727838	-0.159946	-1.218555
C	1.689196	1.260128	0.018329
H	1.379275	1.727070	-0.917090
H	2.404804	1.909914	0.522247
H	0.167038	2.125776	1.139906
H	0.581549	0.612253	1.670114
N	0.442251	1.188841	0.835476
H	-1.239797	-0.791499	1.514162

[C']$_{zw}$

C	-1.397132	-0.972009	0.024435
C	-0.290939	-0.608987	-0.973725
C	-1.138297	0.474609	-0.318433
O	-1.931423	1.280461	-0.954854
H	-0.531064	-0.742415	-2.023820
S	1.454311	-0.939039	-0.685924
C	1.925386	0.389800	0.483763
H	1.931953	0.003840	1.507869
H	2.947253	0.676242	0.230037
C	1.034931	1.612126	0.397614
H	0.981758	2.014244	-0.616206
H	1.390333	2.386606	1.079177
H	-0.929273	2.093728	0.886594
H	-0.361478	0.775434	1.694905
N	-0.357515	1.250300	0.786919
H	-2.304711	-1.347410	-0.444198
C	-1.057366	-1.627511	1.342881
H	-1.800758	-1.387298	2.109279
H	-1.029617	-2.715079	1.227300
H	-0.068884	-1.337228	1.715036

[C]$_{zw,iso}$

C	-1.488674	0.215797	1.199323
C	-0.806302	-0.599667	0.100497
C	-0.742892	0.926106	0.093075
O	-1.215847	1.710790	-0.821123
S	0.738549	-1.447129	0.494593
C	1.727787	-0.484425	-0.700251
H	2.739624	-0.891735	-0.702995
H	1.294479	-0.643526	-1.689553
C	1.763391	1.016686	-0.363457
H	1.622924	1.617812	-1.261563
H	2.700926	1.301253	0.114854
H	0.599498	2.406296	0.650717
H	0.877306	1.020827	1.507341
N	0.662212	1.387859	0.577491
H	-1.089252	0.157693	2.208646
C	-1.663045	-1.270299	-0.947022
H	-1.087332	-1.455197	-1.860272
H	-2.045971	-2.227394	-0.580567
H	-2.507339	-0.626048	-1.205345
H	-2.571955	0.267566	1.138805

C

C	-1.279393	-0.505235	0.663268
C	-0.342410	-0.740772	-0.504322
C	-0.703279	0.656817	-0.106763
O	-1.522773	1.303265	-1.045127
C	-2.734567	-0.884837	0.536079
H	-2.874265	-1.949286	0.745127
H	-3.350683	-0.315046	1.238098
H	-3.095851	-0.680361	-0.474819
H	-0.820799	-1.083804	-1.417992
S	1.328894	-1.396622	-0.377203
C	2.196821	-0.023864	0.467302
H	2.141613	-0.160772	1.551354
H	3.241956	-0.111647	0.161889

C	1.629179	1.339330	0.086485
H	1.676781	1.481542	-0.998275
H	2.237304	2.119886	0.552590
N	0.245586	1.521019	0.517369
H	-0.835435	-0.653191	1.647274
H	-1.888924	2.087055	-0.611957
H	0.170975	1.489685	1.529095

C'

C	-1.284171	-1.034727	0.065829
C	-0.283296	-0.546362	-0.969002
C	-1.074114	0.433966	-0.138358
O	-2.154831	1.055161	-0.790833
H	-0.591363	-0.619939	-2.006525
S	1.497316	-0.771941	-0.812039
C	1.922964	0.429679	0.501522
H	1.927807	-0.068346	1.475396
H	2.940094	0.761406	0.280266
C	0.965779	1.615771	0.522655
H	0.994988	2.131121	-0.443594
H	1.298424	2.326069	1.284748
N	-0.417438	1.258468	0.814293
H	-1.844242	1.906982	-1.126977
H	-0.550286	0.951738	1.772235
H	-2.212315	-1.401072	-0.366989
C	-0.842431	-1.744953	1.320777
H	-1.604558	-1.663717	2.101365
H	-0.675880	-2.806201	1.115130
H	0.092605	-1.337793	1.713653

C_{iso}

C	1.005211	0.061191	1.395343
C	0.555413	0.726482	0.110751
C	0.851515	-0.756011	0.147019
O	2.075480	-1.165889	-0.406491
S	-1.134482	1.364424	-0.050893
C	-2.105971	-0.159765	0.167991
H	-2.220149	-0.392530	1.231410
H	-3.093318	0.059208	-0.245798
C	-1.447474	-1.322786	-0.566126
H	-1.288841	-1.048478	-1.614909
H	-2.113257	-2.190164	-0.546685
N	-0.177461	-1.736407	0.015418
H	1.888577	-1.508454	-1.291314
H	-0.312435	-2.252942	0.878613
C	1.529053	1.645032	-0.596248
H	1.331714	1.664579	-1.672217
H	1.436566	2.664609	-0.208638
H	2.553477	1.305132	-0.435680
H	0.267420	-0.096025	2.177105
H	2.013863	0.281387	1.734014

$[C]^-$

C	-1.402676	-0.449597	0.589421
C	-0.376780	-0.685002	-0.511534
C	-0.741028	0.747348	-0.068623
O	-1.397794	1.571322	-0.875470
H	-0.788112	-0.951787	-1.482131
S	1.210650	-1.475271	-0.211181
C	2.296593	-0.055624	0.235448
H	2.554787	-0.148091	1.293510
H	3.209344	-0.140170	-0.359611
C	1.617421	1.285248	-0.001566
H	1.479697	1.448937	-1.082582
H	2.283137	2.070229	0.372042
H	0.078271	2.313843	0.843358
N	0.348467	1.340305	0.725362
H	-1.037631	-0.687656	1.587578
C	-2.848667	-0.784882	0.321331
H	-3.038946	-1.859524	0.423859
H	-3.513996	-0.257960	1.014901
H	-3.123043	-0.481149	-0.692666

$[C']^-$

C	-1.438302	-0.900567	-0.005695
C	-0.305058	-0.528126	-0.961353
C	-1.087494	0.564121	-0.210816
O	-1.904146	1.378149	-0.876901
H	-0.536802	-0.571327	-2.021312
S	1.415592	-0.964053	-0.660526
C	1.964712	0.370085	0.477049
H	1.994579	-0.024441	1.496705
H	2.977747	0.645527	0.172835
C	1.035014	1.573230	0.433988
H	1.039380	2.008641	-0.579534
H	1.424357	2.328452	1.124822
H	-0.831928	2.018018	1.110322
N	-0.305213	1.183418	0.867261
H	-2.349715	-1.206428	-0.519199
C	-1.138096	-1.663091	1.259852
H	-1.967575	-1.580505	1.970916
H	-0.972954	-2.727945	1.054857
H	-0.241562	-1.268163	1.744230

$[C]^-_{iso}$

C	-1.294511	-0.194613	1.314031
C	-0.589541	-0.724352	0.068072
C	-0.941577	0.770849	0.195762
O	-1.871281	1.322631	-0.574923
S	1.114612	-1.321725	0.207742
C	2.135164	0.168577	-0.147697
H	2.689430	0.416397	0.761258
H	2.845290	-0.090928	-0.936699
C	1.278650	1.357480	-0.554778
H	0.839971	1.180969	-1.550903
H	1.928870	2.235514	-0.627357
H	-0.078572	2.551419	0.380336
N	0.251299	1.592702	0.458725
H	-0.716020	-0.166520	2.233137
C	-1.409394	-1.543964	-0.902550
H	-0.988296	-1.487964	-1.912855
H	-1.445798	-2.598988	-0.606175
H	-2.429124	-1.152764	-0.936111
H	-2.341128	-0.473354	1.422765

TS3

C	-1.148328	-0.469522	0.549082
C	-0.234926	-0.992624	-0.545045

C	-0.946058	0.798239	-0.181919
O	-1.782908	1.273694	-1.012782
H	-0.676907	-1.377928	-1.457492
S	1.434166	-1.371454	-0.202344
C	2.179997	0.302374	-0.132320
H	3.190085	0.181492	0.272228
H	2.251976	0.724775	-1.140977
C	1.371669	1.237238	0.771617
H	1.956684	2.142648	0.965094
H	1.212145	0.753120	1.741440
N	0.085637	1.688693	0.237363
H	0.220449	2.357600	-0.515326
H	-0.653066	-0.496907	1.526127
C	-2.560732	-1.019404	0.609937
H	-2.543682	-2.053611	0.969282
H	-3.199685	-0.428687	1.276017
H	-3.010586	-1.007447	-0.386295

TS3'

C	-1.653935	-0.174889	-0.269262
C	-0.438703	-0.743185	-0.988639
C	-0.729149	0.974668	-0.198739
O	-0.753901	1.976563	-0.995019
H	-0.419128	-0.679288	-2.071361
S	0.918676	-1.494374	-0.203195
C	2.101944	-0.068363	-0.073067
H	3.015769	-0.451135	0.390789
H	2.337773	0.222110	-1.099636
C	1.532789	1.121041	0.727704
H	1.739207	2.056357	0.200957
H	2.017416	1.178951	1.706220
N	0.081837	1.058475	0.972565
H	-0.100099	0.269663	1.583963
H	-2.443209	0.032394	-0.994890
C	-2.203462	-0.889403	0.953214
H	-2.769304	-1.771374	0.633529
H	-1.406210	-1.246060	1.612361
H	-2.869588	-0.242668	1.534113

$TS3_{iso}$

C	0.990451	-0.018777	1.254078
C	0.521726	0.968778	0.205135
C	1.014762	-0.983287	0.141568
O	2.063145	-1.227538	-0.526625
S	-1.173267	1.364238	0.149921
C	-1.939617	-0.137967	-0.583392
H	-3.022776	-0.017011	-0.478525
H	-1.693642	-0.209895	-1.648910
C	-1.487818	-1.404268	0.147130
H	-2.121303	-2.244242	-0.157040
H	-1.633919	-1.272366	1.224891
N	-0.107092	-1.820144	-0.104052
H	-0.001968	-2.196709	-1.041896
H	0.254942	-0.208656	2.042472
C	1.534790	1.726667	-0.599122
H	2.098488	1.070035	-1.289148
H	1.050725	2.502367	-1.201954
H	2.293320	2.228478	0.027982
H	1.987126	0.174621	1.672384

$[D]^-$

C	0.057271	-1.420576	-0.485892
C	-2.107788	0.562524	-0.184612
C	1.000689	-0.612303	0.416644
C	-1.196783	1.424781	0.686444
C	1.132064	0.804803	-0.120195
H	-3.136677	0.632730	0.185659
H	-1.655315	2.405236	0.844195
H	-2.086206	0.932298	-1.216382
H	-1.079277	0.962093	1.672393
O	2.105981	1.170329	-0.796010
N	0.116452	1.686184	0.105886
H	0.222231	2.558217	-0.397472
S	-1.661654	-1.233265	-0.126919
H	0.264650	-1.198279	-1.543244
H	0.553812	-0.559051	1.419152
C	2.387959	-1.246571	0.528191
H	3.083773	-0.623175	1.101292
H	2.300113	-2.220685	1.015854
H	2.815880	-1.399017	-0.467344

$[D]^-_{iso}$

C	0.419416	-1.395812	-0.367255
C	1.443674	1.211241	0.567400
C	-0.753836	-0.724282	-1.089278
C	0.271924	1.930937	-0.093451
C	-1.581373	0.197557	-0.200981
H	2.341297	1.835701	0.501868
H	0.237006	2.971815	0.241006
H	-1.475060	-1.471783	-1.444018
H	1.222297	1.041418	1.626786
H	0.412543	1.943828	-1.180602
O	-2.712354	-0.123891	0.196143
N	-1.035123	1.365210	0.225443
H	-1.615616	1.878172	0.876700
S	1.863626	-0.377893	-0.296179
C	-0.004274	-1.933291	0.992237
H	-0.841802	-2.637989	0.883281
H	0.813846	-2.474218	1.481078
H	-0.350294	-1.160246	1.713747
H	-0.390731	-0.163845	-1.960253

D

C	-0.059373	-1.322372	-0.505259
C	-2.033108	0.677108	-0.224521
C	0.964067	-0.617700	0.410281
C	-1.089084	1.519985	0.632038
C	1.223126	0.798705	-0.096972
H	-3.064826	0.853469	0.091143
H	-1.515739	2.518903	0.743361
H	-1.943912	0.968553	-1.275389
H	-1.003050	1.091185	1.635824
O	2.283907	1.105373	-0.644316
N	0.230671	1.708610	0.044777
H	0.435246	2.613298	-0.360713
S	-1.809515	-1.118902	-0.053957
H	0.091384	-1.030302	-1.550362
H	0.542749	-0.556518	1.421012

C	2.268787	-1.409818	0.457582
H	3.023200	-0.888615	1.050839
H	2.086401	-2.390381	0.904514
H	2.670222	-1.552966	-0.549254
H	0.098114	-2.402902	-0.445471

D_{iso}

C	0.481366	-1.321965	-0.314200
C	1.316752	1.264682	0.567997
C	-0.729231	-0.733469	-1.065916
C	0.123434	1.934901	-0.111225
C	-1.627314	0.122160	-0.190004
H	2.168825	1.949741	0.560981
H	0.061056	2.970521	0.229238
H	-1.357687	-1.557495	-1.411779
H	1.078860	1.040203	1.611802
H	0.269877	1.957997	-1.196386
O	-2.739215	-0.278512	0.163750
N	-1.159472	1.324919	0.213319
H	-1.780540	1.834340	0.829296
S	1.941746	-0.216466	-0.284490
C	0.107808	-1.849526	1.069334
H	-0.733630	-2.545786	0.989342
H	0.952431	-2.371783	1.522924
H	-0.194655	-1.037731	1.739170
H	-0.392571	-0.169717	-1.941574
H	0.853223	-2.153872	-0.920318

TS4

C	-1.457324	-0.285200	0.666876
C	-0.606452	-0.330630	-0.525359
C	-0.410548	1.110706	-0.297622
O	-0.951594	2.027192	-0.997001
H	-1.186521	-0.484850	-1.437115
S	0.913731	-1.482830	-0.706259
C	1.832920	-0.695658	0.654877
H	1.338908	-0.921917	1.606421
H	2.832457	-1.135191	0.670477
C	1.925354	0.813900	0.465465
H	2.385108	1.017264	-0.515410
H	2.584368	1.228098	1.235714
H	0.701332	2.473257	0.630544
N	0.620168	1.462355	0.617621
H	-0.958177	-0.254922	1.630400
C	-2.844112	-0.873740	0.601675
H	-2.853438	-1.969444	0.455976
H	-3.413514	-0.667927	1.514590
H	-3.417676	-0.449374	-0.234254

TS4'

C	1.176800	1.422110	0.051378
C	0.453787	0.536724	-0.890662
C	1.134551	-0.622170	-0.278218
O	2.130112	-1.210664	-0.801216
H	0.804293	0.652160	-1.917933
S	-1.435931	0.495942	-1.035487
C	-1.781680	-0.331378	0.553889
H	-1.659934	0.372511	1.379885
H	-2.826540	-0.648438	0.518131
C	-0.889296	-1.546510	0.763917
H	-1.071336	-2.265178	-0.051541
H	-1.170928	-2.030806	1.704476
H	1.091172	-1.969127	1.172318
N	0.527345	-1.182908	0.866337
H	1.801150	2.171896	-0.438242
C	0.575800	1.848648	1.361472
H	1.248357	2.543830	1.877170
H	-0.398323	2.364695	1.264257
H	0.404909	0.994509	2.033979

$TS4_{iso}$

C	-0.999799	-0.097087	1.561389
C	-0.739519	-0.504761	0.158943
C	-0.734157	0.964130	-0.055001
O	-1.664162	1.613582	-0.634096
S	0.791155	-1.501638	-0.303489
C	2.018991	-0.286994	0.260996
H	2.003834	-0.227164	1.354757
H	2.999220	-0.650750	-0.054962
C	1.735973	1.080988	-0.342596
H	1.721830	0.984139	-1.440787
H	2.548298	1.765903	-0.077218
H	0.369854	2.609090	-0.106130
N	0.497653	1.647781	0.190546
H	-0.164138	0.188100	2.190051
C	-1.916009	-1.215413	-0.485239
H	-1.827826	-1.227756	-1.575897
H	-1.968607	-2.247335	-0.121727
H	-2.843683	-0.704561	-0.219076
H	-1.860421	-0.551754	2.054805

(E)-Crotonamide

C	-3.195928	-0.126680	-0.007110
C	-1.891630	-0.406426	0.048966
C	-0.863653	0.669015	-0.056305
O	-1.158760	1.866097	-0.181964
H	-1.544808	-1.430775	0.173407
S	3.160790	-1.210948	-0.186191
C	2.788063	0.449353	0.512428
H	2.621492	0.368215	1.592784
H	3.639156	1.121061	0.365505
C	1.563771	1.110620	-0.115986
H	1.758245	1.329692	-1.175109
H	1.330464	2.055260	0.385435
H	0.621845	-0.755434	0.005228
N	0.410277	0.238422	0.007058
H	-3.482715	0.916989	-0.132240
C	-4.292821	-1.138383	0.089641
H	-4.948835	-0.911060	0.936372
H	-4.917877	-1.111986	-0.808984
H	-3.898287	-2.149524	0.213155

(Z)-Crotonamide

C	3.106150	-1.039999	0.131219
C	1.776032	-0.937811	0.257739
C	0.961654	0.297038	0.063511
O	1.442807	1.430219	-0.078646
H	1.215644	-1.833453	0.516154

S	-3.308777	-0.906151	-0.238569
C	-2.703094	0.694845	0.434118
H	-3.422452	1.491094	0.220156
H	-2.608576	0.627182	1.524096
C	-1.354795	1.123003	-0.140620
H	-0.995500	2.034986	0.346760
H	-1.454381	1.332304	-1.214988
H	-0.741793	-0.865595	0.078683
N	-0.369538	0.079665	0.073588
C	4.102948	0.017652	-0.231149
H	3.625633	0.948824	-0.527060
H	4.759160	0.211487	0.625421
H	4.745941	-0.347555	-1.038406
H	3.527699	-2.028628	0.311432

Methacrylamide

C	3.491776	0.222211	-0.009779
C	2.187360	0.504640	0.027274
C	1.217093	-0.650835	-0.052972
O	1.600692	-1.824844	-0.144258
S	-2.901615	0.930098	-0.221241
C	-2.436500	-0.695263	0.504763
H	-2.288164	-0.588163	1.585422
H	-3.242512	-1.420357	0.358319
C	-1.164549	-1.285233	-0.100983
H	-1.336247	-1.551397	-1.153077
H	-0.861301	-2.192417	0.430792
H	-0.388860	0.648408	-0.033654
N	-0.084405	-0.320289	-0.005460
H	3.828942	-0.805286	-0.095983
H	4.238376	1.008350	0.045430
C	1.655512	1.909253	0.143912
H	1.036911	2.177889	-0.720018
H	1.040041	2.031169	1.041903
H	2.479796	2.622370	0.199712

MT

C	0.000000	0.000000	-1.130939
H	-0.000000	1.019044	-1.528954
H	0.882518	-0.509522	-1.528954
H	-0.882518	-0.509522	-1.528954
S	0.000000	0.000000	0.710781

TS1' A

C	1.278244	0.441555	-0.209496
C	0.454076	-0.264543	-1.075776
C	1.157182	-0.919651	-0.018397
O	1.410153	-1.976169	0.585337
C	1.807304	1.790566	0.113958
H	0.972631	2.474340	0.304442
H	2.447415	1.767358	0.997416
H	2.378458	2.196626	-0.726489
H	0.196154	-0.309630	-2.124557
S	-1.944072	-0.030016	-0.510294
C	-1.622804	0.399288	1.228182
H	-2.053159	1.369555	1.489386
H	-0.537017	0.460777	1.398840
H	-2.024575	-0.352702	1.912142

TS1' B

C	1.103387	-0.375331	1.223091
C	0.830564	0.550921	0.225096
C	1.324401	-0.756137	-0.085432
O	1.673798	-1.580262	-0.942439
S	-1.600000	0.511848	-0.407920
C	-1.960478	-1.070926	0.410992
H	-2.785658	-0.978867	1.121758
H	-1.072654	-1.399874	0.974155
C	1.018794	2.005485	-0.050943
H	0.673462	2.262224	-1.053495
H	0.480337	2.613064	0.678855
H	2.088502	2.234099	0.024507
H	1.136630	-0.546323	2.291278
H	-2.211006	-1.855877	-0.307661

CP'⁻ A

C	-1.042207	0.706341	0.077048
C	-0.132025	-0.317360	0.663097
C	-1.499208	-0.535948	0.052103
O	-2.334777	-1.455247	-0.198577
C	-1.119198	2.172372	-0.166692
H	-0.415196	2.498347	-0.942535
H	-2.122985	2.460750	-0.492811
H	-0.884445	2.740384	0.741910
H	0.048093	-0.392673	1.744820
S	1.391576	-0.878650	-0.220482
C	2.432219	0.594995	0.019411
H	2.570055	0.792956	1.085191
H	1.972140	1.464880	-0.454114
H	3.407835	0.413326	-0.435930

CP'⁻ B

C	0.935230	-0.340053	1.255652
C	0.432073	0.529988	0.169688
C	1.236165	-0.722348	0.010518
O	1.757319	-1.487246	-0.849043
S	-1.405013	0.530423	-0.300176
C	-1.862022	-1.155519	0.178433
H	-2.931125	-1.274172	-0.005875
H	-1.656795	-1.318959	1.238102
C	0.943079	1.933621	-0.115134
H	0.733613	2.241621	-1.146669
H	0.483282	2.670174	0.555456
H	2.026526	1.962415	0.038505
H	0.969464	-0.451826	2.331295
H	-1.310466	-1.892187	-0.410601

CP' A_{anti}

C	-1.224469	-0.282401	0.521447
C	-0.151297	-0.407504	-0.612206
C	-0.749551	0.880302	-0.236852
O	-0.732221	2.076906	-0.368934
H	-0.530614	-0.868367	-1.523636
S	1.574731	-0.825957	-0.341933
C	2.080328	0.502132	0.789981
H	3.137287	0.333631	0.999951
H	1.528358	0.459733	1.730409
H	1.958861	1.480042	0.321682

H	-0.837169	-0.332937	1.541246
C	-2.588230	-0.906034	0.304641
H	-2.554767	-1.973047	0.537629
H	-3.332792	-0.430386	0.947145
H	-2.907776	-0.787574	-0.734082

CP' A_{syn}

C	1.579125	-0.175577	0.499044
C	0.050516	-0.033901	0.871004
C	0.807105	1.018142	0.175964
O	0.732876	2.056444	-0.431037
H	-0.141888	0.063146	1.937067
S	-1.189242	-0.924216	-0.070828
C	-2.439264	0.388708	-0.213108
H	-3.267334	-0.023764	-0.791407
H	-2.023640	1.250709	-0.736166
H	-2.800359	0.682956	0.773335
H	2.196205	-0.109119	1.393593
C	2.090317	-1.111975	-0.576463
H	3.096960	-0.813215	-0.877782
H	2.130986	-2.136542	-0.197546
H	1.447147	-1.090646	-1.458206

CP' B

C	0.847136	0.018926	1.364124
C	0.263437	0.376498	-0.052340
C	1.538485	-0.333037	0.124167
O	2.492199	-0.837268	-0.407515
S	-1.091377	-0.657490	-0.630099
C	-2.384293	-0.122524	0.526882
H	-3.266674	-0.728923	0.317414
H	-2.630741	0.929812	0.380824
C	0.257305	1.832899	-0.473665
H	0.253068	1.911748	-1.563025
H	-0.629880	2.336147	-0.078675
H	1.141563	2.349749	-0.089629
H	-2.066509	-0.296356	1.557003
H	0.358940	-0.770224	1.930825
H	1.232247	0.849459	1.951956

MA

C	-0.708121	0.000000	0.017287
H	-1.118455	-0.881672	-0.480981
H	-1.061351	-0.000007	1.056829
H	-1.118453	0.881679	-0.480969
N	0.751111	-0.000000	-0.125811
H	1.144607	0.811873	0.341037
H	1.144607	-0.811873	0.341037

TS1'$_N$ A

C	1.034503	-0.363758	0.122030
C	0.087819	0.104845	1.034380
C	0.710405	0.966064	0.064024
O	0.820538	2.121397	-0.387610
C	1.829064	-1.553312	-0.283519
H	2.146511	-2.123929	0.593450
H	1.231547	-2.218459	-0.915638
H	2.712473	-1.254643	-0.851123
H	-0.113620	0.024858	2.096544
C	-1.927356	-0.360627	-0.903230
H	-2.912815	-0.695624	-1.235354
H	-1.167660	-1.040214	-1.297493
H	-1.732343	0.636615	-1.301972
N	-1.809719	-0.319289	0.553607
H	-2.052281	-1.213380	0.974020
H	-2.414693	0.389342	0.961088

TS1'$_N$ B

C	0.760714	-0.183064	1.254778
C	0.385100	0.604831	0.160530
C	1.129780	-0.624626	0.013471
O	1.688523	-1.420638	-0.760736
C	-1.957512	-1.006146	0.135548
H	-3.013340	-1.195271	-0.071391
H	-1.794505	-1.016165	1.215964
C	0.451357	2.050876	-0.220293
H	0.159593	2.208708	-1.260827
H	-0.185084	2.659793	0.427395
H	1.484677	2.388096	-0.097444
H	0.772079	-0.234380	2.334984
H	-1.355451	-1.801364	-0.308477
N	-1.508331	0.280233	-0.393837
H	-2.088030	1.043650	-0.051244
H	-1.546439	0.299188	-1.410417

6 Kinetic study for the reaction of cyclopropenone probe 1 with cysteine

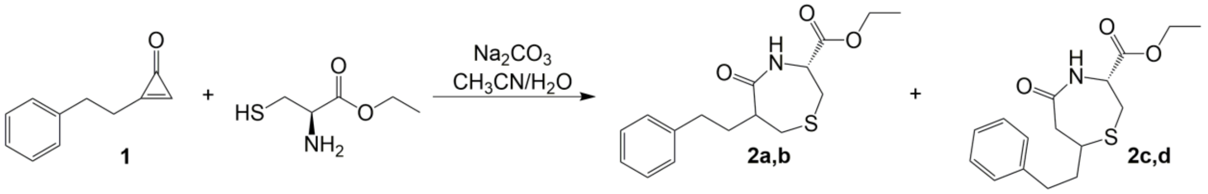

2-phenethylcycloprop-2-en-1-one **1** in CH_3CN (160 µL of 25 mM solution), L-cysteine ethyl ester hydrochloride in H_2O (40 µL of 0.1 M solution) and the internal standard 2-phenylacetamide in CH_3CN (200 µL of 15 mM solution) were added to CH_3CN (640 µL) and H_2O (936 µL) at 4 °C (in a cold room). The reaction was then initiated by adding Na_2CO_3 in H_2O (24 µL of 0.25 M solution) affording the starting reactant concentrations of 1.5 mM (2-phenylacetamide), 2 mM (for cysteine and cyclopropenone **1**) and 3 mM (for Na_2CO_3), and the reaction mixture was stirred at 4 °C. 125 µL of reactant mixture was quenched at different time points with 1 µL of 1M HCl solution. The sample was then analysed by LC–MS as described earlier (page S3). The concentration of 2-phenethylcycloprop-2-en-1-one **1** was then determined at each time point and used to calculate the rate constant as shown in **Figure S6**. Applying the similar kinetic analysis reported for the CBT-cysteine reaction,[9] the resulted 1/[concentration] values for cyclopropenone **1** were plotted against the time and fitted to a linear equation to obtain the second order constant *k* from the slope. The experiment was repeated three times. Data were processed using GraphPad Prism (GraphPad Software, La Jolla, CA, USA).

In a separate experiment, LC–MS analysis was used to confirm that the reaction did not proceed after the quenching with 1 µL of 1M HCl solution, and that the reaction did not start before the initiation with the addition of Na_2CO_3.

a

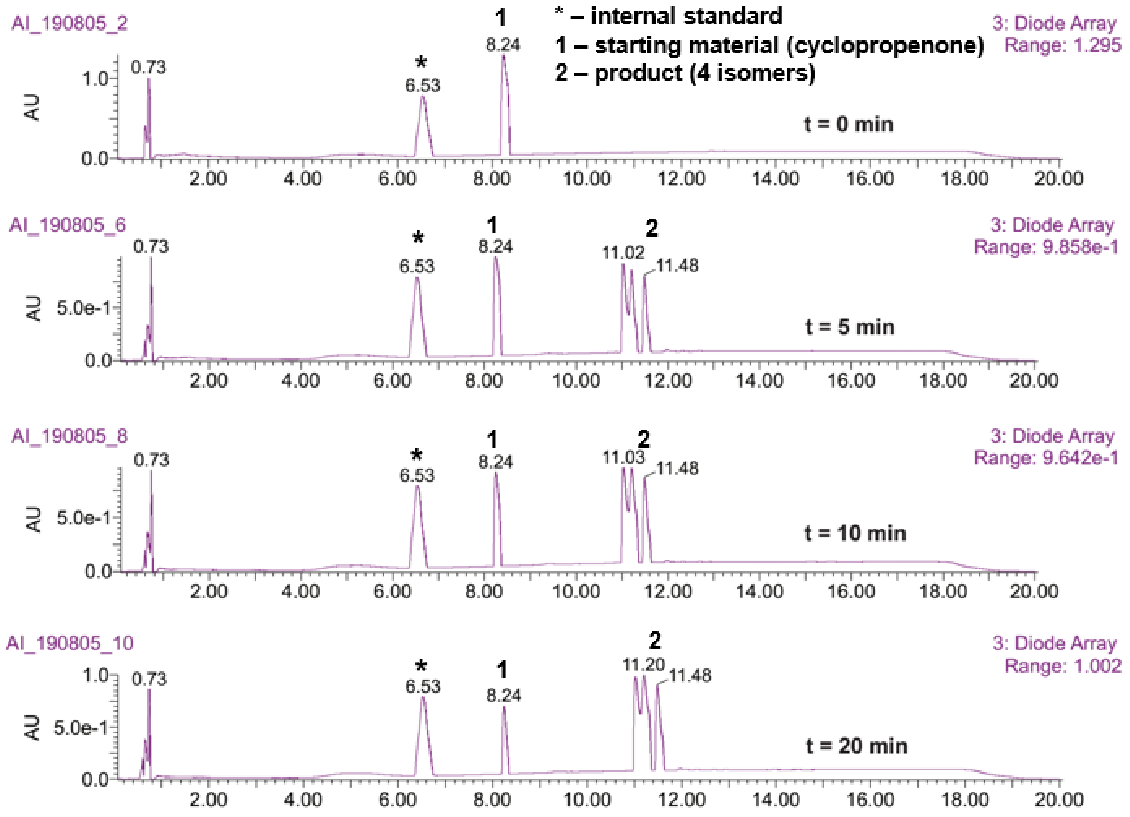

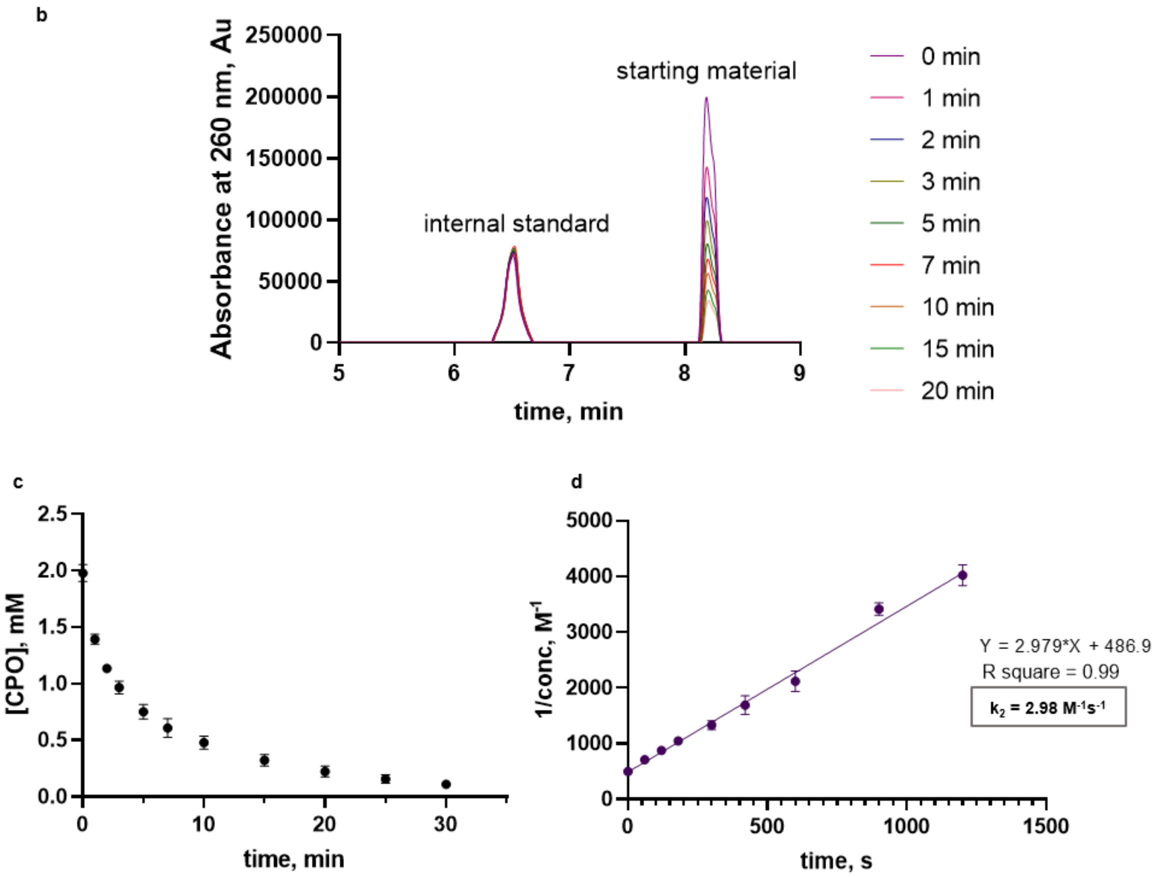

Figure S6 a) Typical chromatograms of the reaction mixture at different time points. b) Ratio of cyclopropenone 1 peak area to the peak area of the internal standard was uses to determine cyclopropenone concentration at different time points. c) Concentration of cyclopropenone **1** vs time for reaction of probe **1** (2 mM) with L-cysteine ethyl ester (2 mM) in CH_3CN/H_2O at 4 ºC. Error bars indicate the standard deviations of three replicate experiments. d) Linear regression analysis of 1/[cyclopropenone] vs. time of the cyclopropenone **1** reaction with cysteine. Error bars indicate the standard deviations of three replicate experiments.

6.1 Stability studies of cyclopropenone probe 1 in buffer

To determine stability in aqueous buffer, cyclopropenone probe **1** (5 mM) was dissolved in 50% CD_3CN/NaP_i buffer (50 mM, pH 7 or 8), incubated at 37 ºC, and monitored by ^1H NMR for one week.

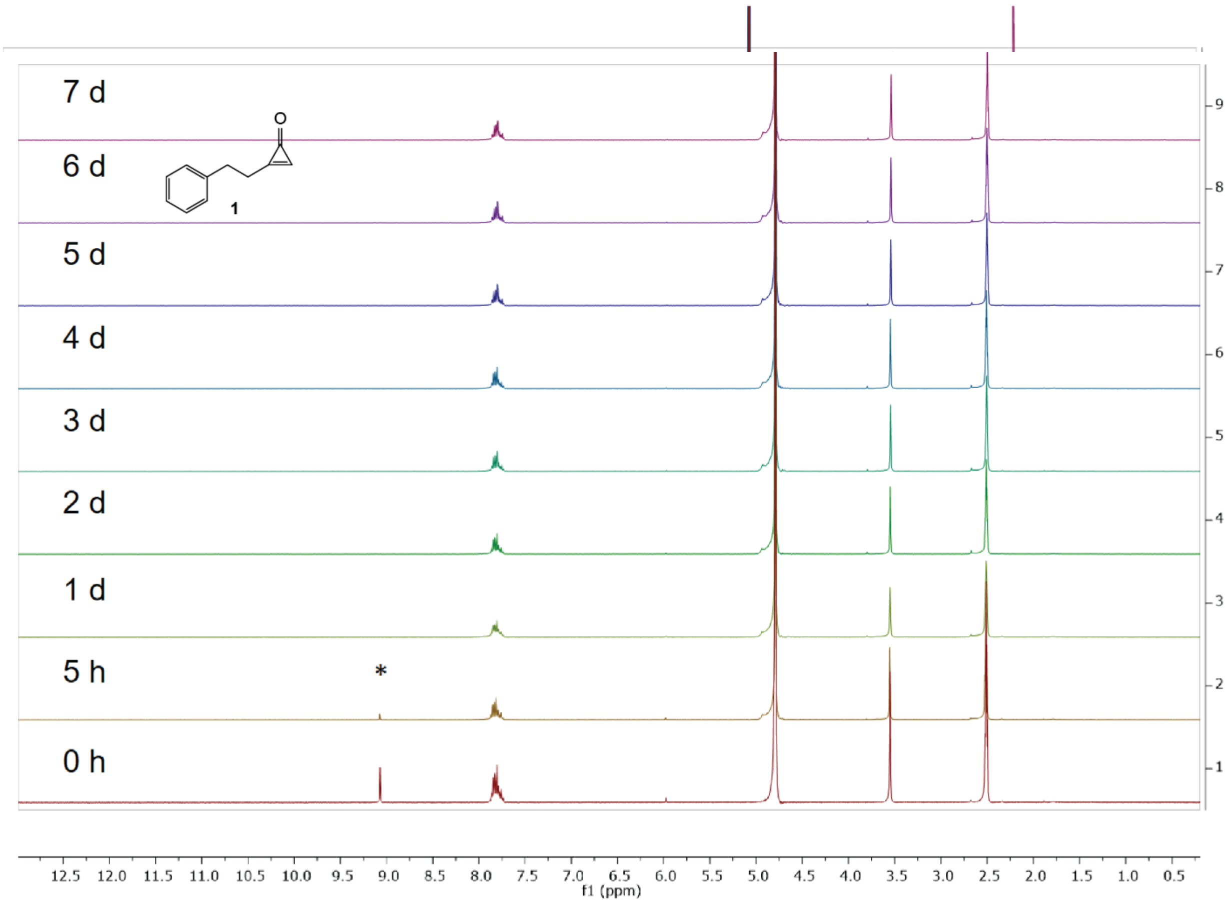

Figure S7 Cyclopropenone **1** is stable in 50% CD_3CN/NaP_i buffer (50 mM, pH 7) at 37 ºC for at least 7 days. *H/D exchange was observed for cyclopropenone-ring proton.

7 Reaction and stability of cyclopropenones with nucleophiles

7.1 Selectivity studies and competition experiments. General methods

Preparation of stock solutions

To determine stability of cyclopropenones towards biological nucleophiles, cyclopropenone probe **1** was incubated with various nucleophiles under conditions used for the reaction with cysteine. To this end, stock solutions of L-cysteine ethyl ester hydrochloride (100 mM in H_2O), L-lysine ethyl ester hydrochloride (100 mM in H_2O), L-threonine methyl ester hydrochloride (100 mM in H_2O), L-tyrosine methyl ester hydrochloride (100 mM in H_2O), L-serine ethyl ester hydrochloride (100 mM in H_2O), L-glutathione (100 mM in H_2O), *N*-(tert-butoxycarbonyl)-L-cysteine methyl ester (100 mM in CH_3CN), cyclopropenone **1** (50 mM in CH_3CN), and Na_2CO_3 (0.25 M in H_2O) were prepared.

Control experiment

Cyclopropenone **1** stock (10 µL, 0.50 µmol) was mixed with CH_3CN (52.5 µL), H_2O (61 µL), Na_2CO_3 stock solution (1.5 µL, 0.375 µmol), incubated at 4 ºC, and analysed by LC–MS after 30 min and 24 h. LC–MS analysis was performed as described on page S3.

In a separate experiment, L-cysteine ethyl ester hydrochloride stock solution (2.5 µL, 0.25 µmol, 1 equiv.) was mixed with CH_3CN (52.5 µL), H_2O (58.5 µL), cyclopropenone **1** (10 µL, 0.50 µmol, 2

equiv.), Na_2CO_3 (1.5 µL, 0.375 µmol, 1.5 equiv.), incubated at 4 ºC, and analysed by LC–MS after 1 h and 24 h. LC–MS analysis was performed as described on page S3.

Stability of cyclopropenone 1 towards lysine, threonine, tyrosine and serine

Next, nucleophile stock solution (2.5 µL, 0.25 µmol, 1 equiv.) was mixed with CH_3CN (52.5 µL), H_2O (58.5 µL), cyclopropenone **1** (10 µL, 0.50 µmol, 2 equiv.), Na_2CO_3 (1.5 µL, 0.375 µmol, 1.5 equiv.), incubated at 4 ºC, and analysed by LC–MS after 30 min and 24 h. LC–MS analysis was performed as described on page S3.

For competition experiments, nucleophile stock solution (1.25 µL, 0.125 µmol, 1 equiv.) and L-cysteine ethyl ester hydrochloride stock solution (1.25 µL, 0.125 µmol, 1 equiv.) were mixed with CH_3CN (52.5 µL), H_2O (58.5 µL), cyclopropenone **1** (10 µL, 0.50 µmol, 4 equiv.), Na_2CO_3 (1.5 µL, 0.375 µmol, 3 equiv.), incubated at 4 °C, and analysed by LC–MS after 30 min. LC–MS analysis was performed as described on page S3.

Stability of cyclopropenone 1 towards thiol nucleophiles (glutathione, N-(tert-butoxycarbonyl)-protected cysteine)

Next, nucleophile stock solution (2.5 µL, 0.25 µmol, 1 equiv.) was mixed with CH_3CN (52.5 µL), H_2O (59.5 µL), cyclopropenone **1** (10 µL, 0.50 µmol, 2 equiv.), Na_2CO_3 (0.5 µL, 0.125 µL, 0.5 equiv.)*, incubated at 4 ºC, and analysed by LC–MS after 30 min and 24 h. LC–MS analysis was performed as described on page S3.

For competition experiments, nucleophile stock solution (1.25 µL, 0.125 µmol, 1 equiv.) and L-cysteine ethyl ester hydrochloride stock solution (1.25 µL, 0.125 µmol, 1 equiv.) were mixed with CH_3CN (52.5 µL), H_2O (59.5 µL), cyclopropenone **1** (5 µL, 0.25 µmol, 2 equiv.), Na_2CO_3 (1 µL, 0.250 µL, 2 equiv.),* incubated at 4 ºC, and analysed by LC–MS after 30 min. LC–MS analysis was performed as described on page S3.

*compared to the experiment above, 1 equiv. less of Na_2CO_3 was used to account for the neutralization of hydrochloride in stock solutions of L-cysteine, L-lysine, L-threonine, L-tyrosine and L-serine.

7.1.1 Control experiment

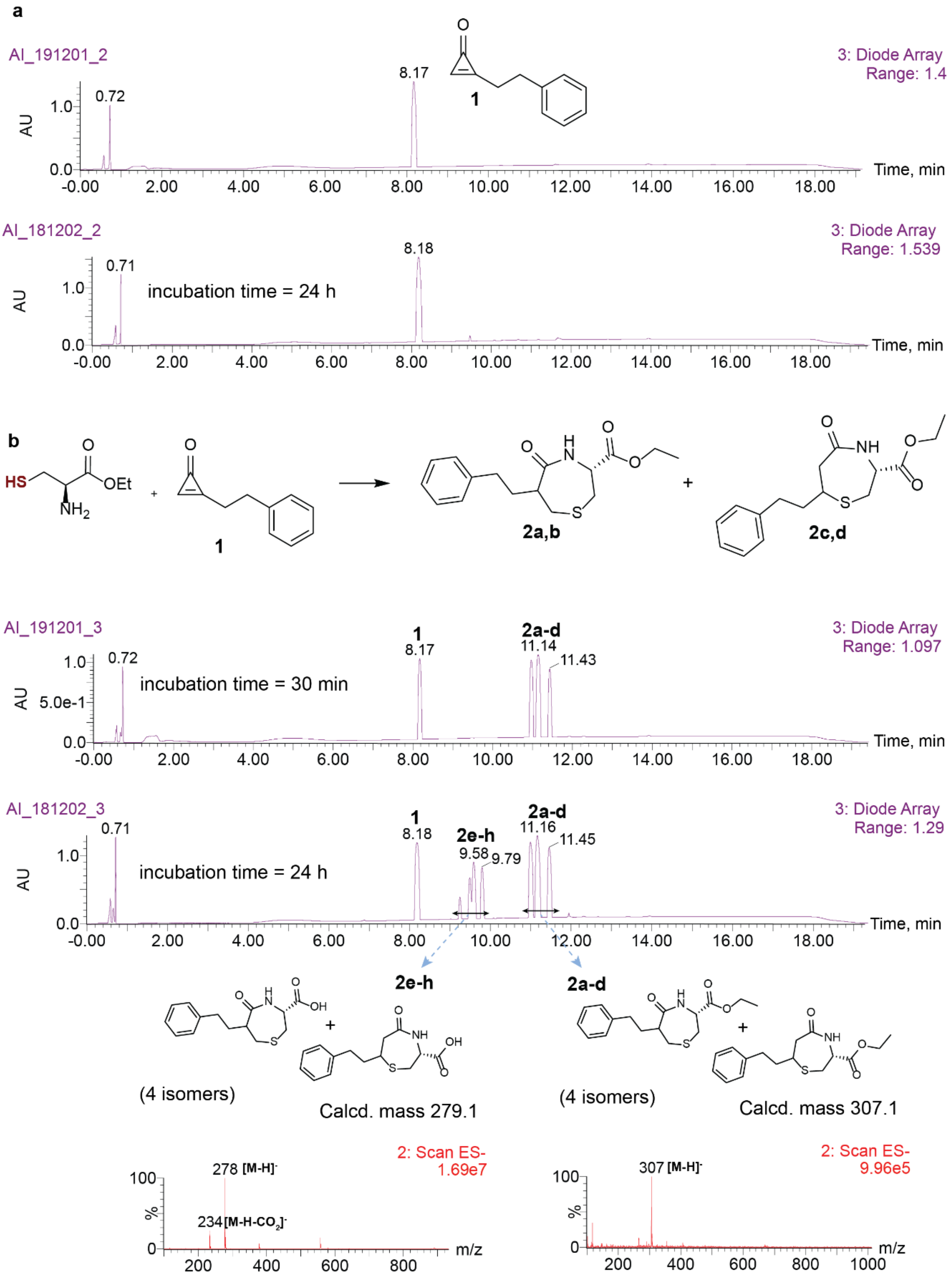

Figure S8 a. 2-Phenethylcycloprop-2-en-1-one **1** is stable under reaction conditions for at least 24 h. **b.** Cysteine reacts with 2-phenethylcycloprop-2-en-1-one forming products **2a–d** after incubation for 30 min. After 24 h, ester hydrolysis is observed (products **2e–h**) while the 1,4-thiazepan-5-one ring is stable.

7.1.2 Selectivity in the presence of lysine

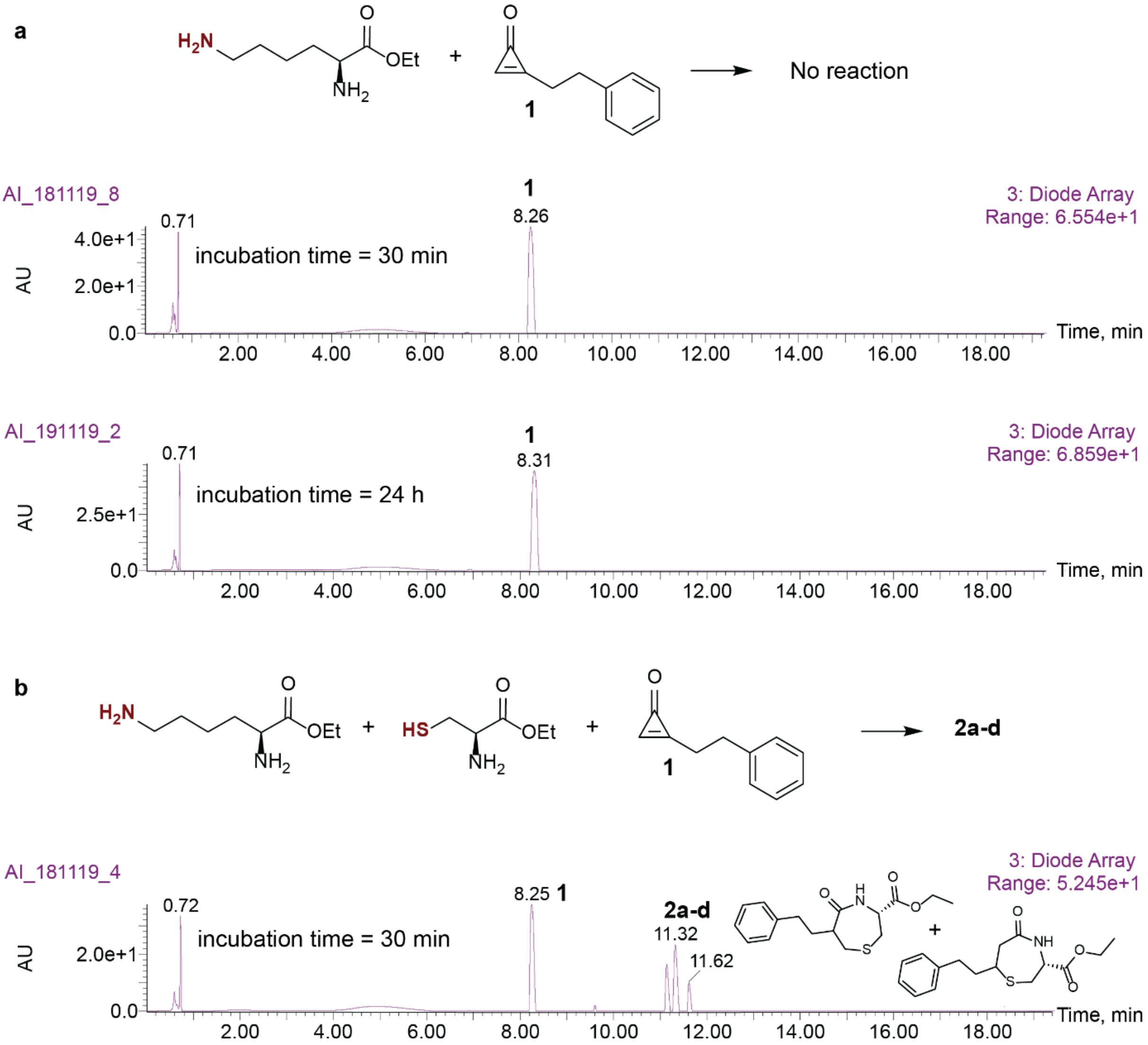

Figure S9 a. 2-Phenethylcycloprop-2-en-1-one **1** shows no reactivity with lysine after 30 min or 24 h incubation. **b.** Lysine does not interfere with the reaction between 2-phenethylcycloprop-2-en-1-one **1** and cysteine.

7.1.3 Selectivity in the presence of tyrosine

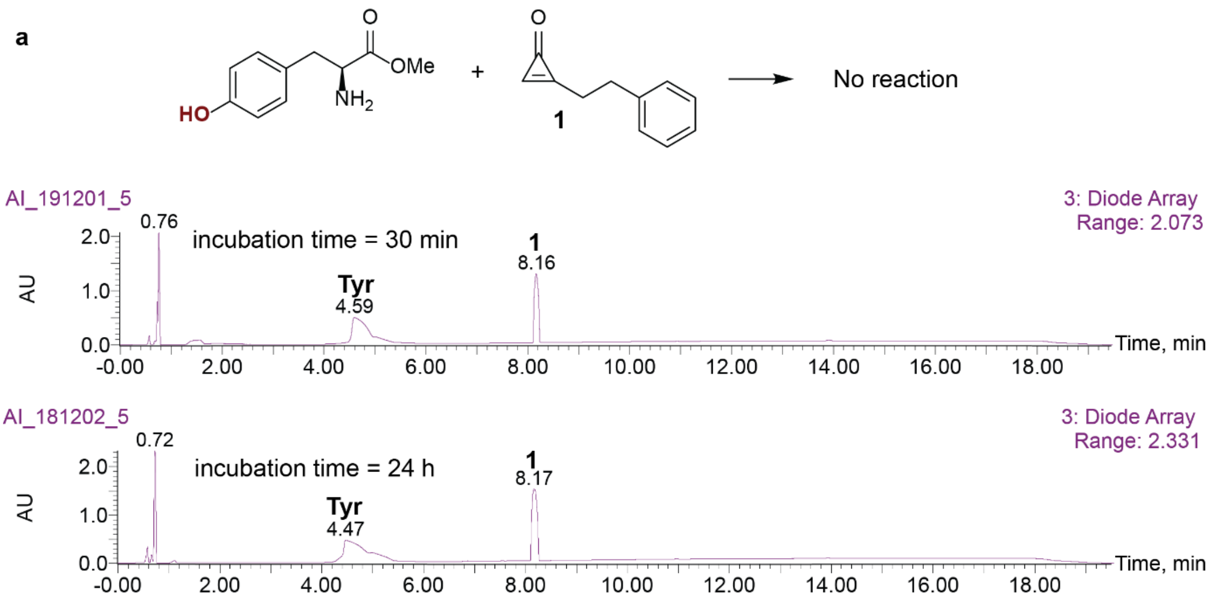

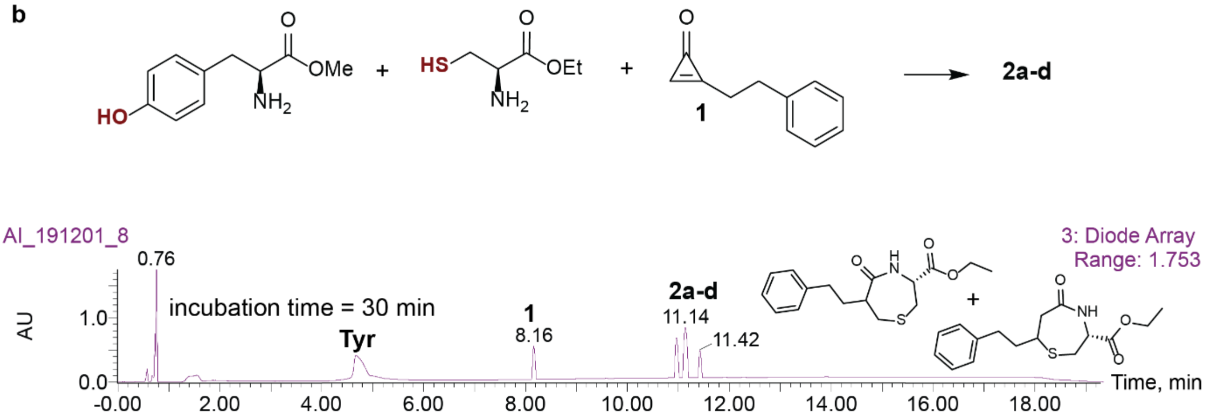

Figure S10 a. 2-Phenethylcycloprop-2-en-1-one **1** shows no reactivity with tyrosine after 30 min or 24 h incubation. **b.** Tyrosine does not interfere with the reaction between 2-phenethylcycloprop-2-en-1-one **1** and cysteine.

7.1.4 Selectivity in the presence of threonine

a

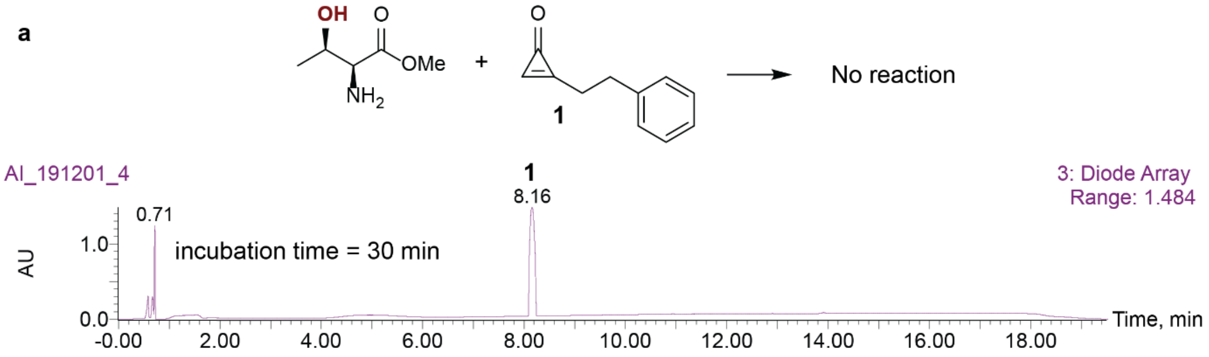

b

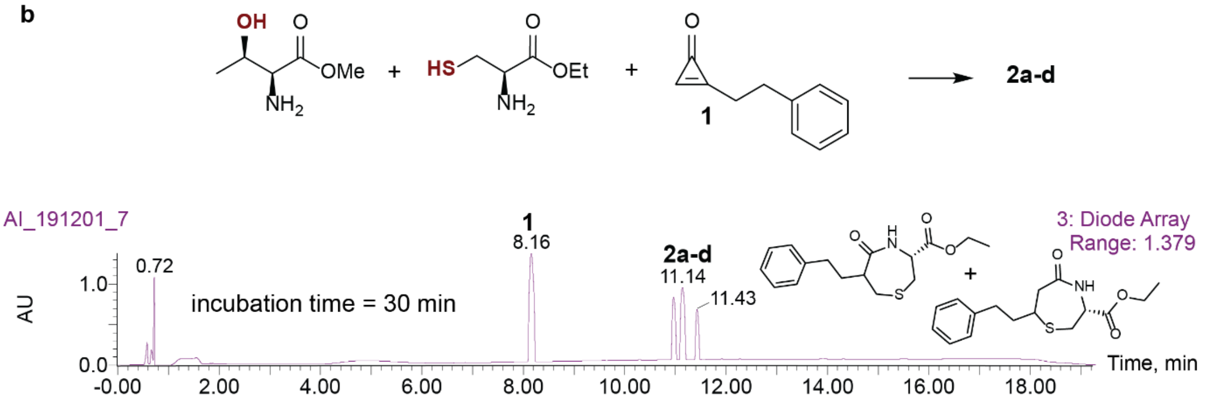

Figure S11 a. 2-Phenethylcycloprop-2-en-1-one **1** shows no reactivity with threonine after 30 min or 24 h incubation. **b.** Threonine does not interfere with the reaction between 2-phenethylcycloprop-2-en-1-one **1** and cysteine.

7.1.5 Selectivity in the presence of serine

a

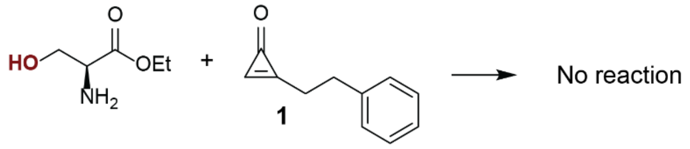

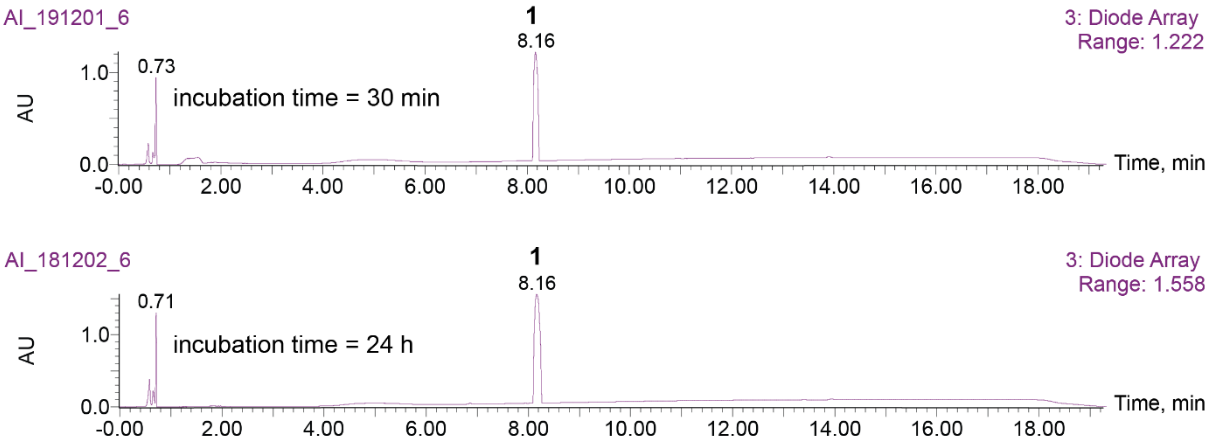

b

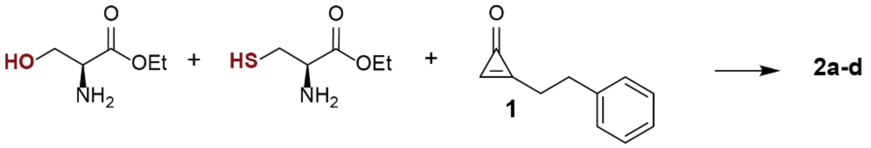

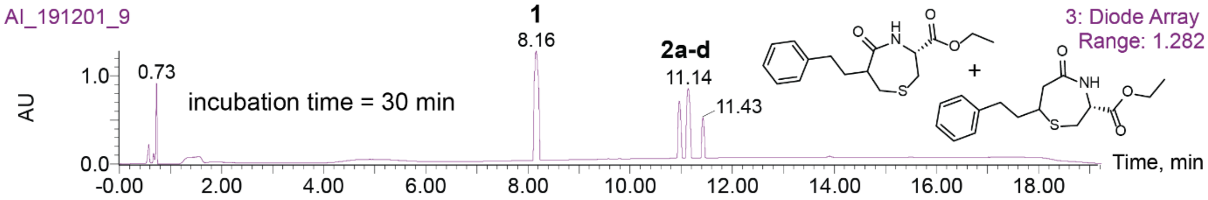

Figure S12 a. 2-Phenethylcycloprop-2-en-1-one **1** shows no reactivity with serine after 30 min or 24 h incubation. **b.** Serine does not interfere with the reaction between 2-phenethylcycloprop-2-en-1-one **1** and cysteine.

7.1.6 Selectivity in the presence of glutathione

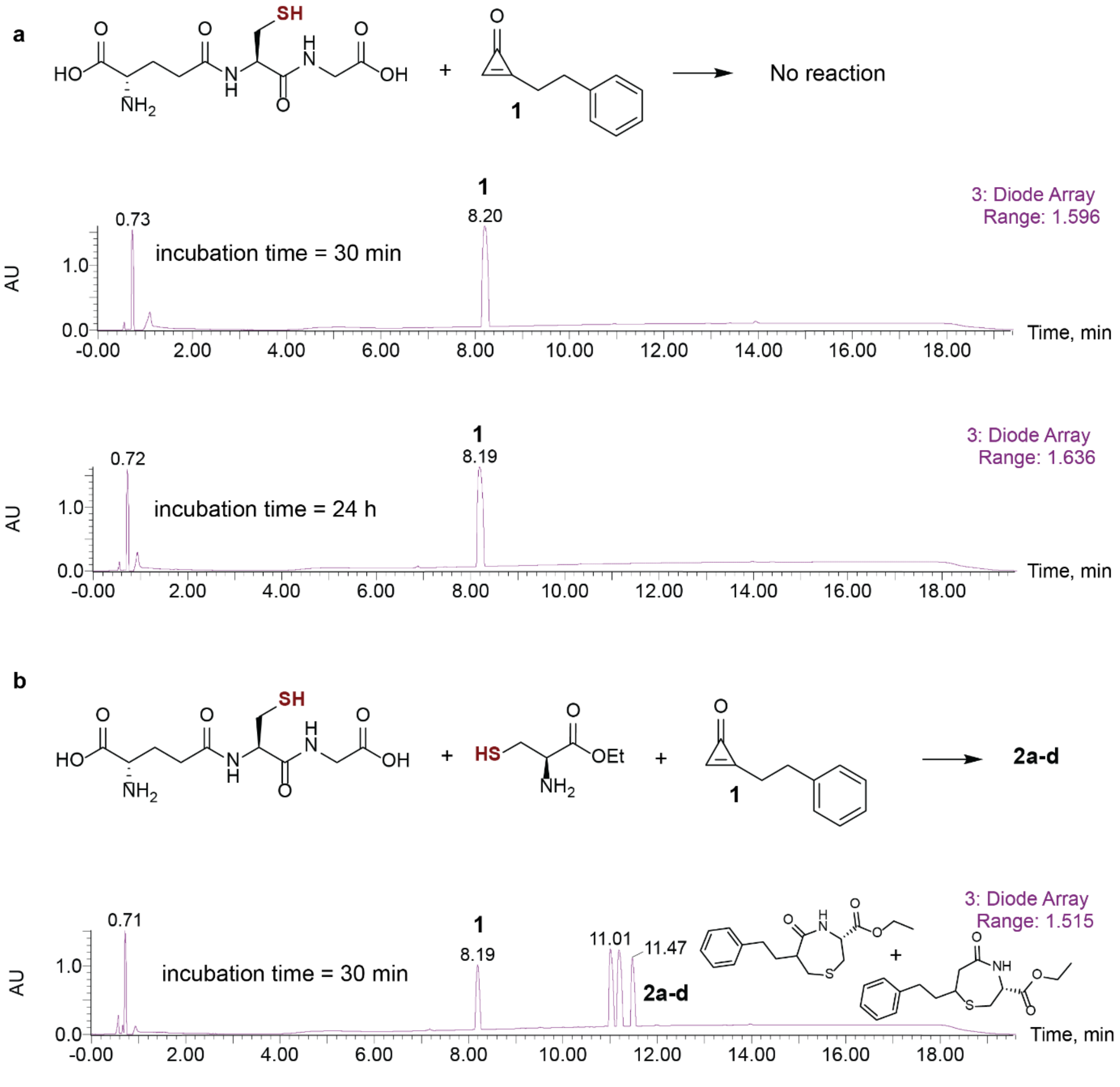

Figure S13 a. 2-Phenethylcycloprop-2-en-1-one **1** shows no reactivity with glutathione after 30 min or 24 h incubation. **b.** Glutathione does not interfere with the reaction between 2-phenethylcycloprop-2-en-1-one **1** and cysteine.

7.1.7 Selectivity in the presence of N-(tert-butoxycarbonyl)-protected cysteine

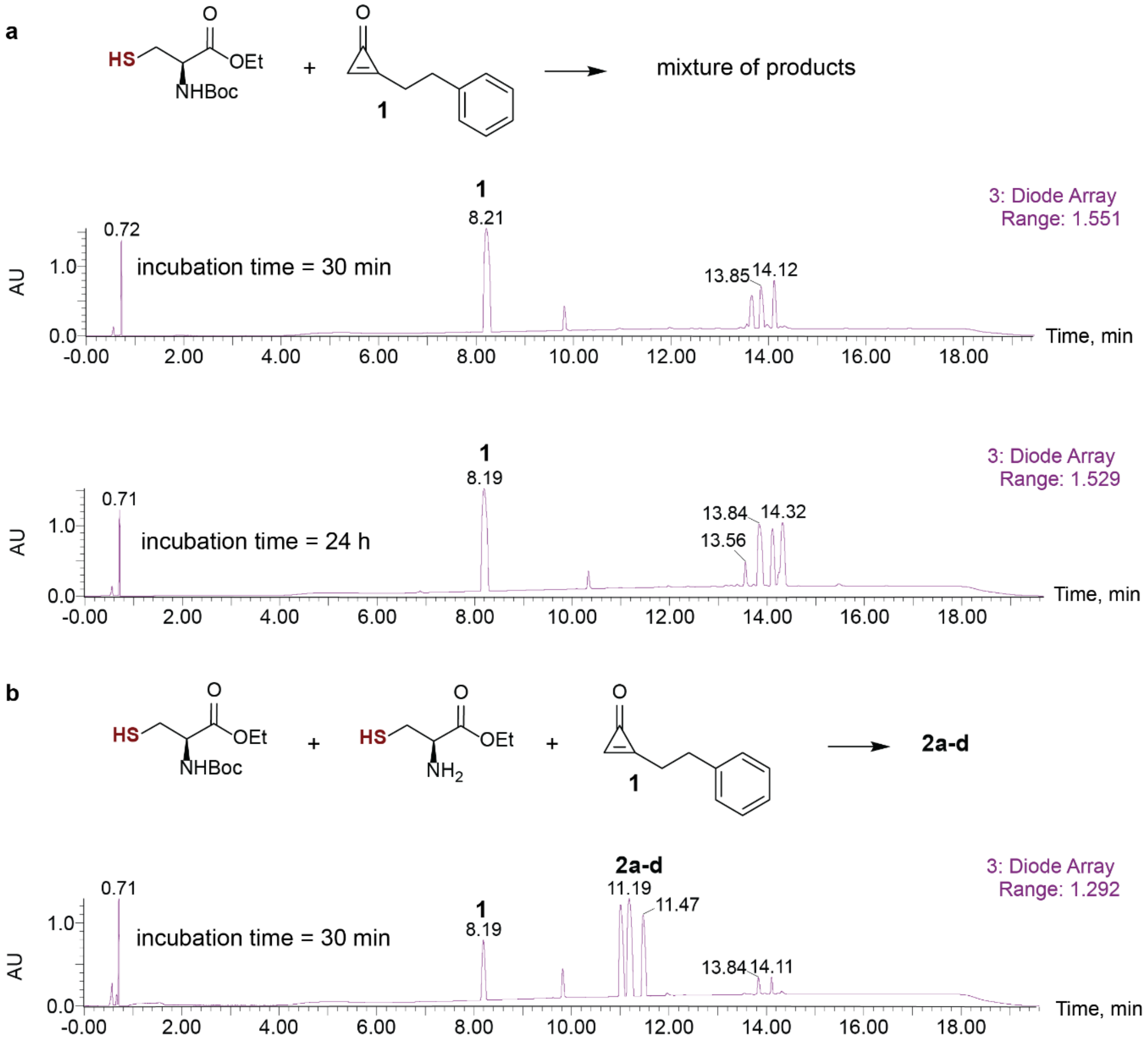

Figure S14 a. 2-Phenethylcycloprop-2-en-1-one **1** reacts with *N*-(tert-butoxycarbonyl)-protected cysteine forming a complex mixture of products. **b.** In the presence of *N*-(tert-butoxycarbonyl)-protected cysteine, 2-phenethylcycloprop-2-en-1-one **1** predominantly reacts with cysteine with free amino group (competition experiment)**.**

8 Peptide modification

8.1 General procedures and characterization methods

LC–MS method for analysis of peptide conjugation

LC–MS was performed on a Waters SQ Detector 2 mass spectrometer coupled to an Acquity UPLC system using an Acquity UPLC BEH C18 column (130Å, 1.7 µm, 2.1 mm X 50 mm). Solvents A, water with 0.1% formic acid and B, 71% acetonitrile, 29% water and 0.075% formic acid were used as the mobile phase at a flow rate of 0.2 mL min^{-1}. The gradient was programmed as follows: 100% A for 2 min, then 100% A to 100% B in 9 min, then 100% B for 5 min, and after that 100% A for 4 min. The electrospray source was operated with a capillary voltage of 3.0 kV and a cone voltage of 30 V. Nitrogen was used as the desolvation gas at a total flow of 800 L h^{-1}. *m/z* values of positively and negatively charged ions were measured in the mass analyser, which was scanned between *m/z* 100–2000 for the generation of mass spectra. The major peak(s) were selected for integration and analysed using MassLynx software (v. 4.1 from Waters).

LC–MS/MS analysis of modified peptides

Peptide solutions were prepared to approximate concentrations of 1 pmol/uL in 50:50 acetonitrile:water + 0.1% formic acid. The solutions were pulled into a syringe and loaded into the mass spectrometer source by means of a syringe pump.

All experiments were performed using an LTQ Orbitrap Velos hybrid ion trap mass spectrometer (Thermo Scientific, Waltham, MA). *m/z* values of positively charged ions were measured in the Orbitrap mass analyser, set at a resolution of 30000 which was scanned between *m/z* 380–1500 for the generation of mass spectra. Ions were then selected manually for MS/MS using higher energy collisional dissociation (HCD, NCE:20%) in the HCD collision cell. Measurement of the resulting fragment ions was performed in the Orbitrap analyser, set at a resolution of 30000 and which was scanned between *m/z* 50-1500. The resulting MS/MS spectra were annotated manually.

Modification of peptides by cyclopropenone-based probes:

Typically, peptide (25 µL, 0.3–1 mM) in NaP_i buffer (pH 7, 20 mM) was incubated with CPO-probe (required amount of 15 mM stock solution in CH_3CN, 2 equiv.) at 4 °C for indicated time before being analysed by LC-MS as described above. For peptides that are poorly soluble in NaP_i buffer, CH_3CN (up to 50%) or DMF (up to 10%) were added to the reaction mixture.

Peptides used in this study:

Entry	Sequence	Chemical Formula	M_wt	Purchased from
**P1**	CAIAI-NH_2	$C_{21}H_{40}N_6O_5S$	488.6	Biomatik
**P2**	CAIKI-NH_2	$C_{24}H_{47}N_7O_5S$	545.7	Biomatik
**P3**	CDPGYIGSR (Laminin)	$C_{40}H_{62}N_{12}O_{14}S$	967.07	Kindly provided by Prof. Pedro M. P. Gois
**P4**	CGAGESGKSTIVKQMK (GTP-Binding Protein Fragment, G alpha)	$C_{66}H_{118}N_{20}O_{23}S_2$	1623.91	APExBIO Technology
**P5**	CYFQNCPRG-NH_2 (Disulfide bridge: 1-6; Vasopressin)	$C_{46}H_{64}N_{14}O_{13}S_2$	1085.22	Abcam

8.2 Reactions and characterization of peptide conjugates

Reaction of peptide **P1** with **CPO-BN** to give **P1-BNa** and **P1-BNb**

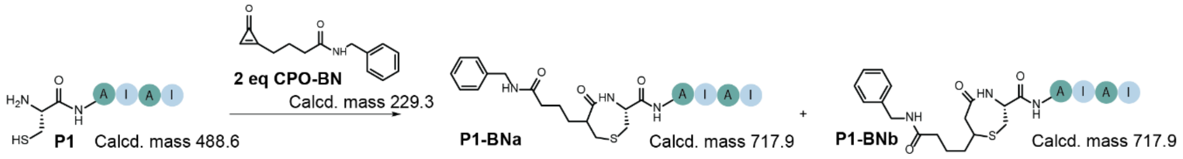

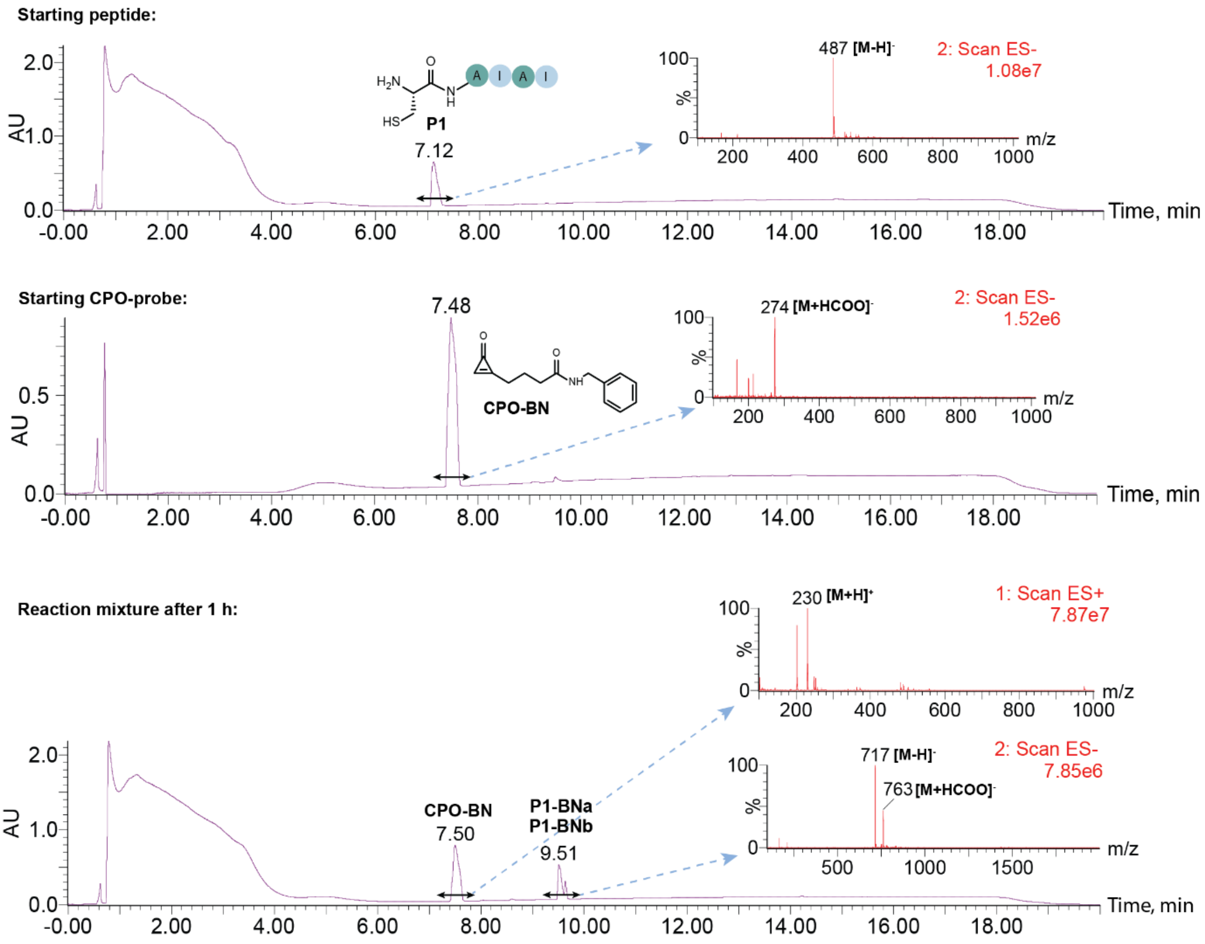

Figure S15. HPLC and MS analysis of reaction between peptide **P1** (15 µL of 0.5 mM stock solution in in NaP$_i$ buffer (pH 7, 20 mM)/DMF 9/1) and **CPO-BN** (10 µL of 1.5 mM stock solution in CH_3CN, 2 equiv.). The reaction was complete after incubation for 1 h at 4 °C.

Reaction of peptide **P1** with **CPO-PEG** to give **P1-PEGa** and **P1-PEGb**

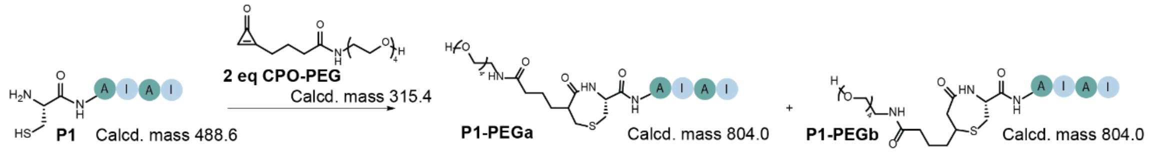

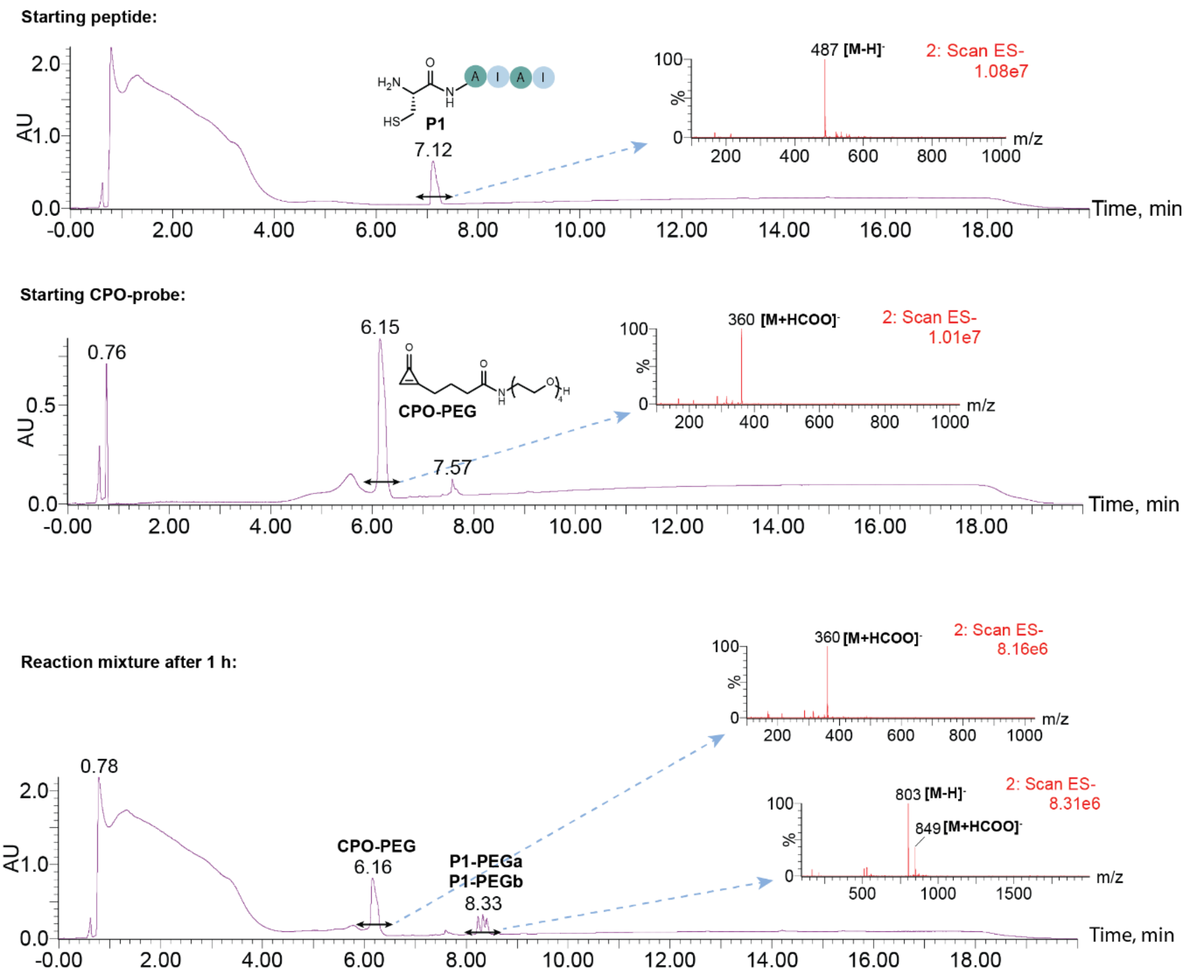

Figure S16. HPLC and MS analysis of reaction between peptide **P1** (15 µL of 0.5 mM stock solution in in NaP_i buffer (pH 7, 20 mM)/DMF 9/1) and **CPO-PEG** (10 µL of 1.5 mM stock solution in CH_3CN, 2 equiv.). The reaction was complete after incubation for 1 h at 4 ºC.

Reaction of peptide **P2** with **CPO-BN** to give **P2-BNa** and **P2-BNb**

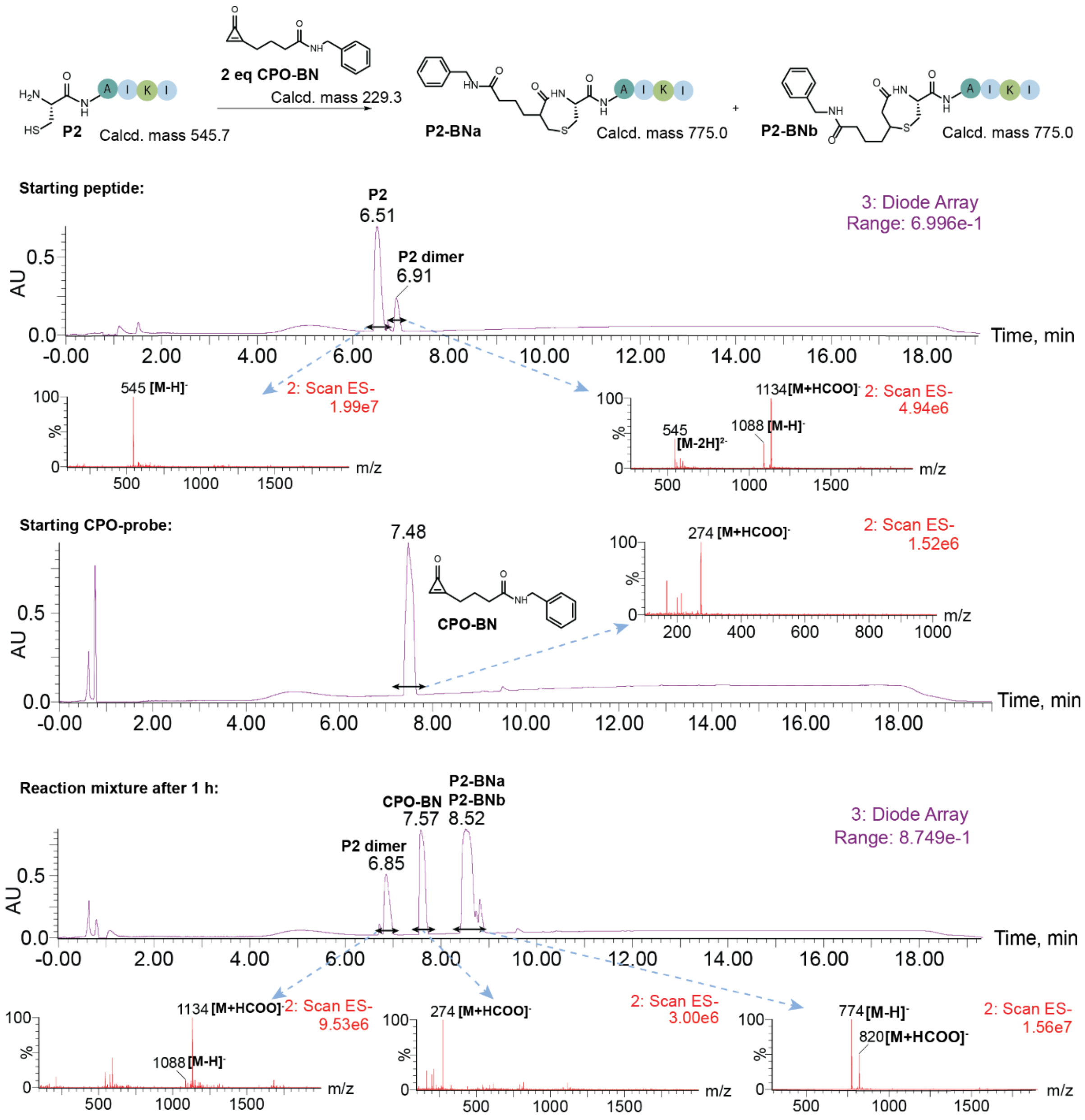

Figure S17. HPLC and MS analysis of reaction between peptide **P2** (20 µL of 0.5 mM stock solution in in NaP_i buffer (pH 7, 20 mM)) and **CPO-BN** (2.5 µL of 8 mM stock solution in CH_3CN, 2 equiv.). The reaction was complete after incubation for 1 h at 4 ºC.

Reaction of peptide **P2** with **CPO-PEG** to give **P2-PEGa** and **P2-PEGb**

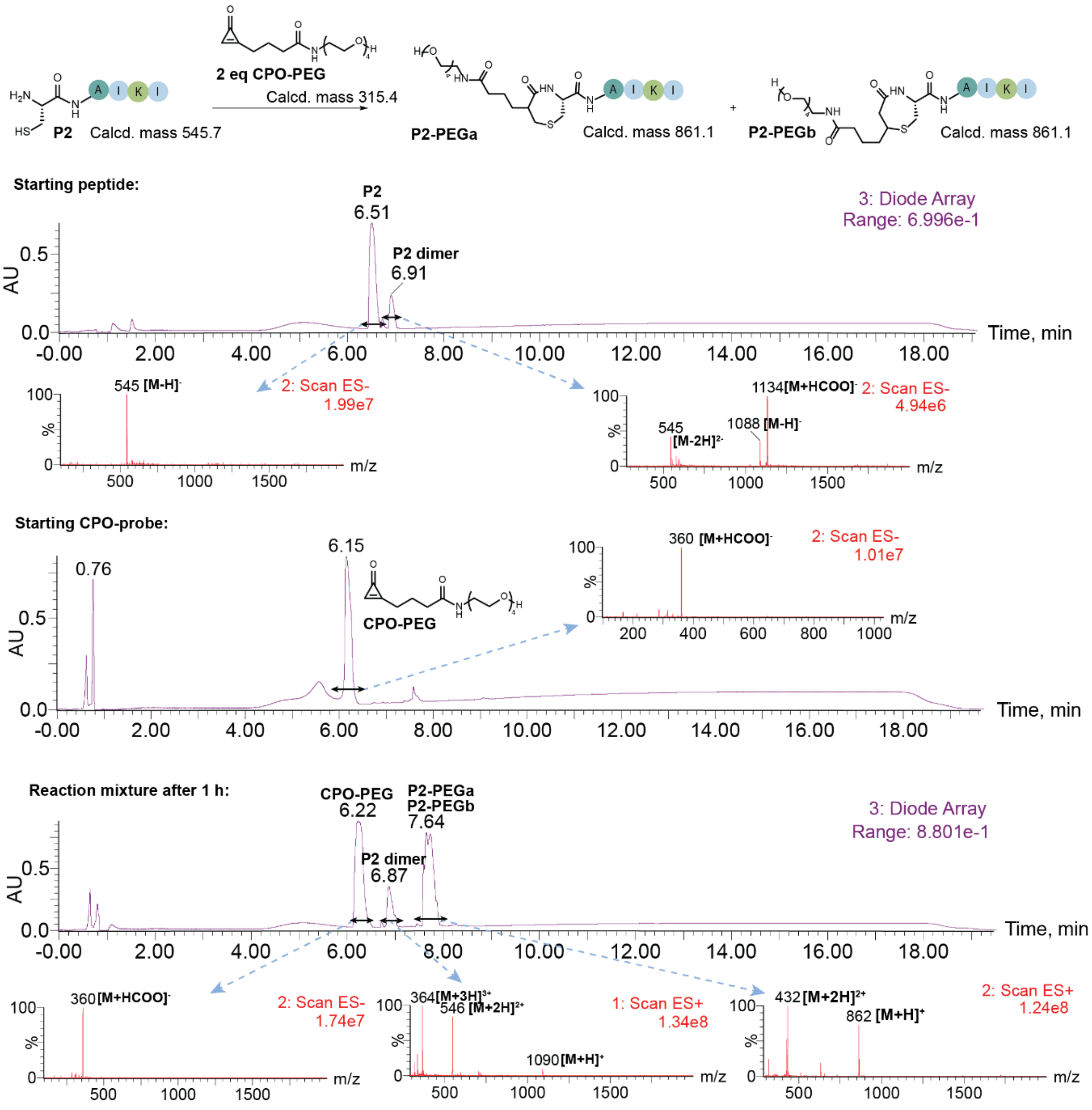

Figure S18. HPLC and MS analysis of reaction between peptide **P2** (20 µL of 0.5 mM stock solution in in NaP$_i$ buffer (pH 7, 20 mM)) and **CPO-PEG** (2.5 µL of 8 mM stock solution in CH_3CN, 2 equiv.). The reaction was complete after incubation for 1 h at 4 ºC.

Reaction of peptide **P3** with **CPO-BN** to give **P3-BNa** and **P3-BNb**

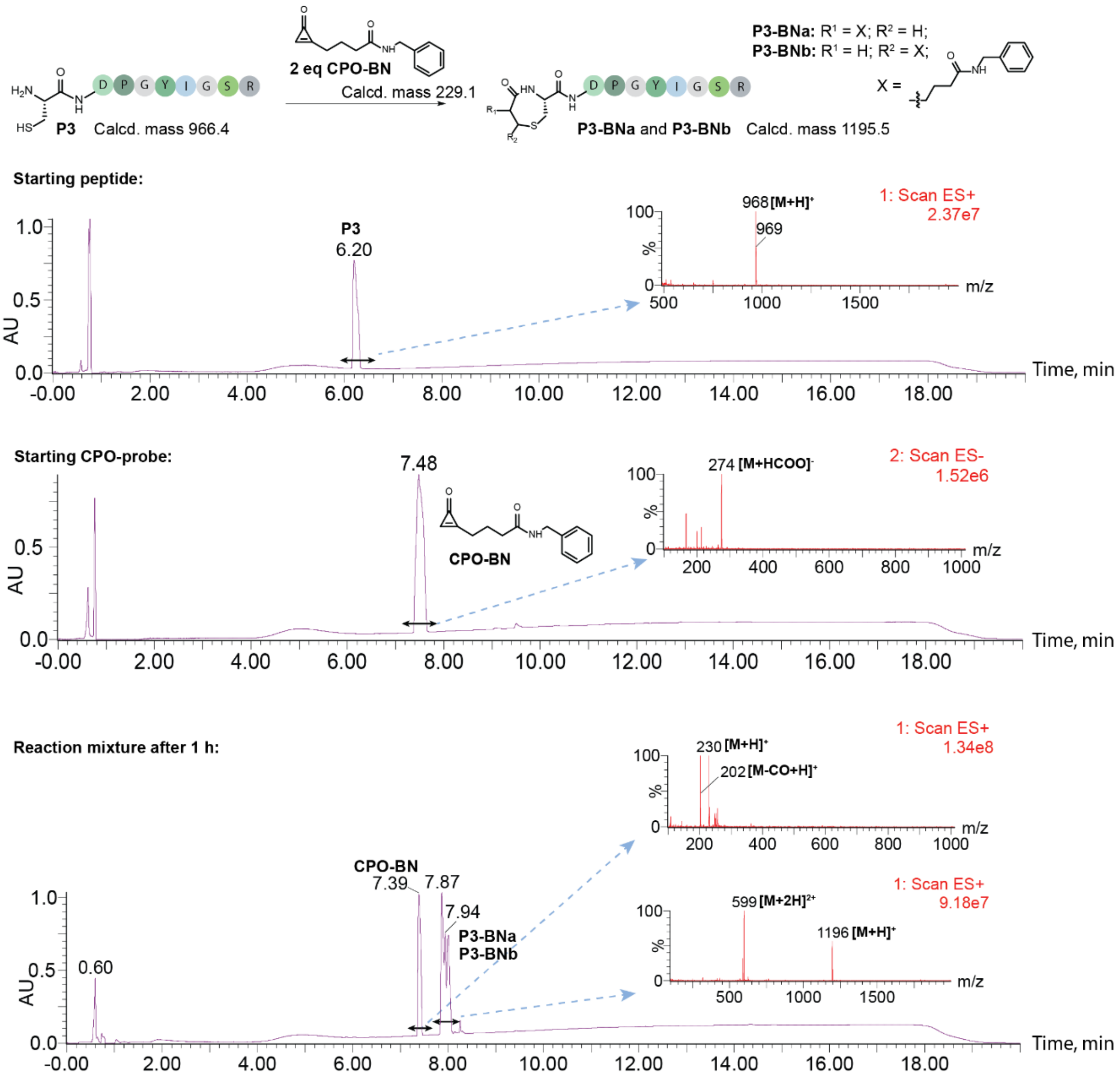

Figure S19. HPLC and MS analysis of reaction between peptide **P3** (25 µL of 0.4 mM stock solution in in NaP$_i$ buffer (pH 7, 20 mM)) and **CPO-BN** (2.5 µL of 8 mM stock solution in CH_3CN, 2 equiv.). The reaction was complete after incubation for 1 h at 4 ºC.

Reaction of peptide **P4** with **CPO-BN** to give **P4-BNa** and **P4-BNb**

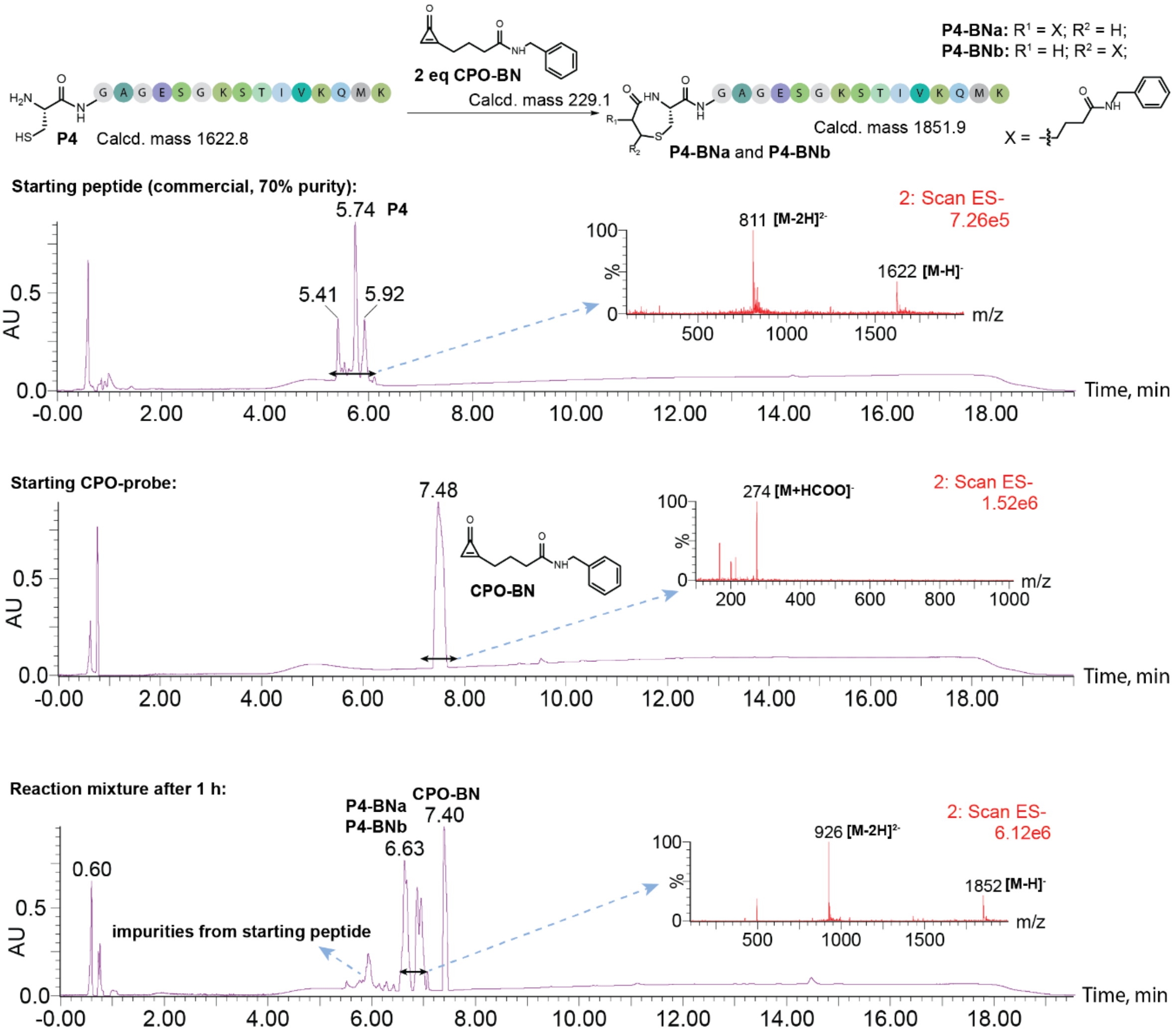

Figure S20. HPLC and MS analysis of reaction between peptide **P4** (25 µL of 0.4 mM stock solution in in NaP$_i$ buffer (pH 7, 20 mM)) and **CPO-BN** (2.5 µL of 8 mM stock solution in CH_3CN, 2 equiv.). The reaction was complete after incubation for 1 h at 4 ºC.

Reduction of peptide **P5** with tris(2-carboxyethyl)phosphine (TCEP) to give **P5-H_2** (**P5-SH)**

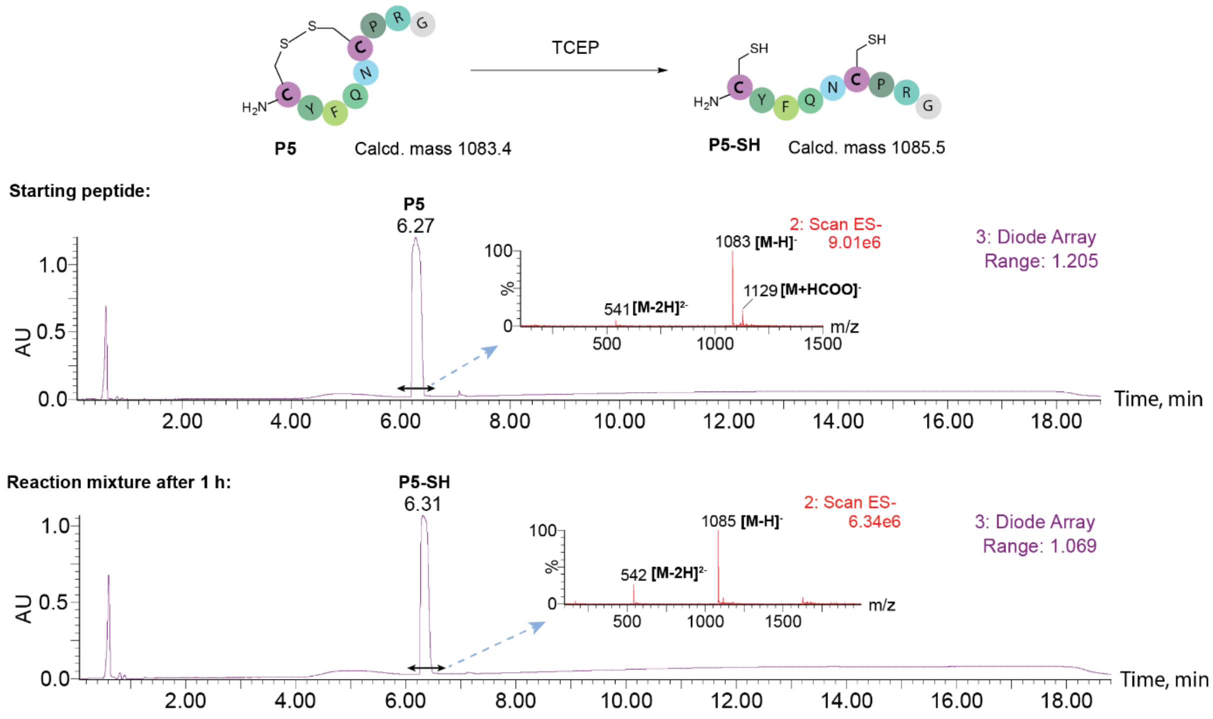

Figure S21. HPLC and MS analysis of reaction between peptide **P5** (50 µL of 2 mM stock solution in in NaP$_i$ buffer (pH 7, 20 mM)) and TCEP (1 µL of 100 mM stock solution in MQ-H_2O, 1 equiv.). The reaction was complete after incubation for 1 h at 25 ºC.

Reaction of peptide **P5-H$_2$** (**P5-SH)** with *N*-methylmaleimide to give **P5-MI**

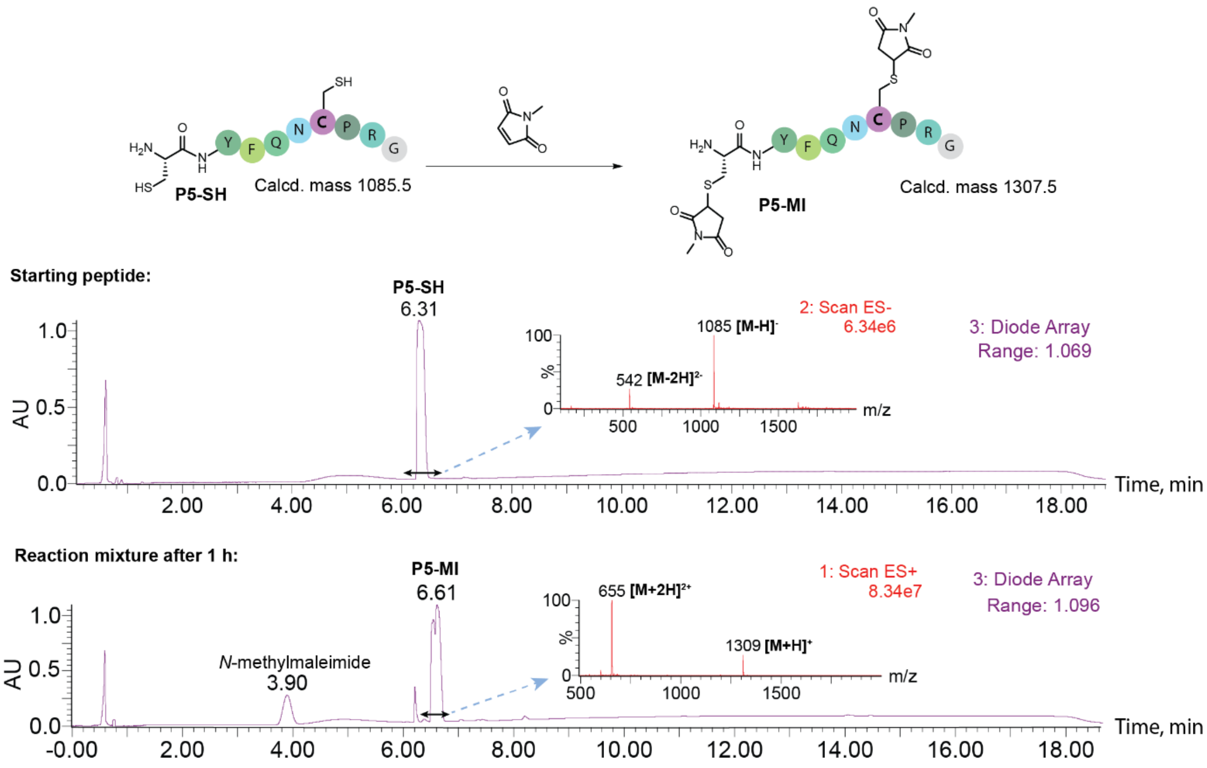

Figure S22. HPLC and MS analysis of reaction between peptide **P5-H$_2$** (**P5-SH)** (25 µL of 0.5 mM stock solution in in NaP$_i$ buffer (pH 7, 20 mM)) and *N*-methylmaleimide (3.9 µL of 8 mM stock solution in CH_3CN, 2.5 equiv.). The reaction was complete after incubation for 1 h at 25 ºC, both cysteine residues were modified.

Reaction of peptide **P5-H$_2$** (**P5-SH**) with 2-cyano-6-hydroxybenzothiazole to give **P5-CBTa** and **P5-CBTb**

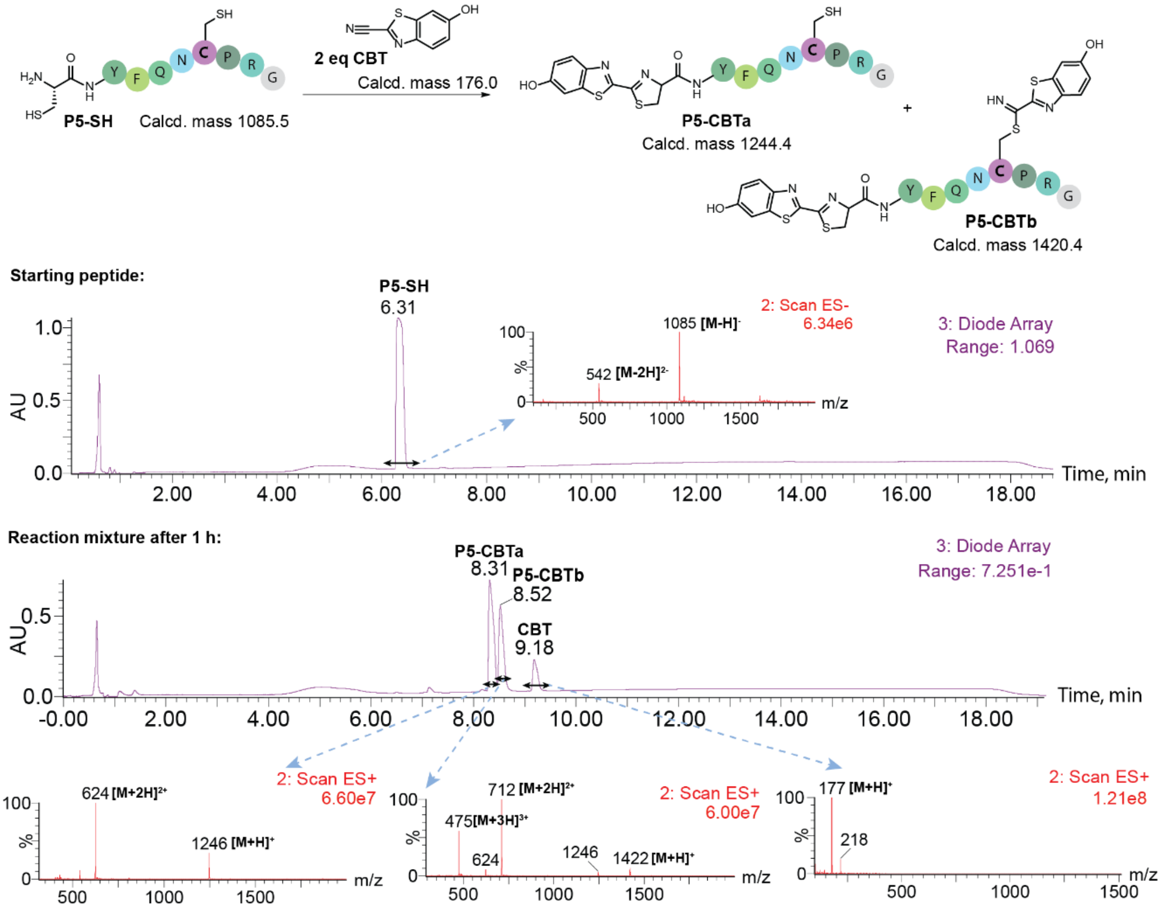

Figure S23. HPLC and MS analysis of reaction between peptide **P5-H$_2$ (P5-SH)** (30 µL of 0.3 mM stock solution in in NaP$_i$ buffer (pH 7, 20 mM)) and 2-cyano-6-hydroxybenzothiazole (1.5 µL of 12 mM stock solution in CH_3CN, 2.0 equiv.). The reaction was analysed after incubation for 1 h at 4 ºC, peptide conjugates with one modification (**P5-CBTa**) and two modifications (**P5-CBTb**) were observed.

Reaction of peptide **P5-H₂ (P5-SH)** with **CPO-BN** to give **P5-BNa** and **P5-BNb**

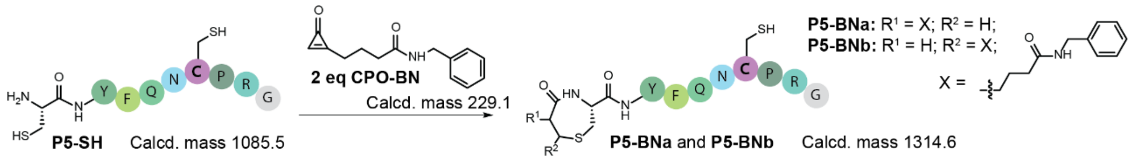

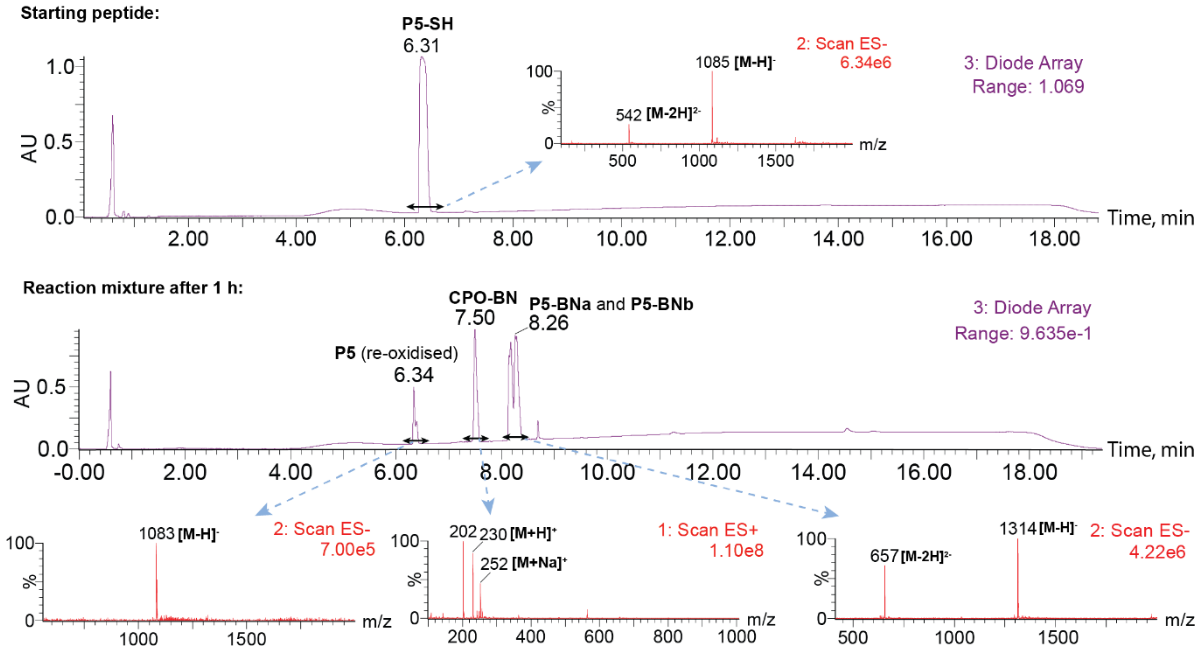

Figure S24. HPLC and MS analysis of reaction between peptide **P5-H₂ (P5-SH)** (30 µL of 0.3 mM stock solution in in NaP$_i$ buffer (pH 7, 20 mM)) with **CPO-BN** (2.25 µL of 8 mM stock solution in CH_3CN, 2.0 equiv.). The reaction was complete after incubation for 1 h at 4 ºC with only one cysteine residue being modified.

Reaction of peptide conjugates **P5-BNa** and **P5-BNb** with N-methylmaleimide to give **P5-BNa-MI** and **P5-BNb-MI**

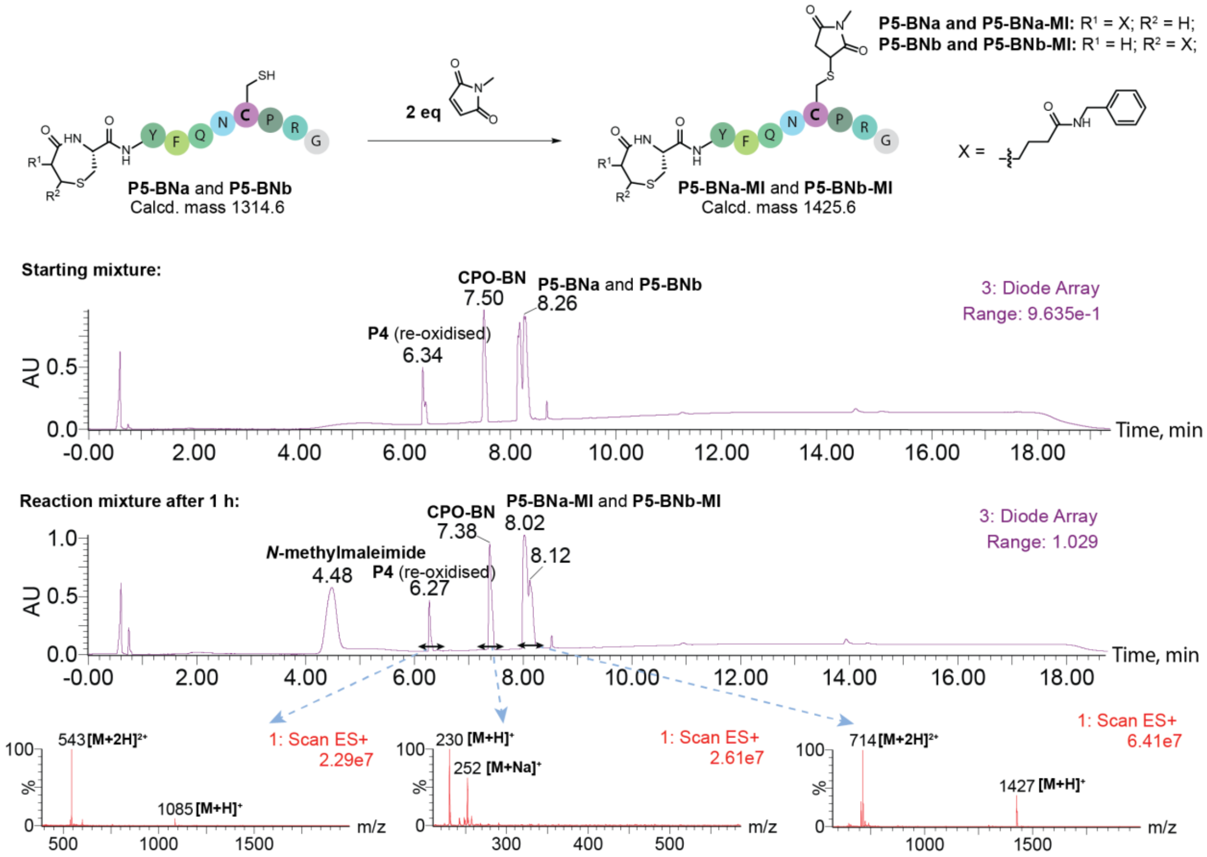

Figure S25. HPLC and MS analysis of reaction between peptides **P5-BNa** and **P5-BNb** (15 µL of the reaction mixture prepared as described above) with *N*-methylmaleimide (1.05 µL of 8 mM stock solution in CH_3CN, 2 equiv.). The reaction was complete after incubation for 1 h at 4 ºC.

LC–MS/MS analysis of **P5-BNa-MI** and **P5-BNb-MI** confirming the modified sites

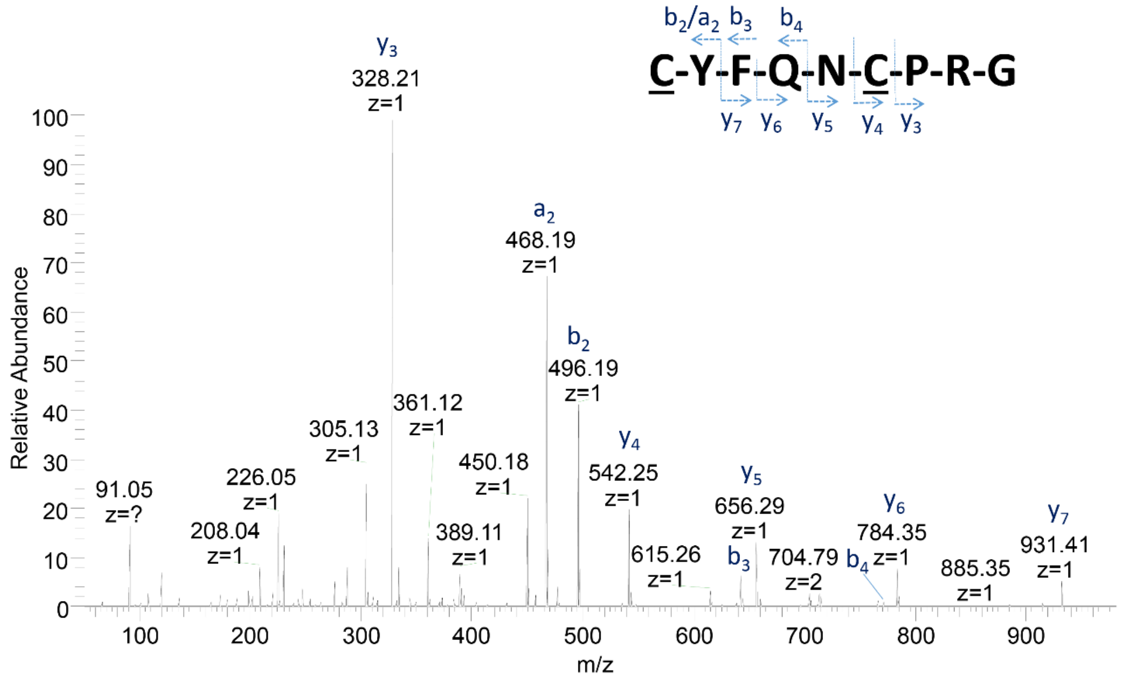

Figure S26. MS/MS spectrum of the *m/z* 713.8 doubly charged ion of the peptide CYFQNCPRG containing a cyclopropenone modification at the N-terminal cysteine residue and an N-methylmaleimide modification at the internal cysteine.

9 Protein modification

9.1 General procedures

9.1.1 Analysis of protein conjugation by LC–MS.

LC–MS method for analysis of protein conjugation. LC–MS was performed on a Waters SQ Detector 2 mass spectrometer coupled to an Acquity UPLC system using an Acquity UPLC Protein BEH C4 column (300Å, 1.7 µm, 2.1 mm × 50 mm). Solvents A, water with 0.1% formic acid and B, 71% acetonitrile, 29% water and 0.075% formic acid were used as the mobile phase at a flow rate of 0.2 mL min^{-1}. The gradient was programmed as follows: 28% B to 71.2% B in 12 min, then 71.2% B for 1 min, 100% B for 2 min, and after that 28% A for 4 min. The electrospray source was operated with a capillary voltage of 3.0 kV and a cone voltage of 30 V. Nitrogen was used as the desolvation gas at a total flow of 800 L h^{-1}. Total mass spectra were reconstructed from the ion series using the MaxEnt algorithm preinstalled on MassLynx software (v. 4.1 from Waters) according to the manufacturer's instructions or using UniDec (4.4.0) using the default settings and a mass range set to 1 Da. To obtain the ion series described, the major peak(s) of the chromatogram were selected for integration and further analysis.

A typical analysis of a conjugation reaction by LC–MS is described below. The total ion chromatogram, combined ion series and deconvoluted spectra are shown for the product of the reaction. Identical analyses were carried out for all the conjugation reactions performed in this work.

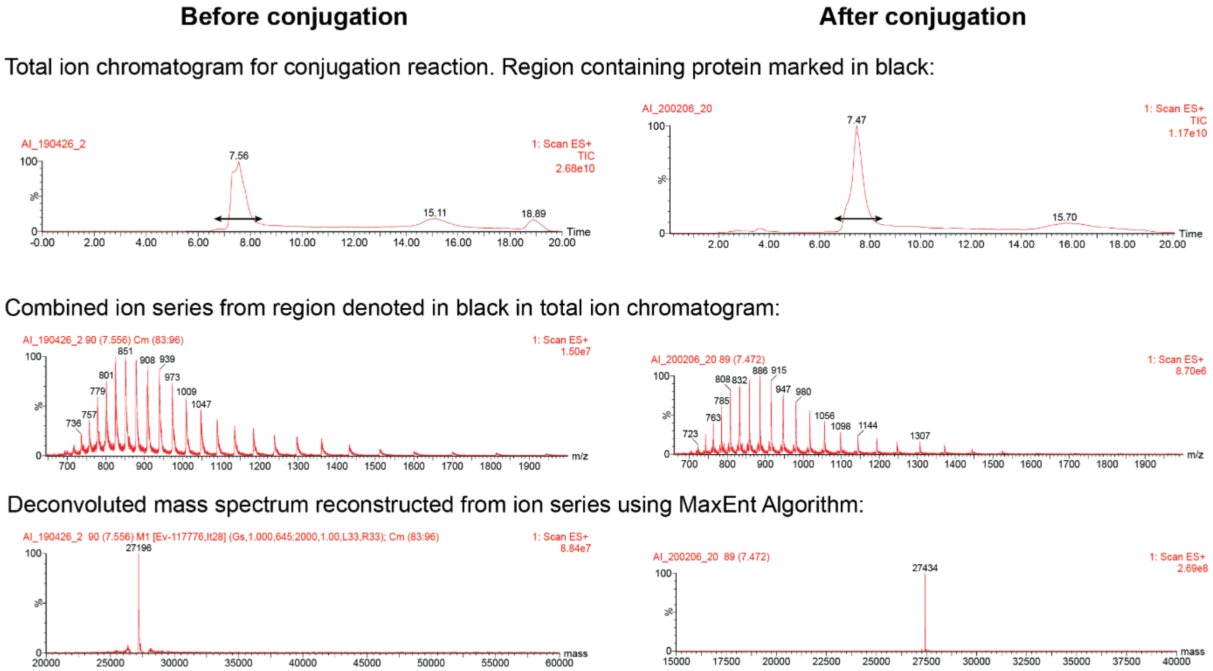

Figure S27. A typical analysis of a conjugation reaction by LC–MS is described for the reaction of **Cys-GFP** protein with cyclopropenone derivative **CPO-BN**. The total ion chromatogram, combined ion series and deconvoluted spectra are shown for the starting material and the product of the reaction of **Cys-GFP** with 100 equiv. of **CPO-BN**. Identical analyses were carried out for all the conjugation reactions performed in this work.

9.1.2 Enzymatic digestion and LC–MS/MS analysis

Protein solutions were subjected to enzymatic digestion in 50 mM ammonium bicarbonate (pH 8) with trypsin overnight at 37 °C. After digestion, the supernatant was pipetted into a sample vial and loaded onto an autosampler for automated LC–MS/MS analysis.

All LC–MS/MS experiments were performed using a Dionex Ultimate 3000 RSLC nanoUPLC (Thermo Fisher Scientific Inc, Waltham, MA, USA) system and a QExactive Orbitrap mass spectrometer (Thermo Fisher Scientific Inc, Waltham, MA, USA). Separation of peptides was performed by reverse-phase chromatography at a flow rate of 300 nL/min and a Thermo Scientific reverse-phase nano Easy-spray column (Thermo Scientific PepMap C18, 2 µm particle size, 100 Å pore size, 75 µm i.d. × 50 cm length). Peptides were loaded onto a pre-column (Thermo Scientific PepMap 100 C18, 5 µm particle size, 100 Å pore size, 300 µm i.d. × 5mm length) from the Ultimate 3000 autosampler with 0.1% formic acid for 3 minutes at a flow rate of 10 µL/min. After this period, the column valve was switched to allow elution of peptides from the pre-column onto the analytical column. Solvent A was water + 0.1% formic acid and solvent B was 80% acetonitrile, 20% water + 0.1% formic acid. The linear gradient employed was 2–40% B in 30 minutes.

The LC eluant was sprayed into the mass spectrometer by means of an Easy-Spray source (Thermo Fisher Scientific Inc.). All *m/z* values of eluting ions were measured in an Orbitrap mass analyser, set at a resolution of 70000 and was scanned between *m/z* 380-1500. Data dependent scans (Top 20) were employed to automatically isolate and generate fragment ions by higher energy collisional dissociation (HCD, NCE:25%) in the HCD collision cell and measurement of the resulting fragment ions was performed in the Orbitrap analyser, set at a resolution of 17500. Singly charged ions and ions with

unassigned charge states were excluded from being selected for MS/MS and a dynamic exclusion window of 20 seconds was employed.

Post-run, the data was processed using Protein Discoverer (version 2.1., Thermo Scientific). Briefly, all MS/MS data were converted to mgf files and the files were then submitted to the Mascot search algorithm (Matrix Science, London UK) and searched against a custom database containing the GFP and a common contaminant sequences (123 sequences; 40594 residues). Variable modifications of oxidation (M), deamidation (NQ) and the **CPO-BN** modification were applied. The peptide and fragment mass tolerances were set to 100 ppm and 0.1 Da, respectively. A significance threshold value of $p<0.05$ and a peptide cut-off score of 20 were also applied.

9.1.3 Circular dichroism spectroscopy

Circular dichroism (CD) spectra were recorded on a Jasco J-810 Spectropolarimeter fitted with a temperature-controlled cell. Samples were analysed in a quartz cuvette (1 mm path length) between 250–190 nm at 25 °C and spectra were averaged over 50 scans. Background spectra of the buffers were acquired immediately prior to data acquisition. The buffer used throughout was NaP_i (20 mM, pH 7); in the case of **Cys-GFP** and **2×Cys-GFP-1**, and **cys-nIL2** the buffer also contained TCEP (100 µM) in order to maintain the cysteine residues in their reduced forms. The concentration of protein was adjusted such that the value for [H/T] did not exceed 600 (typically 1–4 µM). Data were processed in Excel and plotted in gnuplot.

9.1.4 Bio-layer interferometry (BLI)

BLI was performed on a K2 Octet system using streptavidin-coated sensors (Sartorius). The buffer used throughout was 10 mM HEPES, 150 mM NaCl, 3 mM EDTA, 0.05% (v/v) surfactant P-20, and 10 µM BSA. Tips underwent the following steps (30 °C, 500 Hz shaking): Equilibration (60 s), Loading (85 s), Wash (60 s), Baseline (120 s), Association (210 s), Dissociation (450 s). Experimental runs were limited to 1 hour to minimise evaporation. Loading was performed with biotinylated Human β-IL2 receptor (Generon) at 1 ug/mL. Raw sensorgrams were processed in R by first subtracting the background from a reference sample (biotinylated ligand, no analyte). A reference biosensor (no biotinylated ligand) showed no background binding and so were discontinued after the first experiment. Smoothing was applied using Savitzky–Golay filter and sensorgrams were normalised to the average of the last 10 seconds of the baseline step. Fitting was performed with the nls function in R according to the following model:

Association step:

$$y = E_q \cdot (1 - \exp(K_{ob} \cdot t))$$

Dissociation step:

$$y = E_q \cdot (1 - \exp(-K_{ob} \cdot t_0)) \cdot \exp\,(-k_d \cdot (t - t_0))$$

Where:

$$E_q = R_{max} \cdot ([analyte]/([analyte] + \left(\frac{k_d}{k_a}\right))$$

$$K_{ob} = [analyte] \cdot (k_a + k_d)$$

And R_{max} is the maximum response for a given sensor and $t_0$is the time of the association step. R_{max} is a sensorgram-specific (local) parameter and other parameters are global.

9.2 Proteins used in this study

9.2.1 Cys-GFP

Enhanced Green fluorescent protein with N-terminal cysteine (**Cys-GFP**) was expressed and purified as described below.

Genetic construct

The pBAD-EGFP vector containing the EGFP gene was purchased from Addgene (Catalog #54762). This vector contains a N-terminal 6×His-T7-TEV-EGFP gene construct. To introduce a cysteine at the N-terminus of the enhanced green fluorescent protein (EGFP), a single point mutation in the glycine residue of the TEV sequence (ENLYFQ/G) was generated by the NZYMutagenesis kit (nzytech) using the mutagenic primers 5′-gaacctgtacttccagtgctcgagcatggtgag-3’ and 5’-ctcaccatgctcgagcactggaagtaca-ggttc-3’. The amplification products were transformed into *E. coli* NZY5α and the (His-Cys-EGFP) plasmid construct was confirmed by PCR and sequencing at Eurofins Genomics Facility.

Cell growth and protein purification

The vector encoding the His-Cys-EGFP gene was used to transform competent *E. coli* cells (BL21.A1 strain). For protein expression, cells were inoculated into *Luria Bertani* (LB) media containing 100 μg/mL of carbenicillin and grown at 37 ºC, until an optical density of 0.4–0.6 was reached. Expression of the EGFP protein was induced by adding 1 mM isopropyl β-D-1-thiogalactopyranoside (ITPG) and 0.2% of L-arabinose, and the culture is left to incubate with shaking at 18 ºC for 4–6 hours. Cells were harvested by centrifugation and the pellet was resuspended in 1:3 of cold lysis buffer containing 50 mM Tris-HCl pH 7.5, 150 mM NaCl, 20 mM imidazole, EDTA-free Protease Inhibitor Cocktail (cOmplete™, Roche), and 1 mM DTT. The cells were lysed by sonication for 4 min in total (20s on, 20s off/cycle) and the lysate was clarified by centrifugation at 18000 rpm, for 30 min. The supernatant was then loaded into a HisTrap™ HP affinity column (GE Healthcare Life Sciences), pre-equilibrated with 20 mM Tris-HCl pH 7.5, 150 mM NaCl, 20 mM imidazole, and 1 mM DTT. The His-Cys-GFP protein was eluted with 20 mM Tris-HCl pH 7.5, 150 mM NaCl, 20 mM imidazole, 1 mM DTT and 500 mM of imidazole. Fractions enriched with His-Cys-GFP, were pooled and treated with TEV protease (*in house*) to remove the 6×His-tag. The treated sample was loaded into a HisTrap™ column, and the flowthrough (containing the TEV treated Cys-GFP), was pooled and concentrated to 4 mg/mL. Samples containing the **Cys-GFP** protein were analysed by SDS-PAGE (**Figure S28**) and LC–MS (**Figure S29**).

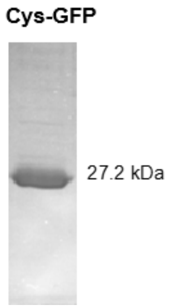

Figure S28. 15% SDS-PAGE of Cys-GFP protein.

Before modification reactions, a batch of **Cys-GFP** (100 µL, 30 µM) was treated with TCEP (1 µL, 30 mM, 10 equiv.) for 1 h at 25 °C. Small molecules were removed Amicon Ultra-0.5 mL Centrifugal Filter (MWCO 3 KDa) to afford the **Cys-GFP** (80 µL, 33 µM) with a reactive cysteine in NaP_i buffer (pH 7, 20 mM). For storage, DTT (0.88 µL, 1.5 M, 500 equiv.) was added to the buffer to prevent cysteine oxidation (final DTT concentration is 16.5 mM).

Amino acid sequence (20 lysines, 3 cysteines, no disulfides):

CSSMVSKGEELFTGVVPILVELDGDVNGHKFSVSGEGEGDATYGKLTLKFICTTGKLPVPWP
TLVTTLTYGVQCFSRYPDHMKQHDFFKSAMPEGYVQERTIFFKDDGNYKTRAEVKFEGDTL
VNRIELKGIDFKEDGNILGHKLEYNYNSHNVYIMADKQKNGIKVNFKIRHNIEDGSVQLADH
YQQNTPIGDGPVLLPDNHYLSTQSALSKDPNEKRDHMVLLEFVTAAGITLGMDELYK

Isotopically Averaged Molecular Weight = 27199 Da.

Note: Formation of the fluorophore in Green Fluorescent Proteins results in the formal loss of H_2O and H_2, resulting in an observed mass 20 Da lower than the mass of the sequence (27219 Da).

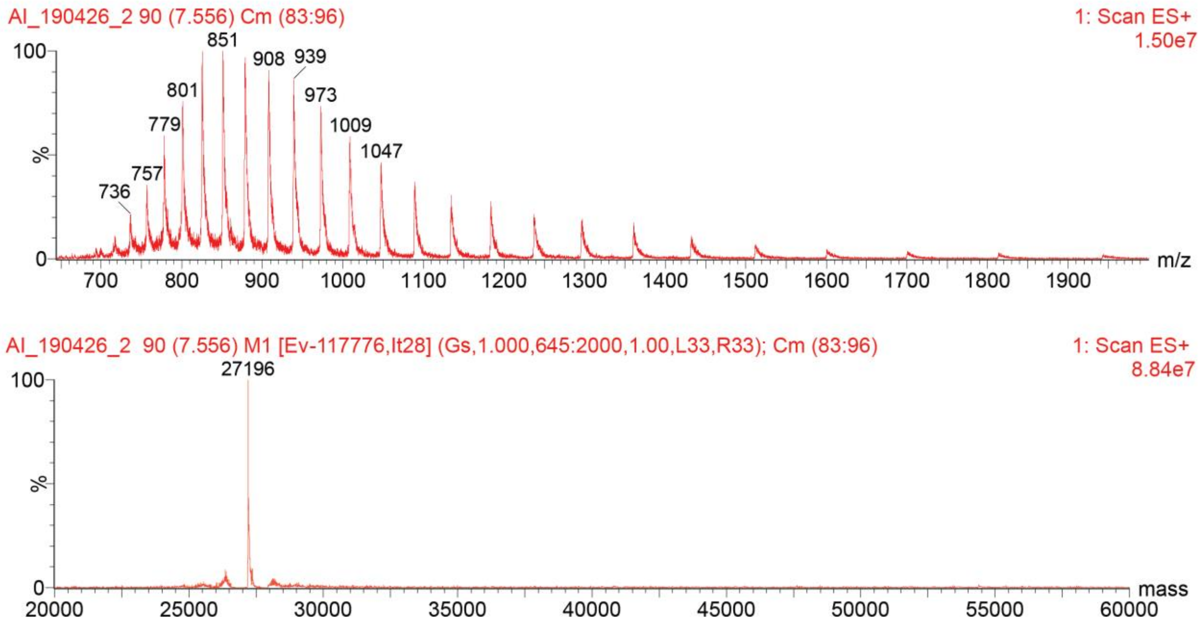

Figure S29. Combined ion series and deconvoluted mass spectra of **Cys-GFP**. Calcd. mass, 27199 Da, found: 27196 Da.

9.2.2 2×Cys-GFP

To produce the **2×Cys-GFP** protein, a S147C point mutation was introduced using the Phusion Site-Directed Mutagenesis Kit (Thermo Fisher Scientific). The mutagenic primers were 5′-AGTACA-ACTACAACTGCCACAACGTCTAT-3’, and 5’-TTGTACTCCAGCTTGTGCCCCAGGAT-3’. The constructs were transformed into the NZY5α strain of E. coli for DNA amplification, and then to the BL21.A1 strain.

For protein expression, cells were inoculated into 250 mL of LB media containing 100 µg/mL of carbenicillin and grown at 37 °C, until an optical density of 0.4–0.6 was reached. Induction was then carried out with 1 mM ITPG and 0.2% of L-arabinose, for 4–6 hours at 18 °C. Cells were harvested by

centrifugation at 8,000 rpm, for 20 min, at 4 °C. The pellet was resuspended in 1:3 of cold lysis buffer containing 50 mM Tris-HCl pH 7.5, 150 mM NaCl, 20 mM imidazole, 1 tablet of EDTA-free Protease Inhibitor Cocktail (cOmplete™, Roche), and 1 mM DTT, and lysed by sonication for 4 minutes total (20s on, 20s off/cycle). The lysate was clarified by centrifugation at 18,000 rpm, for 30 min, and the supernatant was loaded into a HisTrap™ HP affinity column (GE Healthcare Life Sciences), equilibrated with 20 mM Tris-HCl pH 7.5, 150 mM NaCl, 20 mM imidazole, and 1 mM DTT. The captured protein was eluted with 10% of elution buffer containing imidazole (500 mM). Fractions enriched with EGFP and without contaminants, were pooled and treated with TEV protease (produced in house) to remove the His-tag. The treated sample was loaded into a HisTrap™ column, and the flowthrough (containing the TEV treated EGFP), was pooled and concentrated to 4 mg/mL. The expression and purity of the protein was confirmed by SDS-PAGE gel and mass spectrometry.

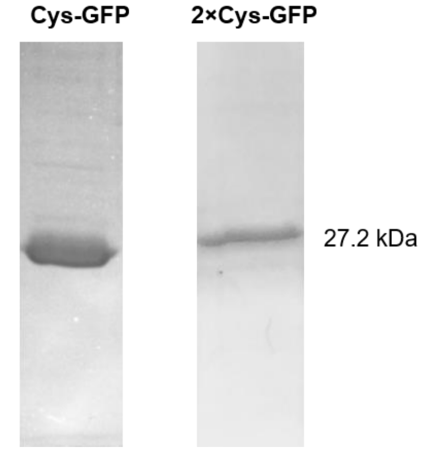

Figure S30. Analysis of purified **Cys-GFP** and **2×Cys-GFP** by SDS-PAGE.

Amino acid sequence (20 lysines, 3 cysteines, no disulfides):

CSSMVSKGEELFTGVVPILVELDGDVNGHKFSVSGEGEGDATYGKLTLKFICTTGKLPVPWP
TLVTTLTYGVQCFSRYPDHMKQHDFFKSAMPEGYVQERTIFFKDDGNYKTRAEVKFEGDTL
VNRIELKGIDFKEDGNILGHKLEYNYN**C**HNVYIMADKQKNGIKVNFKIRHNIEDGSVQLADH
YQQNTPIGDGPVLLPDNHYLSTQSALSKDPNEKRDHMVLLEFVTAAGITLGMDELYK

Isotopically Averaged Molecular Weight = 27214.84 Da.

Note: Formation of the fluorophore in Green Fluorescent Proteins results in the formal loss of H_2O and H_2, resulting in an observed mass 20 Da lower than the mass of the sequence (27234.84 Da).

Prior to protein modification reactions, an aliquot of **2×Cys-GFP** (180 µM, 50 µL, 20 mM Tris-HCl, pH 8.0, 150 mM NaCl) was subjected to buffer exchange using an Amicon Ultra-0.5 mL centrifugal filter (MWCO 3 kDa), according to the manufacturer's instructions, to give **2×Cys-GFP** (45 µM, 200 µL, 20 mM NaP_i, pH 7). The sample was then treated with TCEP (6 µL, 30 mM in water, 20 equiv) for 1.5 h at 25 °C. Excess TCEP was removed using an Amicon Ultra-0.5 mL centrifugal filter (MWCO 3 kDa), according to the manufacturer's instructions, to give **2×Cys-GFP** (45 µM, 200 µL, 20 mM NaP_i, 4.5 mM DTT, pH 7). Analysis of the resulting solution by LC–MS showed the presence of a single protein (found: 27222Da; theoretical: 27215 Da).

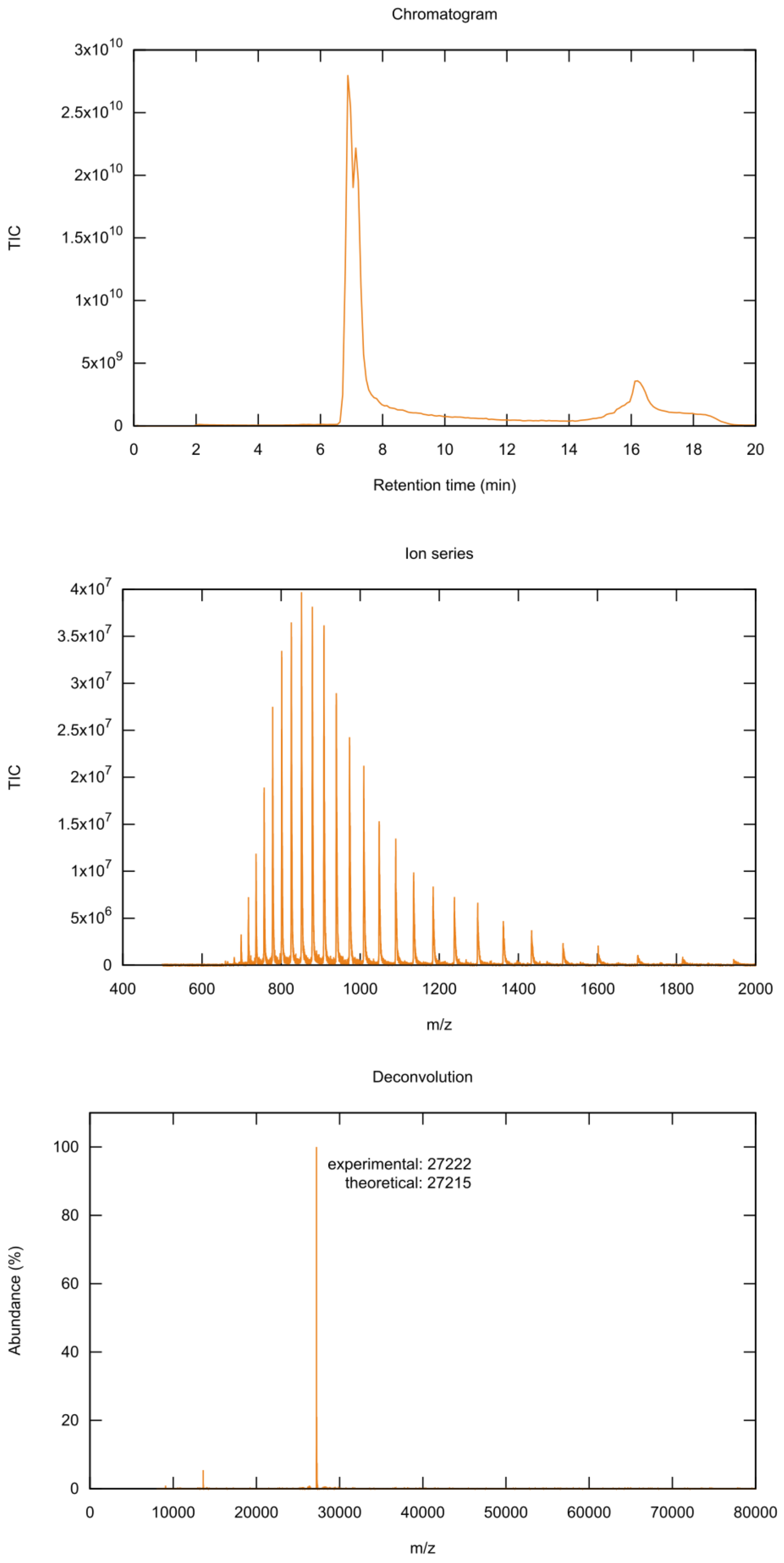

Figure S31. Top: Chromatogram of **2×Cys-GFP** as described in this section. Middle: Combined ion series of protein region. Bottom: Deconvoluted mass spectrum.

9.2.3 C2Am

Engineered variant of the C2A domain of Synaptotagmin-I Cys 95 mutant (**C2Am**) was kindly provided by Dr. André Neves.[10]

Amino acid sequence (14 lysines, 1 free cysteine, no disulfides):

GSPGISGGGGGILDSMVEKLGKLQYSLDYDFQNNQLLVGIIQAAELPALDMGGTSDPYVKVF
LLPDKKKKFETKVHRKTLNPVFNEQFTFKVPY**C**ELGGKTLVMAVYDFDRFSKHDIIGEFKVP
MNTVDFGHVTEEWRDLQSAEK

Isotopically Averaged Molecular Weight = 16222 Da

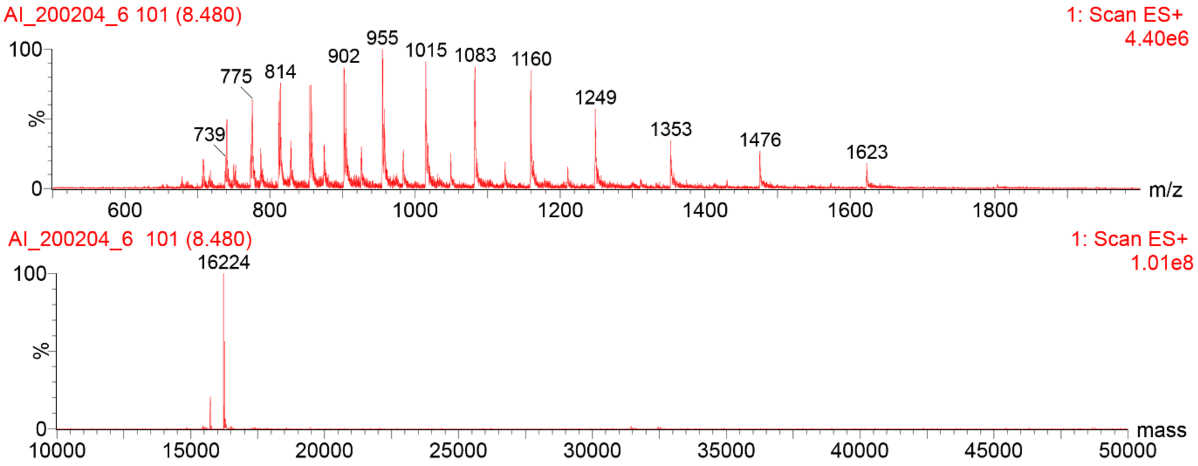

Figure S32. Combined ion series and deconvoluted mass spectra of **C2Am**. Calcd. mass, 16222 Da, found: 16224 Da.

9.2.4 Annexin V

Annexin V was expressed and purified as previously reported.[11]

Amino acid sequence (22 lysines, 1 free cysteine, no disulfides):

AQVLRGTVTDFPGFDERADAETLRKAMKGLGTDEESILTLLTSRSNAQRQEISAAFKTLFGRD
LLDDLKSELTGKFEKLIVALMKPSRLYDAYELKHALKGAGTNEKVLTEIIASRTPEELRAIKQ
VYEEEYGSSLEDDVVGDTSGYYQRMLVVLLQANRDPDAGIDEAQVEQDAQALFQAGELKW
GTDEEKFITIFGTRSVSHLRKVFDKYMTISGFQIEETIDRETSGNLEQLLLAVVKSIRSIPAYLAE
TLYYAMKGAGTDDHTLIRVMVSRSEIDLFNIRKEFRKNFATSLYSMIKGDTSGDYKKALLLL
CGEDD

Isotopically Averaged Molecular Weight = 35805 Da

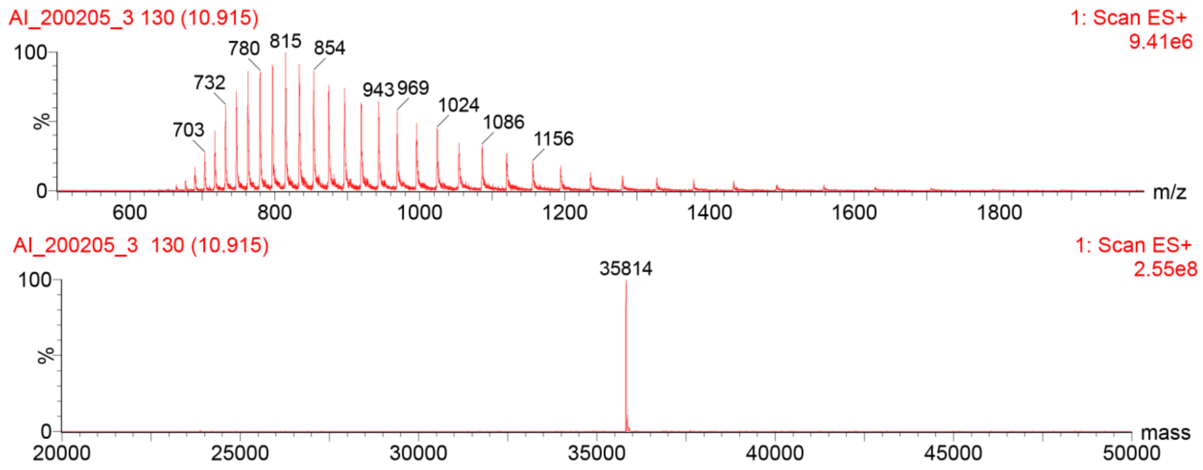

Figure S33. Combined ion series and deconvoluted mass spectra of Annexin V. Calcd. mass, 35805 Da, found: 35814 Da.

9.2.5 Nanobody DesAB-HET[12]

Nanobody Cys57 mutant was kindly provided by Philip Lindstedt and Prof. Michele Vendruscolo.

Amino acid sequence (7 lysines, 1 free cysteine, 1 disulfide):

MRGSHHHHHHGMASMTGGQQMGRDLYDDDDKDPKLEVQLVESGGGLVQPGGSLRLSCAA
SGFNIKDTYIGWVRRAPGKGKEWVASIYPTNGYTRYADSVKGRFTISADTSKNTAYLQMNSL
RAEDTAVYYCAAGSCETLTLREEEAAAWGQGTLVTVSSGT

Isotopically Averaged Molecular Weight = 17391 Da

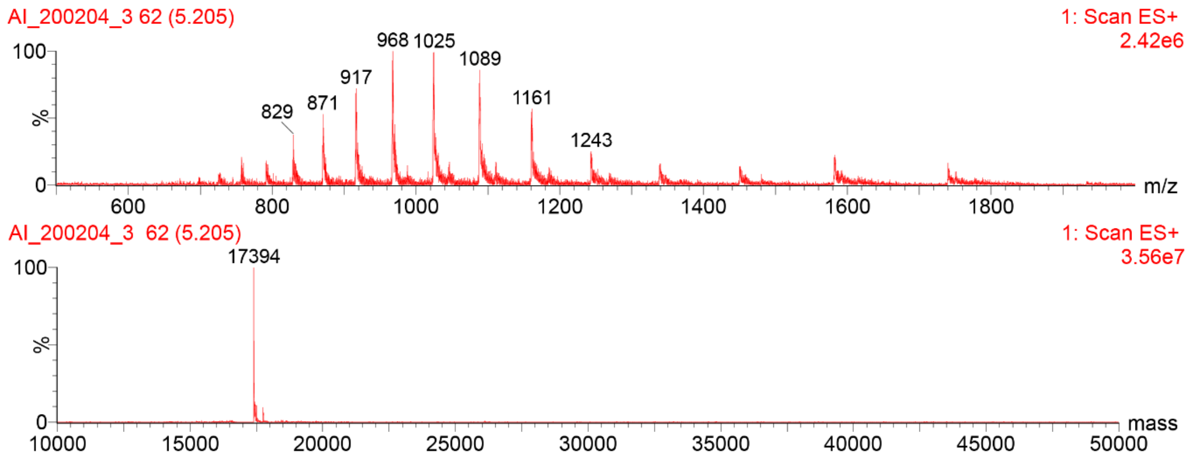

Figure S34. Combined ion series and deconvoluted mass spectra of DesAB-HET nanobody. Calcd. mass, 17391 Da, found: 17394 Da.

9.2.6 cys-nIL2

His-cys-nIL2 (MW: 14146.92, pI 5.40, 122 aa), ***enterokinase cleavage site

MGSSHHHHHHSSGDDDDK***CSHMPKKKIQLHAEHALYDALMILNIVKTNSPPAEEKLEDYAFNFELILEEIARLFESGDQKDEAEKAKRMKEWMKRIKTTASEDEQEEMANAIITILQSWIFS

Observed MS: 14015 (nIL2 – Met), 14136 (nIL2 – Met + Cys via disulfide), 14321 (nIL2 – Met + glutathione via disulfide)

Cys-nIL2 (MW: 12141.94, pI 5.07, 104 aa)

CSHMPKKKIQLHAEHALYDALMILNIVKTNSPPAEEKLEDYAFNFELILEEIARLFESGDQKDEAEKAKRMKEWMKRIKTTASEDEQEEMANAIITILQSWIFS

Observed MS: 12143

Protein expression via high-density transient gene expression

CHO-S cells were grown at 37 °C (150 rpm) in Power-CHO medium (Lonza), supplemented with 8 mM ultraglutamine (Lonza), 4 mM HT-supplement (Gibko) and 1 × antibiotic-antimycotic (Gibco). Cells were kept at a density around 0.5–8 million cells/mL.

For high-density transient gene expression, CHO-S cells were taken up in Power-CHO supplemented with 8 mM ultraglutamine (Lonza), 4 mM HT-supplement and 1x antibiotic-antimycotic (Gibco) at a concentration of 4 million cells/mL. PEI (1 g/L final) and subsequently the plasmid (0.8 µg/10^6 cells) were added to incubate the resulting mixture for 6 days at 31 °C in a shaker incubator (140 rpm).

CHO-S cells were removed by centrifugation and the supernatant filtered (0.22 µm, TPP). **His-cys-nIL2** was captured on Ni-NTA resin (Roche), washed with 300 mM NaCl and 5 mM Imidazole in PBS pH 7.4 and subsequently eluted with 500 mM imidazole in PBS pH 7.4 (no additional purification was needed). The protein was dialyzed into PBS pH 7.4 or enterokinase buffer (20 mM Tris and 1 mM EDTA, pH 7.5) to proceed with the cleavage.

Enterokinase cleavage

To a solution of **His-cys-nIL2** in 20 mM Tris and 1 mM EDTA pH 7 at 1 mg/mL, enterokinase from bovine intestine (Sigma Aldrich) was added at 20U/mg. The cleavage proceeded overnight at 37 °C.

The cleaved protein was purified via size exclusion chromatography with a Superdex 75 10/300GL column on an Äkta Pure FPLC system (Cytiva) with PBS as mobile phase at a flowrate of 0.75 mL/min.

Biochemical analysis of the proteins were performed with SDS-PAGE and Coomassie staining, LC–MS analysis, and circular dichroism (Section 9.5.1). For SDS-PAGE (TruPAGE Precast gels 4-12%, Sigma-Aldrich), 3 µg protein premixed with non-reducing TruPAGE LDS Sample Buffer (Sigma-Aldrich) was loaded and the gel run for 1 h at 180V and 110 mA in TruPAGE TEA-Tricine SDS Running Buffer (Sigma-Aldrich). The gel was incubated for 30 min in Coomassie staining solution for subsequent destaining (destaining solution: 100 mL AcOH + 300 mL MeOH + 600 mL MQ).

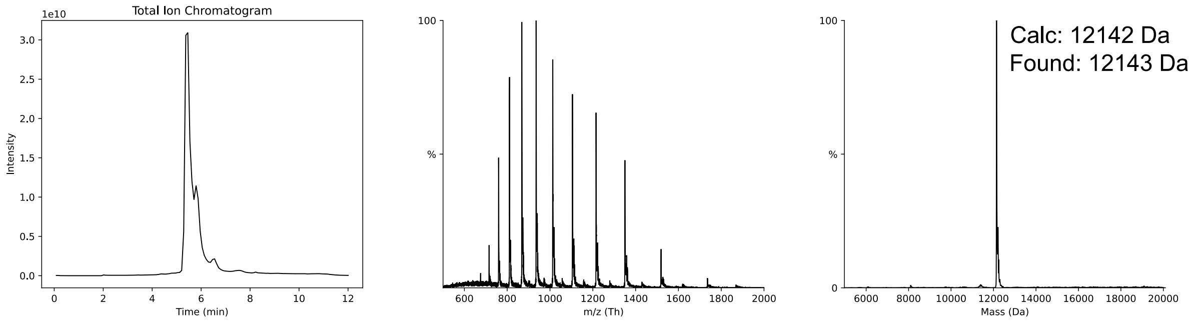

Figure S35. Characterization of **cys-NIL2** by LC–MS.

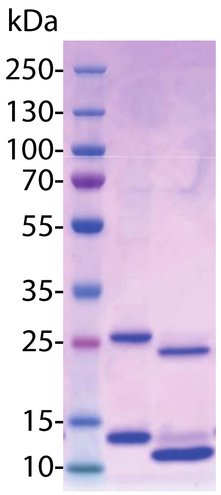

Figure S36. Coomassie stained SDS protein gel (non-reducing conditions) of **His-cys-nIL2** and **cys-nIL2**. Ladder: PageRuler Plus Prestained Protein Ladder (Thermo Scientific, #26620).

9.3 Reactions and characterization of Cys-GFP-conjugates

9.3.1 Reaction of Cys-GFP with CPO-BN to give GFP-CPO-BN

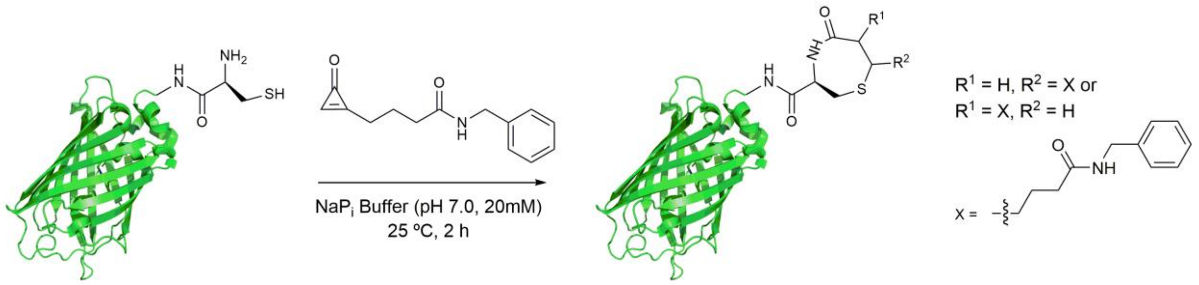

Figure S37. Modification of **cys-GFP** with **CPO-BN**.

A mixture of **Cys-GFP** stock solution (8 µL, 30 µM in 20 mM pH 7 NaP_i buffer containing 16.5 mM DTT), 12 µL of 20 mM pH 7 NaP_i buffer, and 1.5 µL of cyclopropenone reagent **CPO-BN** (16 mM in CH_3CN, 100 equiv.) was shaken in an 1.5 mL microcentrifuge tube at 25 °C for 2 h. The modified product was characterized by LC-MS analysis and complete conversion to the expected product was observed (calculated mass, 27428 Da; observed mass, 27434 Da).

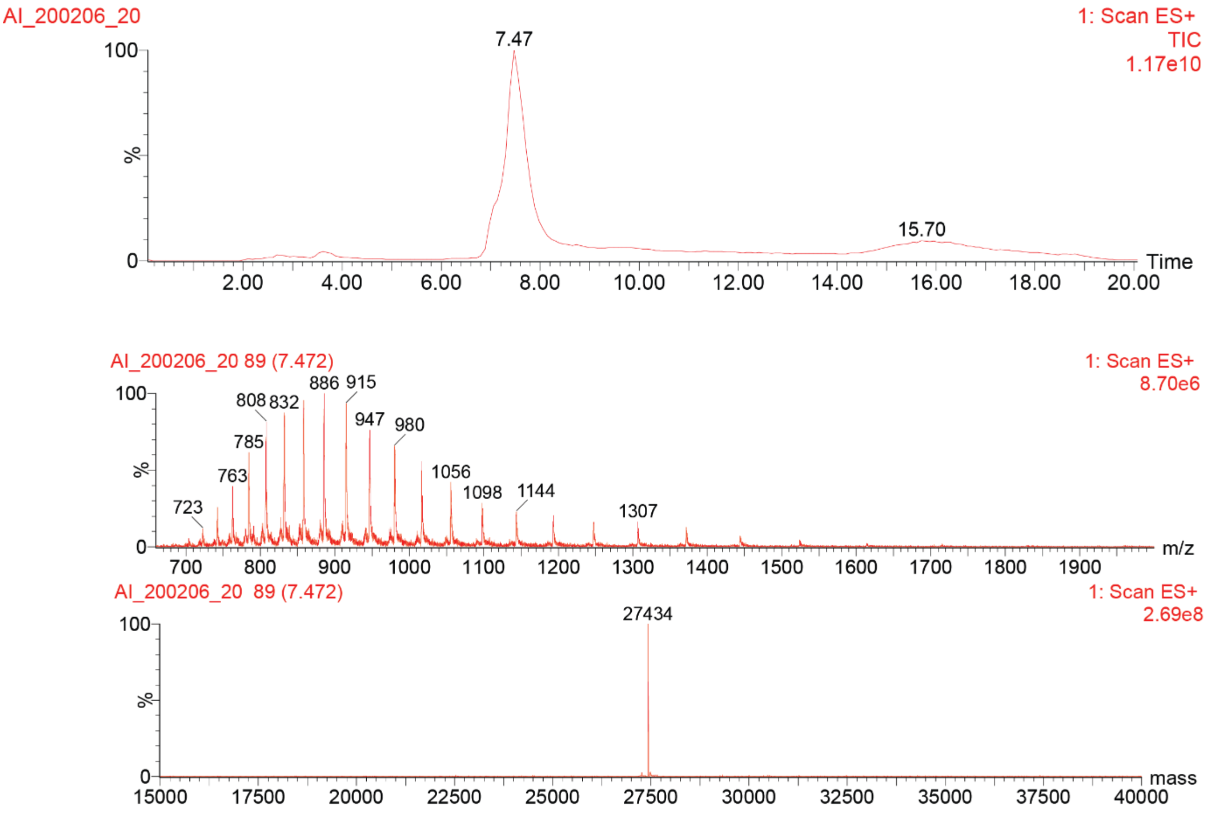

Figure S38. The total ion chromatogram, combined ion series and deconvoluted mass spectrum of the reaction between **Cys-GFP** and **CPO-BN** after 2 h at 25 ºC.

9.3.2 Stability of GFP-CPO-BN in glutathione (GSH).

A 30 µL aliquot of **GFP-CPO-BN** (5 µM) in 20 mM NaP_i buffer at pH 7.0 was thawed. Then, a freshly prepared solution of L-Glutathione reduced form (3 µL, 55 mM stock in MQ-H_2O) was added at room temperature and the resulting mixture vortexed for 10 seconds (final GSH concentration is 5 mM). The resulting reaction mixture was then shaken at 37 ºC. After 1 and 24 h, an aliquot of the reaction mixture was analyzed by LC–MS.

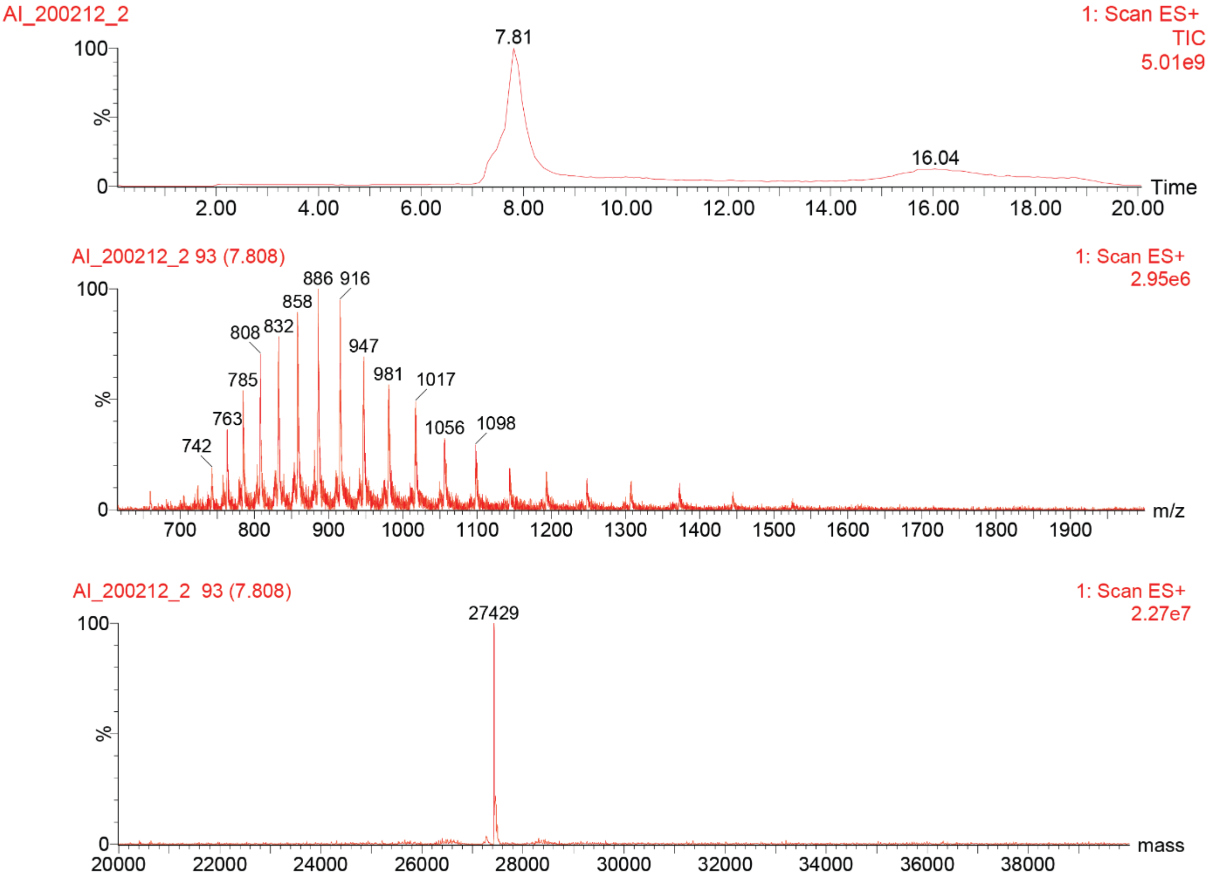

Figure S39. LC–MS trace, combined ion series and deconvoluted mass spectrum of **GFP-CPO-BN** in GSH. Incubation of the bioconjugate **GFP-CPO-BN** with 5 mM with GSH shows no degradation after 1 h at 37 ºC.

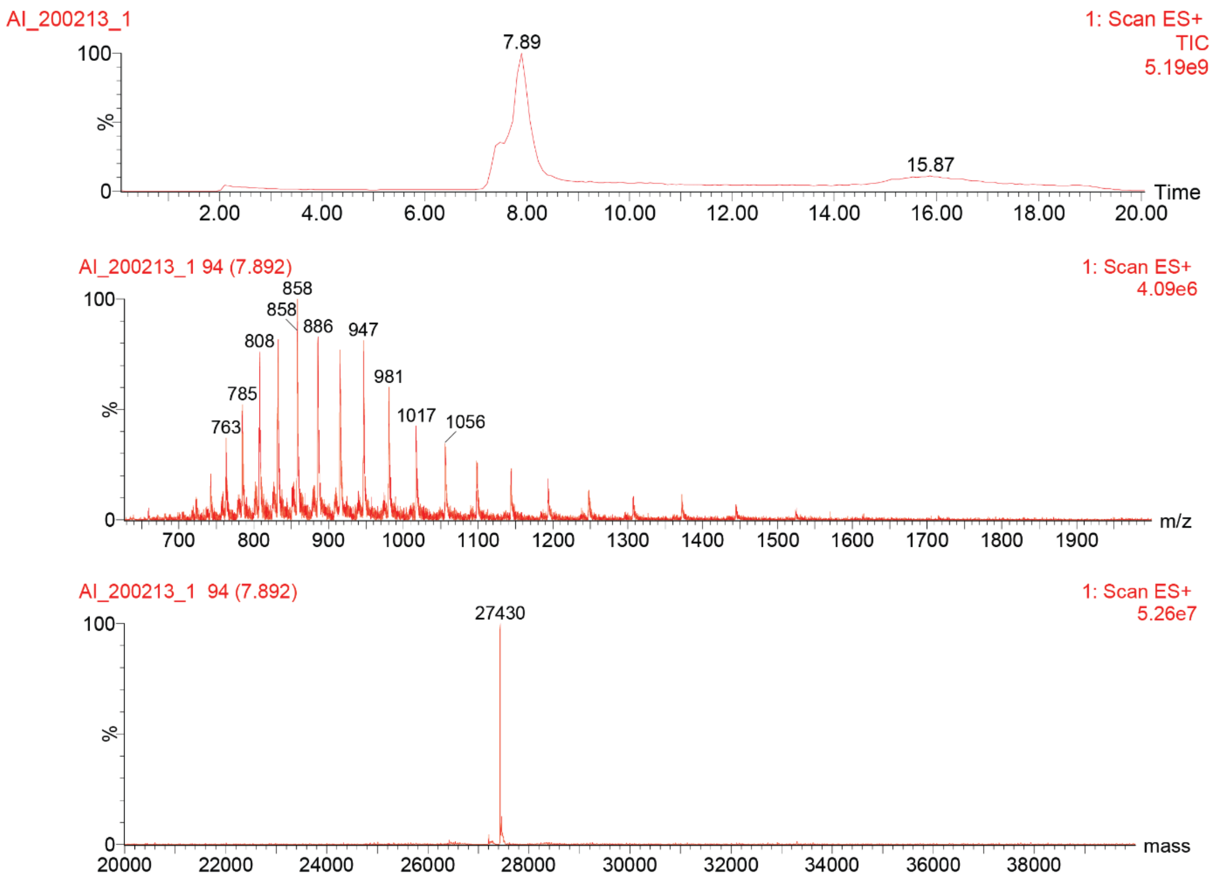

Figure S40. LC–MS trace, combined ion series and deconvoluted mass spectrum of **GFP-CPO-BN** in **GSH**. Incubation of the bioconjugate **GFP-CPO-BN** with 5 mM with **GSH** shows no degradation after 24 h at 37 ºC.

9.3.3 Enzymatic digestion of GFP-CPO-BN and LC–MS/MS analysis confirming the modified site

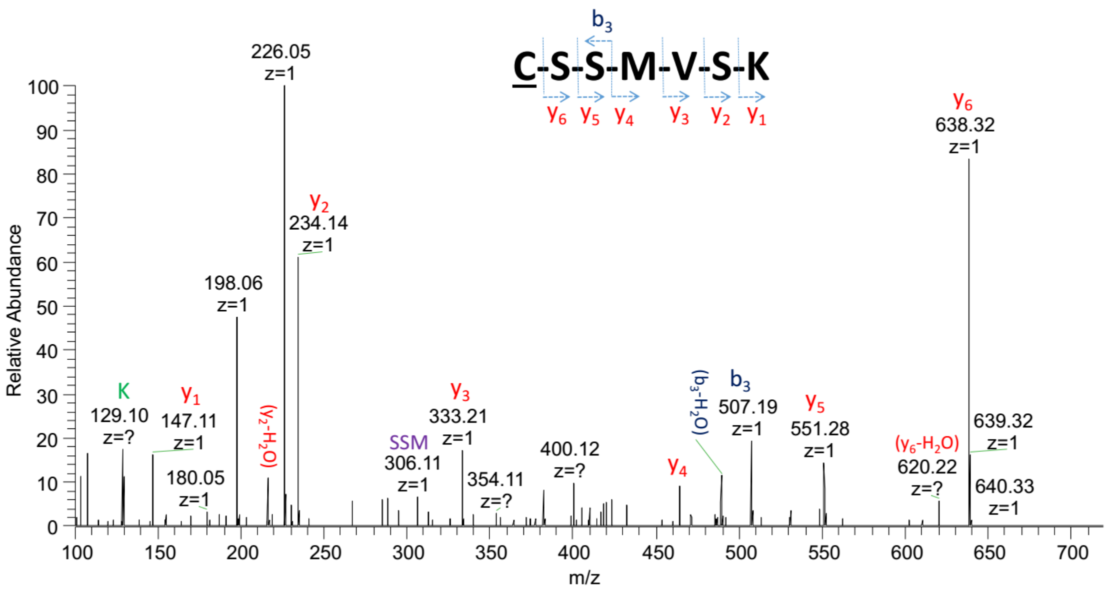

Figure S41. MS/MS spectrum of the *m/z* 485.72 doubly charged ion of the tryptic peptide **C**SSMVSKL from GFP containing a modification at the N-terminal cysteine residue. The underscore relates to the modified amino acid.

9.3.4 Reaction of Cys-GFP with CPO-PEG

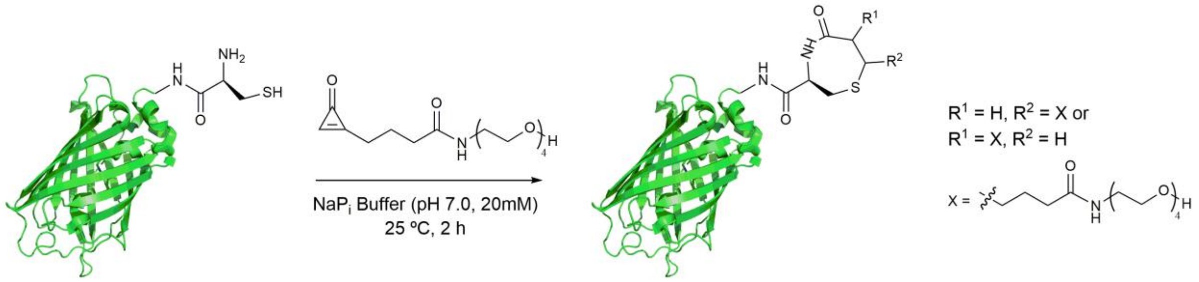

A mixture of 8 µL of Cys-GFP stock solution (30 µM in 20 mM pH 7 NaP$_i$ buffer containing 16.5 mM DTT), 12 µL of 20 mM pH 7 NaP$_i$ buffer, and 1.5 µL of cyclopropenone reagent **CPO-PEG** (16 mM in CH_3CN, 100 equiv.) was shaken in an 1.5 mL microcentrifuge tube at 25 °C for 2 h. The modified product was characterized by LC-MS analysis and complete conversion to the expected product was observed (calculated mass, 27514 Da; observed mass, 27516 Da).

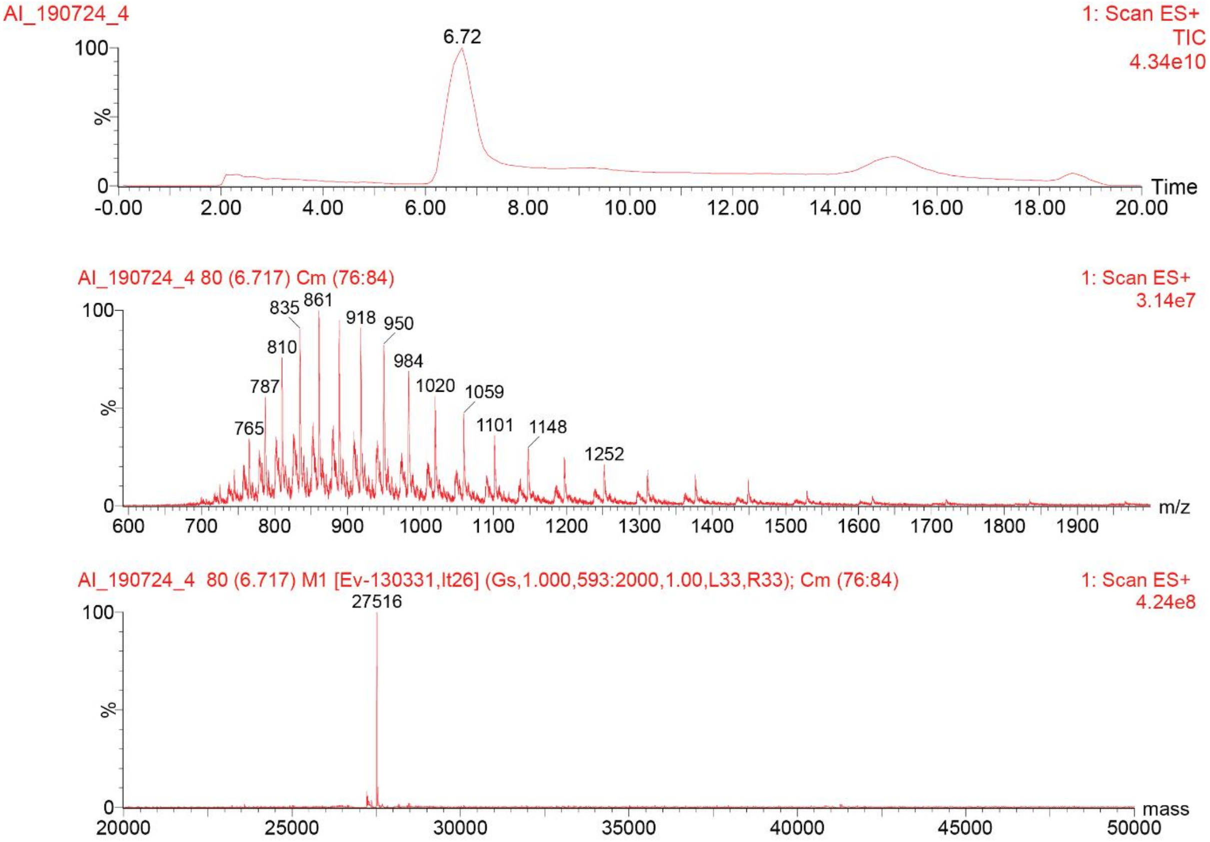

Figure S42. The total ion chromatogram, combined ion series and deconvoluted mass spectrum of the reaction between **Cys-GFP** and **CPO-PEG** after 2 h at 25 ºC.

9.3.5 Reaction of Cys-GFP with CPO-EDANS

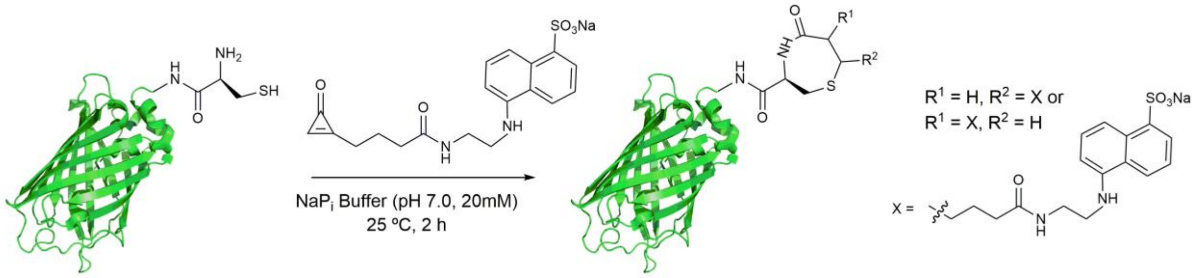

A mixture of 8 µL of Cys-GFP stock solution (30 µM in 20 mM pH 7 NaP$_i$ buffer containing 16.5 mM DTT), 12 µL of 20 mM pH 7 NaP$_i$ buffer, and 1.5 µL of cyclopropenone reagent **CPO-EDANS** (16 mM in DMF, 100 equiv.) was shaken in an 1.5 mL microcentrifuge tube at 25 °C for 2 h. The modified product was characterized by LC-MS analysis and 70% conversion to the expected product was observed (calculated mass for [M-Na$^+$], 27586 Da; observed mass, 27589 Da).

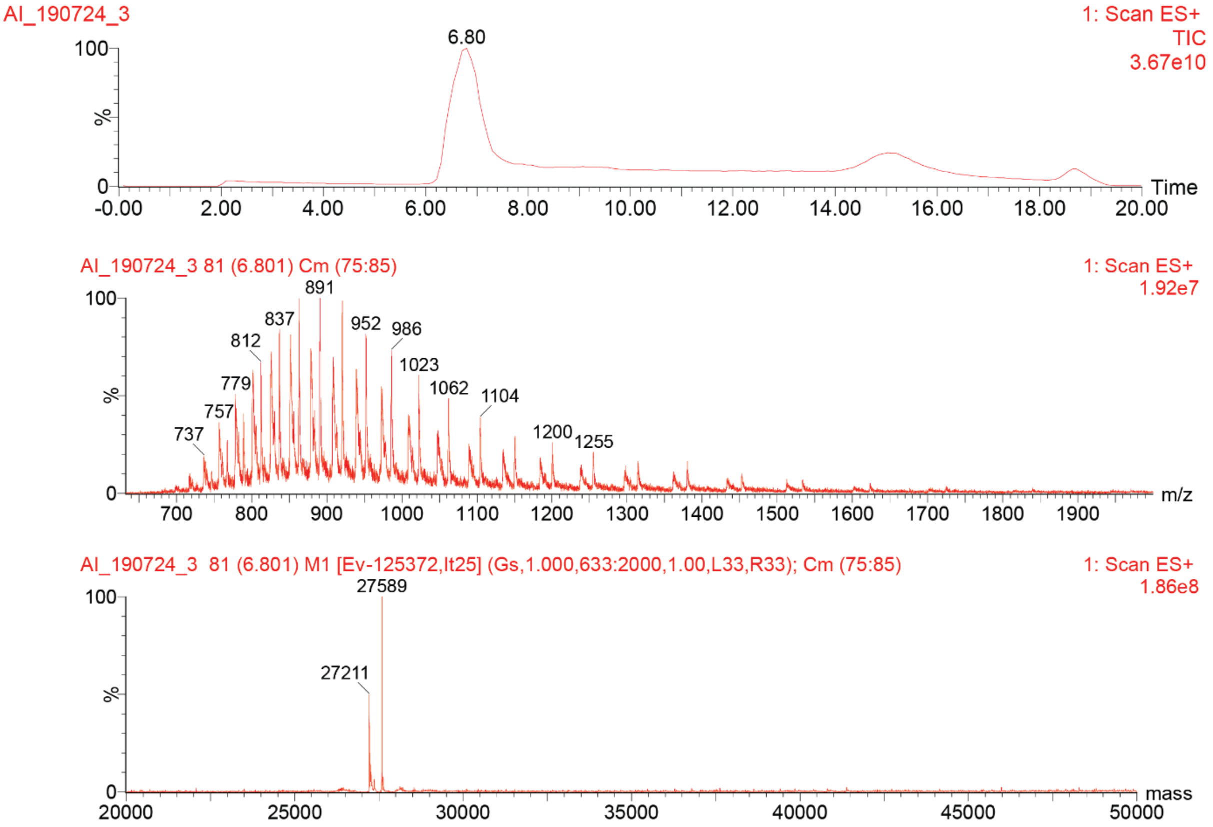

Figure S43. The total ion chromatogram, combined ion series and deconvoluted mass spectrum of the reaction between **Cys-GFP** and **CPO-EDANS** after 2 h at 25 °C.

9.3.6 Reaction of Cys-GFP with *N*-methylmaleimide

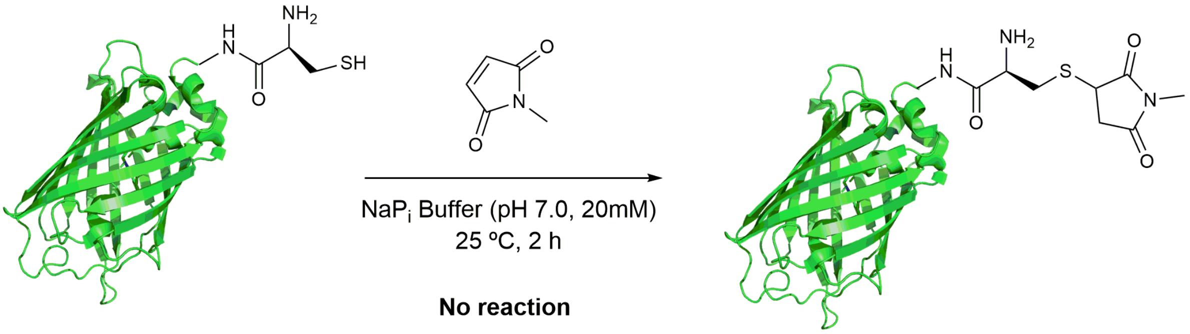

A mixture of 8 µL of Cys-GFP stock solution (30 µM in 20 mM pH 7 NaP$_i$ buffer containing 16.5 mM DTT), 12 µL of 20 mM pH 7 NaP$_i$ buffer, and 1.5 µL of ***N*-methylmaleimide** (16 mM in CH_3CN, 100 equiv.) was shaken in an 1.5 mL microcentrifuge tube at 25 ºC for 2 h. The modified product was characterized by LC-MS analysis and no conversion to the product was observed (calculated mass, 27310 Da; observed mass, 27206 Da (starting protein only)).

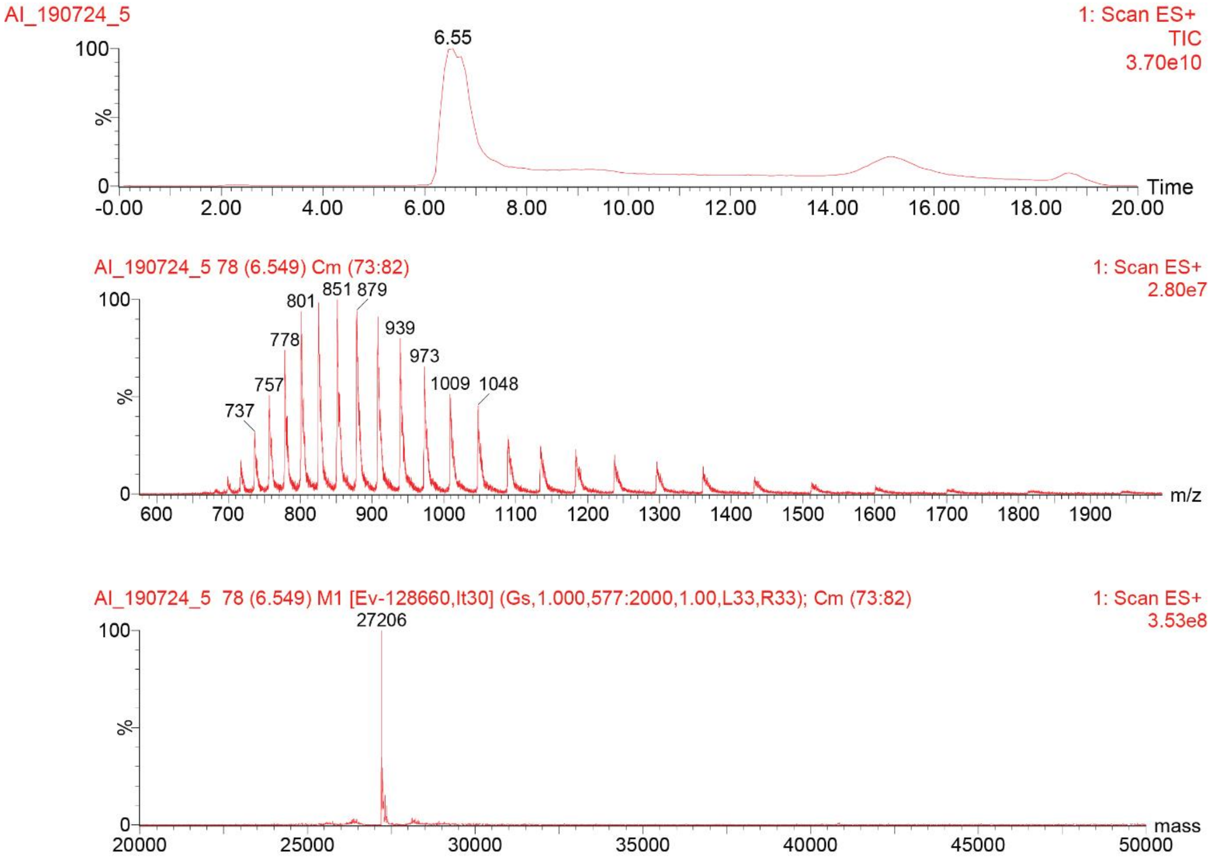

Figure S44. The total ion chromatogram, combined ion series and deconvoluted mass spectrum of the reaction between Cys-GFP and *N*-methylmaleimide after 2 h at 25 °C. No reaction was observed.

9.3.7 Reaction of Cys-GFP with CPO-biotin

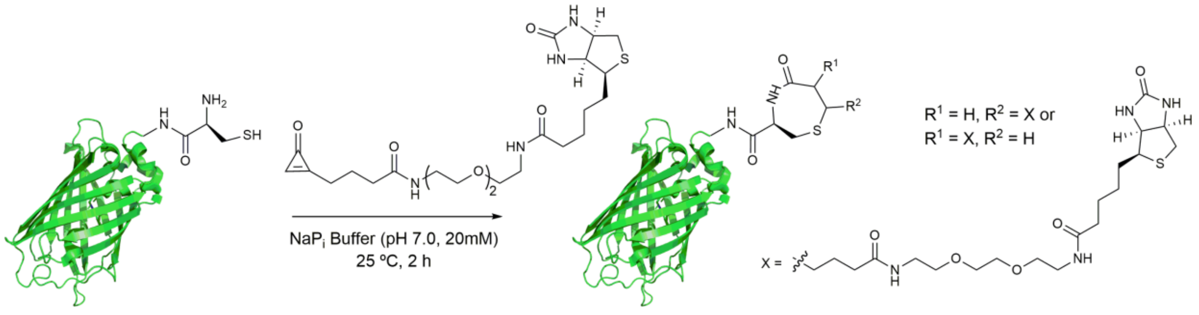

A mixture of 3 µL of Cys-GFP stock solution (25 µM in 20 mM pH 7 NaP$_i$ buffer), 10.65 µL of 20 mM pH 7 NaP$_i$ buffer, 0.6 µL of DTT stock solution (62.5 mM in MQ-H_2O, 500 equiv.), and 0.75 µL of cyclopropenone reagent **CPO-biotin** (10 mM in CH_3CN, 100 equiv., prepared as described on page S69) was shaken in an 1.5 mL microcentrifuge tube at 25 °C for 2 h. The modified product was characterized by LC-MS analysis and complete conversion to the product was observed (calculated mass, 27695 Da; observed mass, 27702 Da).

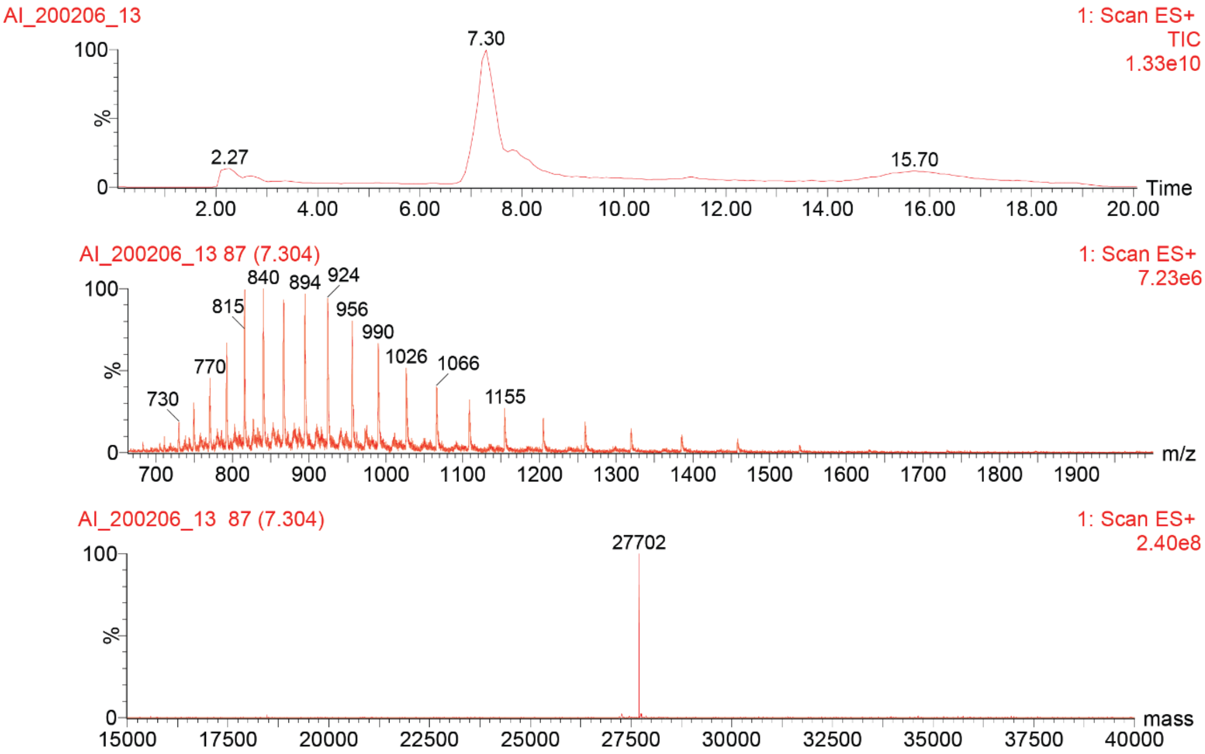

Figure S45. The total ion chromatogram, combined ion series and deconvoluted mass spectrum of the reaction between **Cys-GFP** and **CPO-biotin** after 2 h at 25 °C.

9.3.8 Reaction of Cys-GFP with Maleimide-Biotin

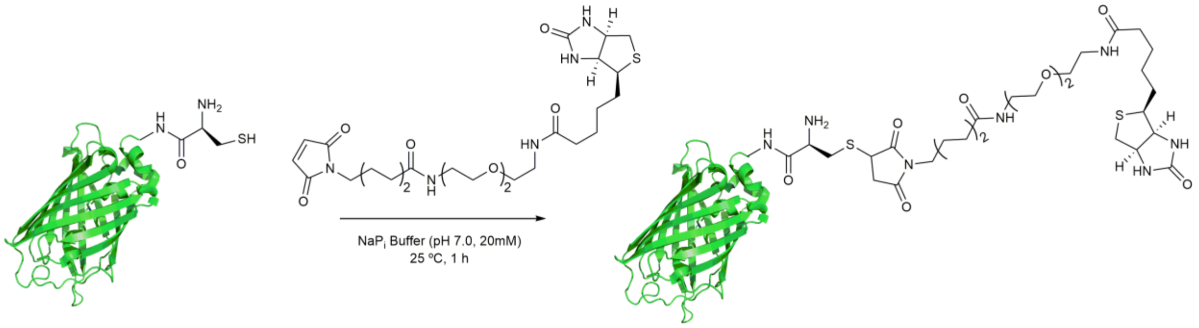

A mixture of 3 µL of Cys-GFP stock solution (25 µM in 20 mM pH 7 NaP$_i$ buffer), 10.65 µL of 20 mM pH 7 NaP$_i$ buffer, 0.6 µL of DTT stock solution (62.5 mM in MQ-H_2O, 500 equiv.), and 0.75 µL of cyclopropenone reagent **MI-Biotin** (10 mM in CH_3CN, 100 equiv., prepared as described on page S70) was shaken in an 1.5 mL microcentrifuge tube at 25 °C for 2 h. The modified product was characterized by LC-MS analysis and 13% conversion to the product was observed (calculated mass, 27766 Da; observed mass, 27771 Da).

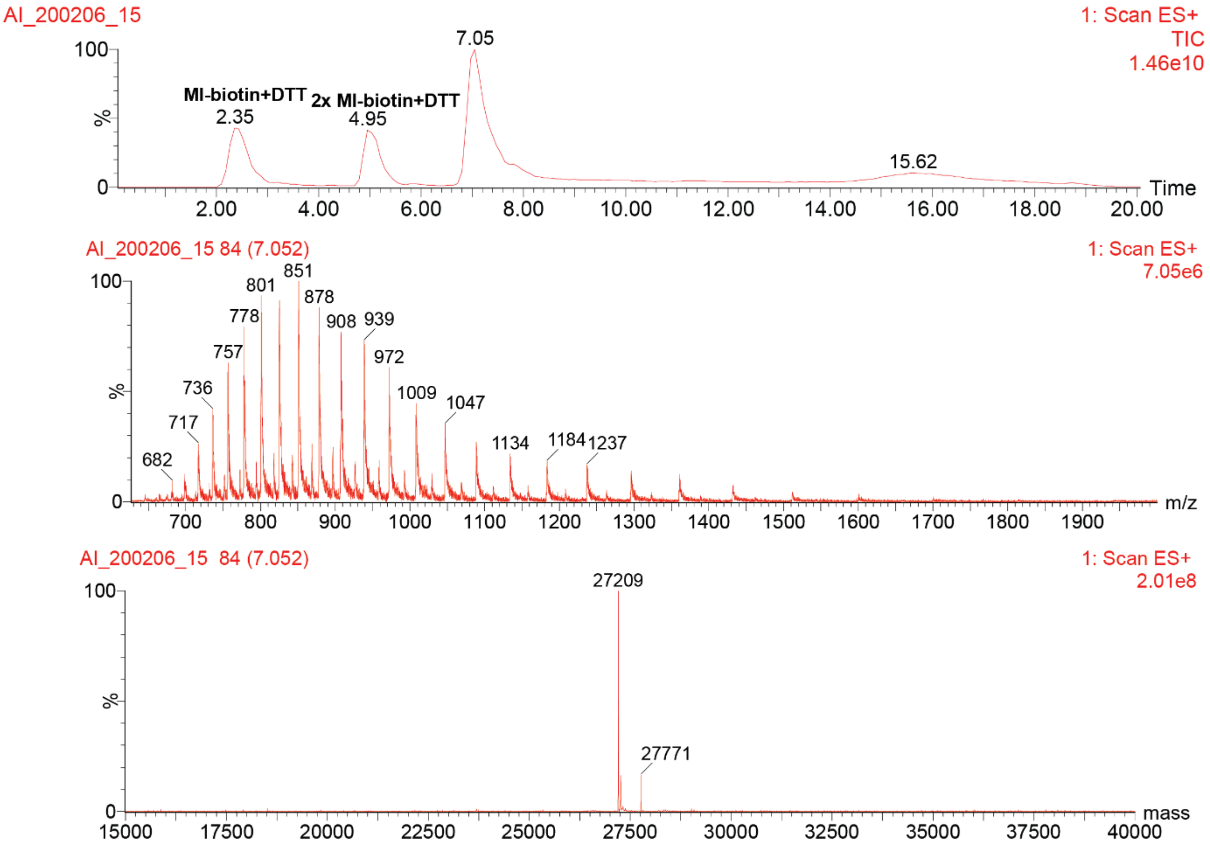

Figure S46. The total ion chromatogram, combined ion series and deconvoluted mass spectrum of the reaction between **Cys-GFP** and **MI-Biotin** after 2 h at 25 °C.

9.3.9 Selective modification of Cys-GFP in a mixture of proteins

Preparation of **CPO-biotin** probe:

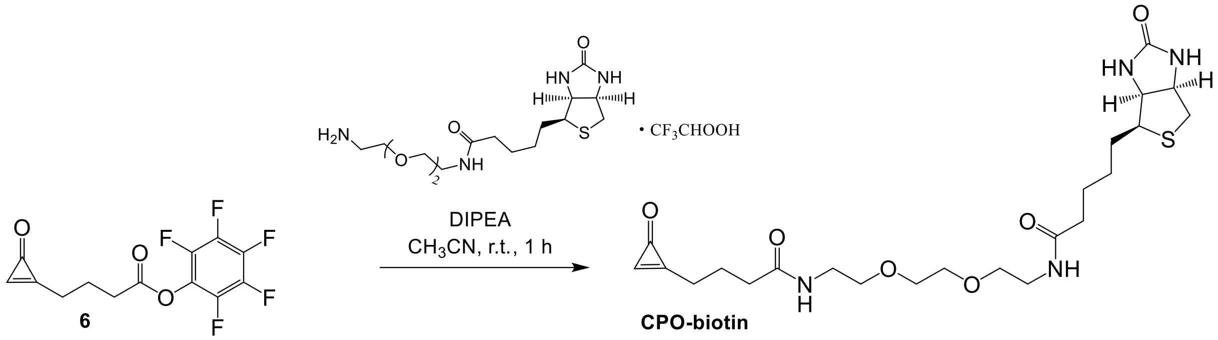

To a glass vial containing 28 µL of CH_3CN, **CPO-PFP** (2.4 mg, 7.84 µmol, 1 equiv.), DIPEA (1.3 µl 7.5 µmol, 1 equiv.) and N-biotinyl-3,6-dioxaoctane-1,8-diamine trifluoroacetate salt solution (167 µL of 25 mg/mL reagent in DMSO, 8.55 µmol, 1.1 equiv.) were added, and the reaction mixture was incubated for 1 h at 25 °C. Next, 1 µL aliquot of the reaction mixture was mixed with 39 µL of CH_3CN, the resulting sample was analyzed by LC–MS, and complete conversion of **CPO-PFP** to **CPO-biotin** probe was observed (**Figure S47).** The resulting **CPO-biotin** solution was diluted to 5 mM with CH_3CN (assuming quantitative conversion) and used for protein selectivity experiment without further purification.

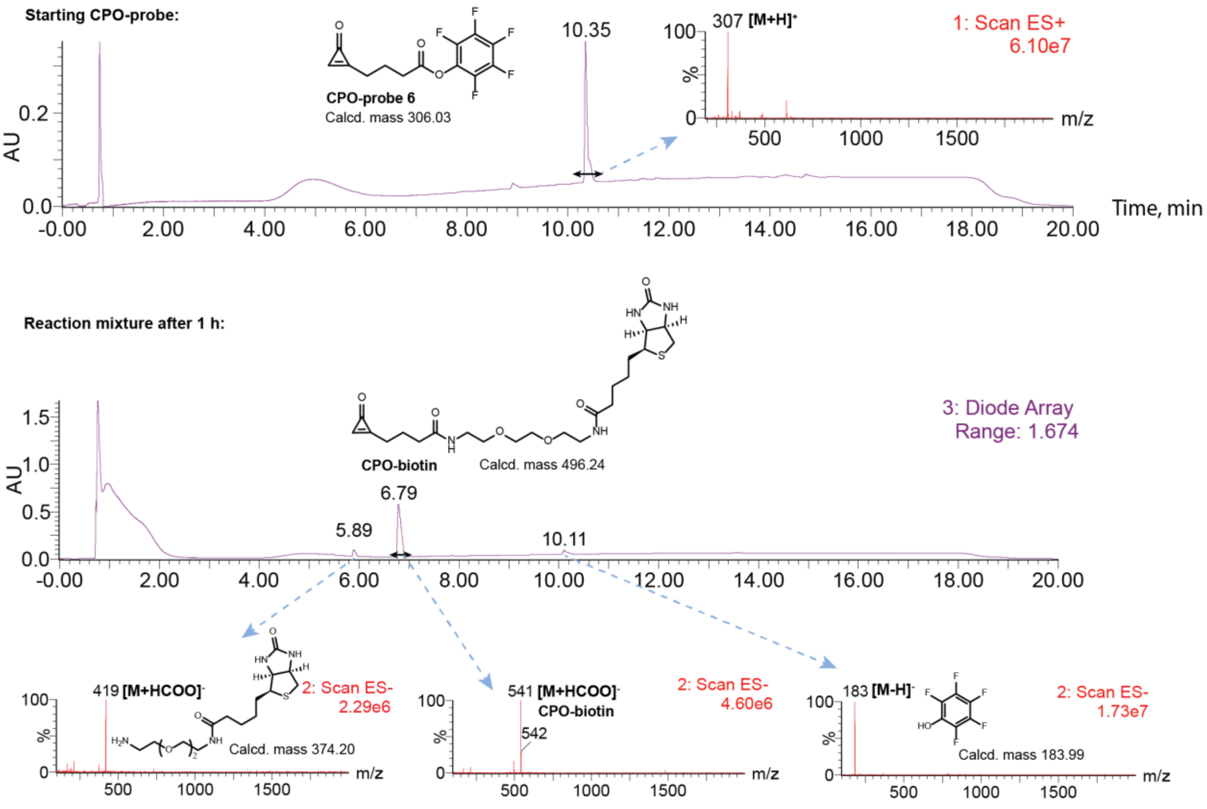

Figure S47. HPLC and MS analysis of reactions between **CPO-PFP** and N-biotinyl-3,6-dioxaoctane-1,8-diamine trifluoroacetate salt solution. The reaction was complete after incubation for 1 h at 25 °C.

Preparation of maleimide-biotin (**MI-biotin**) probe:

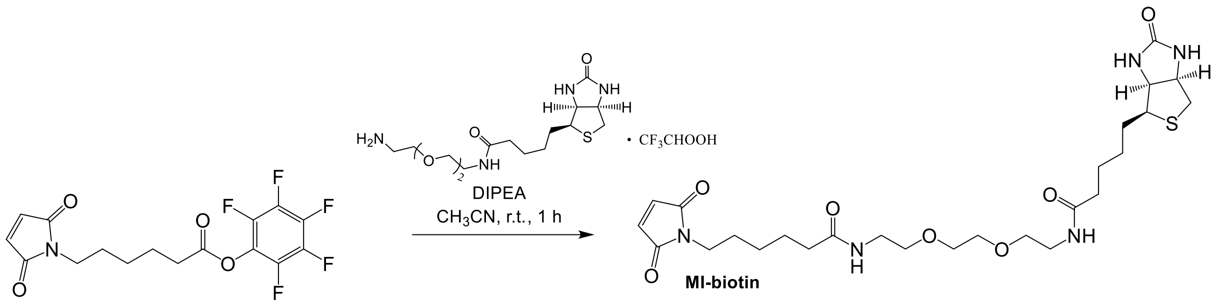

To a glass vial containing 32 µL of CH_3CN, perfluorophenyl 6-(2,5-dioxo-2,5-dihydro-1H-pyrrol-1-yl)hexanoate (3.3 mg, 8.75 µmol, 1 equiv.), DIPEA (1.5 µL, 8.61 µmol, 1 equiv.) and N-biotinyl-3,6-dioxaoctane-1,8-diamine trifluoroacetate salt solution (188 µL of 25 mg/mL reagent in DMSO, 9.62 µmol, 1.1 equiv.) were added, and the reaction mixture was incubated for 1 h at 25 °C. Next, 1 µL aliquot of the reaction mixture was mixed with 39 µL of CH_3CN, the resulting sample was analysed by LC–MS, and complete conversion of perfluorophenyl ester to maleimide-biotin (**MI-biotin**) probe was observed (**Figure S48).** The resulting maleimide-biotin solution was diluted to 5 mM with CH_3CN (assuming quantitative conversion) and used for protein selectivity experiment without further purification.

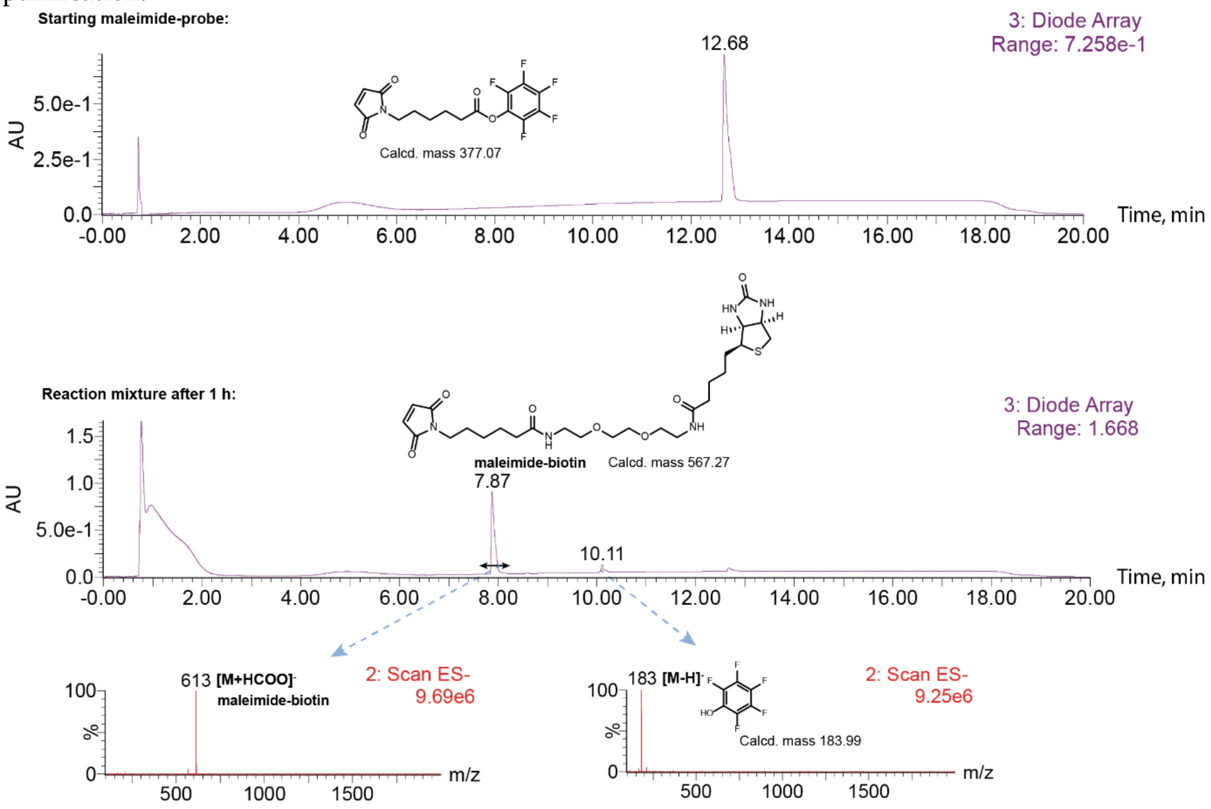

Figure S48. HPLC and MS analysis of reactions between perfluorophenyl 6-(2,5-dioxo-2,5-dihydro-1H-pyrrol-1-yl)hexanoate and N-biotinyl-3,6-dioxaoctane-1,8-diamine trifluoroacetate salt solution. The reaction was complete after incubation for 1 h at 25 °C.

Preparation of protein stocks:

To a centrifuge tube containing 100 µL of 30 µM **Cys-GFP** protein stock solution in Tris HCl buffer (20 mM, pH 7.5), 10 equiv. of TCEP (1 µL of 30 mM stock in MQ H_2O) was added, and the reaction mixed for 2 h at 25 ºC. The resulting sample underwent buffer exchange using an Amicon® Ultra 3K 0.5 mL centrifugal filter device (*Merck*) by centrifugation (3 × 15 min) using NaP_i buffer (pH 7, 20 mM). Next, protein concentration was assessed by measuring absorbance at 280 nm and was determined to be 28 µM. The freshly reduced and buffer-exchanged protein stock solution was then used for protein selectivity experiment.

C2Am, **AnxV** and Nanobody **DesAB-HET** protein stocks underwent buffer exchange using an Amicon® Ultra 3K 0.5 mL centrifugal filter device (*Merck*) by centrifugation (3 × 15 min) using NaP_i buffer (pH 7, 20 mM). Next, protein concentration was assessed by measuring absorbance at 280 nm and was determined to be 55 µM (**C2Am**), 49 µM (**AnxV**) and 48 µM (**DesAB-HET)**. The protein stock solutions were then used for protein selectivity experiment.

Protein modification with CPO-biotin or maleimide-biotin probes:

To a centrifuge tube containing 13 µL of NaP_i buffer (20 mM, pH 7.0), 4.5 µL of a stock solution of **Cys-GFP** (28 µM, final concentration 5 µM), 2.6 µL of a stock solution of **AnxV** (49 µM, final concentration 5 µM), 2.6 µL of a stock solution of **DesAB-HET** (48 µM, final concentration 5 µM), 2.3 µL of a stock solution of C2Am (55 µM, final concentration 5 µM) were added and the resulting mixture was vortexed for 5 seconds. Afterwards, 50 equiv. (relative to **Cys-GFP**) of **CPO-biotin** or **MI-biotin** probe (1.25 µl of 5 mM stock solution) was added to the solution, and the reaction mixed for 1 h at 25 °C. In a control experiment, 1.25 µl of acetonitrile was added instead of the probes. Then, a 10 µL aliquot was analysed by LC–MS and the conversion for each protein was analysed (**Figure S50** and **Figure S51**).

Protein modification with CPO-biotin or maleimide-biotin probes in the presence of DTT:

To a centrifuge tube containing 12 µL of NaP_i buffer (20 mM, pH 7.0), 4.5 µL of a stock solution of Cys-GFP (28 µM, final concentration 5 µM), 2.6 µL of a stock solution of **AnxV** (49 µM, final concentration 5 µM), 2.6 µL of a stock solution of **DesAB-HET** (48 µM, final concentration 5 µM), 2.3 µL of a stock solution of **C2Am** (55 µM, final concentration 5 µM) were added and the resulting mixture was vortexed for 5 seconds. Afterwards, 500 equiv. (relative to **Cys-GFP**) of DTT (1 µl of 62.5 mM stock solution in MQ H_2O) and 50 equiv. (relative to **Cys-GFP**) of **CPO-biotin** or **MI-biotin** probe (1.25 µl of 5 mM stock solution) were added to the solution, and the reaction mixed for 1 h at 25 °C. In a control experiment, 1.25 µl of acetonitrile was added instead of the probes. Then, a 10 µL aliquot was analysed by LC–MS and the conversion for each protein was analysed (**Figure S52** and **Figure S53**).

Table S3. Calculated masses for **Cys-GFP**, **C2Am**, **AnxV** and **DesAB-HET** proteins, and their conjugates with **CPO-biotin** or **MI-biotin**.

	Calculates mass		
Protein	Unmodified	Conjugate with **CPO-biotin**	Conjugate with **MI-biotin**
**DesAB-HET**	17391 Da	17887 Da	17958 Da
**Cys-GFP**	27199 Da	27695 Da	27766 Da
**C2Am**	16222 Da	16718 Da	16789 Da
**AnxV**	35805 Da	36301 Da	36372 Da

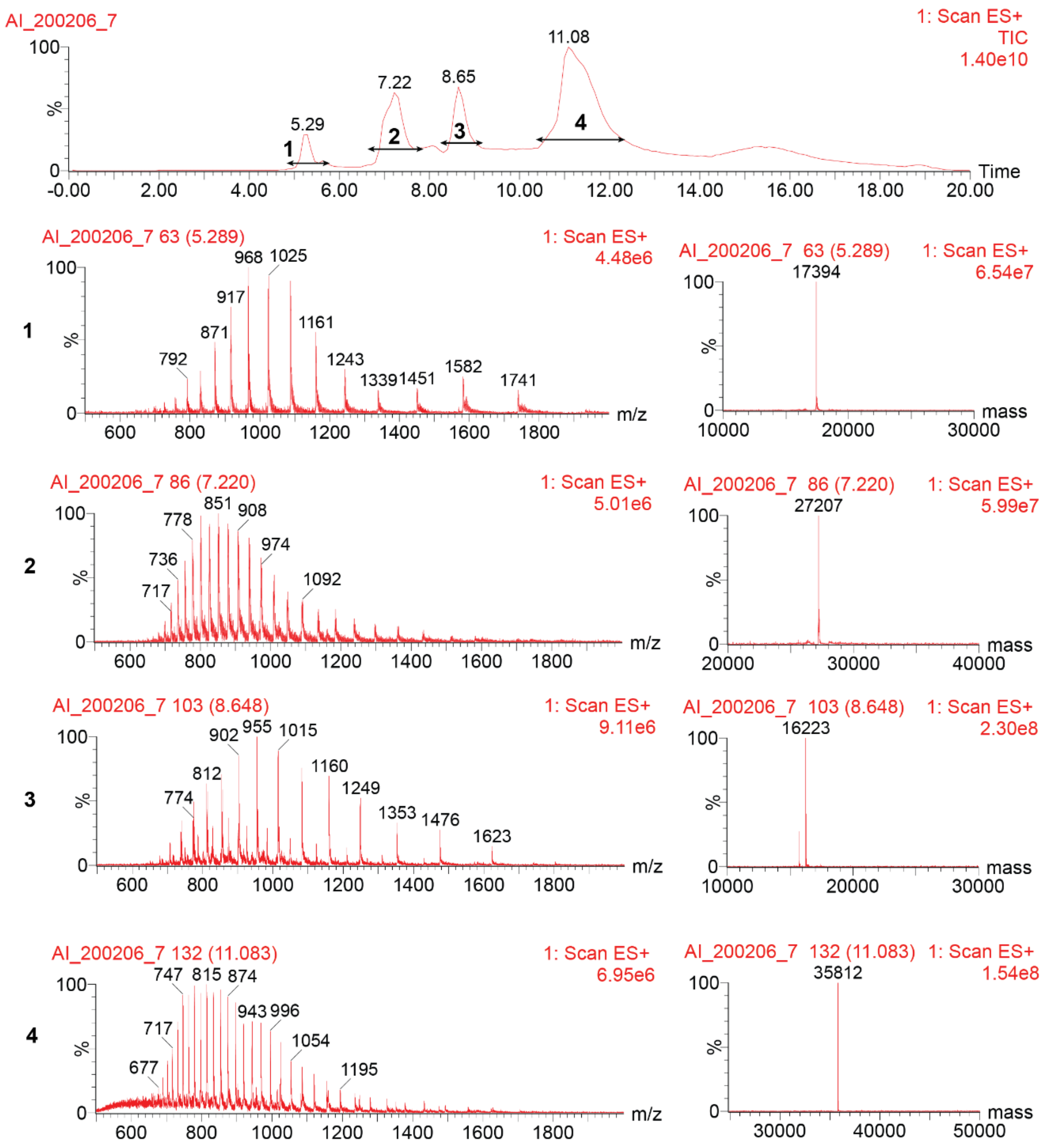

Figure S49. LC–MS trace, combined ion series and deconvoluted mass spectrum of: (**1**) **DesAB-HET**; (**2**) **Cys-GFP**; (**3**) **C2Am**; and (**4**) **AnxV** from a protein mixture after incubation for 1 h at 25 °C.

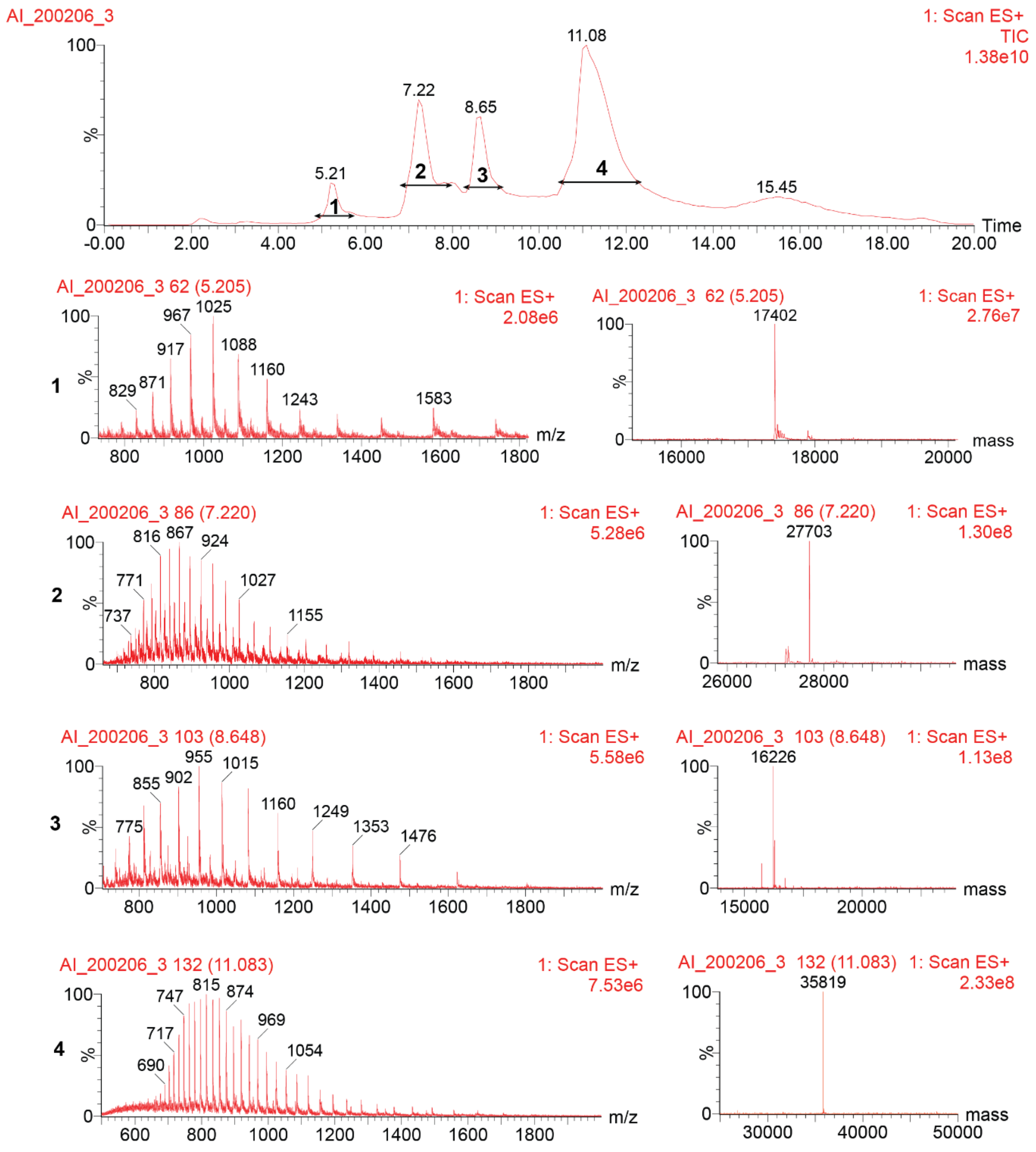

Figure S50. LC–MS trace, combined ion series and deconvoluted mass spectrum of: (**1**) **DesAB-HET**; (**2**) **Cys-GFP**; (**3**) **C2Am**; and (**4**) **AnxV** from a protein mixture after incubation with **CPO-biotin** (50 equiv) for 1 h at 25 °C. **Cys-GFP** protein is modified (>90%) while the other proteins remain unchanged.

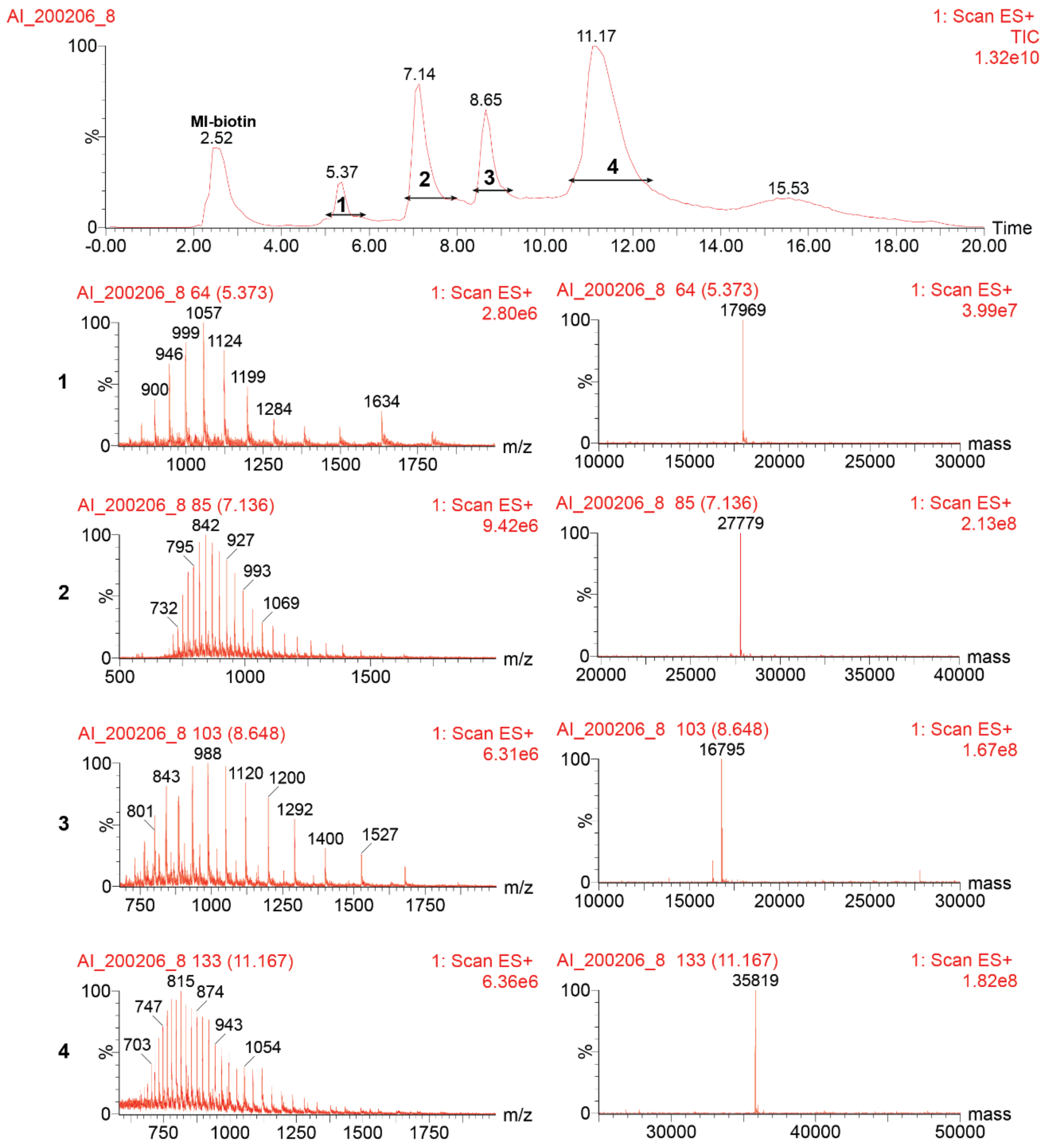

Figure S51. LC–MS trace, combined ion series and deconvoluted mass spectrum of: (**1**) **DesAB-HET**; (**2**) **Cys-GFP**; (**3**) **C2Am**; and (**4**) **AnxV** from a protein mixture after incubation with **MI-biotin** (50 equiv) for 1 h at 25 °C. As expected, full conjugation is observed for **DesAB-HET**, **Cys-GFP**, and **C2Am** proteins, while **AnxV** protein remains unchanged.

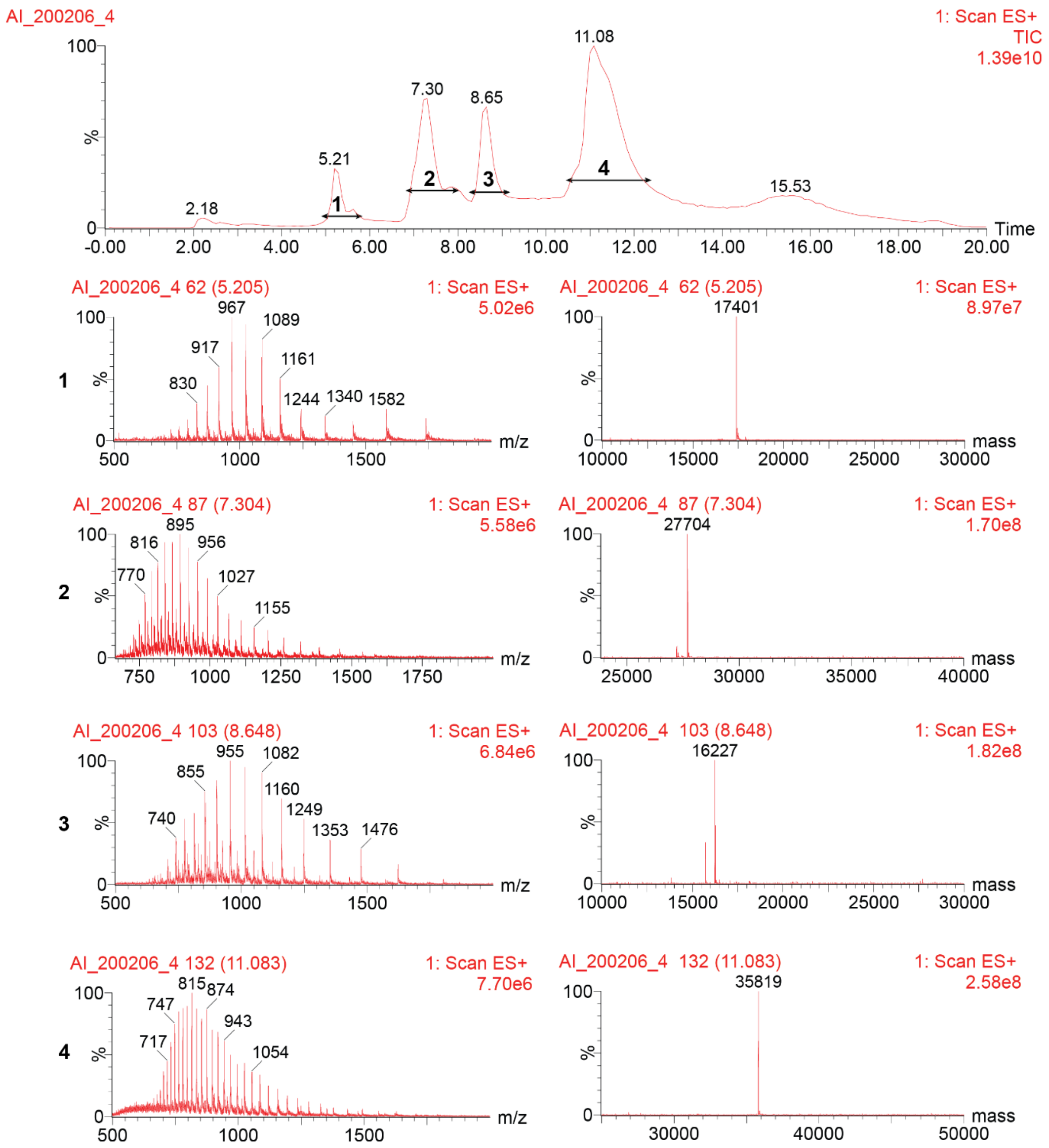

Figure S52. LC–MS trace, combined ion series and deconvoluted mass spectrum of: (**1**) **DesAB-HET**; (**2**) **Cys-GFP**; (**3**) **C2Am**; and (**4**) **AnxV** from a protein mixture after incubation with **CPO-biotin** (50 equiv) in the presence of 2.5 mM DTT for 1 h at 25 °C. **Cys-GFP** is modified (>95%) while the other proteins remain unchanged.

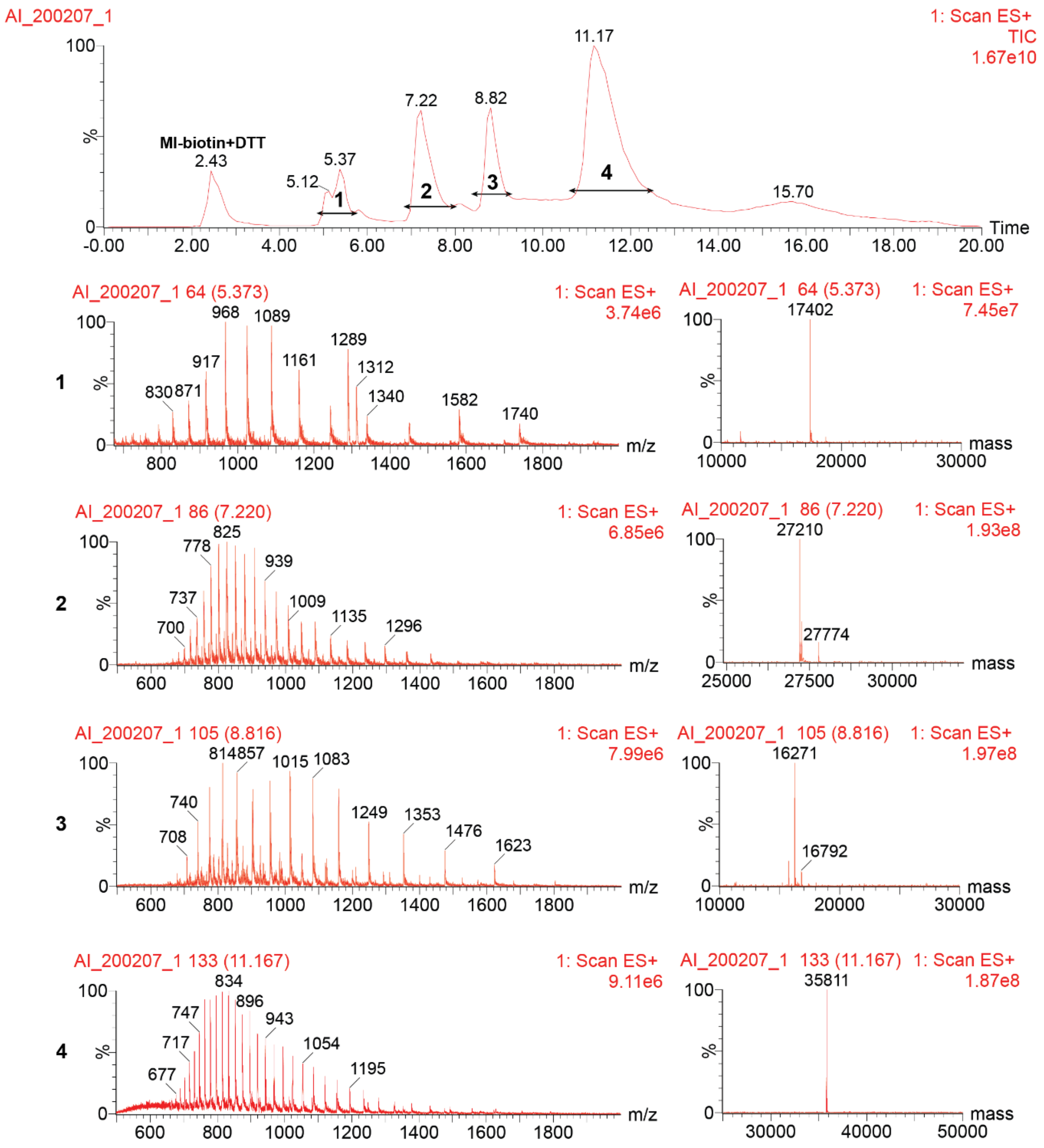

Figure S53. LC–MS trace, combined ion series and deconvoluted mass spectrum of: (**1**) **DesAB-HET**; (**2**) **Cys-GFP**; (**3**) **C2Am**; and (**4**) **AnxV** from a protein mixture after incubation with **MI-biotin** (50 equiv) in the presence of 2.5 mM DTT for 1 h at 25 °C. **DesAB-HET** and **AnxV** remain unreacted, while for **Cys-GFP** and **C2Am** 10–15% modification is observed.

9.4 Reactions of 2×Cys-GFP

A solution of **2×Cys-GFP** (45 µM, 200 µL, 20 mM NaP_i, 4.5 mM DTT, pH 7) as prepared in Section 9.2.2 was treated with an aqueous solution of **CPO-PEG** (57 µL, 16 mM, 100 equiv) and buffer (43 µL, 20 mM NaP_i, pH 7) to give solution with final concentrations of: **2×Cys-GFP**: 30 µM; DTT: 3 mM; **CPO-PEG**: 3 mM; NaP_i : 20 mM (pH 7). The reaction mixture was shaken (500 rpm, 25 °C) for 2 h. Analysis of the reaction mixture by LC–MS showed the presence of a major species corresponding to the product of **2×Cys-GFP** with **CPO-PEG**, **2×Cys-GFP-1** (found: 27536 Da; theory: 27530 Da).

Small molecules were removed from 200 µL of the reaction mixture by passage through a Zeba column (500 µL, reaction mixture split between two filters) according to the manufacturer's instructions. The resulting solution was treated with **CAA-BN** (22 µL, 6 mM, 135 nmols, 22 equiv) and shaken (500 rpm, 25 °C) for 30 min. Analysis by LC–MS showed a major species after mass deconvolution corresponding to **2×Cys-GFP**+**CPO-PEG**+**CAA-BN**, **2×Cys-GFP-2** (found: 27802 Da, theory: 27794 Da).

9.5 Reactions of cys-nIL2

9.5.1 Preparation of nIL2-PEG

The following experiment was performed using a 80 µL aliquot with a protein concentration of 40 µM in PBS.

1. Reduction with TCEP and DTT

Treat an aliquot of **cys-nIL2** (80 µL, 40 µM, 3200 pmols) with TCEP (3 mM, 3.2 µL, 9600 pmols, 3 equiv) and DTT (1.06 µL, 150 mM, 160 nmols, 50 equiv) in PBS for 1 hour at 25 °C.

2. Perform conjugation with **CPO-PEG**

To the reduced solution of **cys-nIL2** (38 µM, 84 uL, 3200 pmols) add **CPO-PEG** (20 µL, 16 mM, 100 equiv, 320 nmols). Incubate at 25 °C for 2.5 h. *Note: Complete conjugation can be assayed by checking an aliquot of the mixture by LC–MS.*

3. Buffer exchange

Small molecules (TCEP, DTT, and **CPO-PEG**) were removed using an Amicon spin filter device (3 kDa cut-off) by repeated centrifugation (4×25 min, 10,000×g, 10 °C) with NaPi (20 mM, pH 7). The concentration of the protein was determined and **nIL2-PEG** used without further purification. The product was characterised by LC–MS (calculated: 12147 Da, found 12458 Da) and circular dichroism.

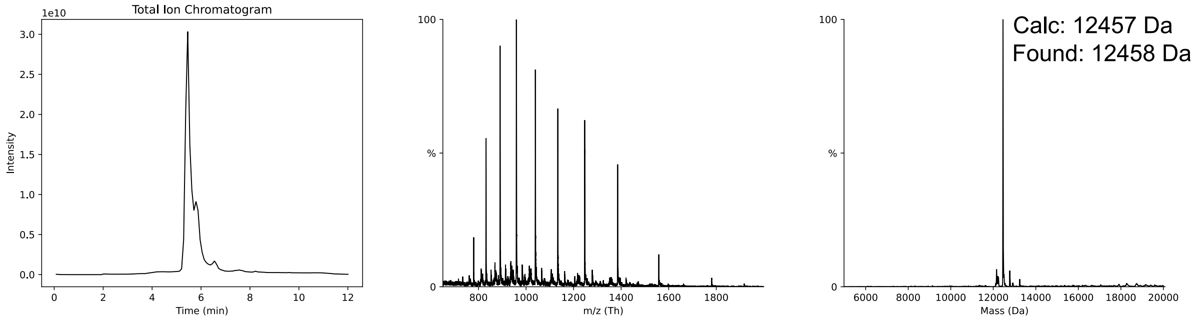

Figure S54. Characterisation of **nIL2-PEG** by LC–MS.

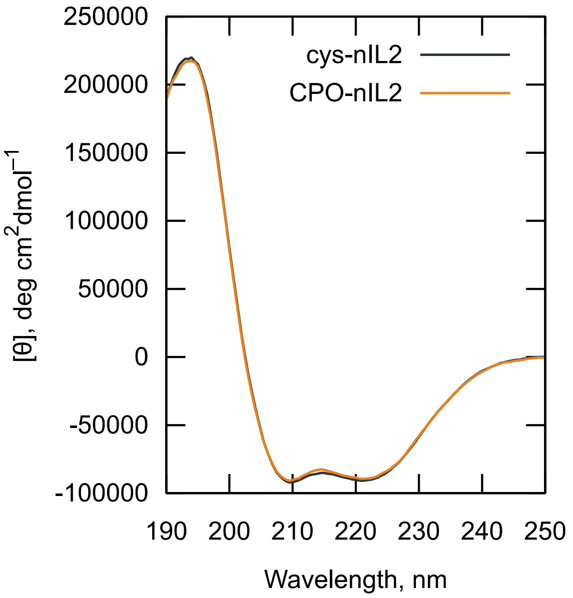

Figure S55. Characterisation of **cys-nIL2** and **nIL2-PEG** by circular dichroism spectroscopy.

9.5.2 Preparation of (nIL2)$_2$

Aliquots of **cys-nIL2** were prepared as described above. The following experiment was performed using a 290 µL aliquot with a protein concentration of 40 µM in PBS.

1. Buffer exchange

Exchange the buffer using an Amicon Ultra 0.5 mL centrifugal filter (MWCO 3 kDa, 4 tubes, 72.5 µL per tube, 3×25 min, 10,000×g, 10 °C) to NaP$_i$ (20 mM, pH 7).

2. Reduction with TCEP and DTT

Combine the four samples of **cys-nIL2** (376 µL, 30.5 µM, 11.6 nmols) with TCEP (20 mM, 0.58 µL, 11.6 nmols, 1 equiv) and DTT (3.86 µL, 150 mM, 580 nmols, 50 equiv) for 1 h at 25 °C. Final volume: ca. 380 µL, ca. 30.5 µM. *Note: Complete reduction can be assayed by checking an aliquot of the mixture by LC–MS.*

3. Add CPO-based reagents

Dilute the sample to 580 µL and split into two equal volumes. To each of the samples (290 µL, ca. 20 µM, 5.8 nmols, 20 mM NaP$_i$, 1 mM DTT, pH 7) add the respective CPO-based reagent: **CPO-DBCO** (11.6 µL, 50 mM, 100 equiv, 580 nmols) or **CPO-N$_3$** (11.6 uL, 50 mM, 580 nmols). The final concentration of the CPO-based probe is 2 mM and the sample contains ca. 4% DMSO.

4. Incubate for four hours at 25 °C (no shaking).
5. Remove small molecules

Split each reaction between two Amicon Ultra 0.5 mL centrifugal filters each (145 µL per tube) and remove small molecules by repeated centrifugation (6×25 min, 10,000×g, 10 °C) with fresh buffer (NaP$_i$, 20 mM, pH 7) to provide samples of **nIL2-N$_3$** and **nIL2-DBCO** (ca. 170 µL each, ca. 34 µM). *Note: Status of CPO-based modification can be assayed by checking an aliquot of the mixture by LC–MS.*

6. Perform coupling of **nIL2-N$_3$** and **nIL2-DBCO**

Combine both samples into one tube and dilute to a final volume of 380 µL (ca. 30 µM) in buffer (NaP_i, 20 mM, pH 7).

7. Incubate at 25 °C for 1 h.

Note: Reaction progress can be monitored by checking an aliquot of the mixture using LC–MS (completion was observed after 30 minutes).

8. Purification by size exclusion chromatography (SEC)

The sample was diluted to 1 mL and purified on an ÄKTA Prime using a Superdex 75 10/300 GL column using PBS as the eluent. The product was observed at an elution volume of ca. 14 mL. Fractions were checked for purity by SDS-PAGE and the appropriate fractions flash cooled with liquid nitrogen and stored at −80 °C.

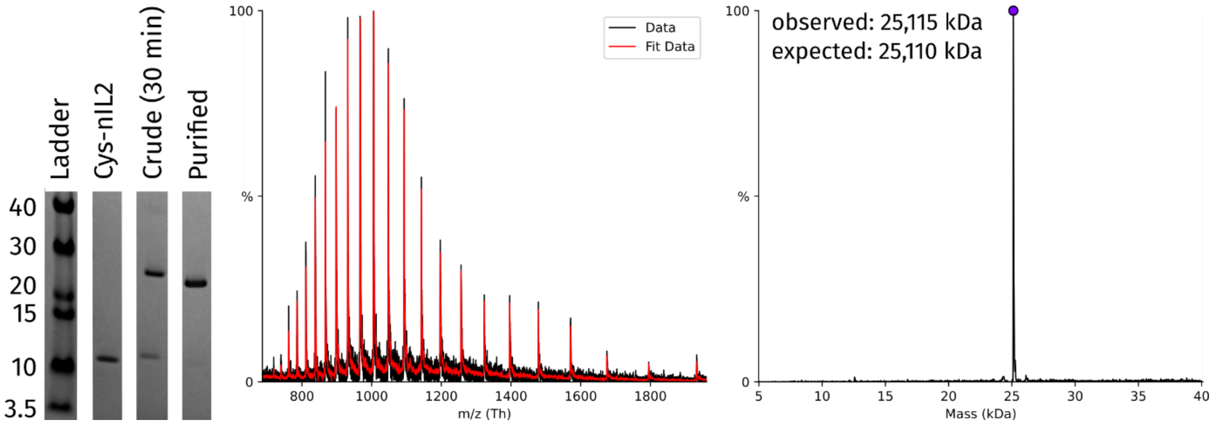

Figure S56. Characterisation of **(nIL2)$_2$**. Left: cropped gel. Right: LC–MS.

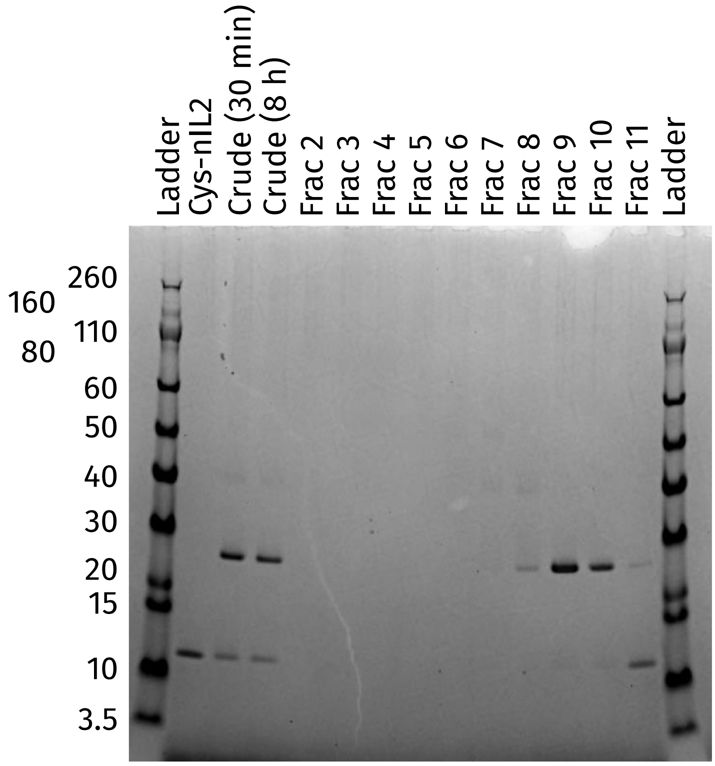

Figure S57. Full uncropped SDS-PAGE gel of crude reaction mixture and purified **(nIL2)$_2$**.

Parameters obtained from fitting BLI data (Section 9.1.4) are provided below:

sample	k_a	k_a error	k_d	k_d error	K_d (nm)	K_d error (nm)
**cys-nIL2**	658715	3446	0.00088	4.03E-06	13.4	0.0929
**nIL2-PEG**	741990	6626	0.00254	9.05E-06	34.3	0.33
**(nIL2)$_2$**	85940	616	2.46E-05	1.68E-06	2.86	0.197

9.6 Enzymatic digestion 2×Cys-GFP-1, 2×Cys-GFP-2, and nIL2-PEG followed by LC−MS/MS analysis

Samples of **1×Cys-GFP**, **2×Cys-GFP**, and **nIL2-PEG** were digested in an analogous manner to Section 9.1.2. In order to improve identification of the **CPO-PEG** modification, samples were analysed using CID.

Variable modifications of oxidation (M), deamidation (NQ) were applied to all samples. In the case of **2×Cys-GFP-1** and **nIL2-PEG**, a variable modification corresponding to **CPO-PEG** was applied. In the case of **2×Cys-GFP-2**, variable modifications corresponding to **CPO-PEG** and **CAA-BN** were applied. The peptide and fragment mass tolerances were set to 20 ppm and 0.8 Da, respectively. A significance threshold value of $p<0.05$ and a peptide cut-off score of 20 were also applied.

9.6.1 2×Cys-GFP-1

2×Cys-GFP contains four cysteine residues (C1, C52, C74, and C151). In theory, only C1 should be modified with **CPO-PEG.** Tryptic peptides containing both C1 and C151 were identified as having the **CPO-PEG** modification. In the case of C1, only the modified peptide was observed and the unmodified was not, ostensibly in agreement with complete modification of C1. In the case of C151, more peptides with higher scores were observed for the unmodified peptide, indicating a lower degree of modification.

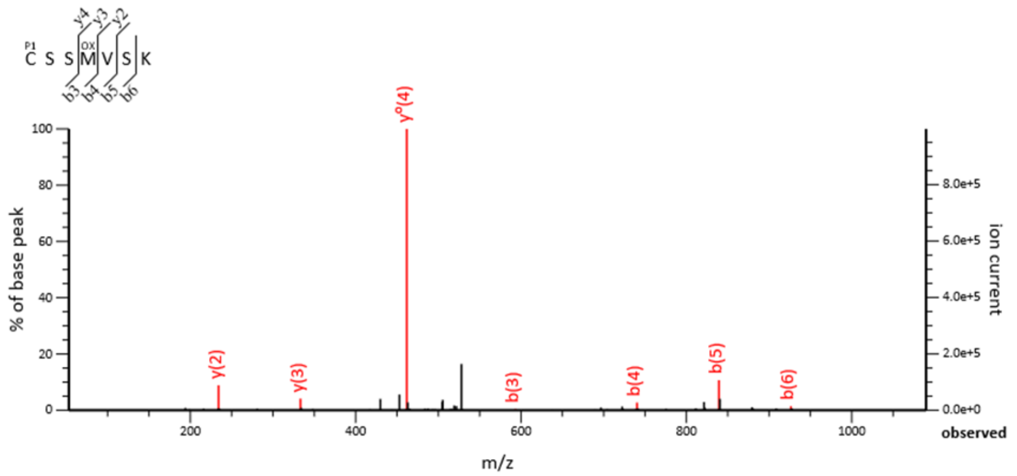

Figure S58. MS/MS spectrum of the doubly charged (*m/z* 536.74) GFP tryptic peptide CSSMVSK from sample **2×Cys-GFP-1** in which the underlined N-terminal cysteine residue is modified (**CPO-PEG**).

9.6.2 2×Cys-GFP-2

2×Cys-GFP contains four cysteine residues (C1, C52, C74, and C151). In theory, C1 should be modified by **CPO-PEG** and C151 should be modified by **CAA-BN**. Tryptic peptides containing C1 and C151 were identified with the **CPO-PEG** modification. In the case of C1, only the modified peptide

was observed and the unmodified was not, ostensibly in agreement with complete modification of this residue. In the case of C151, the most frequently identified peptides with the highest score featured the **CAA-BN** modification.

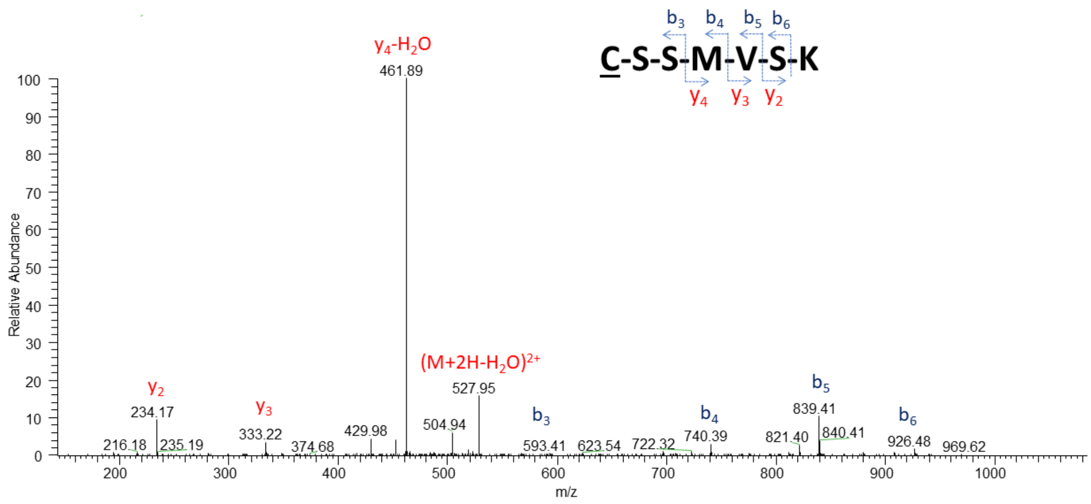

Figure S59. MS/MS spectrum of the doubly charged (*m/z* 536.74) GFP tryptic peptide C̲SSMVSK from sample **2×Cys-GFP-2** in which the underlined N-terminal cysteine residue is modified (**CPO-PEG**).

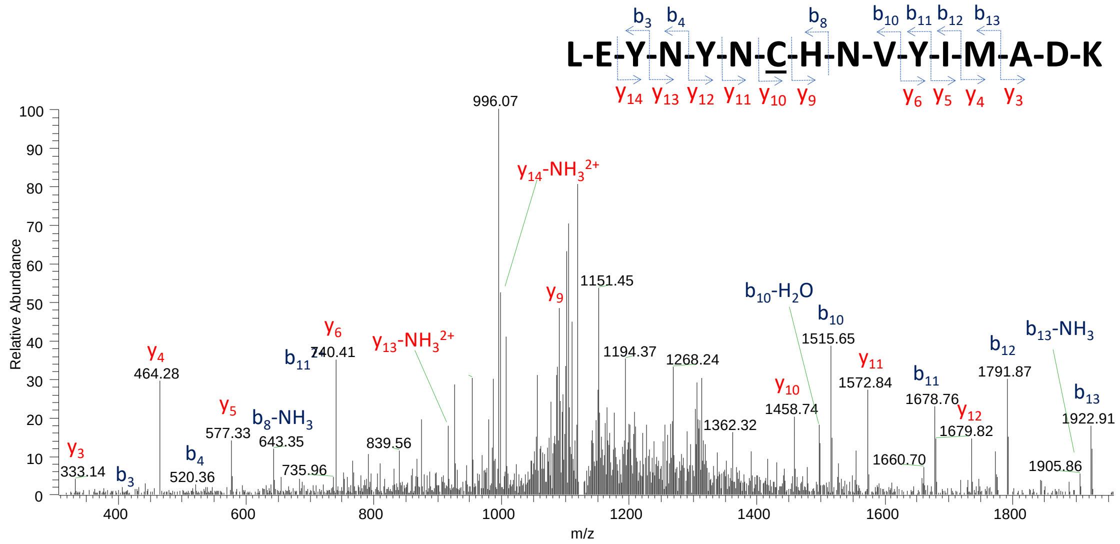

Figure S60. MS/MS spectrum of the doubly charged ion (*m/z* 1128.0) from the GFP tryptic peptide LEYNYNC̲HNVYIMADK from sample **2×Cys-GFP-2** in which the underlined cysteine residue is modified (**CAA-BN**).

9.6.3 nIL2-PEG

nIL2-PEG contains one cysteine residue (C1), in theory modified with **CPO-PEG.** Using the Mascot search procedure above, no cysteine-containing peptides were identified. This is ostensibly due to residues 6 to 8 (KKK) resulting in a missed trypsin cleavage. The resulting peptide, CSHMPKKKIQLHAEHALYDALMILNIVK (28 amino acids), may be too large or too highly charged to generate a reasonable match. No unmodified N-terminal peptide was identified either.

9.7 Control Reactions

9.7.1 Wild-type GFP (WT-GFP) with CPO-PEG (+/- DTT)

1. Prepare an aliquot of **WT-GFP** in PBS (125 µL, 20 µM, pH 7.4)
2. Split the sample into two aliquots (A and B) to assay reactivity in the presence (A) and absence (B) of DTT.

Each tube has a volume of 62.5 µL (20 µM **WT-GFP**, 1.25 nmols) in PBS (pH 7.4).

3. Add DTT to sample A

Add DTT (50 mM stock, 50 equiv, 1.25 µL, 62.5 nmols) to sample A.

4. Split both sample A and sample B into two equal aliquots. One (sample A1, B1) will be used to test reactivity with **CPO-PEG** and the other to assess stability of **WT-GFP** in the presence of MeCN (sample A2, B2).
5. Perform conjugation with **CPO-PEG** or MeCN control

To aliquots A1 and B1 add **CPO-PEG** (100 mM stock, 0.6 µL, 60 µmols, 100 equiv). To aliquots A2 and B2 add MeCN (0.6 µL).

6. Split each sample into two further aliquots to assay the reaction at two different temperatures (25 and 37 °C). Final sample information is provided in **Table S4**.
7. Incubate each sample for 24 hours. Samples were analysed by LC–MS after 2 and 24 h.

Table S4. Conditions for samples tested in Section Wild-type GFP (**WT-GFP**) with **CPO-PEG** (+/- DTT) 9.7.1.

Sample	DTT equiv	CPO-PEG equiv	Temp. (°C)
A1-25	50	100	25
A1-37	50	100	37
A2-25	50	0	25
A2-37	50	0	37
B1-25	0	100	25
B1-37	0	100	37
B2-25	0	0	25
B2-37	0	0	37

Outcome: No addition of **CPO-PEG** to **WT-GFP** was observed in the presence of DTT (samples A1–A2). A minor (12% intensity) peak was observed for addition of **CPO-PEG** and water to **WT-GFP** using the harshest conditions (37 °C, 24 h, no DTT). However, at 25 °C this peak was negligible.

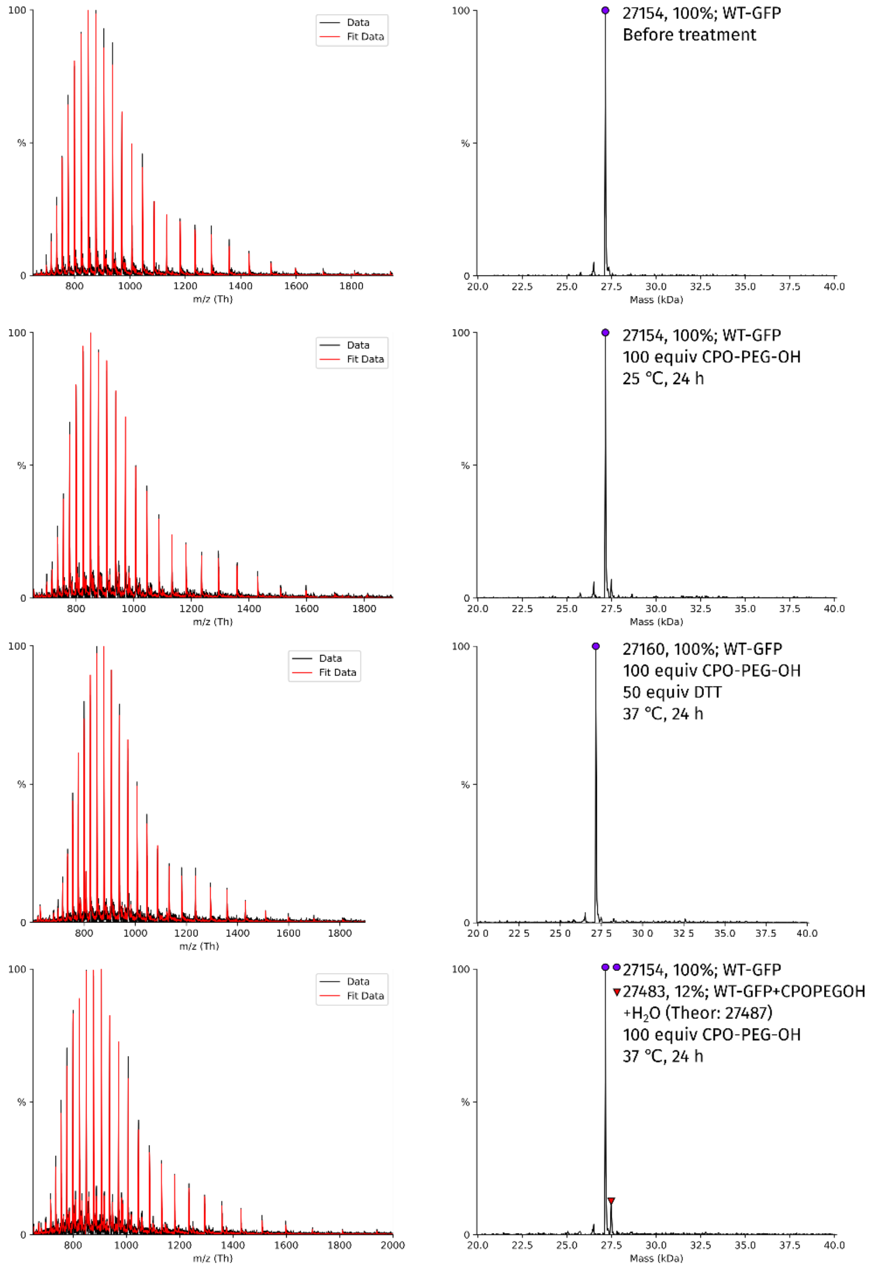

Figure S61 Ion series and deconvoluted mass spectra for selected samples obtained according to Section 9.7.1.

9.7.2 Lysozyme with CPO-PEG (+/- DTT)

An experiment identical to that described for testing the reactivity of **WT-GFP** (Section 9.7.1) was performed starting with an aliquot of commercial lysozyme in PBS (125 µL, 20 µM, pH 7.4). The protocol was otherwise reproduced identically.

Outcome: The outcome of the experiment was similar to that described for the control experiment with **WT-GFP**. A minor (21% total intensity) modification was observed with the harshest conditions (37 °C, 24 h, no DTT). However, by either adding DTT or decreasing the temperature to 25 °C, negligible change to the intact mass of lysozyme was observed.

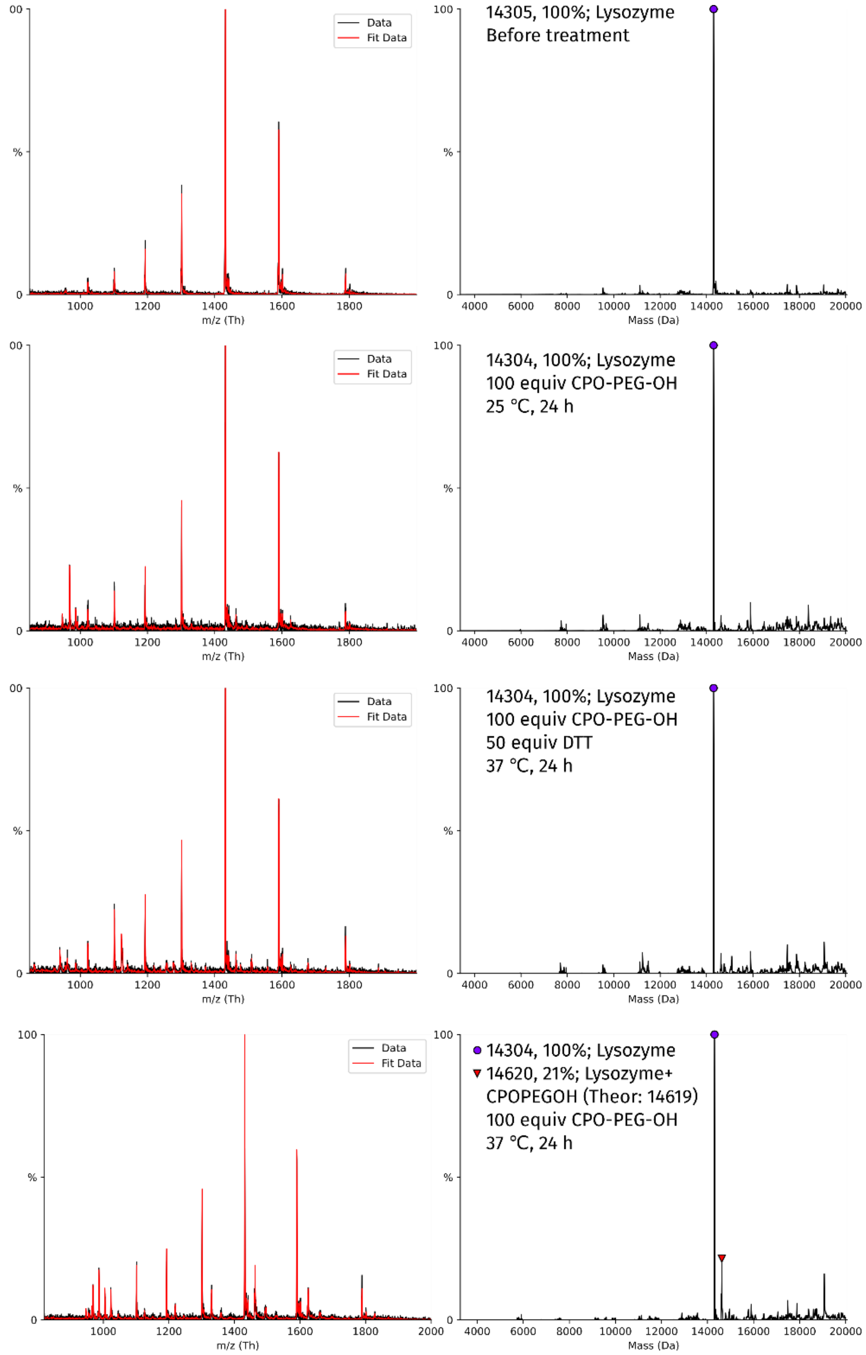

Figure S62. Ion series and deconvoluted mass spectra for selected samples obtained according to Section 9.7.2.

9.7.3 Attempt to prepare a dimer of cys-nIL2 using a bismaleimide reagent (+/- DTT)

1. Reduction of **cys-nIL2** with DTT

An aliquot of **cys-nIL2** (60 µL, 20 µM, 1.2 nmols) in PBS was treated with DTT (100 mM, 50 equiv, 60 nmols, 0.6 µL) for 1 hour at 25 °C. Complete reduction was checked by LC–MS.

2. Split the aliquot into four equal volumes (15 µL), denoted A, B, C, D. Aliquots A and B will be used to test the reaction in the presence of DTT. Aliquots C and D will be used to test the reaction in the absence of DTT.
3. Remove DTT from aliquot C

Perform buffer exchange on aliquot C using a Zeba spin filter (75 µL) into PBS (pH 7.4).

4. Perform conjugation with **BM-PEG$_3$**

Add **BM-PEG$_3$** (100 mM, MeCN, 50 equiv, 15 nmols, 0.15 µL) to tubes A and C. Incubate at 25 °C for 1 hour.

5. Remove excess **BM-PEG$_3$** from aliquots A and C; Remove DTT from aliquot D.

Perform buffer exchange on samples A, C, and D to remove small molecules using a Zeba spin filter (75 µL) into PBS (pH 7.4). This step is intended to remove **BM-PEG$_3$** from aliquots A and C, and to remove DTT from aliquot D.

6. Perform second protein–protein conjugation step

Combine aliquots A with B (DTT-containing aliquots), and C with D (DTT-free aliquots). Incubate for 2 h at 25 °C. Analyse the reaction mixture by LC–MS.

Outcome: In both cases (+/- DTT), small amounts of the anticipated product, **(nIL2)$_2$BM-PEG$_3$**, were observed. However, the major product corresponded to **cys-nIL2-BM-PEG$_3$**, with a single **BM-PEG$_3$** modification. These results may be attributed to one of the following factors: (i) removal of DTT leads to loss of **cys-nIL2** reactivity; (ii) quenching of **BM-PEG$_3$** by DTT; (iii) inefficient desalting of excess **BM-PEG$_3$**.

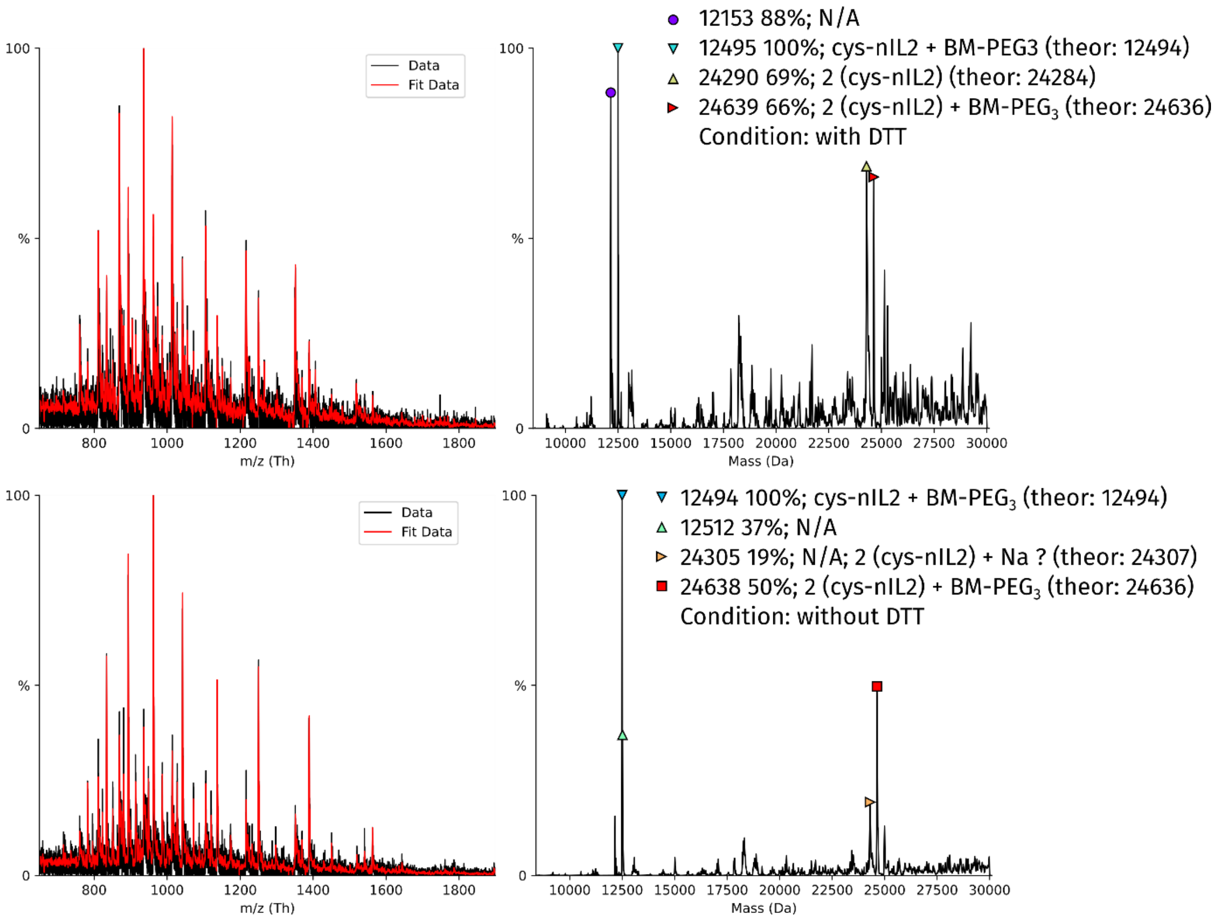

Figure S63. Ion series and deconvoluted mass spectra of products obtained corresponding to Section 9.7.3.

9.7.4 Reactivity of 2×Cys-GFP with NMM

A control was performed to assess the reactivity of **NMM** with **2×Cys-GFP**, which has two reactive cysteine residues. An aliquot of **2×Cys-GFP** (20 µL, 45 µM) in PBS (pH 7.4) containing 4.5 mM DTT was prepared. The sample was split into two equal aliquots and one aliquot (10 µL) was subject to buffer exchange (PBS, pH 7.4) using a zeba spin filter (75 µL) in order to remove DTT. To each aliquot was added a solution of **NMM** (0.45 µL, 100 mM stock, MeCN, 100 equiv). Both samples were incubated at 25 °C for 2 h then analysed by LC–MS. In both cases, peaks corresponding to the addition of two molecules of **NMM** were observed.

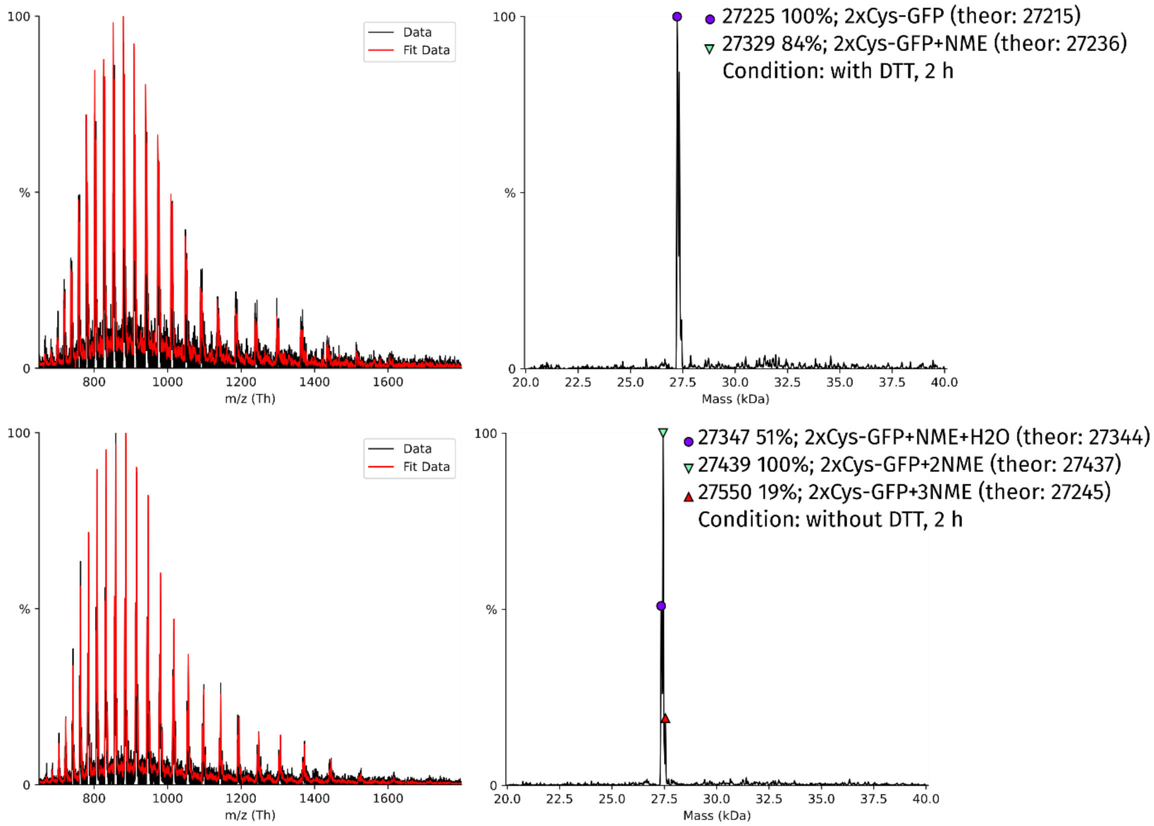

Figure S64. Ion series and deconvoluted mass spectra of the reaction of **2×Cys-GFP** with **NMM** in the presence (top) and absence (bottom) of DTT as described in Section 9.7.4.

9.8 Control to check binding of nIL2-PEG or (nIL2)$_2$ to IL2-R$_\alpha$

In order to test whether the addition of a PEG group in **nIL2-PEG**, or an increase in molecular weight for **(nIL2)$_2$** led to non-specific binding to the α-subunit of the IL2 receptor, a control experiment was performed in which either the biotinylated α-subunit or β-subunit was exposed to the **nIL2** derivative. The concentration of the **nIL2** derivative in both cases was 500 nM. In the case of the β-subunit, strong binding was observed, in accordance with Section 9.5.2. For the α-subunit negligible binding was observed for both species.

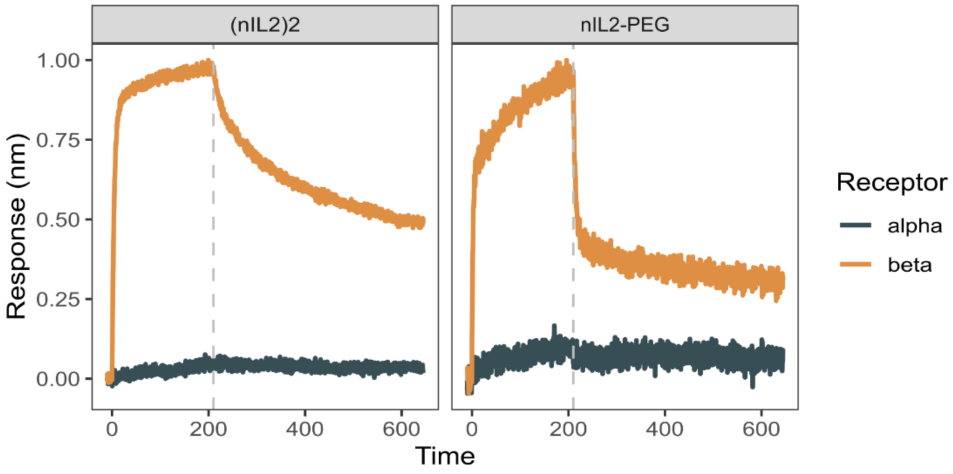

Figure 65. BLI traces for binding of **(nIL2)$_2$** and **nIL2-PEG** to the IL2-Rα and IL2-Rβ subunits respectively.

10 Molecular dynamics (MD) simulations

The crystal structure of GFP (PDB id: 1GFL)[13] was used as starting coordinates for the proteins in the simulations. The missing amino acids in the peptide sequence were added with PyMOL 2.0 (https://pymol.org/2/) and modelled with Chimera.[14] MD simulations were carried out with AMBER 18 package,[15] implemented with ff14SB,[16] and GAFF[17] force fields. Parameters for the unnatural residues were generated with the antechamber module of AMBER, using GAFF force field and with partial charges set to fit the electrostatic potential generated with HF/6-31G(d) by RESP.[18] The charges were calculated according to the Merz-Singh-Kollman scheme using Gaussian 16.[4] Each conjugate was immersed in a water box with a 10 Å buffer of TIP3P water molecules[19] and neutralized by adding explicit Na^+ ions. A two-stage geometry optimization approach was performed. The first stage minimizes only the positions of solvent molecules and ions, and the second stage is an unrestrained minimization of all the atoms in the simulation cell. The systems were then heated by incrementing the temperature from 0 to 300 K under a constant pressure of 1 atm and periodic boundary conditions. Harmonic restraints of 10 kcal·mol^{-1} were applied to the solute, and the Andersen temperature coupling scheme[20] was used to control and equalize the temperature. The time step was kept at 1 fs during the heating stages, allowing potential inhomogeneities to self-adjust. Hydrogen atoms were kept fixed through the simulations using the SHAKE algorithm.[21] Long-range electrostatic effects were modelled using the particle-mesh-Ewald method.[22] An 8 Å cutoff was applied to Lennard-Jones interactions. Each system was equilibrated for 2 ns with a 2-fs time step at a constant volume and temperature of 300 K. Production trajectories were then run for additional 0.5 μs under the same simulation conditions (**Figure S66**).

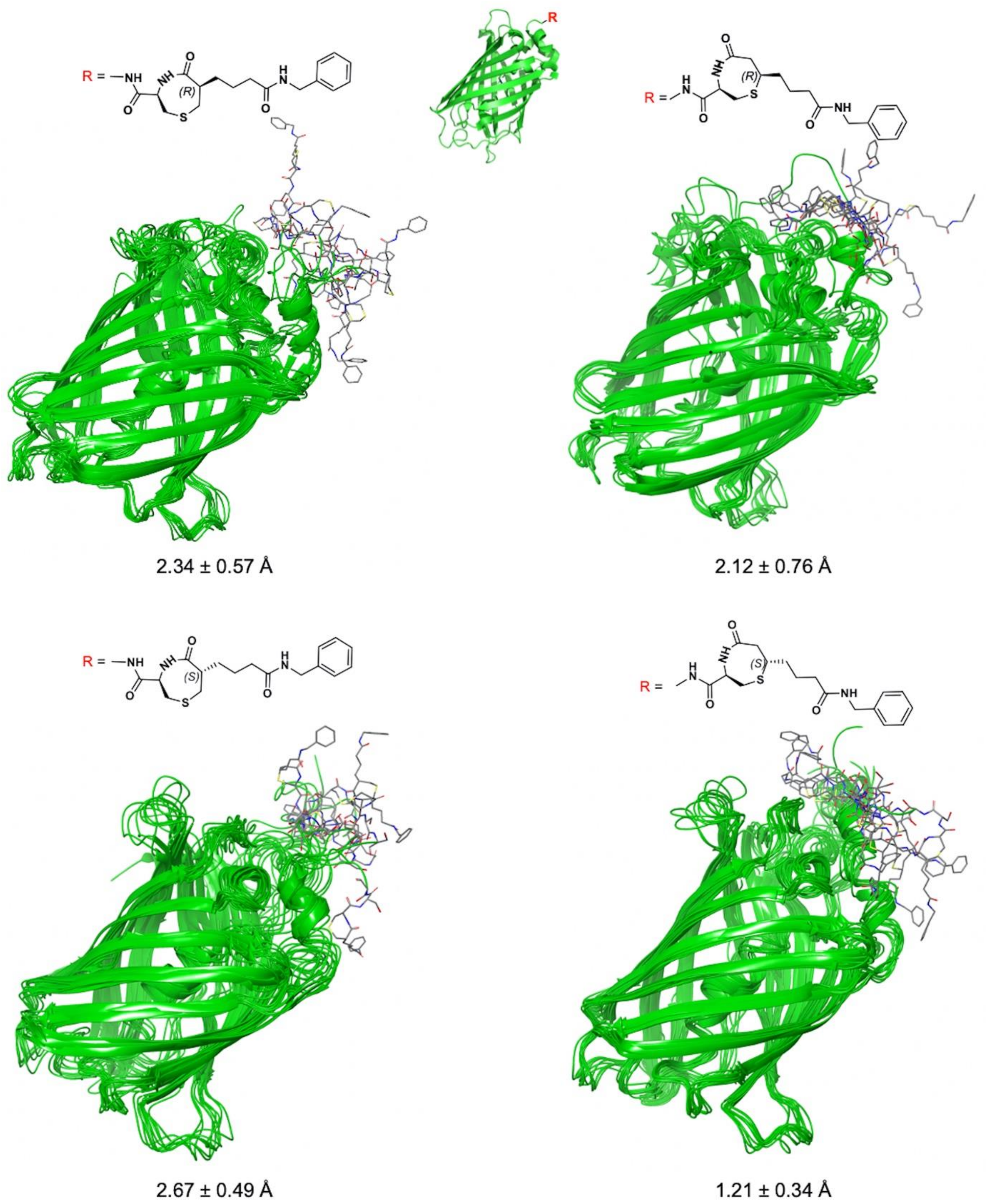

Figure S66. Structural ensembles derived from 0.5 µs MD simulations performed on the four stereoisomers of **GFP-CPO-BN**. The numbers indicate the root-mean-square deviation (RMSD) for heavy-atom superimposition of the backbone of GFP relative to the average structure through the entire trajectory. The low RMSD values indicate that the structure of the proteins remains unaltered upon the chemical modifications, which is essential to retain their biological activity.

11 NMR spectra

^{1}H NMR (400 MHz, $CDCl_3$) of 2-phenethylcycloprop-2-en-1-one **1**

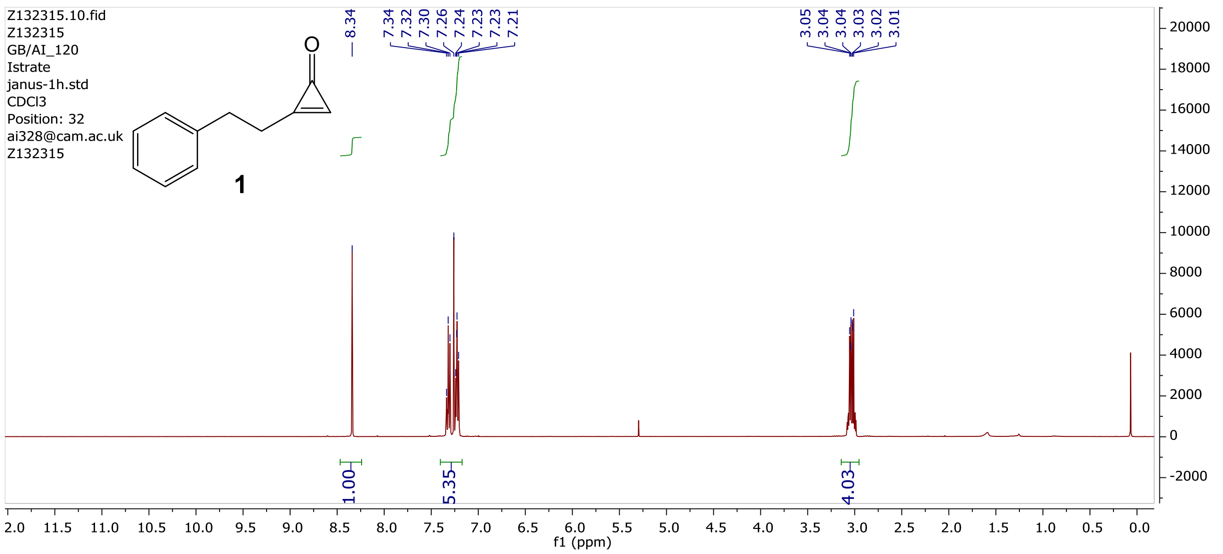

^{13}C NMR ($CDCl_3$, 100 MHz) of 2-phenethylcycloprop-2-en-1-one **1**

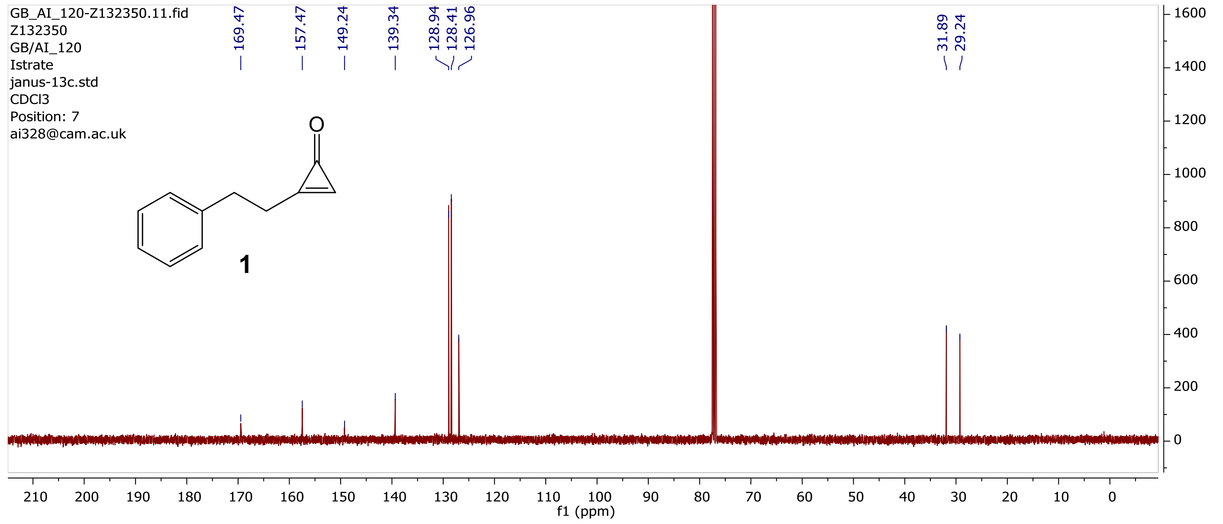

^{1}H NMR (400 MHz, $CDCl_3$) of ethyl (3R,6R)-5-oxo-6-phenethyl-1,4-thiazepane-3-carboxylate **2a**

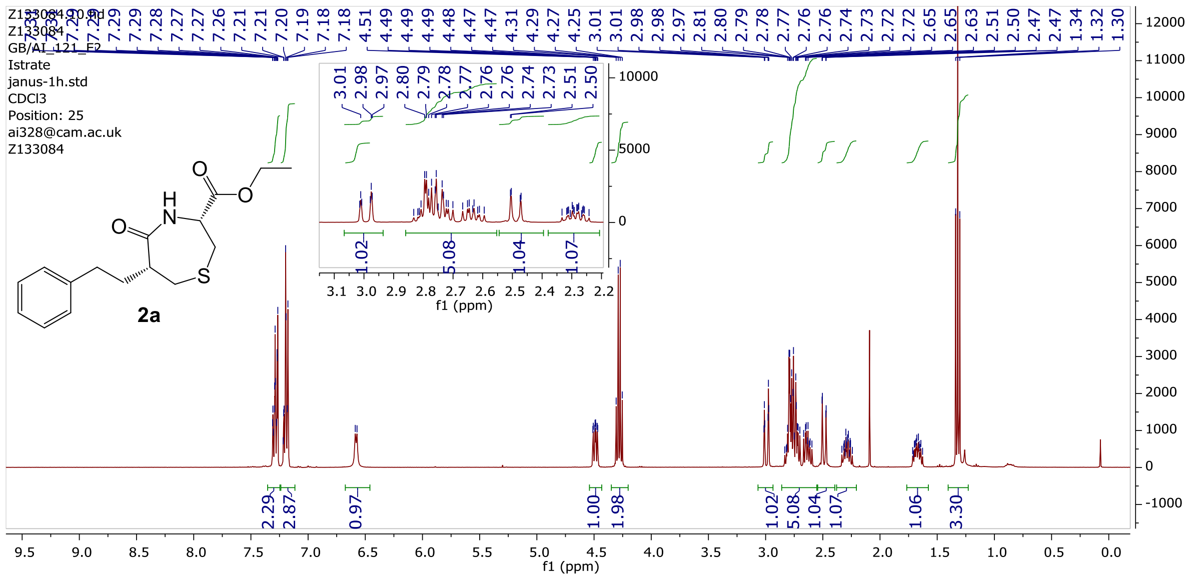

^{13}C NMR ($CDCl_3$, 100 MHz) of ethyl (3R,6R)-5-oxo-6-phenethyl-1,4-thiazepane-3-carboxylate **2a**

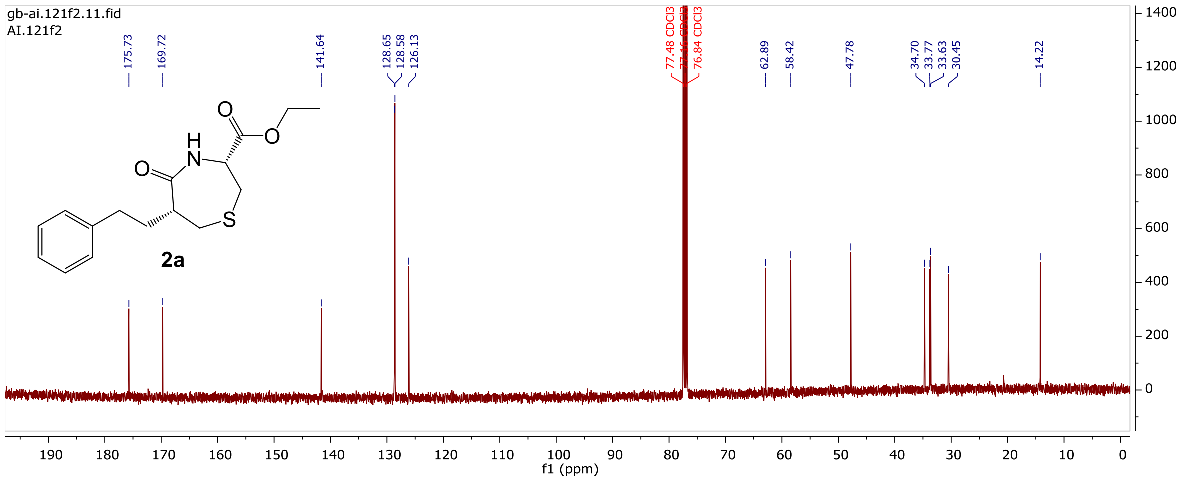

^{1}H NMR (400 MHz, $CDCl_3$) of ethyl (3R,6S)-5-oxo-6-phenethyl-1,4-thiazepane-3-carboxylate **2b**, ethyl (3R,7R)-5-oxo-7-phenethyl-1,4-thiazepane-3-carboxylate **2c**, ethyl (3R,7S)-5-oxo-7-phenethyl-1,4-thiazepane-3-carboxylate **2d**

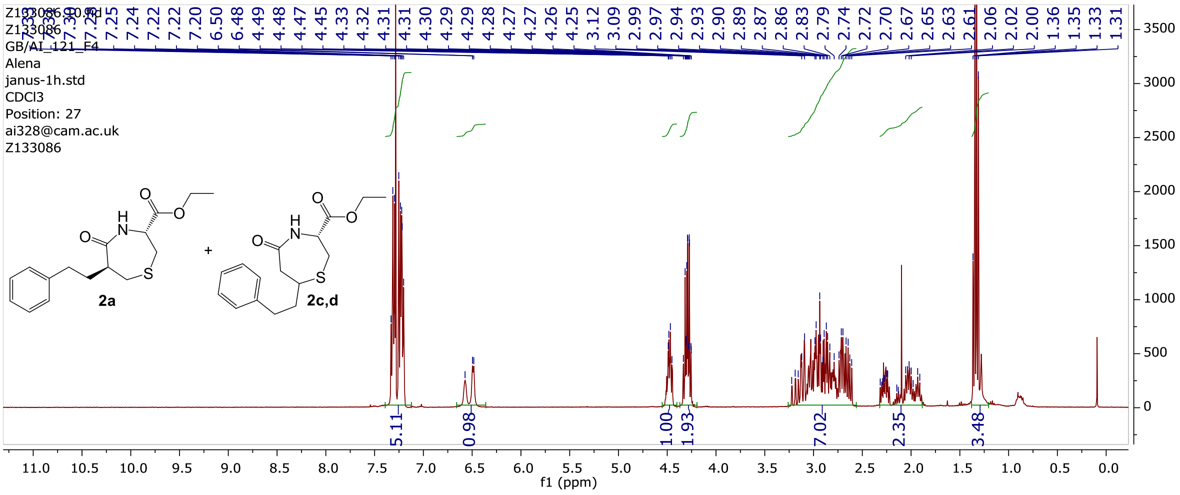

^{13}C NMR ($CDCl_3$, 100 MHz) of ethyl (3R,6S)-5-oxo-6-phenethyl-1,4-thiazepane-3-carboxylate **2b**, ethyl (3R,7R)-5-oxo-7-phenethyl-1,4-thiazepane-3-carboxylate **2c**, ethyl (3R,7S)-5-oxo-7-phenethyl-1,4-thiazepane-3-carboxylate **2d**

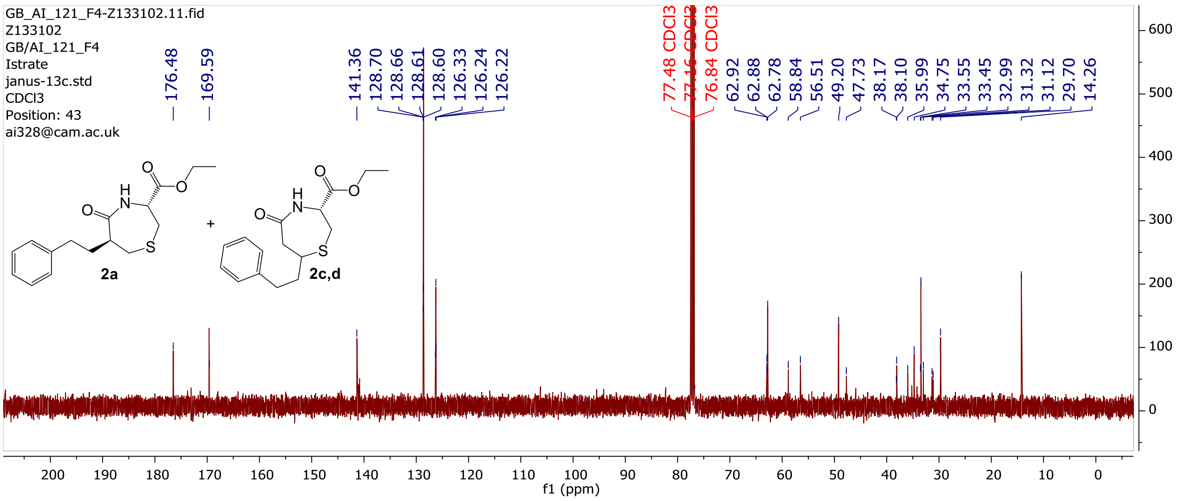

^{1}H NMR (400 MHz, $CDCl_3$) of 6-phenethyl-1,4-thiazepan-5-one **3a,b** (both enantiomers)

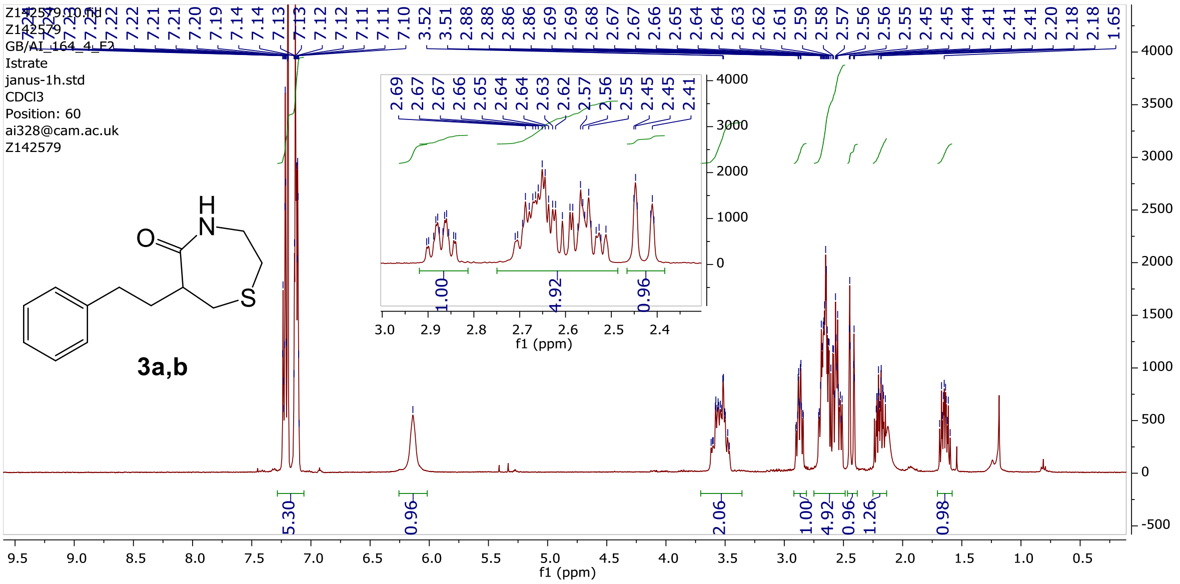

^{13}C NMR ($CDCl_3$, 100 MHz) of 6-phenethyl-1,4-thiazepan-5-one **3a,b** (both enantiomers)

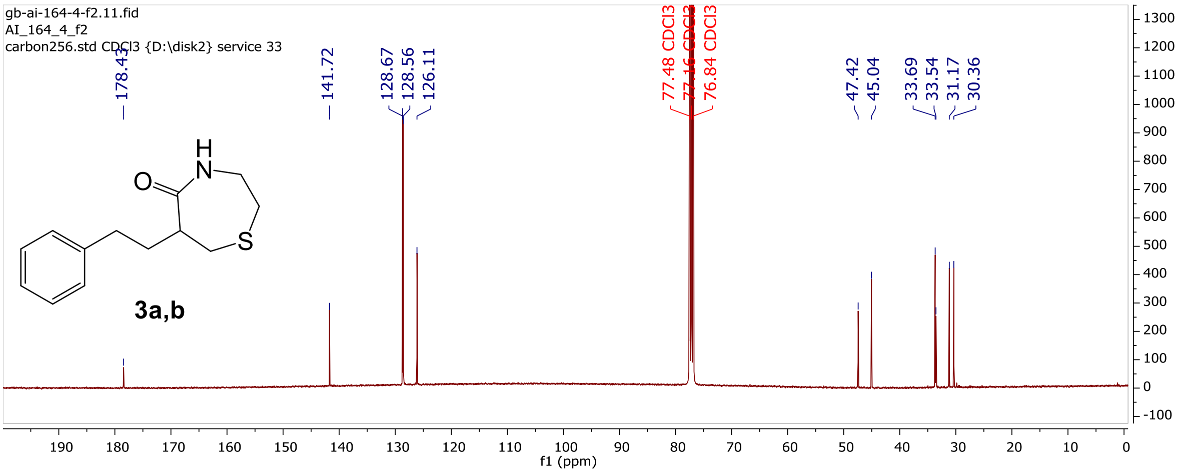

^{1}H NMR (400 MHz, $CDCl_3$) of 7-phenethyl-1,4-thiazepan-5-one **3c,d** (both enantiomers)

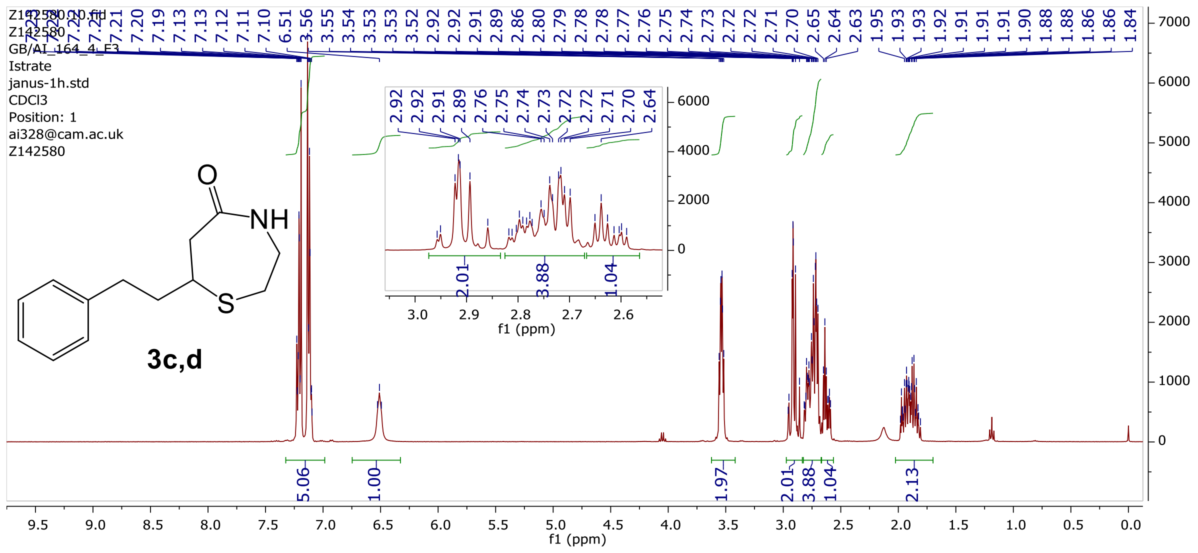

^{13}C NMR ($CDCl_3$, 100 MHz) of 7-phenethyl-1,4-thiazepan-5-one **3c,d** (both enantiomers)

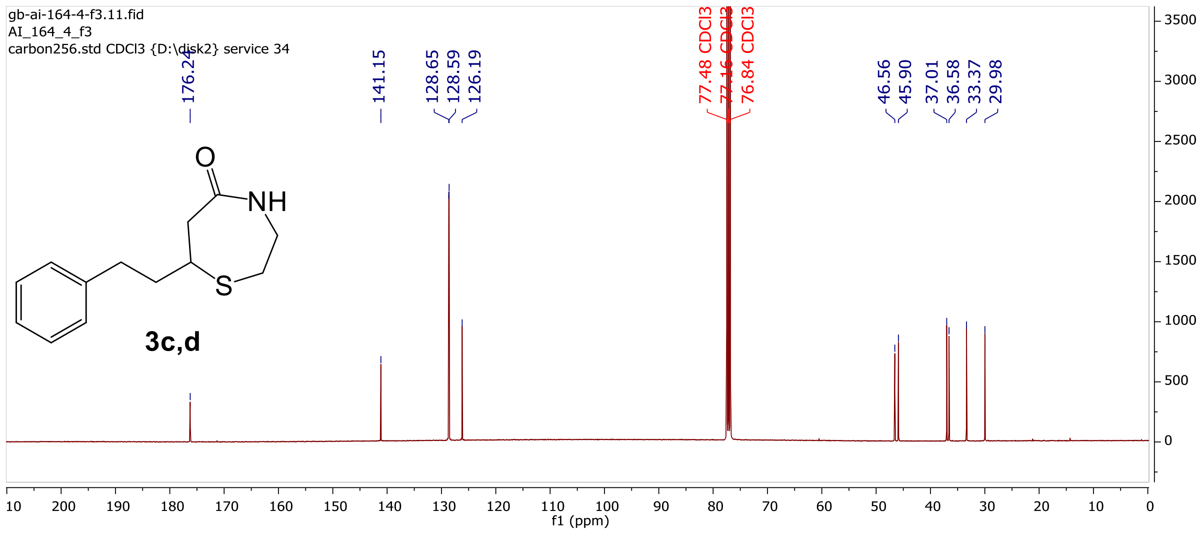

^{1}H NMR (400 MHz, $CDCl_3$) of perfluorophenyl hex-5-ynoate **5**

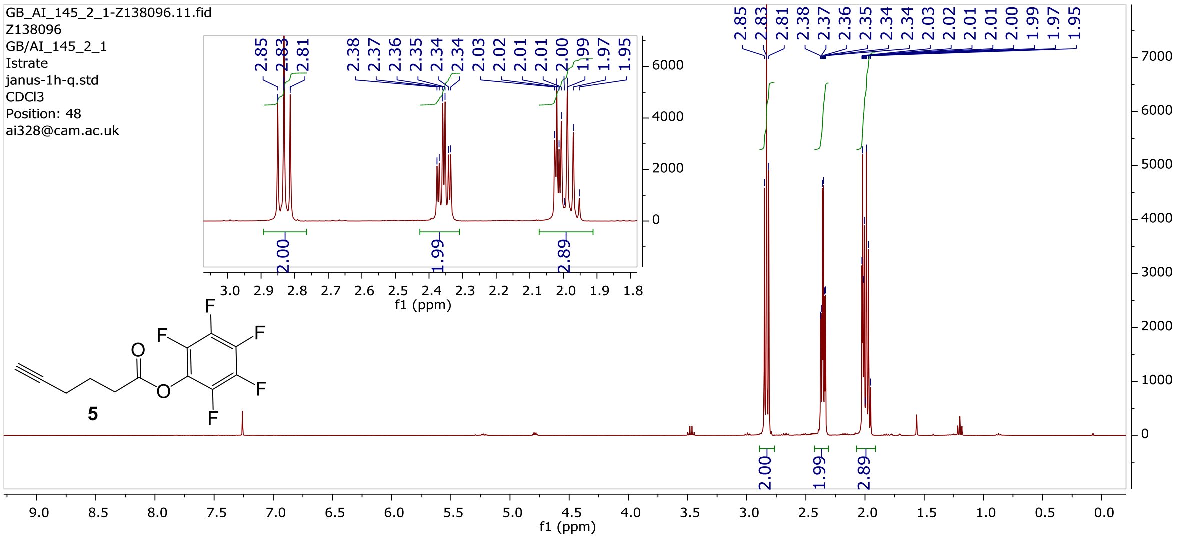

^{13}C NMR (100 MHz, $CDCl_3$) of perfluorophenyl hex-5-ynoate **5**

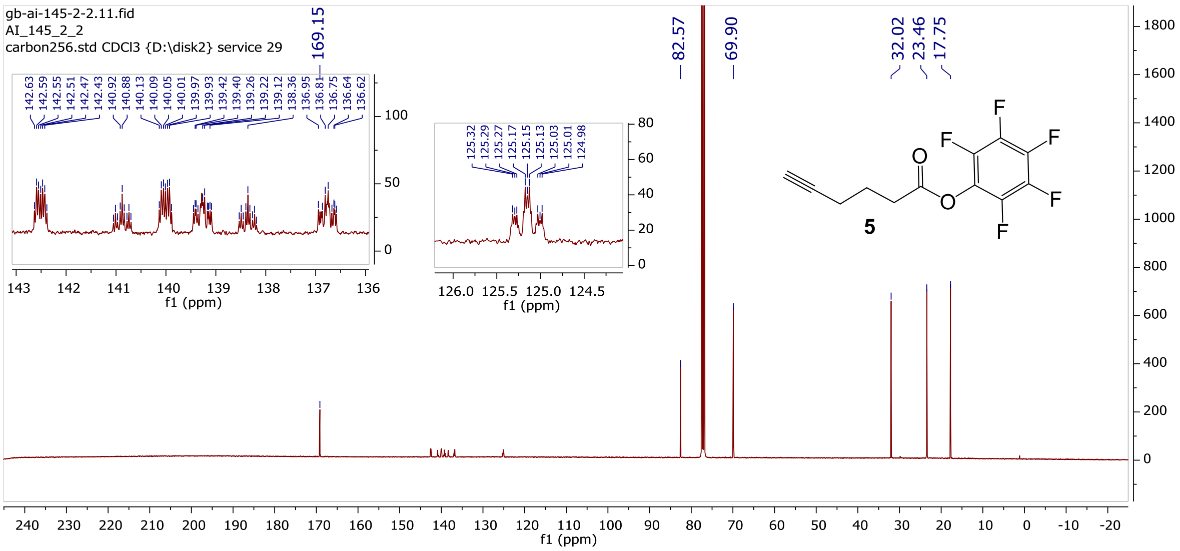

^{19}F NMR (376.5 MHz, $CDCl_3$) of perfluorophenyl hex-5-ynoate **5**

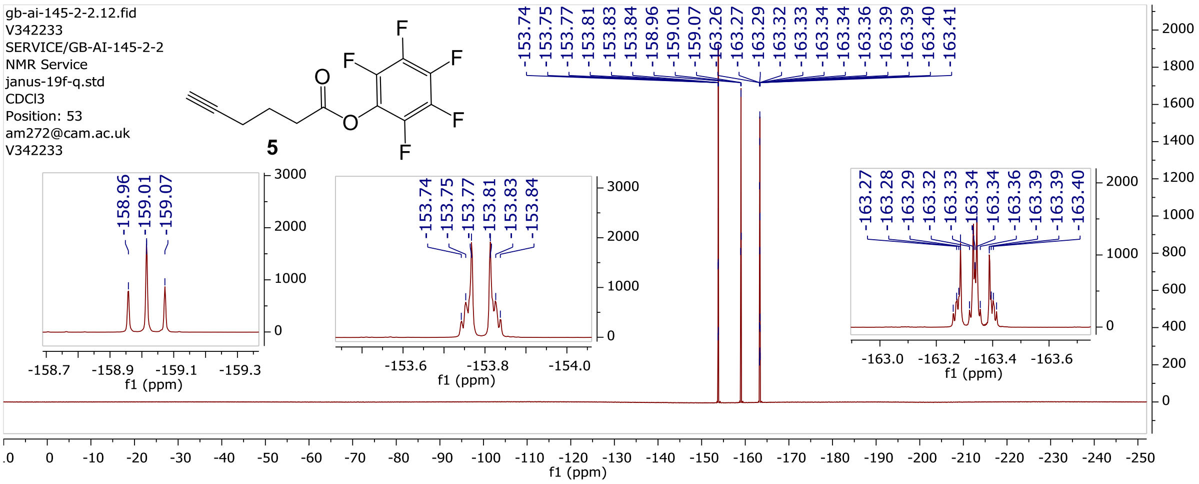

^{1}H NMR (500 MHz, $CDCl_3$) of perfluorophenyl 4-(3'-oxocycloprop-1'-en-1'-yl)butanoate **6**

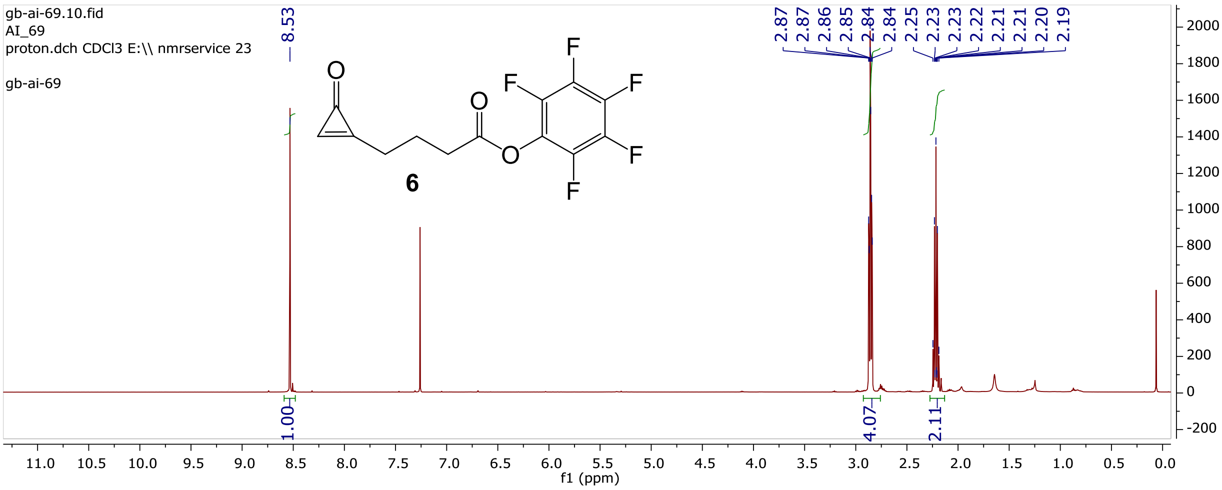

^{13}C NMR (125 MHz, $CDCl_3$) of Perfluorophenyl 4-(3'-oxocycloprop-1'-en-1'-yl)butanoate **6**

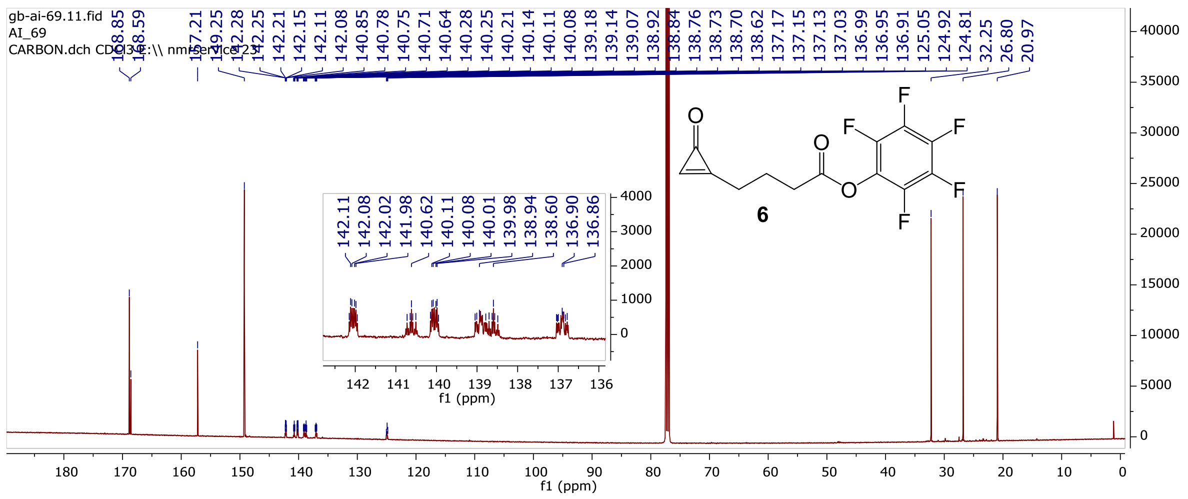

^{19}F NMR (376.5 MHz, $CDCl_3$) of Perfluorophenyl 4-(3'-oxocycloprop-1'-en-1'-yl)butanoate **6**

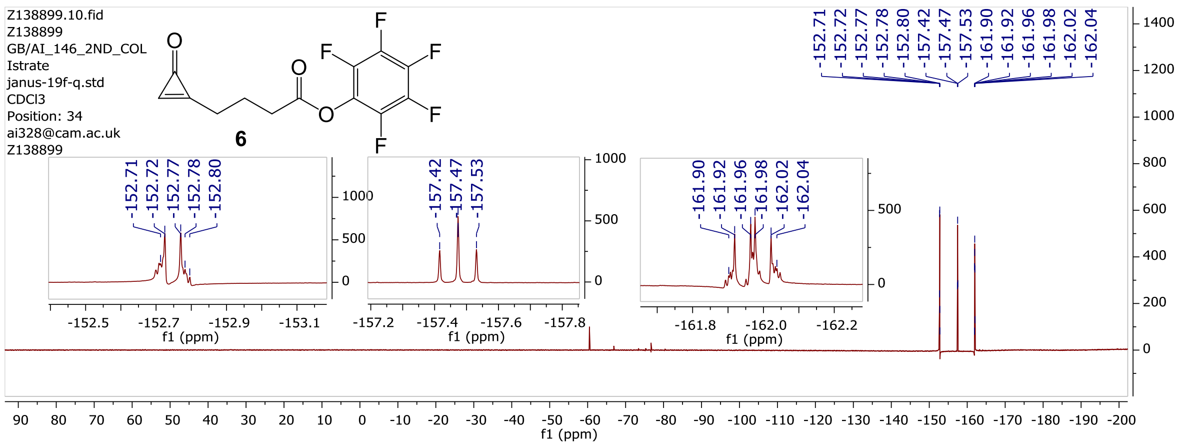

^{1}H NMR (400 MHz, $CDCl_3$) of 4-(3'-oxocycloprop-1'-en-1'-yl)-N-phenylbutanamide **CPO-BN**

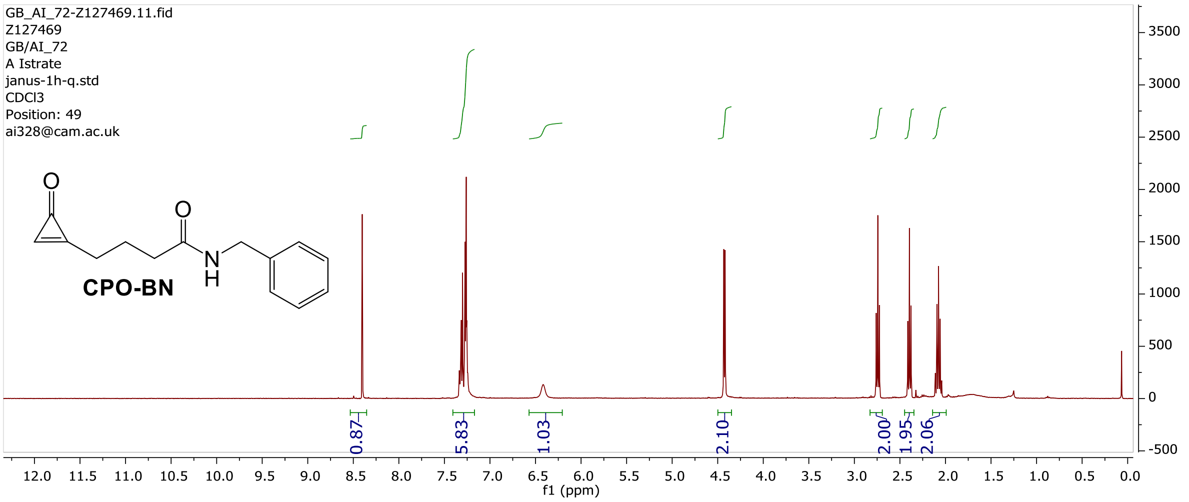

^{13}C NMR (125 MHz, $CDCl_3$) of 4-(3'-oxocycloprop-1'-en-1'-yl)-N-phenylbutanamide **CPO-BN**

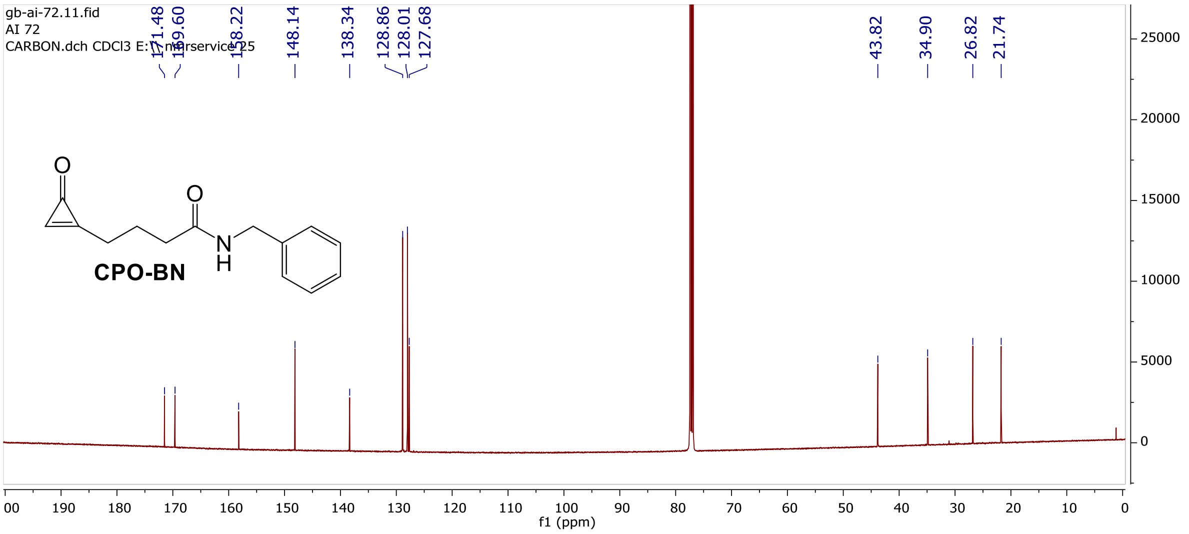

^{1}H NMR (400 MHz, $CDCl_3$) of N-(2-(2-(2-(2-hydroxyethoxy)ethoxy)ethoxy)ethyl)-4-(3'-oxocycloprop-1'-en-1'-yl)butanamide **CPO-PEG**

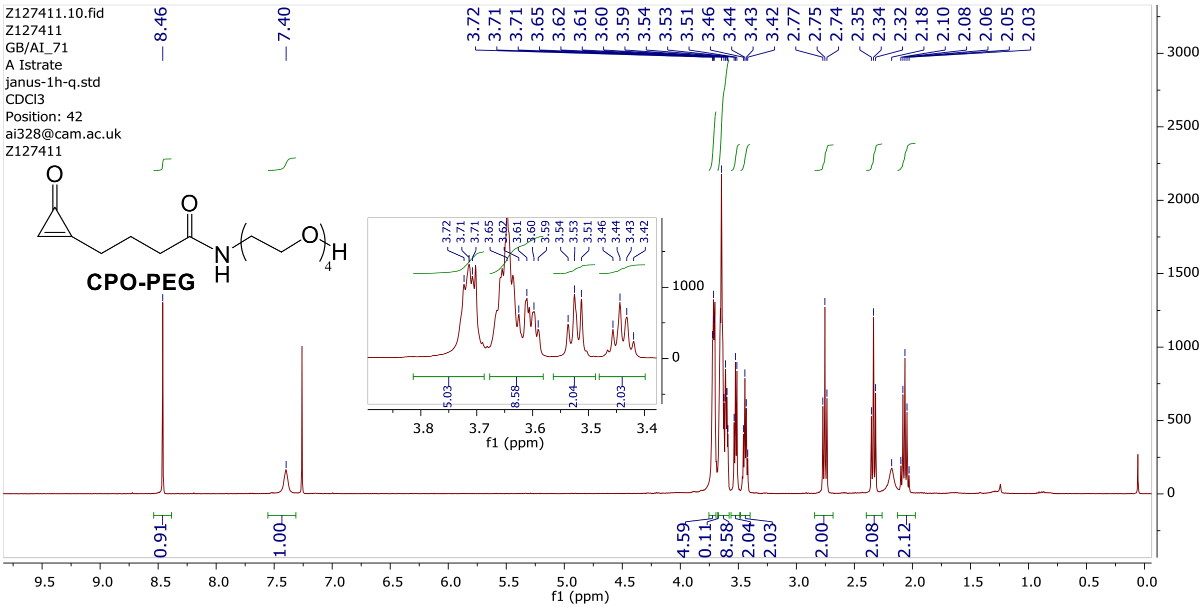

^{13}C NMR (125 MHz, $CDCl_3$) of N-(2-(2-(2-(2-hydroxyethoxy)ethoxy)ethoxy)ethyl)-4-(3'-oxocycloprop-1'-en-1'-yl)butanamide **CPO-PEG**

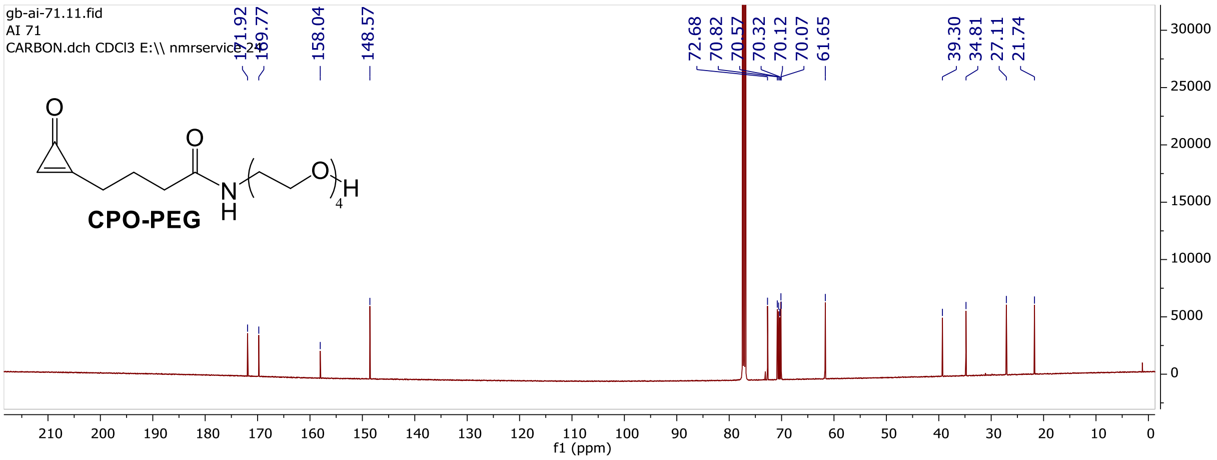

[13]H NMR (400 MHz, $CDCl_3$) of 4-(3’-oxocycloprop-1’-en-1’-yl)-N-(3’’,6’’,9’’,12’’-tetraoxapentadec-14’’-yn-1’’-yl)butanamide **CPO-PEG-Alkyne**

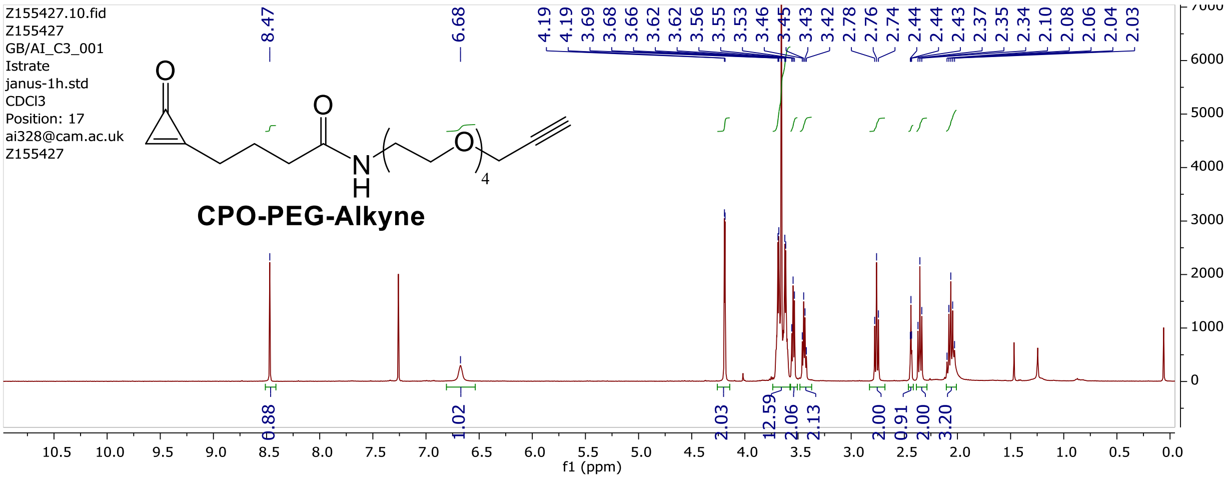

^{13}C NMR (100 MHz, $CDCl_3$) of 4-(3’-oxocycloprop-1’-en-1’-yl)-N-(3’’,6’’,9’’,12’’-tetraoxapentadec-14’’-yn-1’’-yl)butanamide **CPO-PEG-Alkyne**

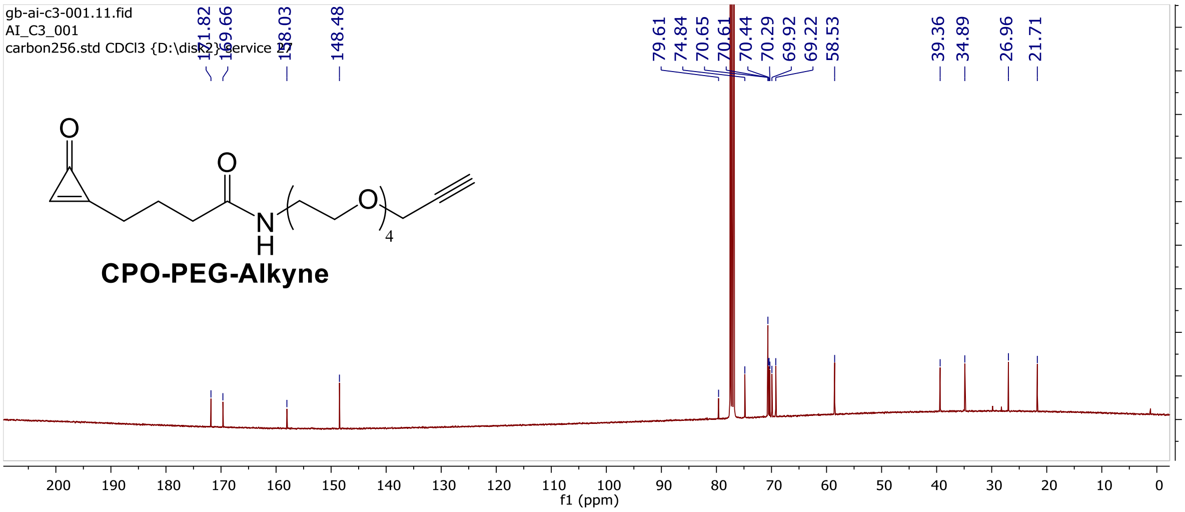

^{13}H NMR (500 MHz, DMF-d_7) of Sodium 5-((2'-(4''-(3'''-oxocycloprop-1'''-en-1'''-yl)butanamido)ethyl)amino) naphthalene-1-sulfonate **CPO-PEG-EDANS**

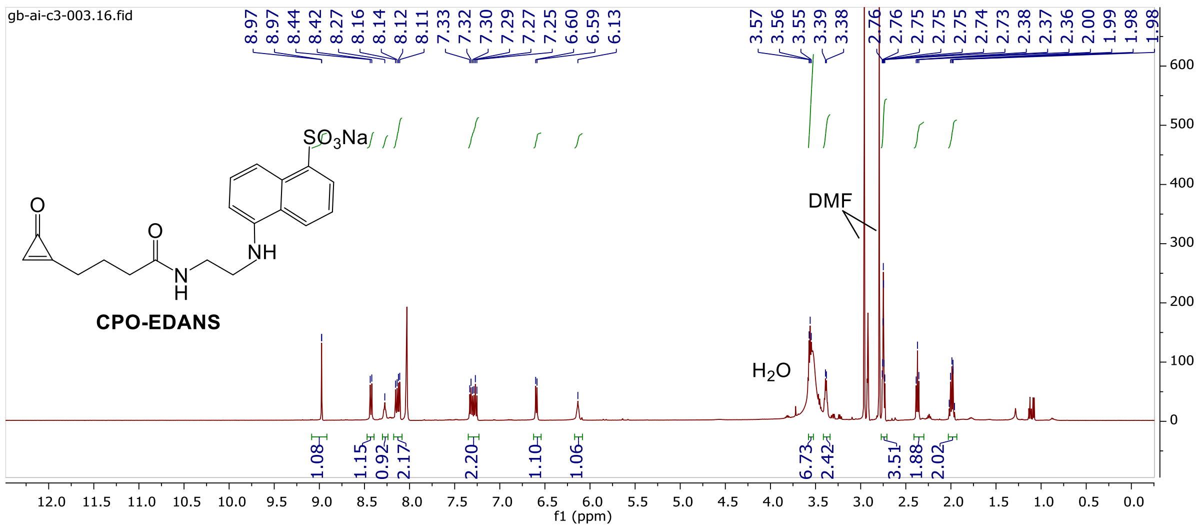

^{13}C NMR (126 MHz, $CDCl_3$) of Sodium 5-((2'-(4''-(3'''-oxocycloprop-1'''-en-1'''-yl)butanamido)ethyl)amino) naphthalene-1-sulfonate **CPO-PEG-EDANS**

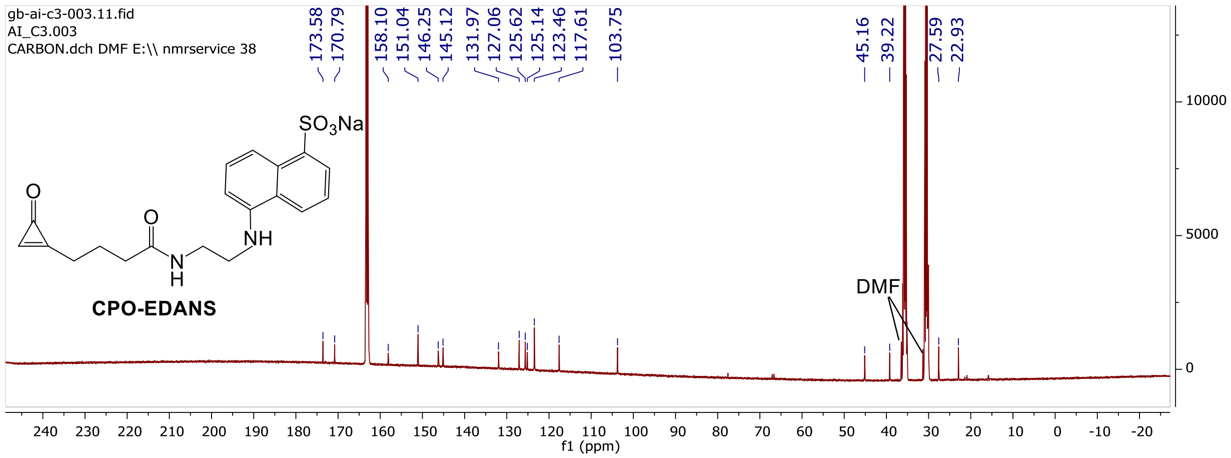

^{1}H NMR spectrum of **CPO-DBCO** in $CDCl_3$ recorded at 400 MHz and 25 ºC.

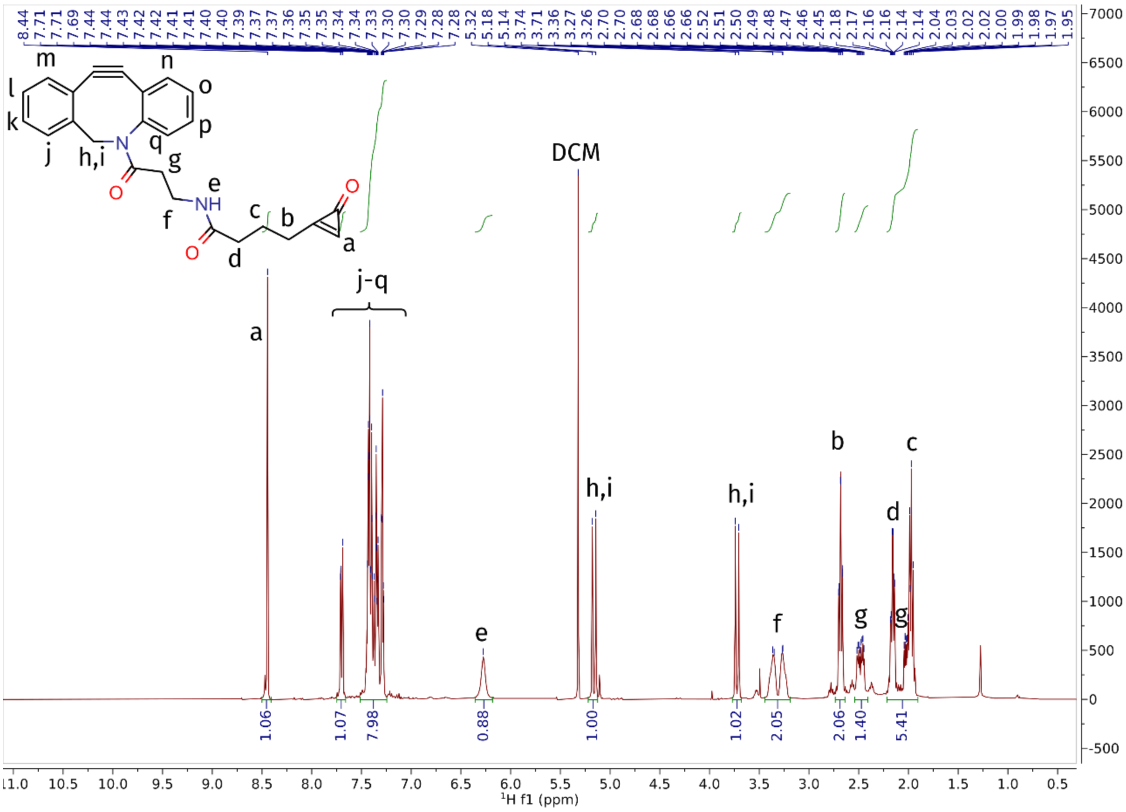

^{13}C NMR spectrum of **CPO-DBCO** in $CDCl_3$ recorded at 101 MHz and 25 ºC.

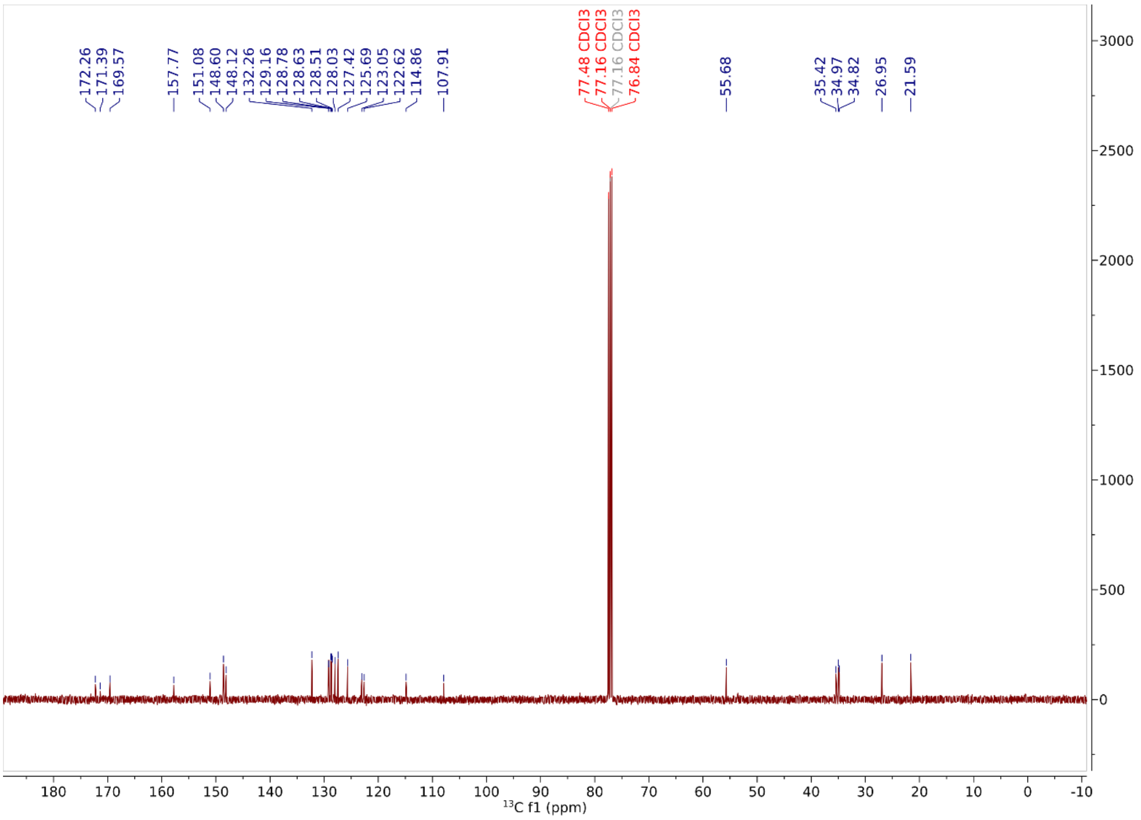

^{1}H NMR spectrum of **CPO-N$_3$** in CDCl$_3$ recorded at 400 MHz and 25 ºC.

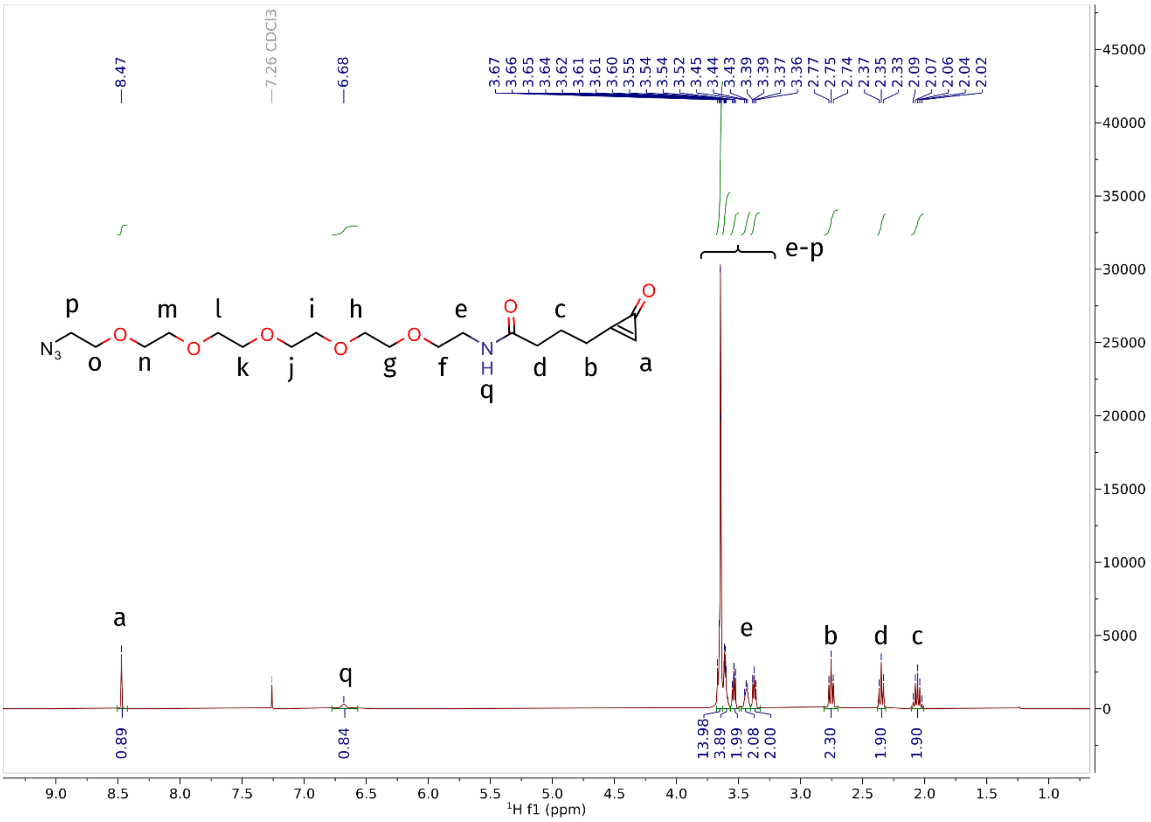

^{13}C NMR spectrum of **CPO-N$_3$** in CDCl$_3$ recorded at 101 MHz and 25 ºC.

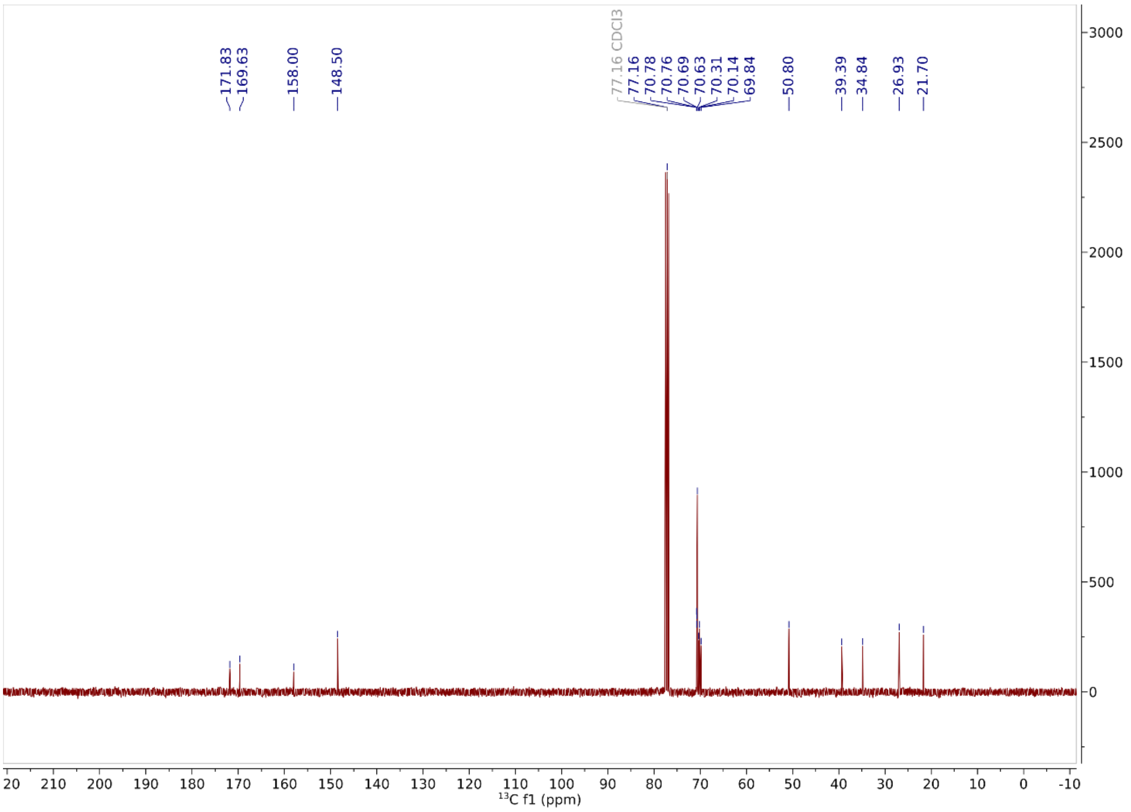

^1H NMR spectrum of **CAA-BN** in $CDCl_3$ recorded at 400 MHz and 25 ºC.

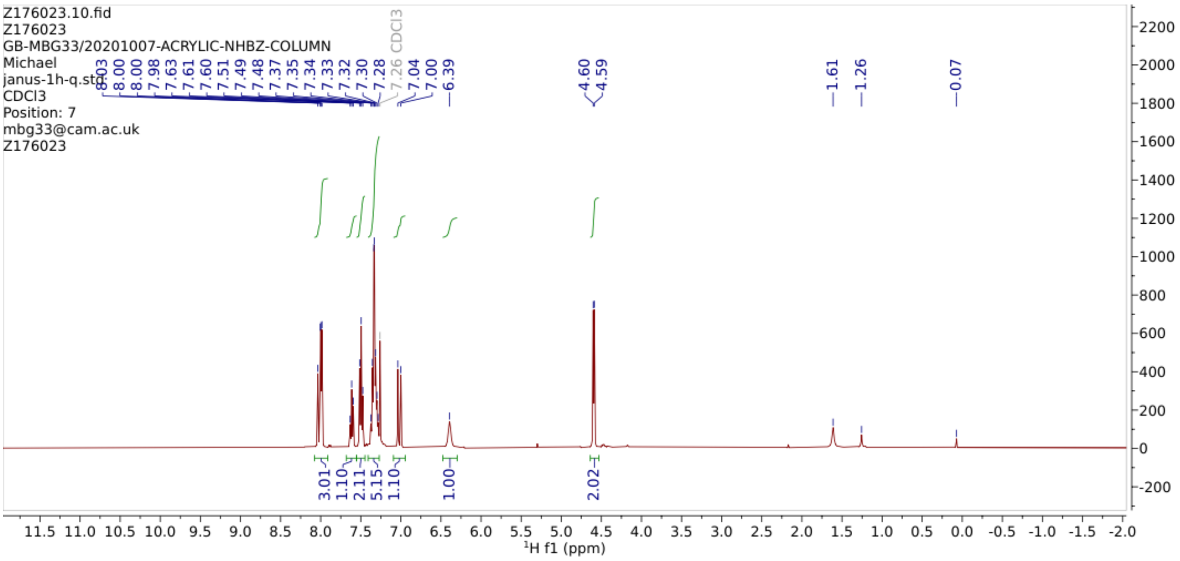

12 References

1. Li, Z.; Wang, Y.; Tang, C.; Xu, J.; Wu, X.; Yao, H., Synthesis of 3-Benzoyl Acrylates/Acrylamides via Dehydrogenation of 3-Benzoyl Propionates/Propionamides Using IBX/p-TsOH. Chin. J. Chem., 2010; Vol. 28, pp 1301–1305.

2. Sheldrick, G., Crystal structure refinement with SHELXL. *Acta Crystallogr. C* **2015,** *71* (1), 3 – 8.

3. Sheldrick, G., SHELXT - Integrated space-group and crystal-structure determination. *Acta Crystallogr. A* **2015,** *71* (1), 3 – 8.

4. Frisch, M. J.; Trucks, G. W.; Schlegel, H. B.; Scuseria, G. E.; Robb, M. A.; Cheeseman, J. R.; Scalmani, G.; Barone, V.; Petersson, G. A.; Nakatsuji, H.; Li, X.; Caricato, M.; Marenich, A. V.; Bloino, J.; Janesko, B. G.; Gomperts, R.; Mennucci, B.; Hratchian, H. P.; Ortiz, J. V.; Izmaylov, A. F.; Sonnenberg, J. L.; Williams; Ding, F.; Lipparini, F.; Egidi, F.; Goings, J.; Peng, B.; Petrone, A.; Henderson, T.; Ranasinghe, D.; Zakrzewski, V. G.; Gao, J.; Rega, N.; Zheng, G.; Liang, W.; Hada, M.; Ehara, M.; Toyota, K.; Fukuda, R.; Hasegawa, J.; Ishida, M.; Nakajima, T.; Honda, Y.; Kitao, O.; Nakai, H.; Vreven, T.; Throssell, K.; Montgomery Jr., J. A.; Peralta, J. E.; Ogliaro, F.; Bearpark, M. J.; Heyd, J. J.; Brothers, E. N.; Kudin, K. N.; Staroverov, V. N.; Keith, T. A.; Kobayashi, R.; Normand, J.; Raghavachari, K.; Rendell, A. P.; Burant, J. C.; Iyengar, S. S.; Tomasi, J.; Cossi, M.; Millam, J. M.; Klene, M.; Adamo, C.; Cammi, R.; Ochterski, J. W.; Martin, R. L.; Morokuma, K.; Farkas, O.; Foresman, J. B.; Fox, D. J. *Gaussian 16 Rev. B.01*, Wallingford, CT, 2016.

5. Zhao, Y.; Truhlar, D. G., The M06 suite of density functionals for main group thermochemistry, thermochemical kinetics, noncovalent interactions, excited states, and transition elements: two new functionals and systematic testing of four M06-class functionals and 12 other functionals. *Theor. Chem. Acc.* **2008,** *120* (1), 215 – 241.

6. Scalmani, G.; Frisch, M. J., Continuous surface charge polarizable continuum models of solvation. I. General formalism. *J. Chem. Phys.* **2010,** *132* (11), 114110.

7. Ribeiro, R. F.; Marenich, A. V.; Cramer, C. J.; Truhlar, D. G., Use of Solution-Phase Vibrational Frequencies in Continuum Models for the Free Energy of Solvation. *J. Phys. Chem. B.* **2011,** *115* (49), 14556 – 14562.

8. Hratchian, H. P.; Schlegel, H. B., Accurate reaction paths using a Hessian based predictor-corrector integrator. *J. Chem. Phys.* **2004,** *120*, 9918 – 9924.

9. Ren, H.; Xiao, F.; Zhan, K.; Kim, Y.-P.; Xie, H.; Xia, Z.; Rao, J., A Biocompatible Condensation Reaction for the Labeling of Terminal Cysteine Residues on Proteins. *Angew. Chem. Int. Ed.* **2009,** *48* (51), 9658 – 9662.

10. Alam, I. S.; Neves, A. A.; Witney, T. H.; Boren, J.; Brindle, K. M., Comparison of the C2A Domain of Synaptotagmin-I and Annexin-V As Probes for Detecting Cell Death. *Bioconjug. Chem.* **2010,** *21* (5), 884 – 891.

11. Freedy, A. M.; Matos, M. J.; Boutureira, O.; Corzana, F.; Guerreiro, A.; Akkapeddi, P.; Somovilla, V. J.; Rodrigues, T.; Nicholls, K.; Xie, B.; Jiménez-Osés, G.; Brindle, K. M.; Neves, A. A.; Bernardes, G. J. L., Chemoselective Installation of Amine Bonds on Proteins through Aza-Michael Ligation. *J. Am. Chem. Soc.* **2017,** *139* (50), 18365 – 18375.

12. Aprile, F. A.; Sormanni, P.; Perni, M.; Arosio, P.; Linse, S.; Knowles, T. P. J.; Dobson, C. M.; Vendruscolo, M., Selective targeting of primary and secondary nucleation pathways in Aβ42 aggregation using a rational antibody scanning method. *Sci. Adv.* **2017,** *3* (6), e1700488.

13. Yang, F.; Moss, L. G.; Phillips, G. N., The molecular structure of green fluorescent protein. *Nat. Biotechnol.* **1996,** *14* (10), 1246 – 1251.

14. Pettersen, E. F.; Goddard, T. D.; Huang, C. C.; Couch, G. S.; Greenblatt, D. M.; Meng, E. C.; Ferrin, T. E., UCSF Chimera—A visualization system for exploratory research and analysis. *J. Comput. Chem.* **2004,** *25* (13), 1605 – 1612.

15. D.A. Case, I. Y. B.-S., S.R. Brozell, D.S. Cerutti, T.E. Cheatham, III, V.W.D. Cruzeiro, T.A. Darden, R.E. Duke, D. Ghoreishi, M.K. Gilson, H. Gohlke, A.W. Goetz, D. Greene, R Harris, N. Homeyer, S. Izadi, A. Kovalenko, T. Kurtzman, T.S. Lee, S. LeGrand, P. Li, C. Lin, J. Liu, T. Luchko, R. Luo, D.J. Mermelstein, K.M. Merz, Y. Miao, G. Monard, C. Nguyen, H. Nguyen, I. Omelyan, A. Onufriev, F. Pan, R. Qi, D.R. Roe, A. Roitberg, C. Sagui, S. Schott-Verdugo, J. Shen, C.L. Simmerling, J. Smith, R. Salomon-Ferrer, J. Swails, R.C. Walker, J. Wang, H. Wei, R.M. Wolf, X. Wu, L. Xiao, D.M. York and P.A. Kollman, AMBER 2018, University of California, San Francisco. **2018**.

16. Maier, J. A.; Martinez, C.; Kasavajhala, K.; Wickstrom, L.; Hauser, K. E.; Simmerling, C., ff14SB: Improving the Accuracy of Protein Side Chain and Backbone Parameters from ff99SB. *J. Chem. Theory Comput.* **2015,** *11* (8), 3696-3713.

17. Wang, J.; Wolf, R. M.; Caldwell, J. W.; Kollman, P. A.; Case, D. A., Development and testing of a general amber force field. *J. Comput. Chem.* **2004,** *25* (9), 1157-1174.

18. Bayly, C. I.; Cieplak, P.; Cornell, W.; Kollman, P. A., A well-behaved electrostatic potential based method using charge restraints for deriving atomic charges: the RESP model. *J. Phys. Chem.* **1993,** *97* (40), 10269-10280.

19. Jorgensen, W. L.; Chandrasekhar, J.; Madura, J. D.; Impey, R. W.; Klein, M. L., Comparison of simple potential functions for simulating liquid water. *J. Chem. Phys.* **1983,** *79* (2), 926-935.

20. Andersen, H. C., Molecular dynamics simulations at constant pressure and/or temperature. *J. Chem. Phys.* **1980,** *72* (4), 2384-2393.

21. Miyamoto, S.; Kollman, P. A., Settle: An analytical version of the SHAKE and RATTLE algorithm for rigid water models. *J. Comput. Chem.* **1992,** *13* (8), 952-962.

22. Darden, T.; York, D.; Pedersen, L., Particle mesh Ewald: An N·log(N) method for Ewald sums in large systems. *J. Chem. Phys.* **1993,** *98* (12), 10089-10092.
